# Planar Core and Macrocyclic Shell Stabilized Atomically Precise Copper Nanocluster Catalyst for Efficient Hydroboration of C–C Multiple Bond

**DOI:** 10.1021/jacs.4c05077

**Published:** 2024-05-30

**Authors:** Badriah Alamer, Arunachalam Sagadevan, Mohammad Bodiuzzaman, Kathiravan Murugesan, Salman Alsharif, Ren-Wu Huang, Atanu Ghosh, Malenahalli H. Naveen, Chunwei Dong, Saidkhodzha Nematulloev, Jun Yin, Aleksander Shkurenko, Mutalifu Abulikemu, Xinglong Dong, Yu Han, Mohamed Eddaoudi, Magnus Rueping, Osman M. Bakr

**Affiliations:** † KAUST Catalysis Center (KCC), Division of Physical Sciences and Engineering, King Abdullah University of Science and Technology (KAUST), Thuwal 23955-6900, Saudi Arabia; ‡ Department of Chemistry, College of Science, Taif University, P.O. Box 11099, Taif 21944, Saudi Arabia; § Henan Key Laboratory of Crystalline Molecular Functional Materials, Green Catalysis Center, College of Chemistry, Henan International Joint Laboratory of Tumor Theranostic Cluster Materials, Zhengzhou University, Zhengzhou 450001, China; ∥ Department of Applied Physics, The Hong Kong Polytechnic University, Hung Hom, Kowloon, 999077 Hong Kong, P. R. China; ⊥ Division of Physical Sciences and Engineering and Functional Materials Design, Discovery and Development Research Group (FMD3), Advanced Membranes and Porous Materials Center, King Abdullah University of Science and Technology (KAUST), Thuwal 23955-6900, Saudi Arabia; # Advanced Membranes and Porous Materials Center, Physical Sciences and Engineering Division, King Abdullah University of Science and Technology (KAUST), Thuwal 23955-6900, Saudi Arabia; ∇ Institute for Organic and Bimolecular Chemistry, Georg-August-University Goettingen Tammannstr, 237077 Goettingen, Germany

## Abstract

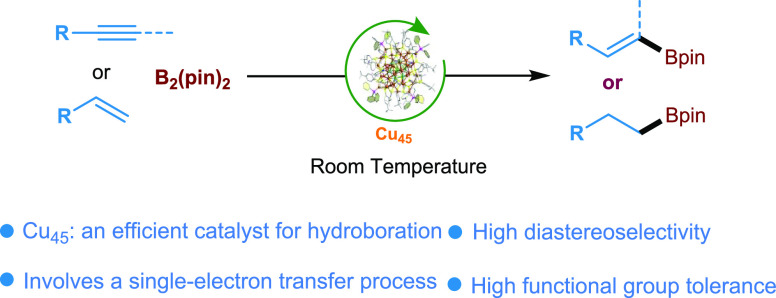

Atomically precise metal nanoclusters (NCs) have become an important class of catalysts due to their catalytic activity, high surface area, and tailored active sites. However, the design and development of bond-forming reaction catalysts based on copper NCs are still in their early stages. Herein, we report the synthesis of an atomically precise copper nanocluster with a planar core and unique shell, [Cu_45_(TBBT)_29_(TPP)_4_(C_4_H_11_N)_2_H_14_]^2+^ (**Cu_45_**) (TBBT: 4-*tert*-butylbenzenethiol; TPP: triphenylphosphine), in high yield via a one-pot reduction method. The resulting structurally well-defined **Cu_45_** is a highly efficient catalyst for the hydroboration reaction of alkynes and alkenes. Mechanistic studies show that a single-electron oxidation of the in situ-formed ate complex enables the hydroboration via the formation of boryl-centered radicals under mild conditions. This work demonstrates the promise of tailored copper nanoclusters as catalysts for C–B heteroatom bond-forming reactions. The catalysts are compatible with a wide range of alkynes and alkenes and functional groups for producing hydroborated products.

## Introduction

The pursuit of efficient catalysts characterized by high stability and catalytic performance is a formidable challenge driven by the need to meet the demands of contemporary chemical manufacturing processes.^1−5^ In recent years, atomically precise ligand-protected metal nanoclusters (NCs) have emerged as promising candidates for catalysis applications.^6−11^ The precision of their defined structures and the adjustability of their sizes enable the modification of catalytic performance and selectivity.^12,13^ In particular, the surface morphology of high-nuclearity NCs with core–shell structures composed of multiple metal-containing motifs was shown to improve catalytic properties.^14−16^ The intricate nature of the interfaces within these structures may result in the formation of distinct active sites tailored for specific reactants.^6,17−19^

The nanocluster family has grown in diversity, with most research focusing on coinage metals, i.e., gold, silver, and, more recently, copper.^10,11,20−23^ The inclusion of copper within the nanocluster family offers cost-reduction advantages and opens new opportunities for applications in sustainable synthesis and catalysis^18,19,24−28^ due to its easily accessible oxidation states and flexible ligand architectures.^29,30^ However, developing new core–shell Cu NCs and understanding their catalytic bond-forming reactions remain in their infancy. To date, only a handful of high-nuclearity Cu NCs have been used as catalysts for a few sets of organic transformations such as click chemistry,^25^ hydrogenations of ketones,^31^ and photocatalytic C–C/C–N cross-couplings.^18,19^ This may be attributed to the significant challenges associated with synthesizing large core–shell copper nanoclusters, including their flexible coordination modes, complex cuprophilic interactions, and susceptibility to aerial oxidation.^32^

Herein, we present a novel core–shell atomically precise nanocluster formulated as [Cu_45_(TBBT)_29_(TPP)_4_(C_4_H_11_N)_2_H_14_]^2+^ (**Cu**_**45**_) TBBT: 4-*tert*-butylbenzenethiol; TPP: triphenylphosphine. The nanocluster possesses a rare planar hexagonal Cu_7_ core, which is fully encapsulated in the Cu skeleton and surrounded by a unique cage-like Cu_38_(TBBT)_29_(PPh_3_)_4_N_2_ shell structure. The Cu_38_ shell is comprised of two distinct layers, namely, Cu_14_(TBBT)_5_N_2_ on the top and Cu_10_(TBBT)_8_ at the bottom, linked by a macrocyclic ring of Cu_14_(TBBT)_16_P_4._ The NC’s diverse shell framework inspired us to explore its potential as a catalyst in C–B *heteroatom* bond-forming reactions via a single-electron transfer (SET) process.

**Scheme 1:**
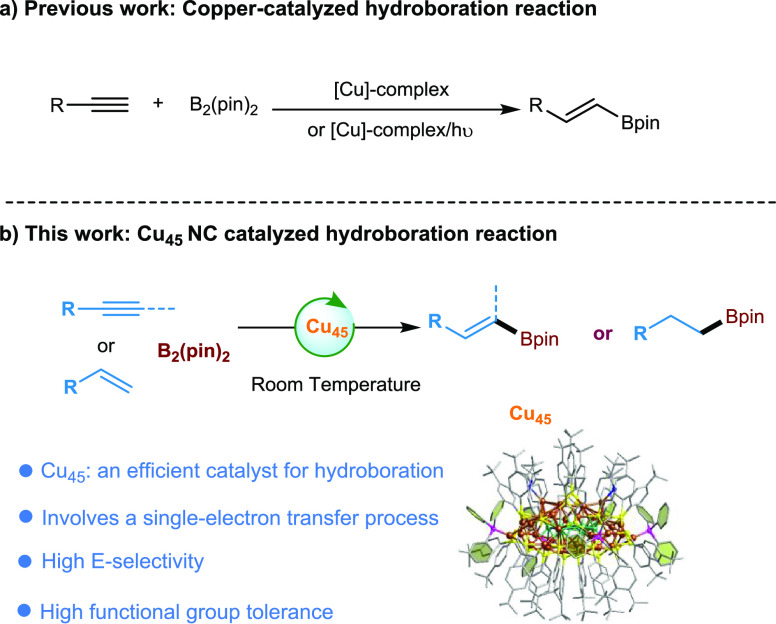
Transition Metal-Catalyzed Hydroboration Reactions

Organoboron compounds are essential building blocks for bond-forming reactions, including (a) Suzuki-Miyaura cross-coupling reactions,^33−35^ (b) Chan-Evans-Lam heteroatom arylation,^36^ (c) allylations,^37^ (d) conjugate addition reactions,^38^ and (e) other reactions.^39^ Furthermore, due to their nontoxicity and bench stability, the synthetic utility of organoboron derivatives has found wide applications in medicinal and pharmaceutical chemistries (e.g., Velcade and Dutogliptin).^40−43^ Apart from the classical methods,^44,45^ an array of powerful metal-catalyzed approaches has been developed for the hydroboration of C–C multiple bonds by using a series of borane reagents.^46−49^ Specifically, the utilization of bench-stable Bis(pinacolato)diboron B_2_Pin_2_ as a reagent in the transition metal-catalyzed hydroboration of C–C multiple bonds (alkene and alkynes) is attractive.^50−52^ Recently, ligand-assisted copper catalysts, copper photoredox catalysis, and Cu–Au alloy NCs have been applied for the hydroboration reactions via classical two-electron^53−62^ or SET processes (Scheme 1a).^63,64^ Herein, we demonstrate that the new **Cu**_**45**_ functions as a potent catalyst for generating boryl radicals from stable B_2_Pin_2_ to enable the borylation of a diverse set of alkynes and alkenes under mild reaction conditions (Scheme 1b). In contrast to previously reported processes,^52−54,63,64^ the newly developed **Cu**_**45**_ reaction strategy features advantages such as (a) new core–shell **Cu**_**45**_ that can act as an efficient and stable catalyst for the hydroboration of C–C multiple bonds under air as well as in wet chemical conditions, (b) not requiring exogenous ligands or photoreactor setups, (c) being an operationally simple method, and (d) functioning with a wide range of alkynes and alkenes for the preparation of organoboron compounds in excellent yield as well selectivity.

## Results and Discussion

### Preparation and Characterization of **Cu_45_**

The synthesis of **Cu**_**45**_ was achieved through a one-pot direct reduction approach, whereby the copper precursor [Cu(CH_3_CN)_4_]BF_4_ was reduced in the presence of the ligand (TBBT) and TPP using the borane *tert*-butylamine complex (^t^BuNH_2_·BH_3_) as a mild reducing agent at room temperature (Figure S1). Initially, the synthesis was carried out using strong reducing agents, such as sodium borohydride (NaBH_4_), which resulted in only insoluble copper complexes. We then attempted a two-step synthesis reaction process, initially forming a mixture of Cu-TBBT and Cu-PPh_3_ complexes followed by reduction, but it was unsuccessful. Our efforts to synthesize the cluster without TPP were also unsuccessful. The critical factors for the successful formation of this cluster are the use of a slow-reducing agent, borane *tert*-butylamine complex, and the inclusion of the secondary ligand, TPP. The presence of the secondary ligand improves the cluster’s stability and facilitates the crystallization process. The synthesis was successfully scaled up, producing ∼0.0748 g of pure crystals via a one-pot process. This is an essential feature as larger-scale synthesis of copper nanocluster crystals under ambient conditions remains a significant challenge due to the highly sensitive nucleation, growth, and crystallization processes involved.^65^

### Structure Analysis of **Cu_45_**

**Figure 1:**
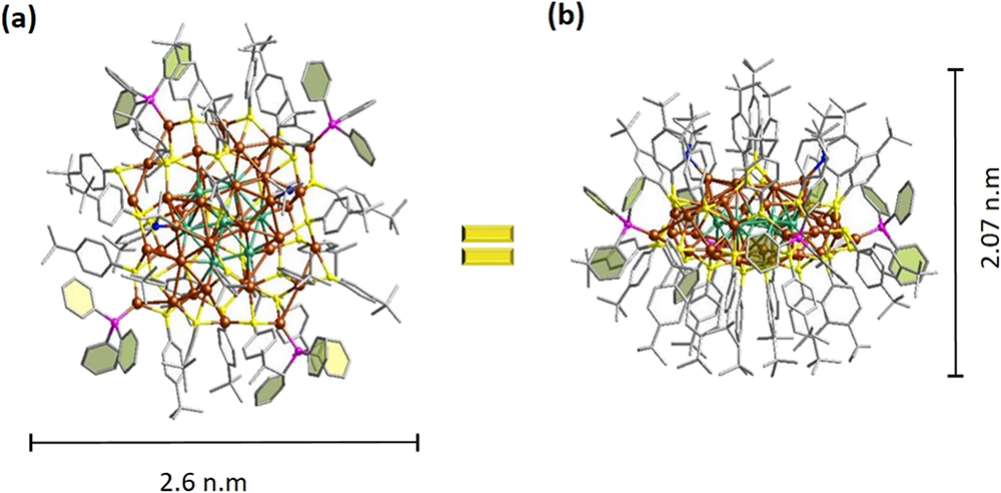
Top (a) and side (b) views of **Cu**_**45**_. Brown (shell)/green (core): Cu; yellow: S; blue: N; gray: C; and pink: P. Carbon tails are shown in wireframe mode, and all H atoms have been omitted for the sake of clarity.

**Figure 2:**
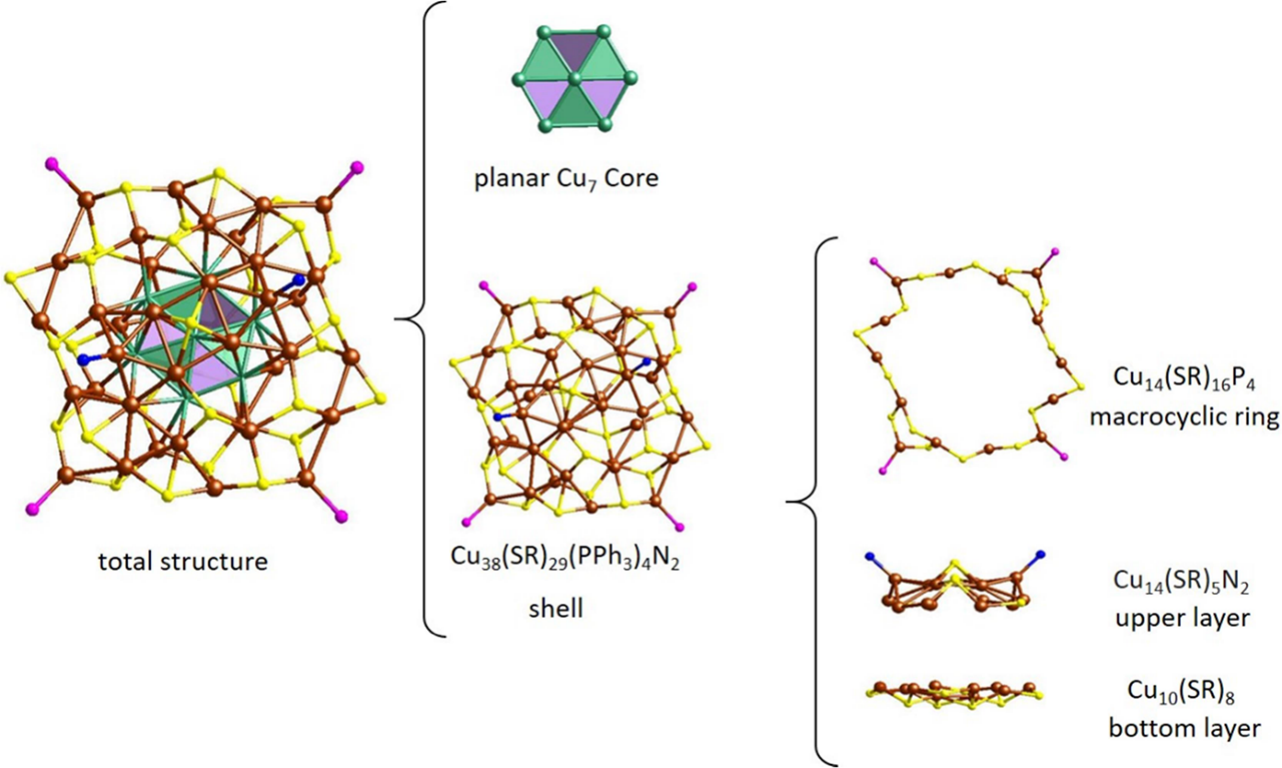
Total structure of **Cu**_**45**_. Core–shell structure in ball-and-stick model showing the upper, bottom, and macrocyclic layers. All C and H atoms are omitted for clarity. Brown (shell)/green (core): Cu; yellow: S; blue: N; and pink: P.

Structural characterization of a high-quality red crystal of **Cu**_**45**_ was carried out by using single-crystal X-ray diffraction (SCXRD). Morphologically, the total structure of **Cu**_**45**_ displays an irregular shape with a distance of 2.6 nm between the two farthest benzene ring carbon atoms and, from the side view, a height of 2.07 nm, as shown in Figure 1. The cluster was crystallized in a triclinic lattice, the *P*-1 space group. The detailed structural analysis reveals that the cluster consists of 45 Cu atoms, 14 hydrides, 29 TBBT, 4 TPP, and two molecules of *tert*-butylamine, ^t^BuNH_2_, as presented in Table S1. This Cu-cluster can be considered one of the top ten largest copper nanoclusters that have been synthesized so far. According to the conventional core–shell anatomy principle, **Cu**_**45**_ was divided into two major components: a hexagonal Cu_7_ core and a cage-like Cu_38_(TBBT)_29_P_4_N_2_ shell, as depicted in Figure 2. The core structure was found to have a completely different structure and symmetry from Ag_7_ and Au_7_ cores in noble metal nanoclusters.^66,67^ Interestingly, the planar and hexagonal shape of the core was first and only observed in Cu_81_.^32^ The core consists of six identical triangles constructed with copper atoms as vertices sharing one copper atom at the center and supported by Cu···Cu cuprophilic interactions at the edges and Cu–S bonds. The Cu–Cu bond length in the Cu_7_ core ranges from 2.54 to 2.87 Å, which is comparable to that in other copper clusters. Furthermore, the Cu···Cu distances between the core and shell range from 2.55 to 2.88 Å, and the average value of 2.73 Å is higher than that within the Cu_7_ core, which is 2.66 Å. The structure features a symmetry of the *C*_2_ axis and one plane passing through the *C*_2_ axis, as shown in (Figure S2).

The architecture of the cage-like shell Cu_38_(TBBT)_29_P_4_N_2_ framework can be viewed as two layers connected by a macrocyclic ring. The upper layer of Cu_14_(TBBT)_5_N_2_ forms a square shape in the center where the square vertexes are sharing one PhS^–^ ligand. In addition, two halves of a honeycomb-shaped configuration of five Cu atoms are connected to two molecules of (^t^BuNH_2_), and the Cu–N bond distance is 1.80 Å. Moreover, there are two Cu atoms observed on the top and, similarly, two Cu atoms at the bottom bridged by Cu–S and Cu–Cu bonds. This layer is considered a metal-rich layer and more likely to exhibit reactivity due to the low ligand coverage. In the bottom layer Cu_10_(TBBT)_8_, thiolates together with copper atoms tend to form the oligomeric “staple-like” motifs. Two tetrameric motifs share two adjacent Cu atoms and two PhS^–^ ligands. These layers’ top and side views are shown in Figure S3. The sulfur atoms resemble the bridges connecting the copper and sulfur atoms in both layers and adopt three different ligation modes: (i) μ_2_, η,^1^ η^1^, (ii) μ_3_, η,^1^ η,^1^ η^1^, and (iii) μ_4_, η,^1^ η,^1^ η,^1^ η^1^ (Figure S4). The Cu···Cu and Cu–S distances are in the range of 2.47–2.82 and 2.23–2.53 Å, respectively. These two layers are connected by Cu_14_(TBBT)_16_P_4_, a large ring-like staple macrocyclic motif. This motif can be separated into two units of a bent monomeric motif and one unit of a cyclic motif. For the bent monomeric motif, the Cu atom is bonded to two PhS^–^ ligands with a S–Cu–S angle of approximately 100°. In the cyclic motif, all the internal angles between Cu and S atoms are different and the average S–Cu–S angle is 130° (Figure S5). The TPP ligands coordinate with the Cu atoms at the corners of the motif, and the Cu–P distances vary from 2.21 to 2.22 Å. Moreover, the capping of the bulky monodentate phosphine ligands solely adopts a linear mode and is terminally bonded to the cyclic motif. Because of their bulky conical configuration and rigid coordination mode, phosphine ligands may direct the extension of the surface framework in specific directions and remain more stable. The presence of the coligand not only enhances the stability of the cluster but also promotes the crystallization process.^65,68,69^ Note that all of our attempts to synthesize the cluster in the absence of TPP were unsuccessful.

**Figure 3:**
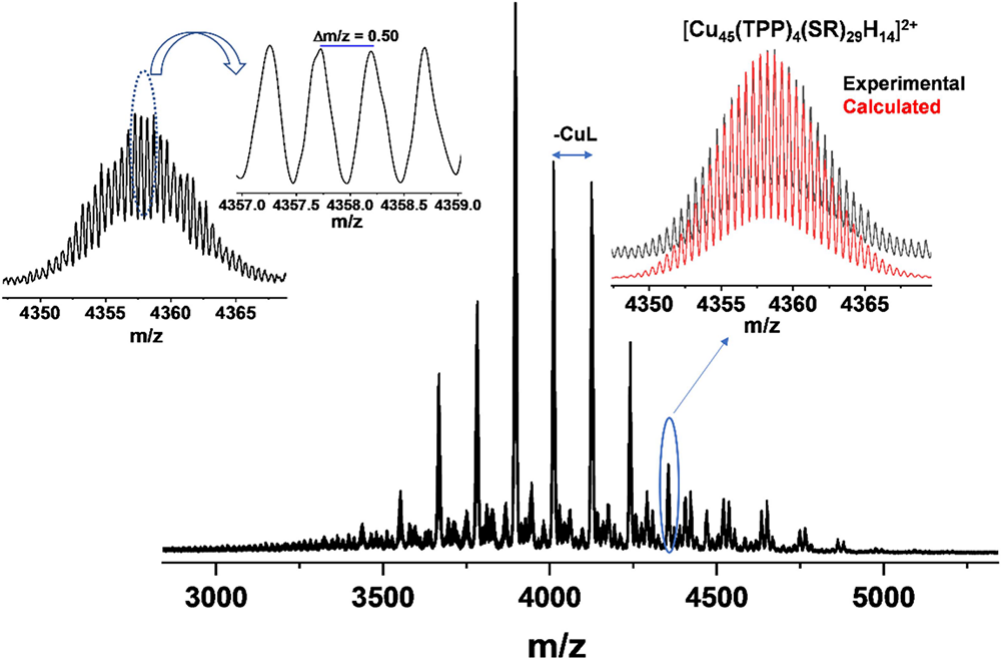
ESI-MS spectrum of **Cu**_**45**_ in positive mode. Molecular ion peak was observed at *m*/*z* 4357. Inset: comparison of the experimental isotopic distributions (black trace) of the peak is in perfect agreement with the simulated isotope pattern (red trace).

The presence of hydrides in a copper cluster is a relatively common phenomenon.^70,71^ However, detecting those hydrides by SCXRD is notoriously difficult. Therefore, to determine the total composition, hydride content, and charge state of **Cu**_**45**_, electrospray ionization mass spectrometry (ESI-MS) was conducted using a few crystals of the cluster that were dissolved in a mixture of a chloroform/acetonitrile solution. The sample preparation and instrument conditions are detailed in the Supporting Information. The full spectrum over the range of *m*/*z* = 3000–5000 was assessed, and the dominant signal was observed only in the positive mode, as presented in Figure 3. The peak centered around *m*/*z* = 4357 is expanded at the inset of Figure 3 and corresponds to [Cu_45_(TBBT)_29_(TPP)_4_H_14_]^2+^, which is derived from losing two molecules of (^t^BuNH_2_) from the parent cluster. This peak matches well with the simulated isotopic pattern of the cluster and thus supports the assigned composition. After expansion, the peak shows a characteristic separation of Δ*m*/*z* = 0.5, confirming the (+2) charged cluster. The other peaks correspond to different cluster species, as the Δ*m*/*z* = 115 between every two successive peaks toward the low-mass region equals the loss of one Cu atom and one ligand.

To further confirm the presence as well as the number of hydrides in the cluster composition, the deuterated analog **Cu**_**45D**_ was synthesized using (^t^BuNH_2_·BD_3_) as a reducing agent following the same procedure used to prepare the protonated cluster. ESI–MS of the deuterated cluster exhibits peaks similar to those of **Cu**_**45**_. The abundant peak assigned in the mass range of *m/z* = 3000–5000 centered at *m*/*z* = 4364 correspond to [Cu_45_ (TBBT)_29_(TPP)_4_D_14_]^2+^. The shift of *m*/*z* in 7 units compared with the peak of [Cu_45_(TBBT)_29_(TPP)_4_H_14_]^2+^ confirms the expected value for 14 hydrides as shown in Figure S6.

X-ray photoelectron spectroscopy (XPS) analysis was performed to further confirm the copper composition and charge state in the cluster. All expected elements (Cu, S, P, N, F, and C) were observed in the survey spectrum of **Cu**_**45**_. The high-resolution XPS spectra of Cu 2p, S 2p, N 1s, F 1s, P 2p, and C 1s are shown in Figure S7. The Cu 2p_1/2_ and Cu 2p_3/2_ peaks at 953.2 and 932.6 eV with a doublet separation of 20.6 eV indicate the presence of Cu(I), and there was no satellite signal at around 943 eV, which illustrates the absence of Cu(II) in the cluster. Furthermore, the Cu LMM auger spectrum of the precursor showed one main peak at 916.3 eV and a shoulder peak at 912.1 eV. Comparison of the Cu LMM spectra of the cluster and copper precursor indicates the existence of a single valence state for Cu(I) in the cluster and that there is no Cu(0) character (Figure S8).

Further, to validate the presence of hydrides in **Cu**_**45**_**,**^2^H NMR (Figure S9) and H_2_ evolution (Figure S10) experiments were conducted. ^2^H NMR of **Cu**_**45D**_ in CHCl_3_ showed the presence of deuterium, as shown in Figure S9, providing clear evidence of hydrides in the structure. Furthermore, the H_2_ evolution experiment showed that H_2_ release began at approximately 135 °C, giving evidence of hydrides in **Cu**_**45**_ (Figure S10). Moreover, density functional theory (DFT) calculations were applied to ascertain the hydride positions in **Cu**_**45**_. The optimization procedure and structures are outlined in the Supporting Information. The coordination environments of 14 hydrides can be classified into five groups in a ratio of 4:2:2:4:2 based on their different structural environments: μ_3_-H_A_, μ_4_-H_B_, μ_4_-H_C_, μ_4_-H_D_, and μ_4_-H_E_ (Figures S11 and S12).

The steady-state ultraviolet–visible (UV–Vis) spectrum of **Cu**_**45**_ in CHCl_3_ demonstrates a smooth curve without any distinctive peaks (Figure S13A). The optical gap was estimated to be 1.9 eV by extrapolating the absorbance to the baseline (Figure S13B). The stability of **Cu**_**45**_ was also probed by monitoring the UV–Vis absorption spectrum for a week in the solution at room temperature, and it was found to be stable in chloroform for at least 48 h and in the solid state for more than three months. Further, we performed ESI-MS of **Cu**_**45**_ after 48 h to confirm its stability which perfectly aligned with the freshly prepared sample, as illustrated in Figure S13C. Further, **Cu**_**45**_ showed decent photo and thermal stability as shown in Figure S14. These notable stability tests of **Cu**_**45**_ emphasize its potential for prolonged and reliable performance in catalysis at room temperature.

We further performed DFT calculations to understand the electronic properties of **Cu**_**45**_. As shown in Figure S15, the calculated energy gap between the highest occupied (HOMO) and lowest unoccupied molecular orbital (LUMO) is 1.92 eV, matching well with the experimental value. The electronic charge density for the HOMO level is delocalized over the core and partially in the protective ligands, while the LUMO level is mainly localized in the core, suggesting that the major optical transition occurs within the cluster core.

### Catalytic Application

To evaluate the catalytic activity of **Cu**_**45**_**,** we conducted initial investigations (Table 1) using 1-(*tert*-butyl)-4-ethynylbenzene (1) and bis(pinacolato)diboron (B_2_Pin_2_) (2) in the presence of 0.025 mol % **Cu**_**45**_ (crystal form) and H_2_O (5.0 equiv) with open-air atmosphere at room temperature (RT). To our delight, hydroborated product **3** was obtained in 90% yield with complete *E*-selectivity (Table 1, Entry 1). Subsequently, we reduced the catalyst loading to 0.01 mol %, which led to a decrease in product yield (Entry 2). Interestingly, a reduced formation of **3** was observed when the reaction was performed under inert conditions (Entry 3). Further reaction optimization includes evaluation of solvents, bases, and water additives (Tables 1 and S2–S4). In addition, we compared **Cu**_**45**_ with **Cu**_**6**1_(19) to establish structure–property correlations since the metal–ligand interface in nanoclusters exhibits greater catalytic activity than the inner core structures.^6^ The structural illustration of **Cu**_**45**_ highlights the potential active sites, including the Cu_14_(TBBT)_16_P_4_ macrocycle, the Cu_10_(TBBT)_8_, and the Cu_14_(TBBT)_5_N_2_ layers. However, control experiments (Figure S17) indicate that the Cu-PPh_3_ complex is more active in the hydroboration reaction (51% yield) than the Cu-TBBT complex (15% yield). This control experiment suggests that the macrocyclic ring (Cu_14_(TBBT)_16_P_4_) of the nanocluster could be the active site for the reaction. It is noteworthy that **Cu**_**61**_ and **Cu**_**45**_ each contain a macrocyclic ring of a qualitatively different composition (i.e., Cu_12_S_6_ in **Cu**_**61**_ and Cu_14_(TBBT)_16_(PPh_3_)_4_ in **Cu**_**45**_), and hence, it is useful to compare their activities. **Cu**_**45**_ exhibited superior catalytic activity (Entry 8) than **Cu**_**61**_, further indicating that phosphine capping in the macrocyclic ring plays a crucial role in their catalytic behavior. In addition, the basic copper source CuCl was investigated as a catalyst under standard conditions (entry 9) instead of **Cu**_**45**_, resulting in a lower yield than **Cu**_**45**_. The nanoscale size of **Cu**_**45**_ provides a larger surface area, enhancing the catalytic reactivity with more active sites. The core–shell arrangement in **Cu**_**45**_ underscores the importance of the metal–ligand interface in catalysis, which promotes more catalytic activity. In contrast, CuCl lacks a core–shell structure, especially the organometallic interface found in **Cu**_**45**_, contributing to the differences in their catalytic behavior. Moreover, various Cu-complexes were investigated as catalysts instead of **Cu**_**45**_ under standard conditions (Figure S17), resulting in lower yields. Due to the exposure to air/wet reaction conditions, the inorganic Cu-complexes may show decreased catalytic activity. In contrast, the atomically precise **Cu**_**45**_ catalyst exhibits a unique cage-like Cu_38_(TBBT)_29_(PPh_3_)_4_N_2_ shell structure, which could prevent aerial oxidation, becoming a stable and efficient catalyst for hydroboration reactions under very mild conditions (for details of stability studies of **Cu**_**45**_ under air/water, see Figure S20). Other control experiments showed that in the absence of the **Cu**_**45**_ catalyst or a base, no reaction or little product formation was observed (Entries 10 and 11).

**Table 1:**
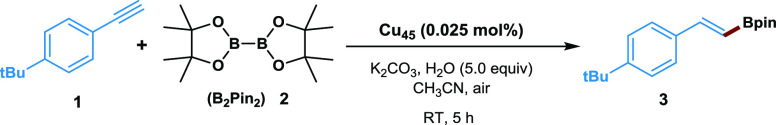
Reaction Optimization^a^

entry	variation from initial conditions	yield of 3 (%)^b^
1	none	92 (90)
2	0.01 mol % **Cu**_**45**_	78
3	in the presence of N_2_	62
4	K_3_PO_4_	66
5	^t^BuOK/Na	15/18
6	CH_3_CN (without H_2_O)	73
7	THF, dioxane, toluene, DMSO, or DMF as solvent	<60
8	Cu_61_ instead of **Cu**_**45**_	58
9	CuCl instead of **Cu**_**45**_	32^c^
10	no catalyst	0
11	no base	14

a Initial conditions: **1** (0.3 mmol), **2** (0.375 mmol), Cu_45_ (0.025 mol %), K_2_CO_3_ 1.2 equiv, H_2_O (5.0 equiv, and acetonitrile (CH_3_CN) (4 mL, 0.075M). Mixture was stirred at RT for 5 h under open air.

b Yield refers to GC yield using 1,3,5-trimethylbenzene (mesitylene) as the internal standard, except for those in brackets which are isolated yields.

c 5 mol % CuCl was used. THF = tetrahydrofuran, DMF = *N*,*N*-dimethylformamide, DMSO = *N*,*N*-dimethyl sulfoxide.

### Substrate Scope

After establishing the optimized reaction conditions (Table 1, Entry 1), we set out to explore the scope of the hydroboration employing various terminal alkynes and internal alkenes (Table 2). In general, a broad range of aryl terminal alkynes, including electron-rich to electron neutral and electron-deficient groups, are amenable to the hydroboration reaction, and the products are obtained with good yields and high stereoselectivity (**3**–**29**, 72 to 92% yields, *E*-selectivity >95%). Remarkably, the **Cu**_**45**_ cluster-catalyzed hydroboration process well tolerates various functional groups including F, Cl, Br, OH, NH_2_, CN, CO_2_Me, CF_3_, and OCF_3_ delivering their corresponding products in good yields (**8**–**20**, 72–88% yields). Aryl alkynes bearing bromides, hydroxyl, and amine functional groups are also tolerated opening up the possibility for subsequent C-heteroatom cross-coupling reactions.^2,72,73^

**Table 2:**
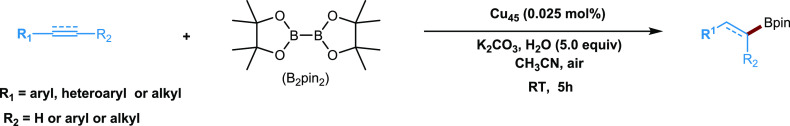

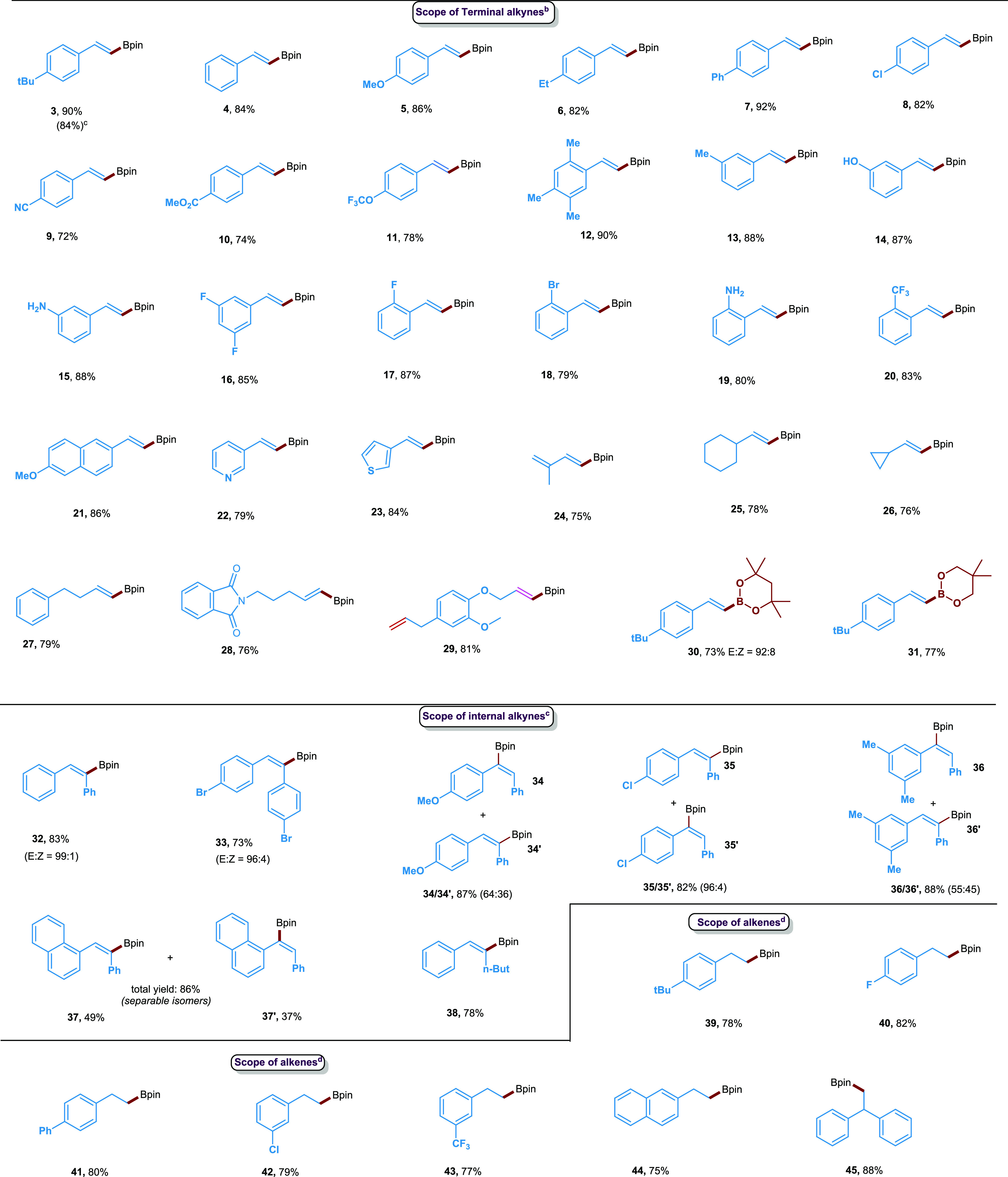
Cu_45_ Catalyzed Hydroboration of Unsaturated Compounds^a^

a Standard conditions.

b *E*-selectivity >95%.

c For optimization, see Table S3. Isolated yield after column chromatography.

d For optimization, see Table S4. Isolated yield after column chromatography.

Heteroaryl alkynes also selectively undergo the hydroboration reaction without influencing either catalyst or borane reagent (**22** and **23**). In addition to aryl alkynes, aliphatic terminal alkynes also exhibit favorable reactivity in this transformation (**24**–**29**, 75–81% yields). The substrate containing both an alkene and an alkyne group (see product **29**) undergoes exclusive hydroboration at the alkyne, leaving the olefin intact. Interestingly, our Cu-cluster methodology can also be extended to other borane reagents (instead of B_2_Pin_2_), providing the corresponding products with high *E*-selectivity (**30** and **31**, 73–77% yields). Internal symmetrical and unsymmetrical alkynes were also examined with a B_2_Pin_2_ coupling partner, and the products were obtained in good yield (**32**–**38**, 73–88% yields). However, unsymmetrically substituted vinyl-hydroborated products (**34**–**37**) were expectedly obtained as a mixture of regioisomers. Besides alkynes, the **Cu**_**45**_ can also be successfully applied to the hydroboration of mono and disubstituted styrene derivatives to provide the borylated alkanes with good yield (**39**–**45**, 75–88% yields). In addition, **Cu**_**45**_ can be recycled (up to 5 cycles) without any significant loss of activity and selectivity (see details in Figure S18). ESI-MS and UV–Vis absorption spectra of the recovered catalyst indicate that **Cu**_**45**_ is intact under the reaction conditions (Figures S18 and S19), highlighting the stability and recyclability of the **Cu**_**45**_ catalyst. Furthermore, owing to the low catalyst loading and the high catalytic activity of the **Cu**_**45**_, it enables gram-scale synthesis of hydroborated product from alkynes with an 84% yield of **3** (Scheme S2).

### Mechanistic Studies

**Scheme 2:**
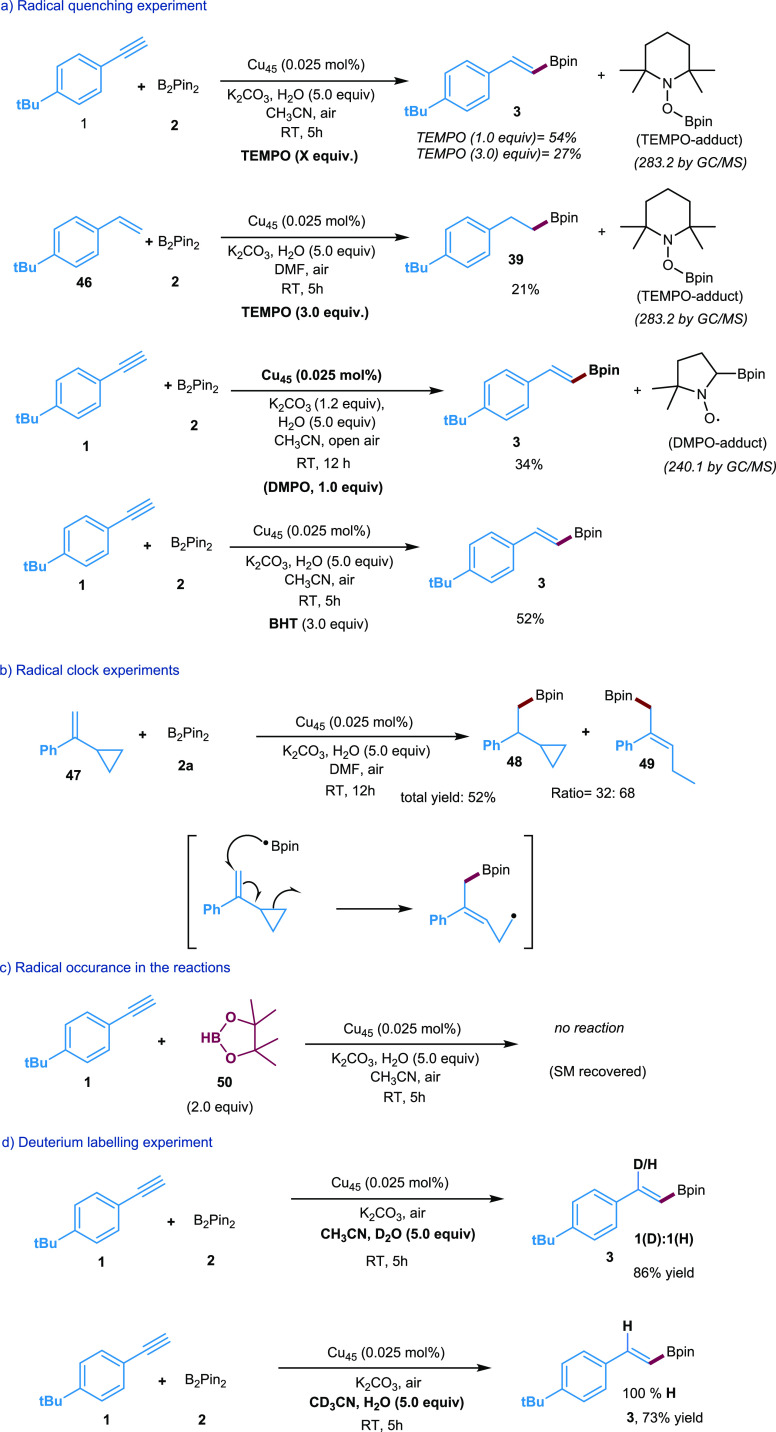
Mechanistic Investigations of the Newly Developed Cu NC Catalyst Hydroboration

To gain a deeper understanding of the reaction mechanism, we conducted control experiments, as illustrated in Scheme 2a–d. Initially, the standard reaction was performed in the presence of well-known radical quenchers such as TEMPO, BHT, and DMPO (Schemes 2a and S4). As a result, the product formation was reduced to 27% (TEMPO), 52% (BHT), and 34% (DMPO). Notably, we have also detected the TEMPO-adducts (MW = 283.2) and DMPO-adducts (MW = 240.1) by GC-MS (see Figures S22–S24). These results are indicative of the radical nature of the **Cu**_**45**_-catalyzed hydroboration reaction.

**Scheme 3:**
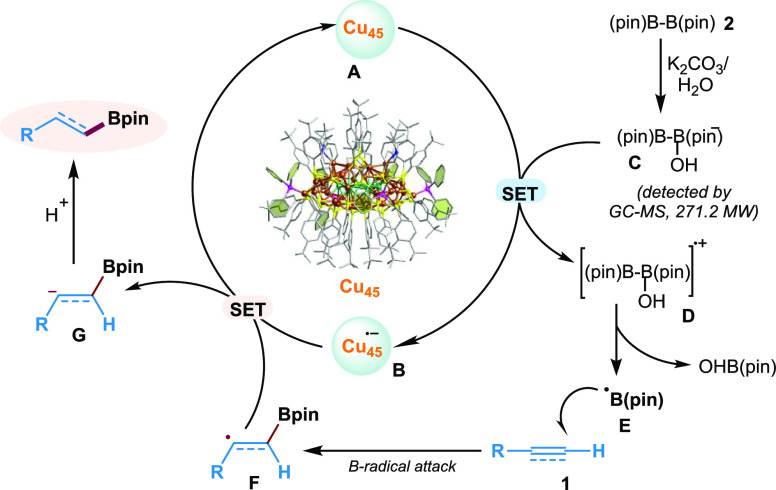
Proposed Reaction Mechanism

Moreover, a radical clock experiment was carried out to determine whether a possible radical process is operative in the current **Cu**_**45**_-catalyzed hydroboration reaction. Using (1-cyclo-propyl-vinyl)benzene **47** as a substrate with B_2_Pin_2_ resulted in product **48** and ring-opening product **49** in a ratio of 32:68 (Scheme 2b, details in Scheme S5), suggesting that the reaction proceeds through a radical pathway. Furthermore, we also investigated the reaction of alkyne **1** with H-Bpin instead of B_2_Pin_2_ under the standard conditions. However, no product was obtained (Scheme 2c), indicating that H-Bpin is not a reactive species or an intermediate. Overall, the above control experiments demonstrate the absence of a two-electron process and suggest the involvement of a radical process. Finally, a deuterium experiment was carried out (Scheme 2d and see details in Scheme S7). The reaction was performed in the presence of D_2_O instead of H_2_O and gave (**3**) in 86% yield with a 1:1 ratio of (H/D) products. In addition, performing the reaction with CD_3_CN and H_2_O yielded the product 100% H incorporation, indicating that the H atom originates from H_2_O. This control study shows that H-abstraction may occur via a hydrogen atom transfer (HAT) process from the solvents (CH_3_CN and H_2_O). However, considering the bond dissociation energy (BDE) of solvents, a HAT process is unlikely.^63^ Instead, the vinyl C-radical **F** (see Scheme 3) has the propensity to form their carbanion intermediate which subsequently undergoes protonation with water to form a stable product (**3**) (for details of proposed mechanism, see Scheme 3).

Based on the above mechanistic control studies and literature reports,^18,74,75^ a plausible mechanism is shown in Scheme 3. The borate *ate* complex **C** ([B_2_Pin_2_]OH^–^) can be formed in situ from B_2_Pin_2_ and Lewis base (OH^–^ from base and H_2_O). Based on the oxidation potential of borate *ate* complex **C** (*E*_1/2_^o^ = ∼+0.37 V vs SCE)^63,64^ and the ground state oxidation potential of **Cu**_**45**_**A** is found to be *E*_1/2_^o^ = ∼+0.755 V vs Ag/AgCl (∼+0.272 V vs Fc/Fc^+^), as shown in Figure S16, a SET from the nanocluster **A** to **C** leads to the formation of the boryl radical **E** and the reduced form of **Cu**_**45**_**B**. The reactive boryl radical **E** has the propensity to attack the C–C multiple bond **1** (e.g., alkenes or alkynes) to generate vinylic-type C-radical intermediate **F**. Subsequently, the radical intermediate **F** can undergo back SET with **B** to generate the **Cu**_**45**_**A** catalyst and a carbanion intermediate **G**, which can undergo protonation to deliver the hydroborated product.

## Conclusions

We have successfully prepared a novel and high-nuclearity copper nanocluster [Cu_45_ (TBBT)_29_(TPP)_4_(C_4_H_11_N)_2_]^2+^ using a one-pot synthesis approach at room temperature. To comprehensively investigate the cluster’s overall structure, chemical composition, and optical properties, an array of characterization techniques was employed, including SCXRD, ESI-MS, NMR, XPS, UV–vis spectroscopy, and DFT. The **Cu**_**45**_ shows high catalytic activity in the hydroboration of alkynes and alkenes using different diboranes under very mild conditions. Notably, our new **Cu**_**45**_ catalyzed methodology – which our mechanistic studies indicate involves the formation of boryl radicals – is compatible with various electronically and sterically diverse alkynes and alkenes. The in-depth findings of our combined experimental and computational studies pave the way for tailoring NC properties to a broad range of bond-forming catalysis reactions.

## Supporting Information Available

Experimental procedures and characterization data (PDF)
