# Supplementary material for: Planar Core and Macrocyclic Shell Stabilized Atomically Precise Copper Nanocluster Catalyst for Efficient Hydroboration of C–C Multiple Bond
Source: J Am Chem Soc. 2024 May 30;146(23):16295–305. doi: 10.1021/jacs.4c05077 (PMC11177319; doi:10.1021/jacs.4c05077)
Supplement: Supplementary file 1 — ja4c05077_si_001.pdf [file ja4c05077_si_001.pdf]

Planar Core and Macrocyclic Shell Stabilized Atomically Precise Copper Nanocluster Catalyst for Efficient Hydroboration of C-C Multiple Bond

Badriah Alamer[†#§], Arunachalam Sagadevan[†§], Mohammad Bodiuzzaman [†], Kathiravan Murugesan[†], Salman Alsharif[†], Ren-Wu Huang[¶], Atanu Ghosh[‡], Naveen Halappa[†], Chunwei Dong[†], Saidkhodzha Nematulloev[†], Jun Yin[⁋], Aleksander Shkurenko[ɸ], Mutalifu Abulikemu[†], Xinglong Dong[ǁ], Yu Han[ǁ], Mohamed Eddaoudi[ɸ], Magnus Rueping[†]*, Osman M. Bakr[†]*.

[†]KAUST Catalysis Center (KCC), Division of Physical Sciences and Engineering, King Abdullah University of Science and Technology (KAUST), Thuwal 23955-6900, Saudi Arabia.

#Department of Chemistry, College of Sciences, Taif University, Taif, P.O. Box11099, Taif 21944, Saudi Arabia.

[¶]Henan Key Laboratory of Crystalline Molecular Functional Materials, Green Catalysis Center, College of Chemistry, Henan International Joint Laboratory of Tumor Theranostic Cluster Materials, Zhengzhou University, Zhengzhou 450001, China.

[⁋]Department of Applied Physics, The Hong Kong Polytechnic University, Hung Hom, Kowloon 999077, Hong Kong, P. R. China.

[ɸ]Division of Physical Sciences and Engineering and Functional Materials Design, Discovery and Development Research Group (FMD3), Advanced Membranes and Porous Materials Center, King Abdullah University of Science and Technology (KAUST), Thuwal 23955-6900, Saudi Arabia.

[ǁ]Advanced Membranes and Porous Materials Center, Physical Sciences and Engineering Division, King Abdullah University of Science and Technology (KAUST), Thuwal 23955-6900, Saudi Arabia.

[‡]Institute for Organic and Bimolecular Chemistry, Georg-August-University Goettingen Tammannstr. 237077 Goettingen, Germany.

*Corresponding author. Email address: Osman.bakr@kaust.edu.sa, magnus.rueping@kaust.edu.sa

Experimental section:

Chemicals

Materials: Cuprous oxide, tetrafluoroboric acid solution (HBF_4, 48 wt. % in water), tertbutyl benzenethiol, triphenylphosphine (TPP), sodium borodeuteride ($NaBD_4$), tert-butylamine complex ($^tBuNH_2{\cdot}BH_3$) and high-performance liquid chromatography (HPLC) grade solvents (acetonitrile, tetrahydrofuran, chloroform, methanol, acetone, and hexane) were purchased from Sigma–Aldrich. All chemicals were used directly without further purification.

Synthesis of the reducing agent ($^tBuNH_2{\cdot}BD_3$)

To a 5 ml of tetrahydrofuran (THF), 200 mg of $NaBD_4$ and 350 mg of $(NH_4)_2SO_4$ as solid was transferred to a 25 mL dry round bottom flask fitted with a water-cooled reflux condenser. After that, 520 µL of tert-butylamine $(CH_3)_3CNH_2$ was added. The heterogeneous reaction mixture was brought to reflux under vigorous stirring for 8 h. Upon completion of the reaction, the product was cooled to room temperature and filtered with around 1.8 g of celite. The product was washed three times by THF and then dried using a rotary evaporator and vacuumed for one day.

Synthesis of Cu_{45}

All procedures for the synthesis of the cluster were performed under ambient conditions. To a solution mixture of 4 mL each of $CHCl_3$ and CH_3CN, 0.1 g of $[Cu(CH_3CN)_4]{\cdot}BF_4$ and 0.05 g of TPP as solid were sequentially added. To this solution, 0.1 g of the reducing agent (*tert*-butylamine borane complex) in 3 ml of MeOH was added successively to reduce the metal-ligand complexes and initiate the formation of the cluster. The color of the solution turns from transparent to dark red immediately. After approximately 45 mins, the reaction was completed and the product in the solution was kept at room temperature in a dark environment. 3 weeks later, good quality crystals of **Cu_{45}** were obtained in ~39% yield (on a copper atom basis).

For the deuterated **Cu_{45D}** synthesis, the above procedure is followed by replacing ($^tBuNH_2{\cdot}BH_3$) with ($^tBuNH_2{\cdot}BD_3$) in the same solvent. The crystals of Cu_{45} and **Cu_{45D}** were collected and washed with acetonitrile and then dried for further characterization.

Characterization Details

UV-vis absorption spectroscopy

UV-vis measurement of the cluster was performed using a Cary 5000 UV-Vis-NIR spectrometer equipped with the integrating sphere.

Optical crystals image

Optical microscopy image of all crystals was performed using an Olympus BX61 Materials Microscope.

Electrospray ionization mass spectrometry (ESI-MS)

Bruker MicroTOF-II mass spectrometer was used for the mass spectrometry measurement. The as obtained cluster crystals were dissolved in $CHCl_3$ and the solution was electrosprayed at 700 µL/h flow rate. The spectrometer was operated in the mass range of m/z 3000–5000 for positive mode.

To obtain 2+ charge state

Flow rate: 700 µL h-1

Capillary voltage: 5 kV

Nebulizer: 0.2 bar

Dry gas flow: 4.0 l/min

Dry temperature: 100 °C

X-ray photoelectron spectroscopy (XPS)

XPS studies were carried out in a Kratos Axis Ultra DLD spectrometer equipped with a monochromatic Al Ka X-ray source (hν = 1486.6 eV) operating at 150 W, a multi-channel plate and delay line detector under a vacuum of ~10–9 mbar. All spectra were recorded using an aperture slot of 300 µm x 700 µm. Survey spectra were collected using a pass energy of 160 eV and a step size of 1 eV. A pass energy of 20 eV and a step size of 0.1 eV were used for the high-resolution spectra. Samples were mounted in floating mode in order to avoid differential charging. Charge neutralization was required for all the samples. Binding energies were referenced to the C 1s peak of (C–

C, C–H) bond, which was set at 284.8 eV. The fresh sample with a small amount of mother liquor was dried totally in the glovebox, and then transferred to the sample holder of the XPS instrument.

Hydrogen evolution

The H_2 evolution experiment of Cu_{45} was performed using a mass spectrometer equipped with a lab-scale fix-bed reactor. The sample was packed into a quartz column (5.8 mm I.D. ×150 mm) with silane-treated glass wool filling the void space. A helium flow (3 cm3 / min) was used to purge the device and material for 30 mins. The temperature of the sample was ramping from 30 to 180 °C at speed of 10 °C/ min and then keep for 30 mins. The effluent from the column was monitored using an online mass spectrometer (GSD 320 Omnistar).

Single-crystals X-ray diffraction (SCXRD) for Cu_{45}

Single Crystal X-ray Diffraction data of Cu_{45} were collected using Bruker X8 PROSPECTOR APEX2 CCD diffractometer using CuKα radiation (λ = 1.54178 Å). Indexing was performed using APEX3 v2018.7-2[1] (Difference Vectors method). Data integration and reduction were performed using SaintPlus 8.38A.[2] Absorption correction was performed by multi-scan method implemented in SADABS-2016/2.[3] Space group was determined using XPREP implemented in APEX3.[1] The structure was solved using Direct Methods (SHELXS-2008)[4] and refined using SHELXL-2018/3[5] (full-matrix least-squares on F^2) contained OLEX2 program[6] package.

The structure reveals significant disorder of coordinated organic ligands; therefore, a large set of restraints and constraints was applied to make both geometry and ADPs of the atoms reasonable. Thus, geometry of the benzene rings was constrained by AFIX 66. In the case of disorder, the lengths of the single C–C bonds [C_{sp3}–C_{sp2}] were restrained by DFIX 1.50. Directionality and planarity of single bonds C–C, S–C and P–C were restrained by SADI and FLAT commands. Bond P–C and S–C lengths in the disordered ligands were restrained by DFIX 1.83 and 1.78, respectively. ADPs of atoms of the disordered moieties were restrained by SIMU 0.015. All the hydrogen atoms were located from the difference Fourier maps first and then placed at the calculated positions

and refined using a riding model with U_{iso}(H) = 1.2U_{eq}(C_{sp2}) or 1.5U_{eq}(C_{sp3}). The uninterpretable electron density was found in the voids in the structure and masked from the refinement using the SQUEEZE routine implemented in the program PLATON: the structure factors were augmented via reverse Fourier transform methods.[7] The resultant FAB file containing the structure factor contribution from the electron content of the void space was used in together with the original HKL file in the further refinement. The FAB file with details of the SQUEEZE results is appended to this CIF file. The SQUEEZE procedure corrected for 840 electrons within the solvent accessible voids (totally 1893 Å^3). The final crystallographically estimated formula of the material is $[Cu_{45}H_{14}(C_{10}H_{14}S)_{29}(TPP)_4(C_4H_{11}N)_2](BF_4)_2 \cdot 24(C_3H_6O)$.

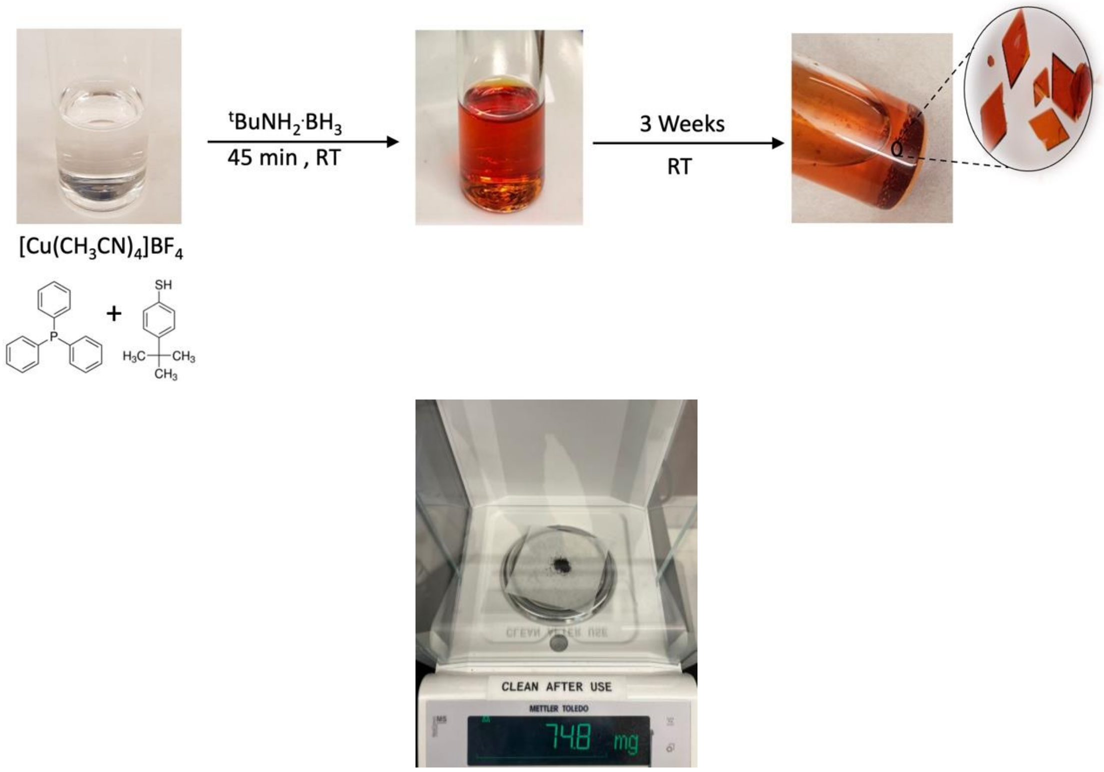

Figure S1. Synthesis of **Cu_{45}** cluster and photograph of **Cu_{45}** crystals (0.0748 g).

Table S1. Crystal data and structure refinement for **Cu_{45}**

Empirical formula	$C_{442}H_{617}B_8Cu_{45}F_8N_2O_{24}P_4S_{29}$
CCDC deposition number	2297555
Formula weight	10493.74
Crystal system, space group	Triclinic, *P*-1
Unit cell dimensions	a = 24.0437(5) Å, b = 26.1542(5) Å, c = 39.9545(8) Å, α = 80.298(1)°, β = 86.562(1)°, γ = 62.985(1)°
Volume	22059.6(8) $Å^3$
Z, calculated density	2, 1.580 Mg m^{-3}
F(000)	10832
Temperature (K)	123.0 (1)
Radiation type, λ	Cu $K\alpha$, 1.54178 Å
Absorption coefficient	4.15 mm^{-1}
Absorption correction	Multi-scan
Max and min transmission	0.149 and 0.048
Crystal size	0.012 × 0.12 × 0.25 mm
Shape, colour	Rhombus plate, red
θ range for data collection	3.0 – 58.9°
Limiting indices	$-26 \leq h \leq 26$, $-29 \leq k \leq 29$, $-44 \leq l \leq 44$
Reflection collected / unique / observed with $I > 2\sigma(I)$	209985 / 62839 (R_{int} = 0.058) / 44314
Completeness to θ_{full} = 58.9°	99.1 %
Refinement method	Full-matrix least-squares on F^2
Data / restraints / parameters	62839 / 5794 / 5164
Final *R* indices [$I > 2\sigma(I)$]	R_1 = 0.0566, wR_2 = 0.1525
Final *R* indices (all data)	R_1 = 0.0859, wR_2 = 0.1744
Weighting *scheme*	$[\sigma^2(F_o^2) + (0.0941P)^2 + 52.0504P]^{-1*}$
Goodness-of-fit	1.03
Largest diff. peak and hole	1.38 and -0.67 e $Å^{-3}$

$^*P = (F_o^2 + 2F_c^2)/3$

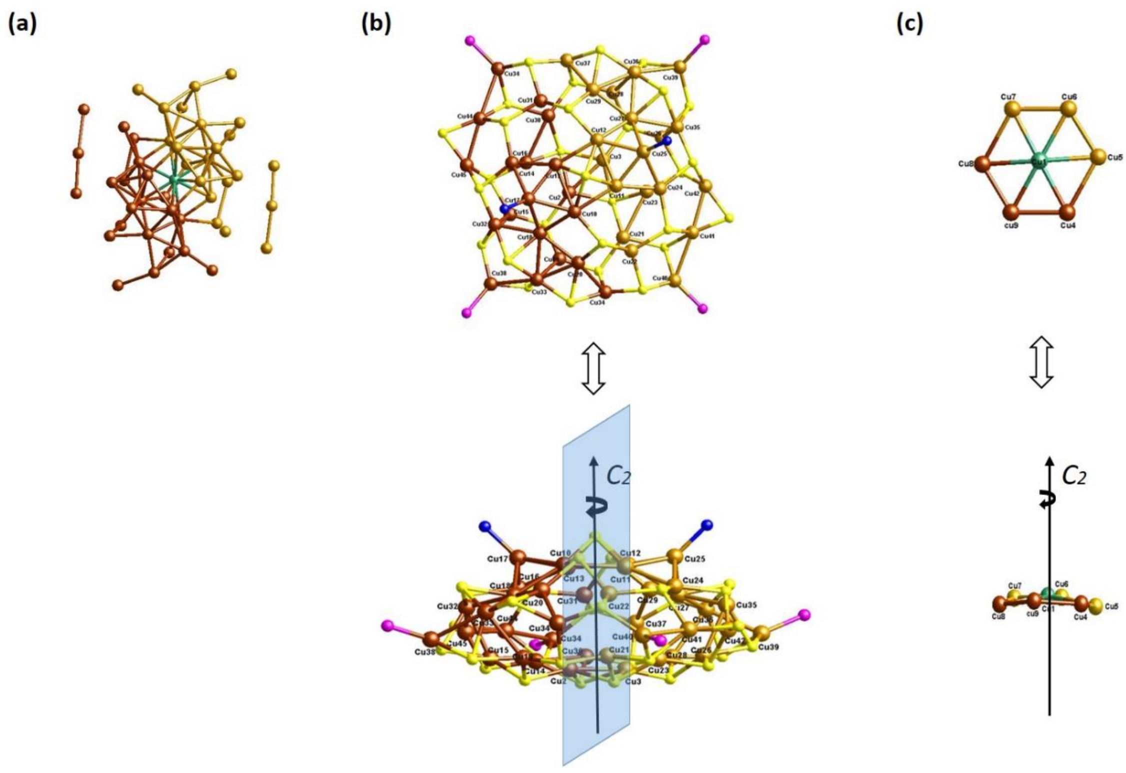

Figure S2. (a) The Cu…Cu interaction in the **Cu_{45}**. (b) Top and side view of the symmetrical shell (c) Top and side views of the symmetrical core.

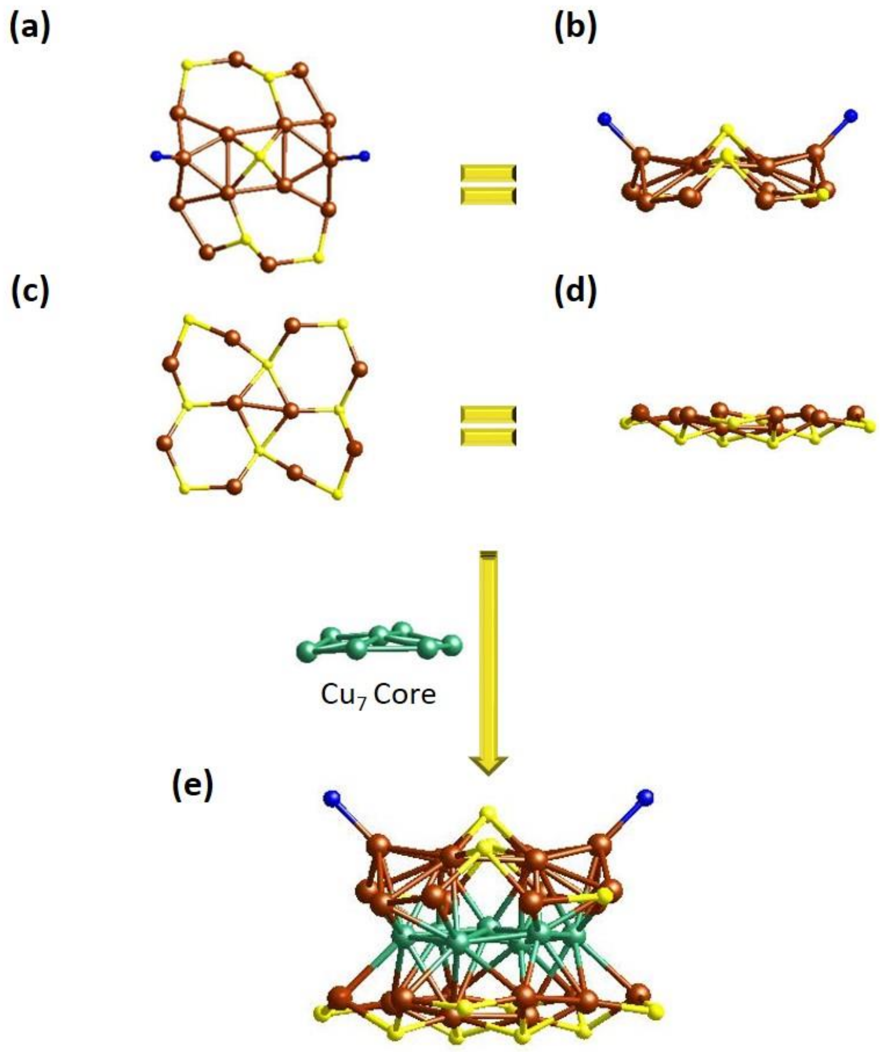

Figure S3. Ball-and-stick models of the two layers showing the top (a) and side (b) views of the upper layer; (c and d) the top and side views of the bottom layer; respectively. (e) Both layers are connected via Cu_7 core. Brown (layers)/green (core): Cu; yellow: S; blue: N.

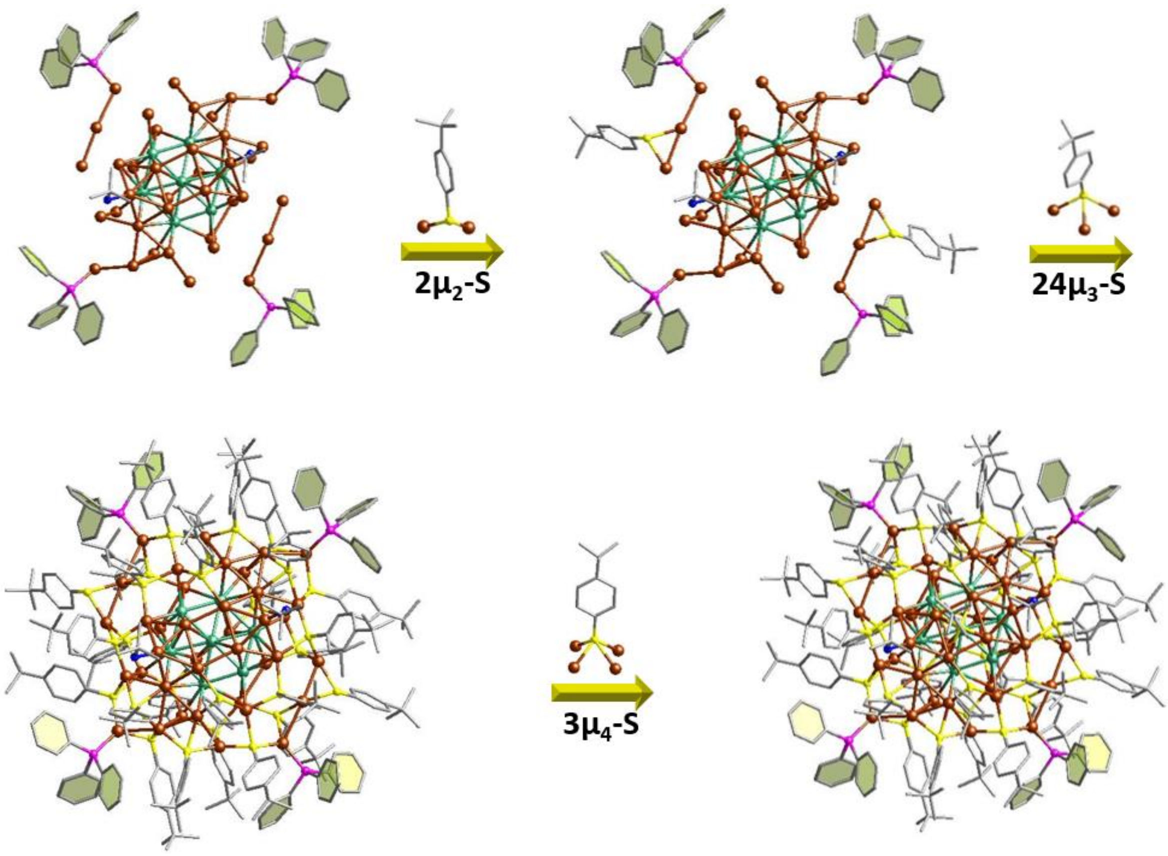

Figure S4. There are three different types of S−Cu coordination modes observed on the surface of **Cu_{45}**. Carbons are drawn in the stick model. Brown (shell)/green (core): Cu; yellow: S; blue: N; gray: C; pink: P.

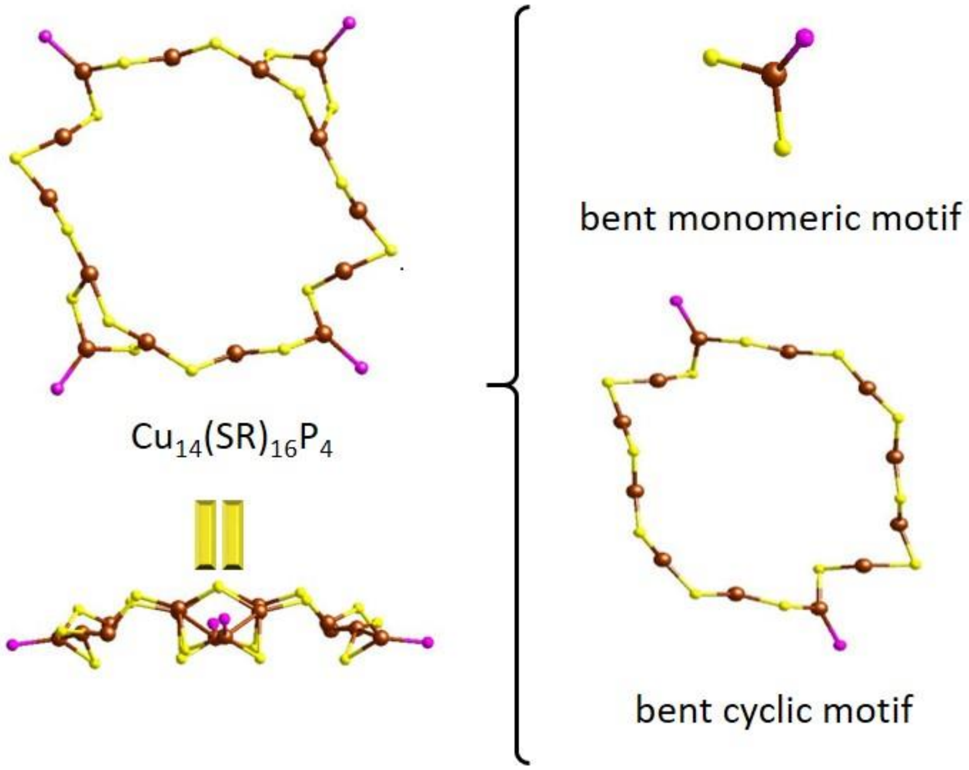

Figure S5. Ball-and-stick models of the shell (2). There are two different types of staple-like motifs on the surface of the Cu_{45} nanocluster. Brown: Cu; yellow: S; pink: P.

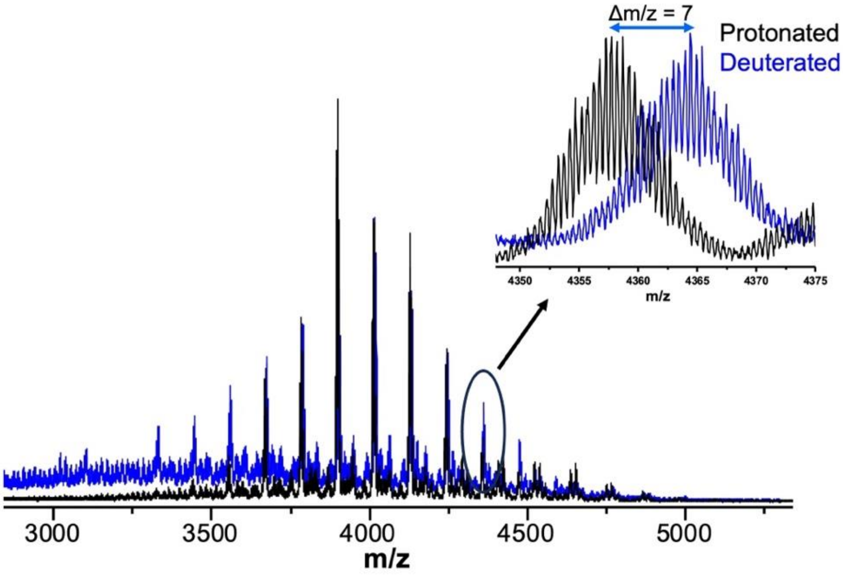

Figure S6. ESI−MS mass spectrum of $\mathbf{Cu_{45D}}$ in positive ion mode. The zoomed-in mass spectrum of $\mathbf{Cu_{45}}$ (black trace) and $\mathbf{Cu_{45D}}$ (blue trace) is in the range of 4000 – 4500 m/z. Insets: Comparison of the most dominant peak of $\mathbf{Cu_{45}}$ and $\mathbf{Cu_{45D}}$.

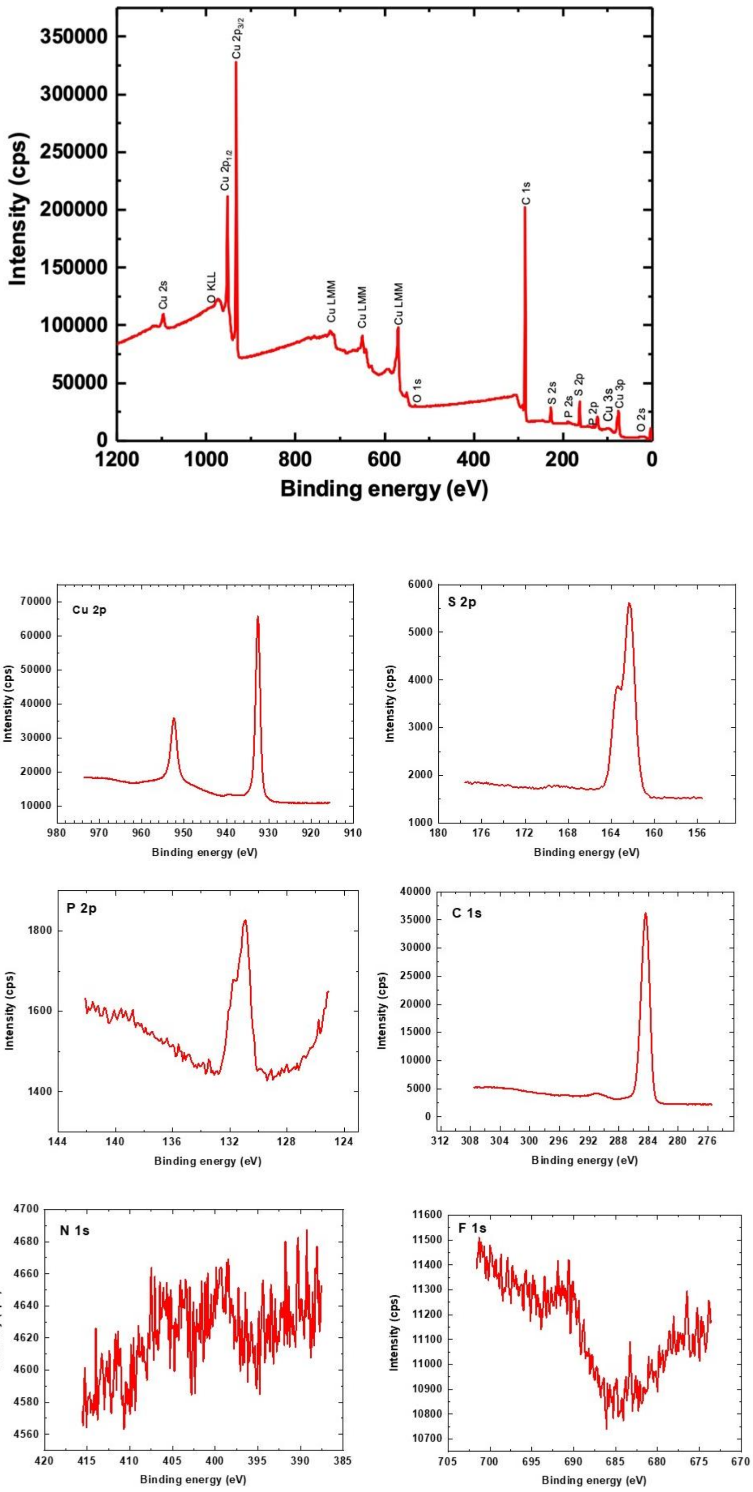

Figure S7. The full XPS spectrum of **Cu$_{45}$** and high-resolution XPS spectra of Cu2p, S 2p, C1s, N 1s, P 2p, and F 1s.

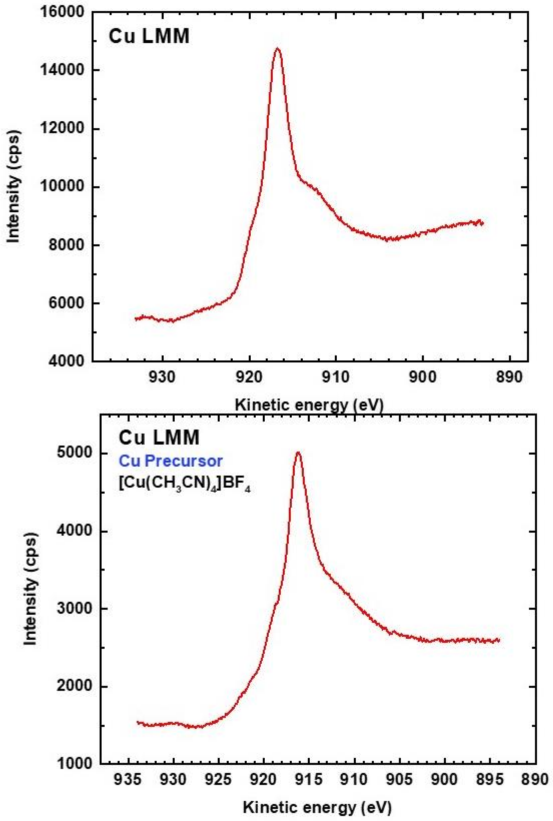

Figure S8. Cu LMM spectra of **Cu_{45}** and [Cu $(CH_3CN)_4$]BF_4.

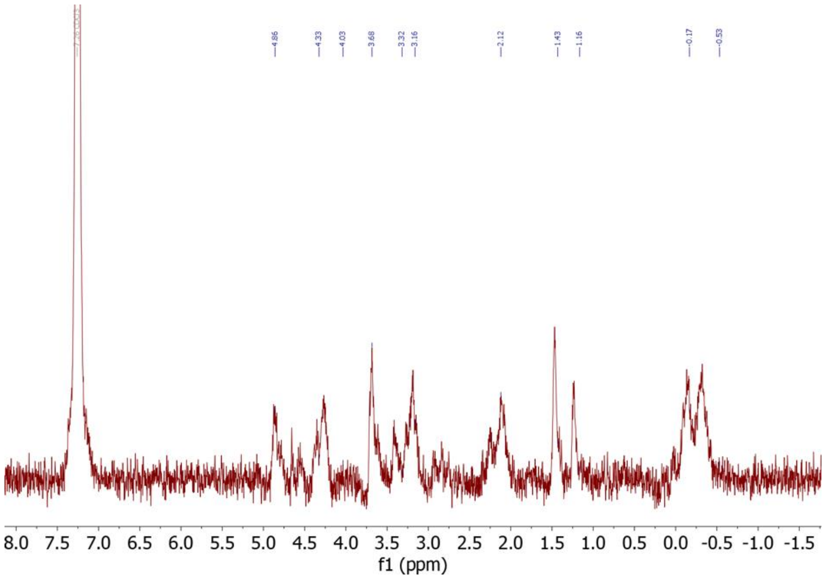

Figure S9. ^2H NMR of **Cu_{45D}.** ($CHCl_3$, 950 MHz) spectrum of **Cu_{45D}**. The spectrum was calibrated by the $CDCl_3$ peak (7.26 ppm). The spectrum was collected at room temperature (ca. 298 K).

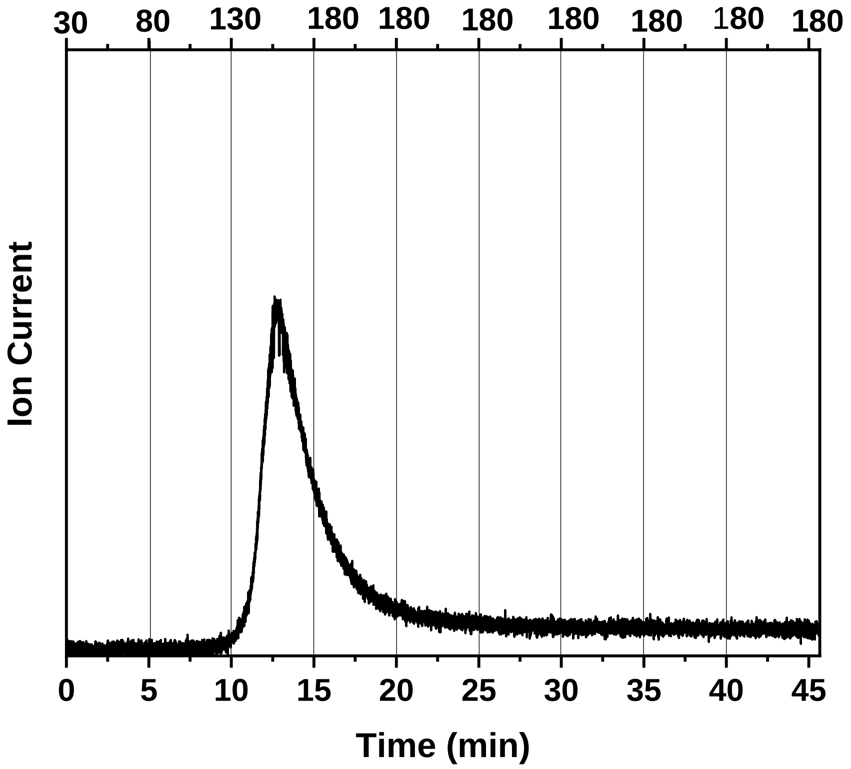

Figure S10. Hydrogen evolution experiments for Cu_{45}. Collected MS signal of *m/z* = 2 under a heating rate of 10 °C/ min from 30 to 180 °C. The *m/z* = 2 channel corresponds to H_2. Owing to the presence of numerous unassignable solvent molecules (chloroform, acetone, and hexane) in the crystals, we are not able to determine the precise molar weight of the cluster crystals, which lead to a failure to quantify H_2 based on this experimental result.

Liquid state nuclear magnetic resonance spectroscopy

^{2}H NMR was recorded on a Bruker AVANCE III-950 MHz spectrometer in $CHCl_3$. All spectra were processed by Topspin 2.1 and calibrated by the $CDCl_3$ peak (7.23 ppm) for ^{2}H NMR. For sample preparation, 3 mg fresh crystals were dissolved in 500 μL solvent.

Density functional theory (DFT) calculations

The geometry of the nanocluster **Cu_{45}** with 14 hydrides was optimized by using the projector-augmented wave (PAW) method as implemented in the Vienna Ab initio simulation package (VASP). The generalized gradient approximation (GGA) with Perdew-Burke-Ernzerhof (PBE) exchange-correlation functional was used. The Γ-point sampling was used, and the plane-wave basis set cut off the wave functions was set at 450 eV. Starting from the experimental structure of the cluster, the initial coordinates of the hydrides were set according to the coordination behavior of hydrides observed in previously reported hydride-containing Cu clusters. Only the hydrides and Cu atoms were allowed to relax during the optimization in order to retain the crystal symmetry of the nanocluster. Based on the optimized geometry of **Cu_{45}** in the ground state, the orbital energies and corresponding electronic change densities of Cu_{45} were calculated with the hybrid functional B3LYP using the Gaussian 09 code (version D.01). The LANL2DZ basis set was used for Cu atoms and the 6-31G(d) basis set was used to describe H, C, N, S, and P atoms.

Hydride Determination

Due to the small size and X-ray transparency of hydrides, accurately determining their precise locations during SCXRD measurements presents a challenge. Thus, the density functional theory (DFT) calculations were applied to ascertain the hydride positions in **Cu_{45}**. The strategy used here was to allow the hydrides to relax initially, and subsequently Cu together with H were fully relaxed. The hydrides were first optimized in the experimental crystallographic structure of **Cu_{45}**. The final optimized structure possesses the same symmetry, average bond length, and structure packing as shown in **(Figure S11)**. Note, the surface ligands and the Cu atoms that are not directly bound to

hydride are omitted in (b&c)). The 14 hydrides can be classified into five groups in a ratio of 4:2:2: 4:2 based on their different structural environments: μ_3−H_A, μ_4−H_B, μ_4−H_C, μ_4−H_D, and μ_4−H_E. The hydrides are distributed in the space between the two layers (upper and bottom layers) and the core (middle layer). Among the 14 hydrides, two in red color are co-protecting the upper layer and forming like a triangular face capping with three-fold coordination μ_3−H mode with an average Cu−H distance of 1.691 Å. The remaining hydrides take the μ_4−H coordination mode and are nestled in distorted tetrahedral cavities formed by Cu atoms. Two hydrides in pink connect one Cu atom of the upper layer with three Cu atoms from the core via μ_4 mode with an average Cu−H distance of 1.833 Å. The space between the upper, middle, and bottom layers filled with two hydrides in gray color bound to four Cu atoms from each layer with an average Cu−H distance of 1.785 Å in the μ_4−H mode. Moreover, there are four hydrides in black color that link the three layers together with an average distance of 1.767 Å through μ_4−H mode. The last four hydrides in blue link the middle and bottom layers together with an average distance of 1.737 Å forming four coordination modes. These results indicate the critical rule of hydride arrangements in bridging the interior copper atoms and exterior copper atoms and, hence, the construction and stabilization of the cluster configuration.

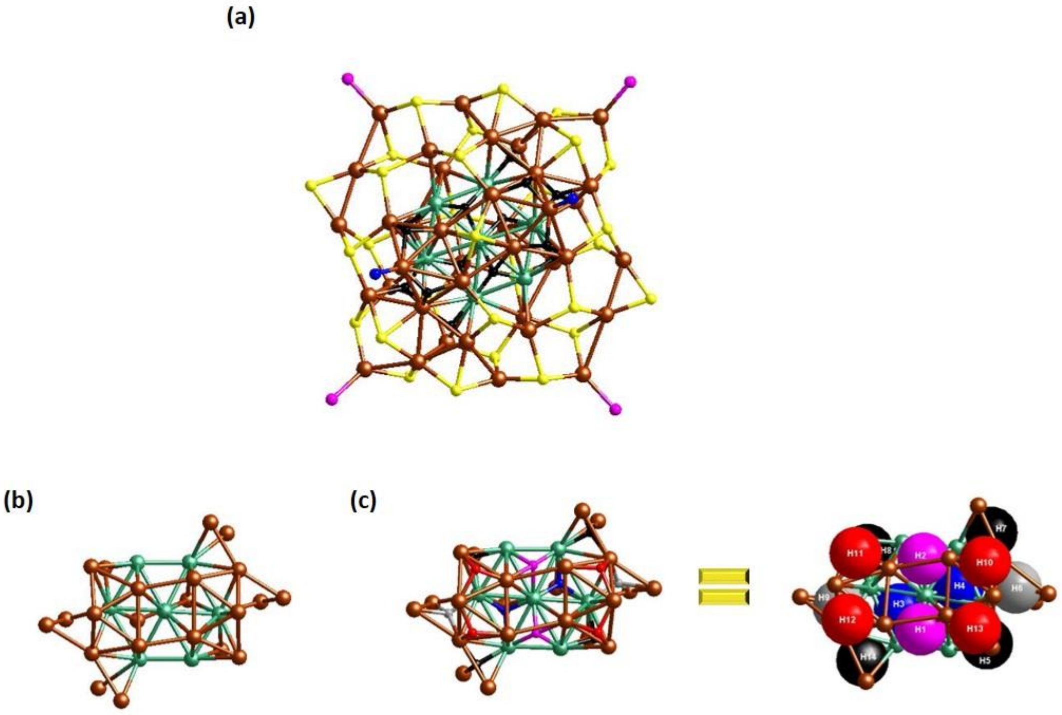

Figure S11. (a) Optimized geometry of **Cu_{45}** obtained from DFT calculations (the aromatic rings of Ph−S are omitted). (b) The initial cluster model (c) The optimized structure (the surface ligands and the Cu atoms that are not directly bound to hydride are omitted). The hydrides within different structural environments are represented using different colors: two hydrides (in pink) between the upper and middle layers, four hydrides (in red) in the upper layer, two hydrides (in gray) between the up, bottom, middle and outer shell layers, and two hydrides (in blue) between the middle and bottom layers, and four hydrides (in black) between the upper, middle and bottom layers. Brown (shell)/green (core): Cu; black/red/blue/pink/gray: H.

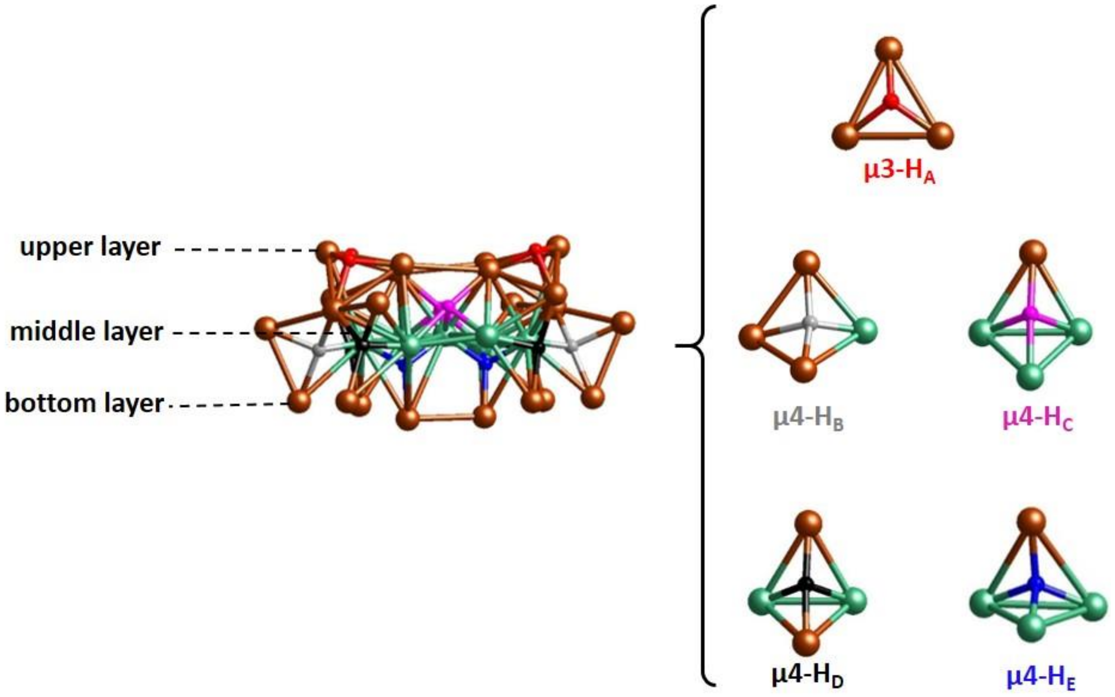

Figure S12. The optimized geometry of **Cu_{45}** was obtained from DFT calculations. The C and protons were omitted for clarity. The coordination environments of the hydrides in **Cu_{45}**. The hydrides connect the Cu atoms to form a Cu−H network. Ten hydrides adopt μ_4 mode and four hydrides adopt μ_3 mode.

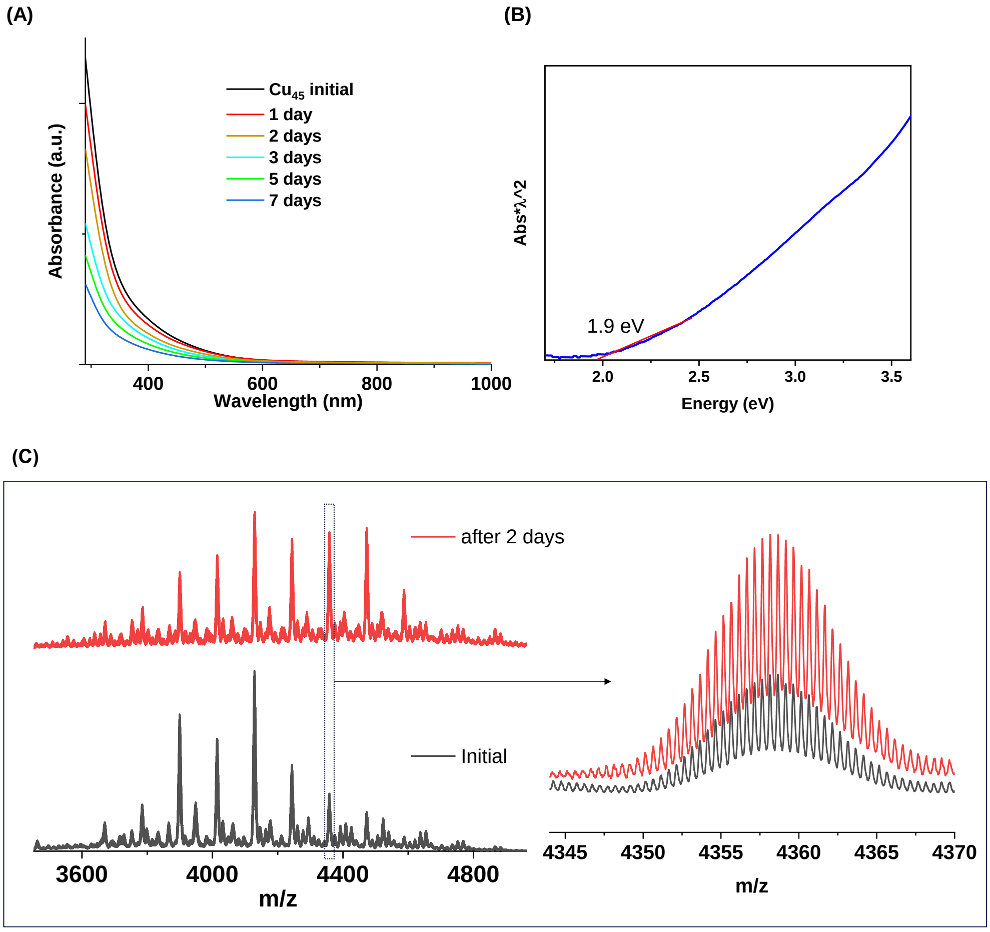

Figure S13. (A) Time-dependent UV-vis spectra of **Cu_{45}** crystals dissolved in $CHCl_3$. Inset: photograph of the $CHCl_3$ solution of **Cu_{45}**. (B) The spectrum is plotted on the photon energy scale (the y-axis is transformed from the wavelength scale spectrum by (Abs* λ^2). (C) Comparison of ESI MS of Cu_{45} before and after 2 days in ambient condition.

Stability test

Photostability was performed under a 10-Watt white LED light. The results indicated no significant changes in the spectra for up to 6 hours, except for a slight decrease in absorbance (Figure S14A). Concerning thermal stability, the cluster was heated at 50°C in dichlorobenzene (Figure S14B). Significant spectral changes were observed between 6 and 9 hours and a yellow precipitate was observed. We also evaluated thermal stability in the crystalline state using thermogravimetric analysis. This analysis demonstrated that the cluster is stable up to 125°C, beyond which it begins to lose ligands, as shown in Figure S14C. Seemingly, thermal stability of Cu_{45} at high temperature is limited in solution phase.

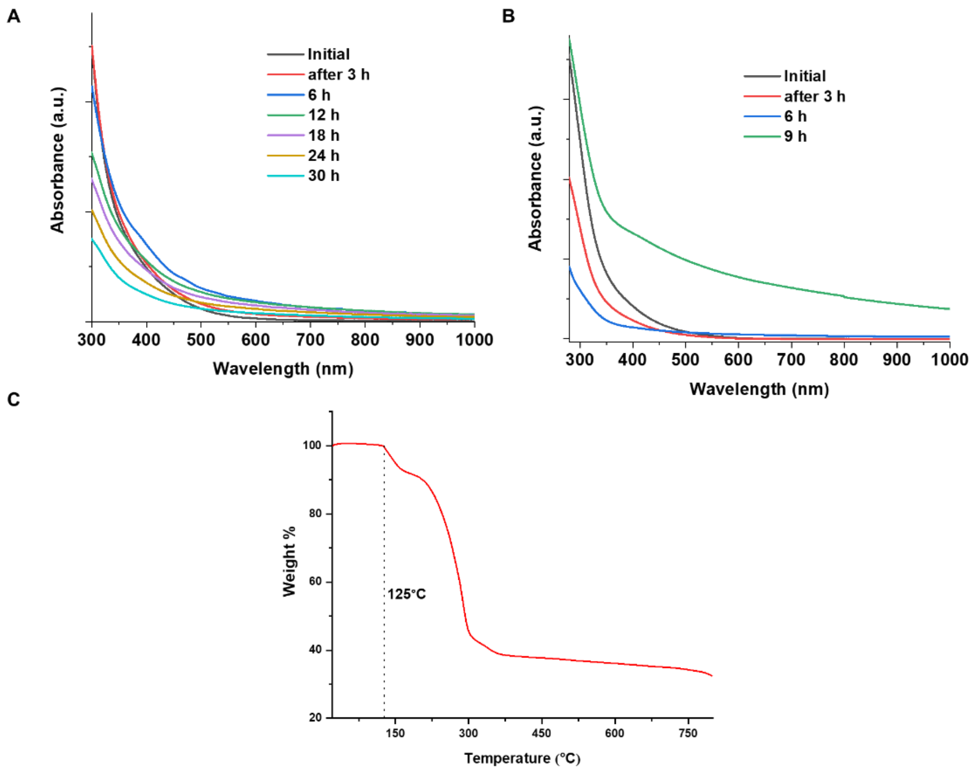

Figure S14. (A) Stability in presence of light. (B) Thermal stability at 50° C in solution of dichlorobenzene. (C) Thermogravimetry of Cu_{45} in crystalline state.

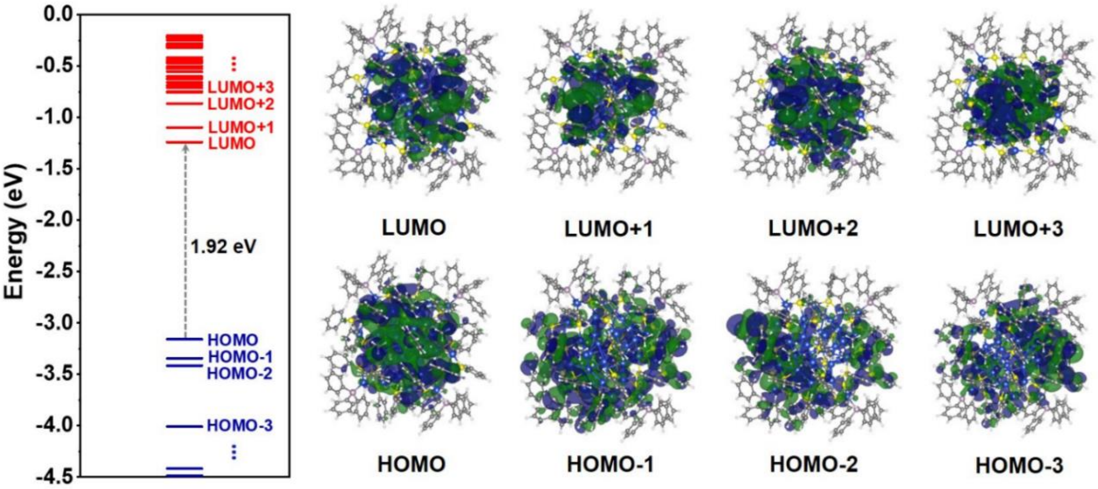

Figure S15. Energy levels and corresponding electronic charge densities for HOMO–3 to LUMO+3 of the optimized **Cu_{45}** cluster, as calculated at B3LYP/6-31G (d) +LANL2DZ level of theory.

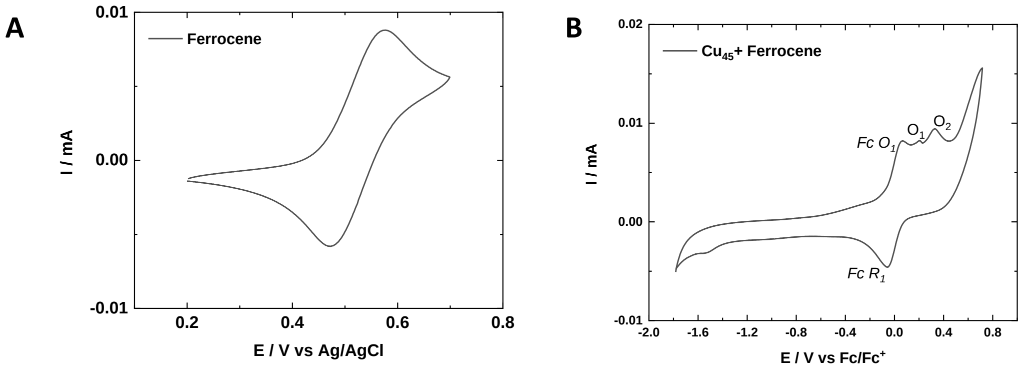

Figure S16. Cyclic voltammograms of (A) Ferrocene (FC) and (B) Cu_{45} with ferrocene were measured in dichloromethane containing tetrabutylammonium perchlorate (TBAP) at a scan rate of 0.05 V/s. Platinum wire, Ag/AgCl and gold disc electrodes were used as counter electrode, reference electrode, and working electrode, respectively. CV data were collected in an Ar atmosphere using three-electrode system measured in Gamry potentiostat. O_1 to O_2 represents the oxidation potentials and R1 represents the reduction potentials.

Cu_{45}-Catalyzed Hydroboration

Experimental section: All reactions were conducted under an open-air atmosphere in glassware. All chemical reactions were conducted at room temperature, (~30 °C) expect those mentioned in bracket. Solvents of anhydrous quality were purchased and used as received. Starting materials were commercially available (Sigma–Aldrich or Alfa–Aesar) and used as received. For brevity, all the reported yields refer to the isolated compounds. Column chromatography was conducted using an Interchim PuriFlash®215 or Biotage Selekt automatic purification system. Organic solutions were concentrated under reduced pressure on a Büchi rotary evaporator.

All isolated compounds were characterized by ^{1}H NMR, ^{13}C NMR spectroscopy, and gas chromatography-mass spectroscopy (GC-MS). ^{1}H and ^{13}C NMR spectra were recorded at 298 K on 400 or 500-MHz Bruker NMR spectrometers. Signal positions were recorded in δ ppm and measured from the center of the signal, excluding multiples, which are given as a range. Splitting patterns are reported using the abbreviations s, br. s, d, t, q, quin, sept, and m (or combinations thereof) denoting singlet, broad singlet, doublet, triplet, quartet, quintet, septet, and multiplet respectively. The abbreviation "app" refers to "apparent". All ^{1}H NMR and ^{13}C chemical shifts were referenced to the residual solvent peak of $CDCl_3$ (^{1}H referenced to 7.26 ppm and ^{13}C referenced to 77.16 ppm). All coupling constants, J, are quoted in Hz and reported to the nearest 0.1 Hz as observed in the spectra. Thin-layer chromatography (TLC) was performed on commercially available pre-coated Merck aluminum TLC sheets (silica gel 60 F254). Gradient elution using 100% hexane and EtOAc/hexane was performed using the TLC. Visualisation of spots on TLC plates was achieved under UV light at 254 nm. All GC analyses (calibrated GC yields) were performed on an Agilent 7890A GC system with an FID detector using a J & W HP–5ms column (10 m, 0.1 mm I.D.) and mesitylene was used as an internal standard. All GCMS analyses were performed using an Agilent 7890A GC system with a J & W DB–5ms column (30 m, 0.1 mm I.D.) connected to a 5975C inert XL EI/CI MSD (with triple-axis detector).

Details of Catalytic Procedure

Scheme S1

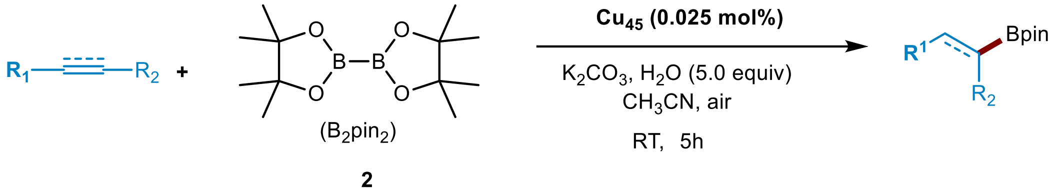

R_1 = aryl, heteroaryl or alkyl

R_2 = H or aryl or alkyl

A 10 mL vial was charged with a magnetic stirrer bar, **Cu_{45}**, (0.000075 mmol, 0.025 mol%), K_2CO_3 (1.2 equiv, 0.36 mmol), B_2pin_2 **2** (0.45 mmol, 1.5 equiv) and alkyne or olefin (0.3 mmol, 1.0 equiv), H_2O (5.0 equiv), and solvent acetonitrile (4.0 mL) was added to the vial. The reaction mixture was then stirred at room temperature (~30 °C) under an open-air atmosphere for 5 hrs. In the case of olefin and internal alkynes, the reaction mixture was heated to (~45 °C) under an open-air atmosphere for 20 hrs. The mixture was filtered through celite and silica gel pads and washed with ethyl acetate. The filtrate was concentrated, and the residue was purified by column chromatography on silica gel to collect the desired products.

Preparative scale for Hydroboration of alkyne by using catalytic Cu_{45}NC.

Scheme S2

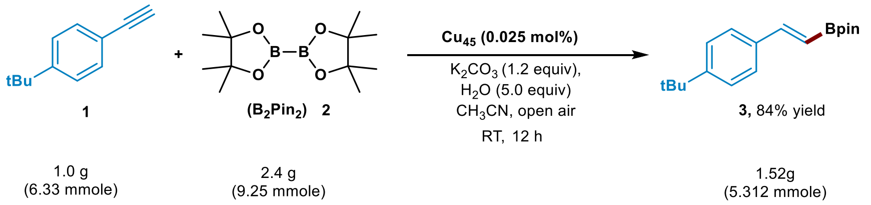

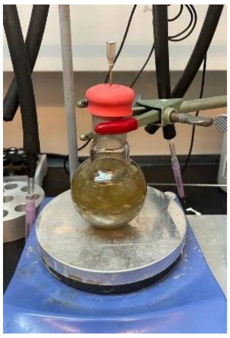

A 100 mL vial was charged with a magnetic stirrer bar, **Cu_{45}**, (0.00177 mmol, 0.025 mol%, 8.0mg), K_2CO_3 (1.2 equiv, 7.57 mmol), B_2pin_2 **2** (9.25 mmol, 1.5 equiv, 2.4g) and 1-(tert-butyl)-4-ethynylbenzene **1** (6.33 mmol, 1.0 equiv, 1.0g), H_2O (5.0 equiv), and solvent acetonitrile (75.0 mL) was added to the vial. The reaction mixture was then stirred at room temperature (~30 °C) under an open-air atmosphere for 12 hrs. The

mixture was filtered through celite and silica gel pads and washed with ethyl acetate. The filtrate was concentrated, and the residue was purified by column chromatography on silica gel to collect the corresponding hydroborated product **3** in 84 % yield (1.52 g).

Table S2. Reaction Optimization for Terminal Alkynes[a]

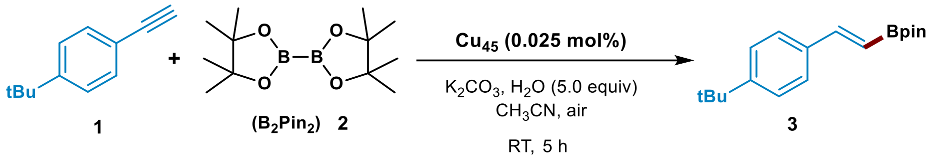

Entry	Variation from initial conditions	Yield of 3a (%)[b]
1	None	92 (90)
2	0.01 mol% **Cu_{45}**	78
3	in the presence of N_2	62
4	K_3PO_4	66
5	$^tBuOK/Na$	15/18
6	CH_3CN (without H_2O)	73
7	THF, dioxane, toluene, DMSO, or DMF as solvent	<60
8	Cu_{61} instead of **Cu_{45}**	58
9	CuCl or $CuCl_2$ instead of **Cu_{45}**	<36[c]
10	no catalyst	0
11	no base	14

[a]Initial conditions: **1** (0.3 mmol), **2** (0.375 mmol), Cu_{45} (0.025 mol%), K_2CO_3 1.2 eq., H_2O (5. 0 eq., and acetonitrile (CH_3CN) (4 mL, 0.075M). The mixture was stirred at RT for 5 h under open air. [b] Yield refers to GC yield using 1,3,5-trimethylbenzene (mesitylene) as the internal standard, except for those in brackets which are isolated yields. [c]5 mol-% CuCl or $CuCl_2$ was used. THF=tetrahydrofuran, DMF = N,N-dimethylformamide, DMSO = N,N-dimethylsulphoxide

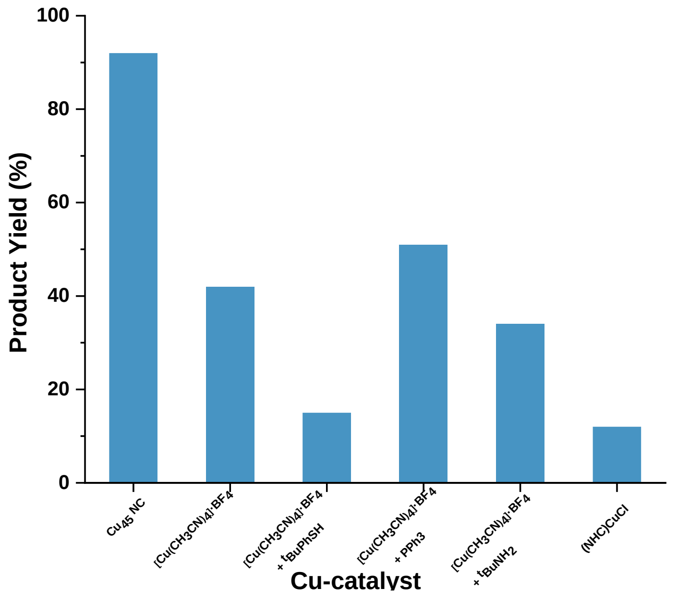

Figure S17. Comparisons of Cu_{45}NC with various Cu-complex for the hydroboration reaction. Cu-complexes= $[Cu(CH_3CN)_4]\cdot BF_4$ or $Cu(CH_3CN)_4]\cdot$BF4+ ^{t}BuPhSH or $Cu(CH_3CN)_4]\cdot$BF4+ PPh_3 or $Cu(CH_3CN)_4]\cdot$BF4+ $^tBuNH_2$ or (NHC)CuCl.

Table S3. Reaction Optimization for Internal Alkynes[a]

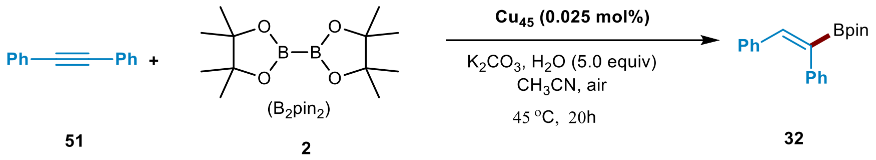

Entry	Variation from initial conditions	Yield of 3a (%)[b]
1	None	85 (83)
2	0.01 mol% **Cu_{45}**	66
3	in the presence of N_2	58
4	K_3PO_4	73
5	CH_3CN (without H_2O)	71
6	THF, dioxane, toluene, DMSO, or DMF as solvent	<70
7	Cu_{61} instead of **Cu_{45}**	63
8	CuCl instead of **Cu_{45}**	46[c]
9	no catalyst	0
10	no base	22

[a]Initial conditions: **42 (**0.3 mmol)**, 2 (**0.375 mmol)**,** Cu_{45} (0.025 mol%), K_2CO_3 1.2 eq., H_2O (5. 0 eq., and acetonitrile (CH_3CN) (4 mL, 0.075M). The mixture was stirred at 45 °C for 20 h under open air. [b] Yield refers to GC yield using 1,3,5-trimethylbenzene (mesitylene) as the internal standard, except for those in brackets which are isolated yields. [c]5 mol-% CuCl was used. THF=tetrahydrofuran, DMF = N,N-dimethylformamide, DMSO = N,N-dimethylsulphoxide

Table S4. Reaction Optimization for Terminal Alkynes[a]

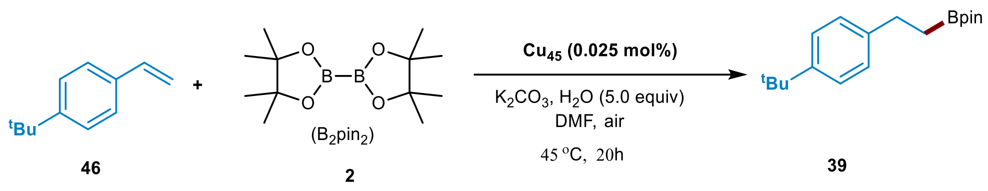

Entry	Variation from initial conditions	Yield of 3a (%)[b]
1	None	80 (78)
2	0.01 mol% **Cu_{45}**	64
3	in the presence of N_2	58
4	K_3PO_4	52
6	DMF (without H_2O)	58
7	THF, dioxane, toluene, DMSO, or CH_3CN as solvent	<65
8	Cu_{61} instead of **Cu_{45}**	55
9	CuCl instead of **Cu_{45}**	41[c]
10	no catalyst	0
11	no base	18

[a]Initial conditions: **43 (**0.3 mmol)**, 2a (**0.375 mmol)**,** Cu_{45} (0.025 mol%), K_2CO_3 1.2 eq., H_2O (5. 0 eq., and Dimethylformamide (DMF) (4 mL, 0.075M). The mixture was stirred at 45 °C for 20 h under open air. [b] Yield refers to GC yield using 1,3,5-trimethylbenzene (mesitylene) as the internal standard, except for those in brackets which are isolated yields. [c]5 mol-% CuCl was used. THF=tetrahydrofuran, CH_3CN= acetonitrile, DMSO = N,N-dimethylsulphoxide

Post Reaction Stability and Recyclability Study:

Scheme S3

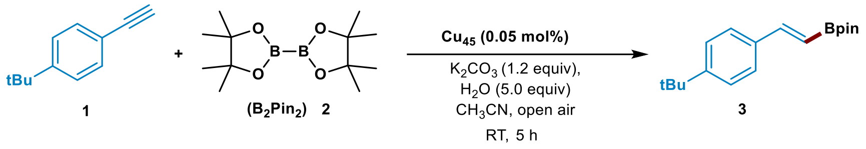

A 10 mL vial was charged with a magnetic stirrer bar, **Cu45**, (0.00015 mmol, 0.05 mol%), K_2CO_3 (1.2 equiv, 0.36 mmol), B_2pin_2 **2** (0.45 mmol, 1.5 equiv) and terminal alkyne **1** (0.3 mmol, 1.0 equiv), H_2O (5.0 equiv), and solvent acetonitrile (4.0 mL) was added to the vial. The reaction mixture was then stirred at room temperature (~30 °C) under an open-air atmosphere for 5 hrs. The reaction progress was monitored by GC-FID. After the completion of the reaction, the reaction mixture was transferred to a centrifuge tube and centrifuged for 10 minutes, the organic part was removed, and continue the same process by washing with 6 mL of acetonitrile/water (8:2). Then, subjected to analysis the ESI MS and UV-vis spectra all indicate that the **Cu45** is remained intact (Figure S19). The recovered $Cu_{45}NC$ dried under a high vacuum (to remove trace solvent). The recover/dried $Cu_{45}NC$ has been used for the next runs (upto 5 runs) (see, Figure S18).

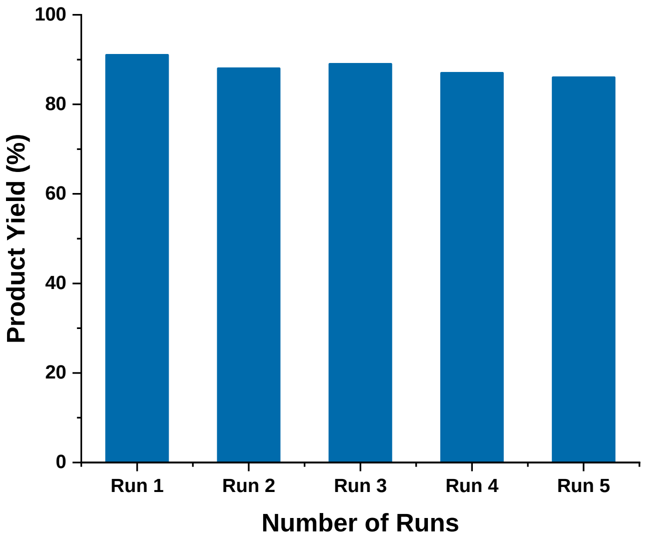

Figure S18. Catalyst stability and recyclability of $Cu_{45}NC$-catalyzed hydroboration reaction.

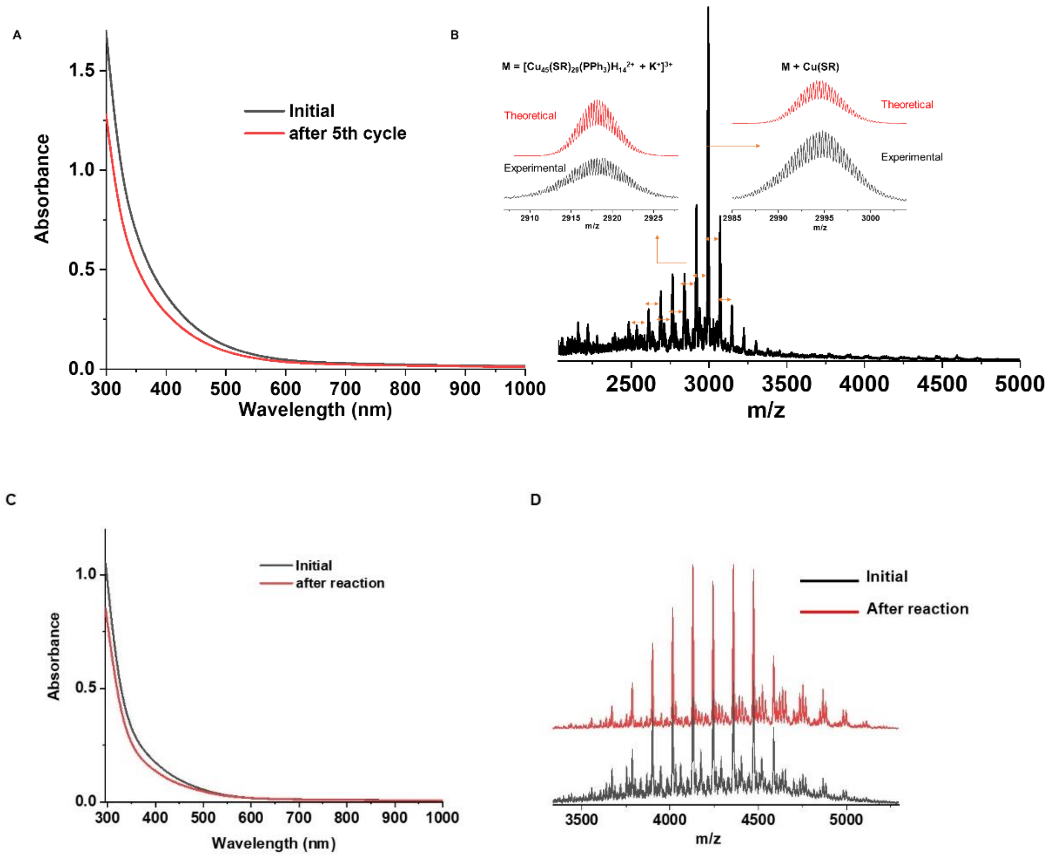

Figure S19. (A) UV-Vis spectra of Cu_{45} before and after reactions of five cycles. (B) ESI MS of Cu_{45} after reactions of five cycles. After reactions there is an attachment of potassium in the ESI MS and ionized in 3+ charge state. Matching of isotopic distribution of theoretical and experimental spectra confirms the assignments. Fragmentation pattern (loss and attachment of Cu(SR)) is akin to fresh Cu_{45}. Double headed arrows present the loss or attachment of one Cu(I)SR unit. (C) and (D) UV-Vis spectra and ESI MS of Cu_{45} before and after reaction with radical quenchers, respectively.

Stability of Cu_{45} catalyst under air/wet condition.

To further prove the stability of the **Cu_{45}** cluster under air, we conducted a control experiment: **Cu_{45}** was combined in acetonitrile solvent stirred for 5 hours under air/wet conditions. Subsequently, XPS measurements were performed to demonstrate whether the **Cu_{45}** cluster maintained the same oxidation state even under air/wet conditions. Notably, the XPS Cu LMM spectra indicated that **Cu_{45}** remained in the same oxidation state (Cu(I)) as fresh **Cu_{45}** sample (Figure S20). *These results would potentially support our claim that the* **Cu_{45}** *catalyst is highly stable under air/wet conditions compared to conventional Cu catalysts."*

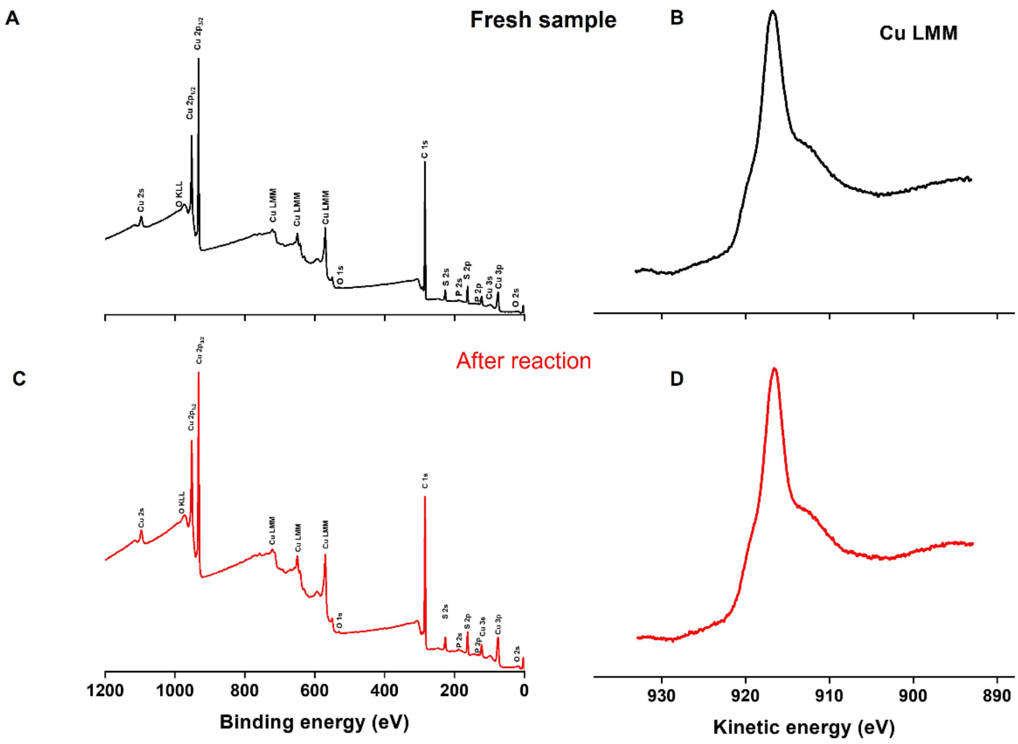

Figure S20. (A) & (B) correspond to XPS survey and Cu LMM spectra of fresh Cu_{45}. (C) & (D) illustrate the XPS survey and Cu LMM spectra of Cu_{45} after reaction with air and water.

Mechanistic Control Experiments

Radical Trap Experiment

Scheme S4

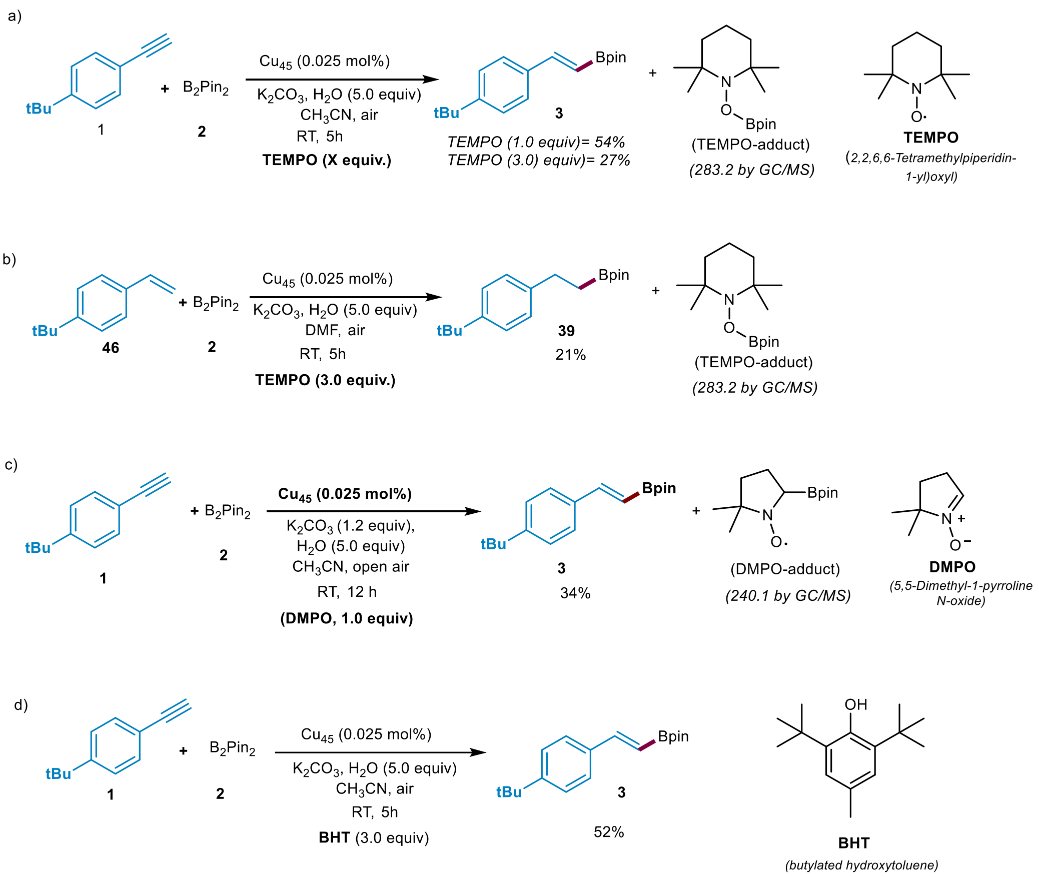

A 10 mL vial was charged with a magnetic stirrer bar, **Cu₄₅,** (0.000075 mmol , 0.025 mol%), K_2CO_3 (1.2 equiv, 0.36 mmol) and 4-^{t}Bu-phenylacetylene **1** (0.3 mmol, 1.0 equiv), B_2pin_2 **2** (0.45 mmol, 1.5 equiv), H_2O (5.0 equiv), and radical quenchers (TEMPO (1.0 or 3.0 equivalent) or BHT (3.0 equivalent) or DMPO (1.0 equivalent), and solvent acetonitrile (4.0 mL) was added to the vial. The reaction mixture was then stirred at room temperature (~30 °C) under an open-air atmosphere for 5 hrs. The mixture was filtered through celite and silica gel pads and washed with ethyl acetate. The filtrate was subjected to GC analysis, resulting the product **3** formation was reduced to 27 % (TEMPO), 52% (BHT), and 34% (DMPO). Notably, we have also detected the TEMPO-adducts (MW=283.2) and DMPO-adducts (MW= 240.1) by GC-MS (Figures S22-S24). This control experiments indicates that in line with the radical nature of the current **Cu_{45}** catalyzed hydroborylation reaction.

Radical Clock Experiment

Scheme S5

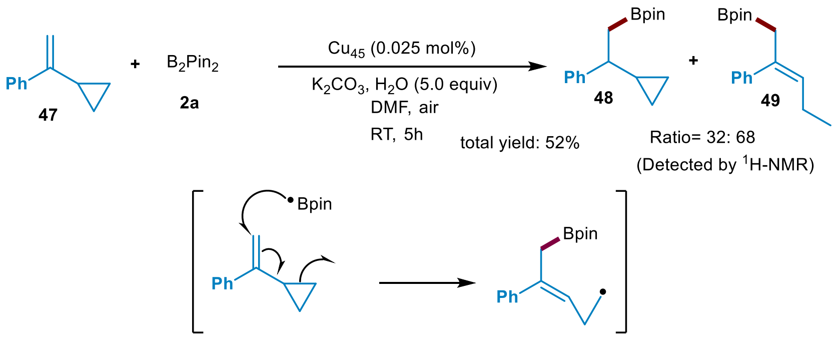

A 10 mL vial was charged with a magnetic stirrer bar, **Cu_{45}**, (0.000075 mmol, 0.025 mol%), K_2CO_3 (1.2 equiv, 0.36 mmol) and *1-cyclopropylvinyl)benzene* **47** (0.3 mmol, 1.0 equiv), B_2pin_2 **2** (0.45 mmol, 1.5 equiv), H_2O (5.0 equiv), and solvent DMF (4.0 mL) was added to the vial. The reaction mixture was then stirred at room temperature (~45 °C) under an open-air atmosphere for 12 hrs. The mixture was filtered through celite and silica gel pads and washed with ethyl acetate. The filtrate was concentrated, and the residue was subjected to the column chromatography on silica gel to remove unreacted starting materials (47 & B_2pin_2), and then collect the hydroborated products in the form of a colorless liquid, which consisted of a 32:68 ratio of products **48** & **49** respectively, determined by NMR analysis (relative integrations of the signals at 5.7 and 2.13 ppm were used) (Figure S21), and followed by GC/MS spectra analysis (MW=272.2) (Figures 25 & 26). *Thus, this control experiment suggests that the proposed reaction proceeds through the radical pathway.*

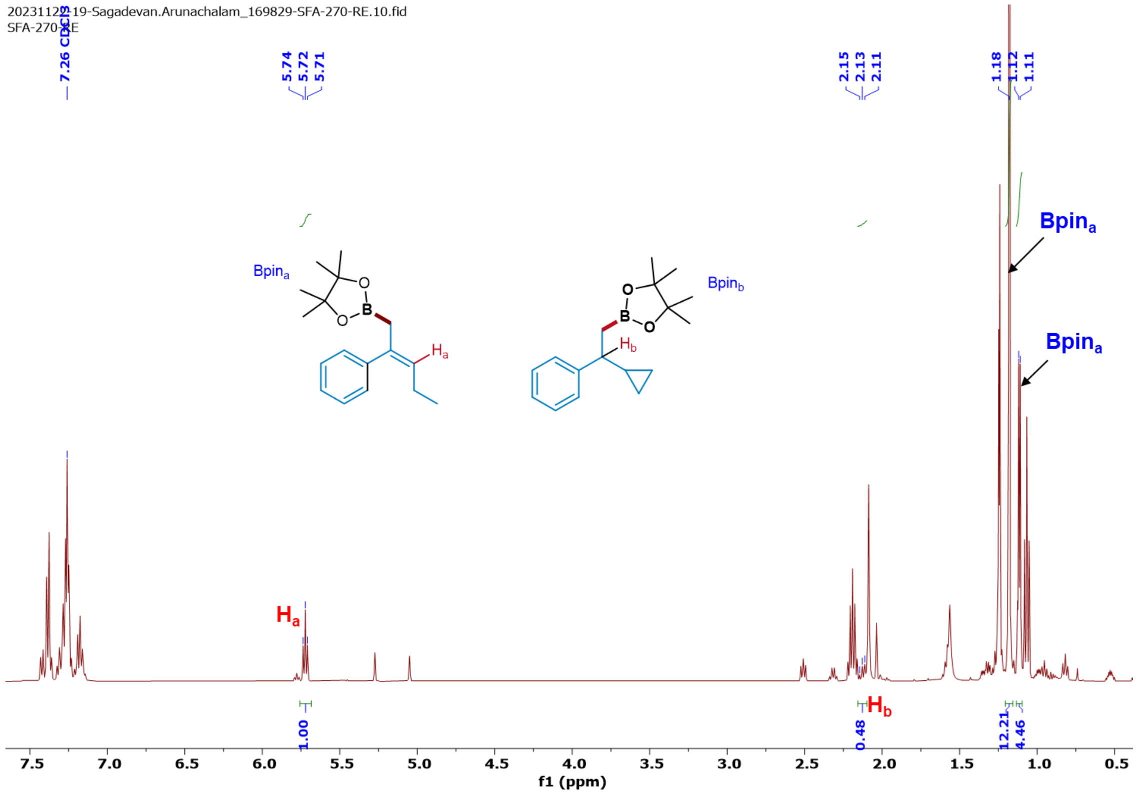

Figure S21: ^{1}H-NMR spectra of radical clock experiment results.

Radical Event in The Reaction

Scheme S6

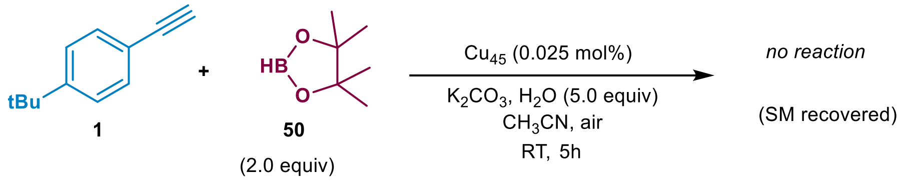

As per the standard conditions, the reaction of alkynes with the H-Bpin reagent instead of B_2Pin_2 in CH_3CN solvent under standard conditions. However, no product was obtained in these cases (Scheme S6), indicating that H-BPin is not a reactive species or intermediate **Note:** The aforementioned control experiments indicates rule out the possibilities of a two-electron process in the Cu_{45}-catalyzed hydroboration reactions and strongly suggest the involvement of a radical process.

Deuterium Labelling Experiment:

Scheme S7

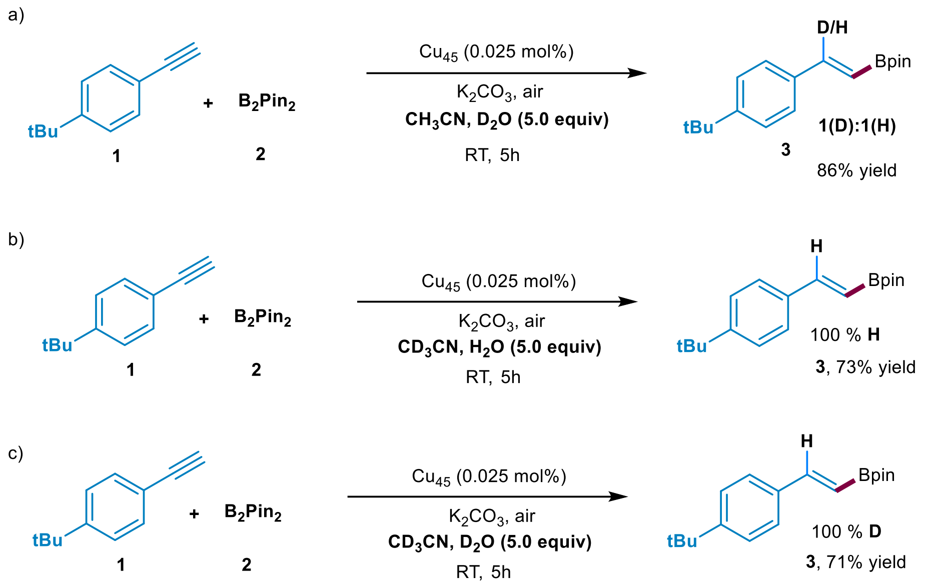

Three set of 10 mL vial was taken; a) first vial charged with a magnetic stirrer bar, $\mathbf{Cu_{45}}$, (0.000075 mmol, 0.025 mol%), K_2CO_3 (1.2 equiv, 0.36 mmol) and 4-^{t}Bu-phenylacetylene **1** (0.3 mmol, 1.0 equiv), B_2pin_2 **2** (0.45 mmol, 1.5 equiv), D_2O (5.0 equiv), and solvent acetonitrile (4.0 mL). b) Second vial charged with same as first vial except H_2O (5.0 equiv), and solvent deuterated acetonitrile CD_3CN (4.0 mL). c) Third vial charged with same as first vial except CD_3CN & (4.0 mL) D_2O (5.0 equiv). The reaction mixture was then stirred at room temperature (~30 °C) under an open-air atmosphere for 5 hrs. The mixture was filtered through celite and silica gel pads and washed with ethyl acetate. The filtrate was subjected to GC analysis and the first vial shows product 3 in 86% with a 1:1 ratio of (H/D) products, indicating that vinylic C-radical can be abstracting H atom from both CH_3CN and water and that H atom originating from both CH_3CN and H_2O. The second vial obtained in slightly less yield of product **3** in 73% but 100% H, with no deuterium exchanged. Third vial obtained in 71% yield with complete deuterium incorporated. This control study shows that H-abstraction may involve a hydrogen atom transfer (HAT) process with solvents (CH_3CN & H_2O), however, considering the Bond Dissociation Energy (BDE) of solvents, a HAT process is unlikely.[1] Thus, the vinyl C-radical **F** has the propensity to form their carbanion intermediate rather than involving a HAT process and subsequent protonation with water to form stable product (**3**) (Details see, proposed mechanism, Scheme 3).

Spectroscopic Data

(E)-2-(4-(tert-butyl)styryl)-4,4,5,5-tetramethyl-1,3,2-dioxaborolane (3)

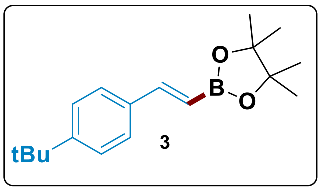

White solid, 90% yield; **^{1}H NMR** (500 MHz, $CDCl_3$) δ 7.43 (t, *J* = 8.8 Hz, 3H), 7.37 (d, *J* = 8.0 Hz, 2H), 6.13 (d, *J* = 18.4 Hz, 1H), 1.32 (s, 21H). **^{13}C NMR** (126 MHz, $CDCl_3$) δ 152.25, 149.53, 134.92, 126.97, 125.64, 115.51, 83.40, 34.83, 31.38, 24.95. **GCMS (EI)** m/z calcd for $C_{18}H_{27}BO_2$ [M^+] 286.2, found 286.2. The data are in accordance with those reported in the literature.[1-2]

(E)-4,4,5,5-tetramethyl-2-styryl-1,3,2-dioxaborolane (4)

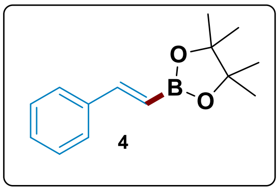

White solid, 84% yield; **^{1}H NMR** (400 MHz, $CDCl_3$) δ 7.49 (d, *J* = 6.6 Hz, 2H), 7.41 (d, *J* = 18.4 Hz, 1H), 7.37 – 7.27 (m, 3H), 6.18 (d, *J* = 18.5 Hz, 1H), 1.32 (s, 12H). **^{13}C NMR** (101 MHz, $CDCl_3$) δ 149.65, 137.57, 129.03, 128.75, 128.70, 127.19, 83.48, 24.93. **GCMS (EI)** m/z calcd for $C_{14}H_{19}BO_2$ [M^+] 230.1, found 230.2. The data are in accordance with those reported in the literature.[1-2]

(E)-2-(4-methoxystyryl)-4,4,5,5-tetramethyl-1,3,2-dioxaborolane (5)

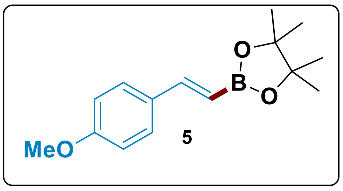

White solid, 86% yield; **^{1}H NMR (400 MHz, $CDCl_3$)** δ 7.47 – 7.40 (m, 2H), 7.35 (d, *J* = 18.4 Hz, 1H), 6.90 – 6.80 (m, 2H), 6.01 (d, *J* = 18.4 Hz, 1H), 3.80 (s, 3H), 1.30 (s, 12H). **^{13}C NMR (101 MHz, $CDCl_3$)** δ 160.40, 149.19, 130.49, 128.58, 114.07, 83.33, 55.38, 24.92. **GCMS (EI)** m/z calcd for $C_{15}H_{21}BO_3$ [M^+] 260.1, found 260.3. The data are in accordance with those reported in the literature.[1-2]

(E)-2-(4-ethylstyryl)-4,4,5,5-tetramethyl-1,3,2-dioxaborolane (6)

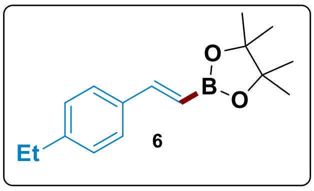

White solid, 82% yield; **^{1}H NMR (400 MHz, $CDCl_3$)** δ 7.45 – 7.35 (m, 3H), 7.17 (d, *J* = 8.2 Hz, 2H), 6.13 (d, *J* = 18.5 Hz, 1H), 2.64 (q, *J* = 7.6 Hz, 2H), 1.32 (s, 12H), 1.23 (t, *J* = 7.6 Hz, 3H). **^{13}C NMR (101 MHz, $CDCl_3$)** δ 149.64, 145.43, 135.14, 128.68, 128.21, 127.22, 83.39, 28.81, 24.93, 15.54. **GCMS (EI)** m/z calcd for $C_{16}H_{23}$BO2 [M^+] 258.2, found 258.3. The data are in accordance with those reported in the literature.[1-2]

(E)-2-(2-([1,1'-biphenyl]-4-yl)vinyl)-4,4,5,5-tetramethyl-1,3,2-dioxaborolane (7)

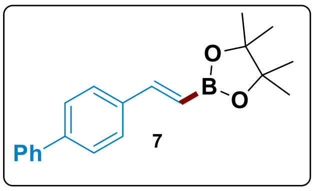

White solid, 92% yield; ^{1}H NMR (400 MHz, $CDCl_3$) δ 7.63 – 7.54 (m, 6H), 7.51 – 7.42 (m, 3H), 7.36 (t, J = 7.3 Hz, 1H), 6.23 (d, J = 18.4 Hz, 1H), 1.34 (s, 12H). ^{13}C NMR (101 MHz, $CDCl_3$) δ 149.13, 141.71, 140.66, 136.59, 128.92, 127.65, 127.58, 127.39, 127.34, 127.10, 83.49, 24.95. **GCMS (EI)** m/z calcd for $C_{20}H_{23}BO_2$ [M^+] 306.2, found 306.3. The data are in accordance with those reported in the literature.[1-2]

(E)-2-(4-chlorostyryl)-4,4,5,5-tetramethyl-1,3,2-dioxaborolane (8)

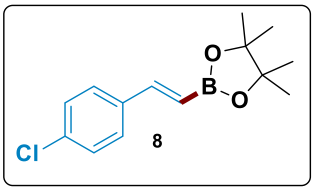

White solid, 82% yield; ^{1}H NMR (400 MHz, $CDCl_3$) δ 7.46 – 7.24 (m, 5H), 6.13 (d, J = 18.4 Hz, 1H), 1.30 (s, 12H). ^{13}C NMR (101 MHz, $CDCl_3$) δ 148.14, 136.06, 134.72, 128.91, 128.35, 117.26, 83.58, 24.92. **GCMS (EI)** m/z calcd for $C_{14}H_{18}BClO_2$ [M^+] 264.6, found 264.5. The data are in accordance with those reported in the literature.[1-3]

(E)-4-(2-(4,4,5,5-tetramethyl-1,3,2-dioxaborolan-2-yl)vinyl)benzonitrile (9)

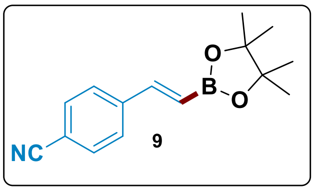

White solid, 72% yield; **^{1}H NMR** (400 MHz, $CDCl_3$) δ 7.61 (d, J = 8.4 Hz, 2H), 7.53 (d, J = 8.4 Hz, 2H), 7.35 (d, J = 18.3 Hz, 1H), 6.26 (d, J = 18.5 Hz, 1H), 1.30 (s, 12H). **^{13}C NMR** (101 MHz, $CDCl_3$) δ 147.18, 141.73, 132.53, 127.51, 121.03, 118.89, 112.06, 83.81, 83.42, 24.89. **GCMS (EI)** m/z calcd for $C_{15}H_{18}BNO_2$ [M^+] 255.1, found 255.3. The data are in accordance with those reported in the literature.[1-3]

Methyl (E)-4-(2-(4,4,5,5-tetramethyl-1,3,2-dioxaborolan-2-yl)vinyl)benzoate (10)

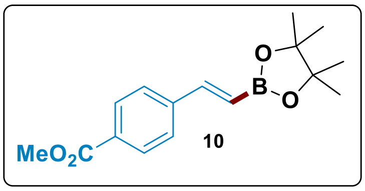

White solid, 74% yield; **^{1}H NMR** (400 MHz, $CDCl_3$) δ 7.99 (d, J = 8.4 Hz, 2H), 7.52 (d, J = 8.4 Hz, 2H), 7.40 (d, J = 18.4 Hz, 1H), 6.26 (d, J = 18.4 Hz, 1H), 3.89 (s, 3H), 1.30 (s, 12H). **^{13}C NMR** (101 MHz, $CDCl_3$) δ 166.86, 148.23, 141.78, 130.21, 130.01, 126.99, 119.63, 83.65, 52.22, 24.90. **GCMS (EI)** m/z calcd for $C_{16}H_{21}BO_4$ [M^+] 288.2, found 288.2. The data are in accordance with those reported in the literature.[1-2]

(E)-4,4,5,5-tetramethyl-2-(4-(trifluoromethoxy)styryl)-1,3,2-dioxaborolane (11)

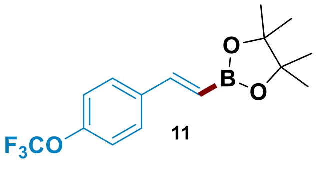

11

White solid, 78% yield; **^{1}H NMR** (400 MHz, $CDCl_3$) δ 7.53 – 7.44 (m, 2H), 7.36 (d, *J* = 18.4 Hz, 1H), 7.22 – 7.13 (m, 2H), 6.14 (d, *J* = 18.4 Hz, 1H), 1.31 (s, 12H). **^{13}C NMR** (101 MHz, $CDCl_3$) δ 149.57 (d, *J* = 2.3 Hz), 147.86, 136.27, 128.46, 121.84, 121.08, 119.29, 117.76, 83.63, 24.91. **GCMS (EI)** m/z calcd for $C_{15}H_{18}BF_3O_3$ [M^+] 314.1, found 314.2. The data are in accordance with those reported in the literature.[2, 4]

(E)-4,4,5,5-tetramethyl-2-(2,4,5-trimethylstyryl)-1,3,2-dioxaborolane (12)

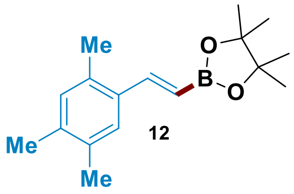

12

White solid, 90% yield; **^{1}H NMR** (400 MHz, $CDCl_3$) δ 7.63 (d, *J* = 18.3 Hz, 1H), 7.37 (s, 1H), 6.94 (s, 1H), 6.07 (d, *J* = 18.3 Hz, 1H), 2.38 (s, 3H), 2.24 (d, *J* = 3.1 Hz, 6H), 1.34 (s, 12H). **^{13}C NMR** (101 MHz, $CDCl_3$) δ 147.27, 137.40, 134.17, 134.12, 133.81, 131.90, 127.10, 116.71, 83.29, 24.92, 19.56, 19.47, 19.25. **GCMS (EI)** m/z calcd for $C_{17}H_{25}BO_2$ [M^+] 272.2, found 272.3. The data are in accordance with those reported in the literature.[1].

(E)-4,4,5,5-tetramethyl-2-(3-methylstyryl)-1,3,2-dioxaborolane (13)

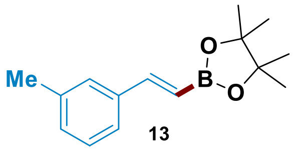

13

White solid, 88% yield; **^{1}H NMR** (400 MHz, $CDCl_3$) δ 7.39 (d, *J* = 18.5 Hz, 1H), 7.33 – 7.27 (m, 3H), 7.23 (t, *J* = 7.8 Hz, 1H), 7.11 (d, *J* = 7.4 Hz, 1H), 6.16 (d, *J* = 18.4 Hz, 1H), 2.35 (s, 3H), 1.32 (s, 12H). **^{13}C NMR** (101 MHz, $CDCl_3$) δ 149.79, 138.18, 137.54, 129.82, 128.56, 127.88, 124.34, 116.26, 83.40, 24.91, 21.50. **GCMS (EI)** m/z calcd for $C_{15}H_{21}BO_2$ [M^+] 244.1, found 244.2. The data are in accordance with those reported in the literature.[1-2]

(E)-3-(2-(4,4,5,5-tetramethyl-1,3,2-dioxaborolan-2-yl)vinyl)phenol (14)

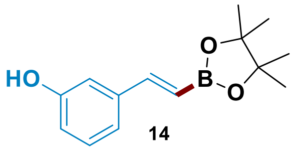

14

White solid, 87% yield; **^{1}H NMR** (400 MHz, $CDCl_3$) δ 7.33 (d, *J* = 18.4 Hz, 1H), 7.20 (t, *J* = 7.8 Hz, 1H), 7.05 (d, *J* = 7.6 Hz, 1H), 6.95 (d, *J* = 2.5 Hz, 1H), 6.78 (d, *J* = 8.0 Hz, 1H), 6.13 (d, *J* = 18.4 Hz, 1H), 5.14 (s, 1H), 1.31 (s, 12H). **^{13}C NMR** (101 MHz, $CDCl_3$) δ 155.92, 149.25, 139.30, 129.93, 120.16, 116.17, 113.51, 83.60, 24.93. **GCMS (EI)** m/z calcd for $C_{14}H_{19}BO_3$ [M^+] 246.1, found 246.2. The data are in accordance with those reported in the literature.[1, 5]

(E)-3-(2-(4,4,5,5-tetramethyl-1,3,2-dioxaborolan-2-yl)vinyl)aniline (15)

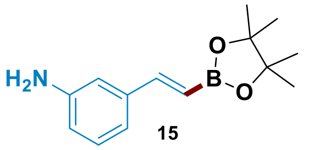

Brown solid, 88% yield; **^{1}H NMR** (500 MHz, $CDCl_3$) δ 7.31 (d, *J* = 18.4 Hz, 1H), 7.12 (t, *J* = 7.8 Hz, 1H), 6.90 (d, *J* = 7.7 Hz, 1H), 6.80 (s, 1H), 6.62 (dd, *J* = 7.5, 1.9 Hz, 1H), 6.10 (d, *J* = 18.4 Hz, 1H), 3.59 (s, 2H), 1.31 (s, 12H). **^{13}C NMR** (126 MHz, $CDCl_3$) δ 149.85, 146.63, 138.67, 129.55, 118.01, 116.01, 113.48, 83.41, 24.92. **GCMS (EI)** m/z calcd for $C_{14}H_{20}BNO_2$ [M^+] 245.2, found 245.3. The data are in accordance with those reported in the literature.[1-2]

(E)-2-(3,5-difluorostyryl)-4,4,5,5-tetramethyl-1,3,2-dioxaborolane (16)

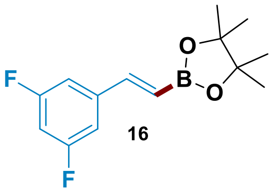

White solid, 85% yield; **^{1}H NMR** (400 MHz, $CDCl_3$) δ 7.26 (d, *J* = 18.3 Hz, 1H), 6.96 (dt, *J* = 6.8, 2.1 Hz, 2H), 6.72 (tt, *J* = 8.8, 2.3 Hz, 1H), 6.15 (d, *J* = 18.4 Hz, 1H), 1.30 (s, 12H). **^{13}C NMR** (101 MHz, $CDCl_3$) δ 164.51 (d, *J* = 12.8 Hz), 162.04 (d, *J* = 12.7 Hz), 146.95 (t, *J* = 2.8 Hz), 141.02 (t, *J* = 9.2 Hz), 119.73, 110.46 – 109.58 (m), 104.08 (t, *J* = 25.7 Hz), 83.77, 24.90. **GCMS (EI)** m/z calcd for $C_{14}H_{17}BF_2O_2$ [M^+] 266.1, found 266.3. The data are in accordance with those reported in the literature.[1-2]

(E)-2-(2-fluorostyryl)-4,4,5,5-tetramethyl-1,3,2-dioxaborolane (17)

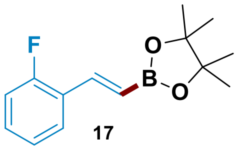

Colorless pasty liquid, 87% yield; **^{1}H NMR** (500 MHz, $CDCl_3$) δ 7.62 – 7.53 (m, 2H), 7.29 – 7.21 (m, 1H), 7.11 (td, *J* = 7.6, 1.2 Hz, 1H), 7.03 (ddd, *J* = 10.7, 8.2, 1.2 Hz, 1H), 6.24 (d, *J* = 18.6 Hz, 1H), 1.31 (s, 12H). **^{13}C NMR** (126 MHz, $CDCl_3$) δ 161.84, 159.84, 141.46, 141.43, 130.34, 130.27, 127.53, 127.51, 125.55, 125.46, 124.24, 124.21, 119.35, 116.03, 115.85, 83.57, 24.93.**GCMS (EI)** m/z calcd for $C_{14}H_{18}BFO_2$ [M^+] 248.1, found 248.1. The data are in accordance with those reported in the literature.[1, 6]

(E)-2-(2-bromostyryl)-4,4,5,5-tetramethyl-1,3,2-dioxaborolane (18)

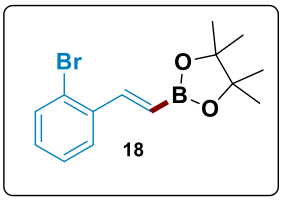

Colorless pasty solid, 79% yield; **^{1}H NMR** (500 MHz, $CDCl_3$) δ 7.71 (d, *J* = 18.2 Hz, 1H), 7.61 (dd, *J* = 7.8, 1.7 Hz, 1H), 7.55 (dd, *J* = 8.0, 1.3 Hz, 1H), 7.28 (t, *J* = 6.9 Hz, 1H), 7.13 (td, *J* = 7.7, 1.7 Hz, 1H), 6.12 (d, *J* = 18.3 Hz, 1H), 1.32 (s, 12H). **^{13}C NMR** (126 MHz, $CDCl_3$) δ 147.66, 137.53, 133.18, 130.02, 127.63, 127.42, 124.44, 120.20, 83.63, 24.96. **GCMS (EI)** m/z calcd for $C_{14}H_{18}BBrO_2$ [M^+] 308.1, found 308.1 & 310.1. The data are in accordance with those reported in the literature.[1, 5]

(E)-2-(2-(4,4,5,5-tetramethyl-1,3,2-dioxaborolan-2-yl)vinyl)aniline (19)

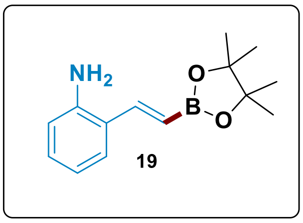

Brown solid, 80% yield; **^{1}H NMR** (500 MHz, $CDCl_3$) δ 7.50 (d, *J* = 18.3 Hz, 1H), 7.38 (d, *J* = 7.8 Hz, 1H), 7.13 – 7.06 (m, 1H), 6.75 (t, *J* = 7.5 Hz, 1H), 6.66 (d, *J* = 9.2 Hz, 1H), 6.06 (d, *J* = 18.2 Hz, 1H), 3.89 (s, 2H), 1.31 (s, 12H). **^{13}C NMR** (126 MHz, $CDCl_3$) δ 144.95, 144.58, 129.88, 127.55, 123.60, 118.87, 117.88, 116.45, 83.42, 24.93. **GCMS (EI)** m/z calcd for $C_{14}H_{20}BNO_2$ [M^+] 245.2, found 245.1. The data are in accordance with those reported in the literature.[1-2]

(E)-4,4,5,5-tetramethyl-2-(2-(trifluoromethyl)styryl)-1,3,2-dioxaborolane (20)

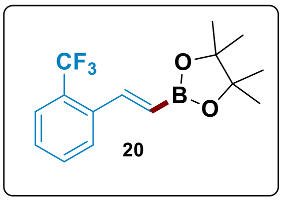

White solid, 83% yield; **^{1}H NMR** (400 MHz, $CDCl_3$) δ 7.80 – 7.68 (m, 2H), 7.63 (d, *J* = 7.8 Hz, 1H), 7.51 (t, *J* = 7.6 Hz, 1H), 7.37 (t, *J* = 7.8 Hz, 1H), 6.16 (d, *J* = 18.0 Hz, 1H), 1.31 (s, 12H). **^{13}C NMR** (101 MHz, $CDCl_3$) δ 144.85, 144.83, 137.01, 132.01, 131.51, 130.79, 128.33, 127.98, 127.68, 127.59, 125.81, 125.75, 125.70, 122.98, 83.67, 83.38, 24.95, 24.89, 24.56. **GCMS (EI)** m/z calcd for $C_{15}H_{18}BF_3O_2$ [M^+] 298.1, found 298.3. The data are in accordance with those reported in the literature.[1].

(E)-2-(2-(6-methoxynaphthalen-2-yl)vinyl)-4,4,5,5-tetramethyl-1,3,2-dioxaborolane (21)

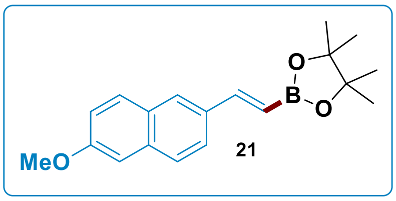

White solid, 86% yield; ^{1}H NMR (400 MHz, $CDCl_3$) δ 7.78 (s, 1H), 7.72 (d, *J* = 8.9 Hz, 1H), 7.69 (s, 2H), 7.57 (d, *J* = 18.4 Hz, 1H), 7.22 – 6.99 (m, 2H), 6.26 (d, *J* = 18.4 Hz, 1H), 3.90 (s, 3H), 1.34 (s, 12H). **^{13}C NMR** (101 MHz, $CDCl_3$) δ 158.26, 149.77, 135.09, 133.02, 130.02, 128.88, 127.96, 127.19, 124.08, 119.11, 115.57, 105.92, 83.39, 55.37, 24.91. **GCMS (EI)** m/z calcd for $C_{19}H_{23}BO_3$ [M^+] 310.2, found 310.3. The data are in accordance with those reported in the literature.[1].

(E)-3-(2-(4,4,5,5-tetramethyl-1,3,2-dioxaborolan-2-yl)vinyl)pyridine (22)

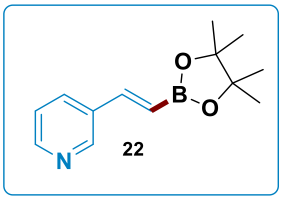

Off-white pasty solid, 79% yield; **^{1}H NMR** (500 MHz, $CDCl_3$) δ 8.72 (s, 1H), 8.56 (s, 1H), 7.80 (d, *J* = 7.9 Hz, 1H), 7.37 (d, *J* = 18.5 Hz, 1H), 7.29 (s, 1H), 6.25 (d, *J* = 18.4 Hz, 1H), 1.31 (s, 12H). **^{13}C NMR** (126 MHz, $CDCl_3$) δ 149.73, 149.12, 145.81, 133.39, 125.16, 123.90, 119.57, 83.76, 24.94. **GCMS (EI)** m/z calcd for $C_{13}H_{18}BNO_2$ [M^+] 231.1, found 231.1. The data are in accordance with those reported in the literature.[3]

(E)-4,4,5,5-tetramethyl-2-(2-(thiophen-3-yl)vinyl)-1,3,2-dioxaborolane (23)

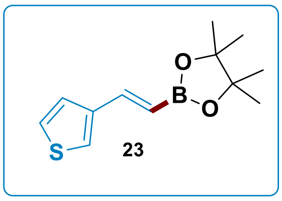

Off-white pasty solid, 84% yield; **^{1}H NMR** (500 MHz, $CDCl_3$) δ 7.38 (d, *J* = 18.4 Hz, 1H), 7.33 – 7.28 (m, 2H), 7.28 – 7.22 (m, 1H), 5.94 (d, *J* = 18.4 Hz, 1H), 1.30 (s, 12H). **^{13}C NMR** (126 MHz, $CDCl_3$) δ 143.29, 141.37, 126.25, 125.17, 125.00, 116.27, 83.45, 24.94.. **GCMS (EI)** m/z calcd for $C_{12}H_{17}BO_2S$ [M^+] 236.1, found 236.1. The data are in accordance with those reported in the literature.[1, 6]

(E)-4,4,5,5-tetramethyl-2-(3-methylbuta-1,3-dien-1-yl)-1,3,2-dioxaborolane (24)

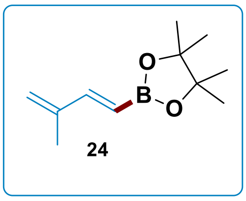

Colorless liquid, 75% yield**; ^{1}H NMR** (500 MHz, $CDCl_3$) δ 7.11 (d, *J* = 18.2 Hz, 1H), 5.56 (d, *J* = 18.2 Hz, 1H), 5.18 – 5.12 (m, 2H), 1.85 (s, 2H), 1.28 (s, 12H).**^{13}C NMR** (126 MHz, $CDCl_3$) δ 152.36, 143.19, 120.26, 83.39, 24.94, 17.87. **GCMS (EI)** m/z

calcd for $C_{11}H_{19}BO_2$ [M^+] 194.1, found 194.2 The data are in accordance with those reported in the literature.[7]

(E)-2-(2-cyclohexylvinyl)-4,4,5,5-tetramethyl-1,3,2-dioxaborolane (25)

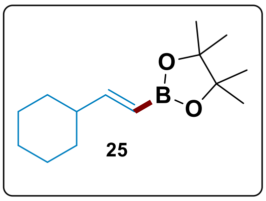

Colorless liquid, 78% yield; **^{1}H NMR** (500 MHz, $CDCl_3$) δ 6.56 (dd, J = 18.2, 6.2 Hz, 1H), 5.36 (dd, J = 18.2, 1.5 Hz, 1H), 2.06 – 1.96 (m, 1H), 1.76 – 1.67 (m, 4H), 1.63 (ddt, J = 12.6, 3.5, 1.8 Hz, 1H), 1.25 (s, 15H), 1.15 – 1.02 (m, 3H). **^{13}C NMR** (126 MHz, $CDCl_3$) δ 159.96, 115.54, 83.09, 43.37, 32.05, 26.30, 26.09, 24.91. **GCMS (EI)** m/z calcd for $C_{14}H_{25}BO_2$ [M^+] 236.2, found 236.3 The data are in accordance with those reported in the literature.[1, 6]

(E)-2-(2-cyclopropylvinyl)-4,4,5,5-tetramethyl-1,3,2-dioxaborolane (26)

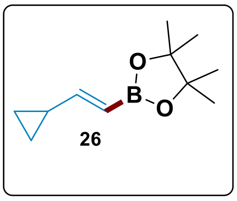

Colorless liquid, 76% yield; **^{1}H NMR** (500 MHz, $CDCl_3$) δ 6.07 (dd, J = 17.8, 9.3 Hz, 1H), 5.49 (d, J = 17.8 Hz, 1H), 1.51 (dddd, J = 12.7, 9.3, 8.0, 4.7 Hz, 1H), 1.25 (s, 12H), 0.85 – 0.75 (m, 2H), 0.56 – 0.50 (m, 2H). **^{13}C NMR** (126 MHz, $CDCl_3$) δ 158.73, 83.07, 24.89, 17.16, 8.03. **GCMS (EI)** m/z calcd for $C_{11}H_{19}BO_2$ [M^+] 194.1, found 194.1 The data are in accordance with those reported in the literature.[1-2, 6]

(E)-4,4,5,5-tetramethyl-2-(4-phenylbut-1-en-1-yl)-1,3,2-dioxaborolane (27)

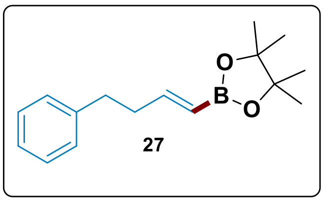

Colorless pasty solid, 79% yield; **^{1}H NMR** (500 MHz, $CDCl_3$) δ 7.28 (t, J = 7.5 Hz, 2H), 7.19 (d, J = 7.4 Hz, 3H), 6.71 (dt, J = 18.0, 6.2 Hz, 1H), 5.51 (d, J = 18.0 Hz, 1H), 2.75 (dd, J = 9.6, 6.6 Hz, 2H), 2.48 (q, J = 7.9, 7.2 Hz, 2H), 1.28 (s, 12H). **^{13}C NMR** (126 MHz, $CDCl_3$) δ 153.54, 141.90, 128.46, 125.97, 119.16, 83.20, 37.61, 34.71, 24.91. **GCMS (EI)** m/z calcd for $C_{16}H_{23}BO_2$ [M^+] 258.2, found 258.3 The data are in accordance with those reported in the literature.[1, 8]

(E)-2-(5-(4,4,5,5-tetramethyl-1,3,2-dioxaborolan-2-yl)pent-4-en-1-yl)isoindoline-1,3-dione (28)

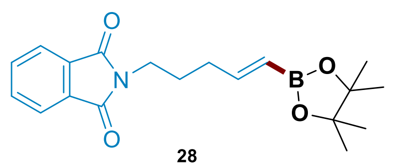

28

Brown pasty solid, 76% yield; **^{1}H NMR** (500 MHz, $CDCl_3$) δ 7.28 (t, *J* = 7.5 Hz, 2H), 7.19 (d, *J* = 7.4 Hz, 3H), 6.71 (dt, *J* = 18.0, 6.2 Hz, 1H), 5.51 (d, *J* = 18.0 Hz, 1H), 2.75 (dd, *J* = 9.6, 6.6 Hz, 2H), 2.48 (q, *J* = 7.9, 7.2 Hz, 2H), 1.28 (s, 12H). **^{13}C NMR** (126 MHz, $CDCl_3$) δ 153.54, 141.90, 128.46, 125.97, 119.16, 83.20, 37.61, 34.71, 24.91. **GCMS (EI)** m/z calcd for $C_{19}H_{24}BNO_2$ [M^+] 341.2, found 341.3 The data are in accordance with those reported in the literature.[1, 8-9]

(E)-2-(3-(4-allyl-2-methoxyphenoxy)prop-1-en-1-yl)-4,4,5,5-tetramethyl-1,3,2-dioxaborolane (29)

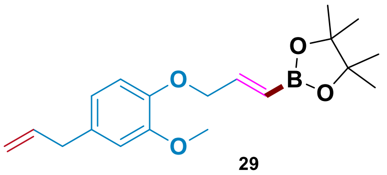

29

Colorless pasty liquid, 81% yield; **^{1}H NMR** (500 MHz, $CDCl_3$) δ 7.28 (t, *J* = 7.5 Hz, 2H), 7.19 (d, *J* = 7.4 Hz, 3H), 6.71 (dt, *J* = 18.0, 6.2 Hz, 1H), 5.51 (d, *J* = 18.0 Hz, 1H), 2.75 (dd, *J* = 9.6, 6.6 Hz, 2H), 2.48 (q, *J* = 7.9, 7.2 Hz, 2H), 1.28 (s, 12H). **^{13}C NMR** (126 MHz, $CDCl_3$) δ 153.54, 141.90, 128.46, 125.97, 119.16, 83.20, 37.61, 34.71, 24.91. **GCMS (EI)** m/z calcd for $C_{19}H_{27}BO_4$ [M^+] 330.2, found 330.3 The data are in accordance with those reported in the literature.

(E)-2-(4-(tert-butyl)styryl)-4,4,6,6-tetramethyl-1,3,2-dioxaborinane (30)

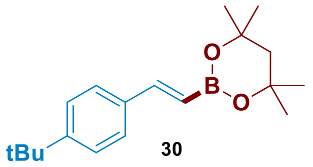

30

White solid, 73% yield; ^{1}H NMR (500 MHz, $CDCl_3$) δ 7.42 (d, *J* = 8.4 Hz, 2H), 7.37 – 7.29 (m, 2H), 7.27 (d, *J* = 8.0 Hz, 1H), 6.08 (d, *J* = 18.1 Hz, 1H), 1.40 (s, 12H), 1.31 (s, 9H). **^{13}C NMR** (126 MHz, $CDCl_3$) δ 151.49, 146.41, 135.48, 126.84, 125.51, 70.77, 49.15, 34.77, 31.96, 31.42. **GCMS (EI)** m/z calcd for $C_{19}H_{29}BO_2$ [M^+] 300.2, found 300.3. The data are in accordance with those reported in the literature.[10]

(E)-2-(4-(tert-butyl)styryl)-5,5-dimethyl-1,3,2-dioxaborinane (31)

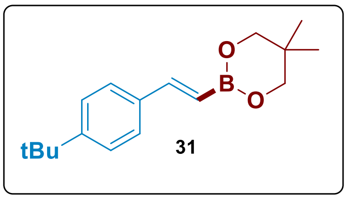

White solid, 77% yield; **^{1}H NMR** (500 MHz, $CDCl_3$) δ 7.43 (d, *J* = 8.2 Hz, 2H), 7.38 – 7.32 (m, 2H), 7.30 (s, 1H), 6.06 (d, *J* = 18.3 Hz, 1H), 3.70 (d, *J* = 1.3 Hz, 4H), 1.32 (s, 9H), 1.01 (s, 6H). **^{13}C NMR** (126 MHz, $CDCl_3$) δ 151.85, 147.13, 135.22, 126.90, 125.59, 72.33, 34.80, 32.00, 31.40, 29.85, 22.03. **GCMS (EI)** m/z calcd for $C_{17}H_{25}BO_2$ [M^+] 272.2, found 272.2. The data are in accordance with those reported in the literature.[10]

(Z)-2-(1,2-diphenylvinyl)-4,4,5,5-tetramethyl-1,3,2-dioxaborolane (32)

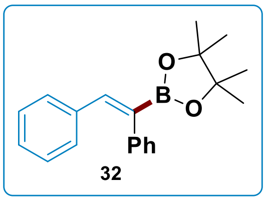

White solid, 83% yield; ^{1}H NMR (500 MHz, $CDCl_3$) δ 7.36 (s, 1H), 7.26 (t, *J* = 7.2 Hz, 3H), 7.20 (t, *J* = 7.3 Hz, 1H), 7.19 – 7.15 (m, 2H), 7.13 – 7.08 (m, 3H), 7.05 (dd, *J* = 6.6, 3.0 Hz, 2H), 1.31 (s, 12H). **^{13}C NMR** (126 MHz, $CDCl_3$) δ 143.30, 140.57, 137.13, 130.10, 129.00, 128.38, 127.99, 127.72, 126.40, 83.93, 24.93. **GCMS (EI)** m/z calcd for $C_{20}H_{23}BO_2$ [M^+] 306.2, found 306.3. The data are in accordance with those reported in the literature.[1]

2-(1,2-bis(4-bromophenyl)vinyl)-4,4,5,5-tetramethyl-1,3,2-dioxaborolane (33)

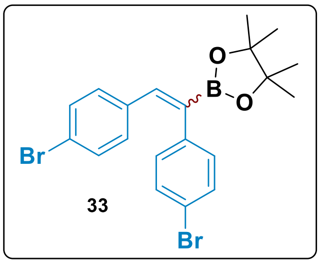

White solid, 73% yield; **^{1}H NMR** (500 MHz, $CDCl_3$) δ 7.49 – 7.42 (m, 1H), 7.39 (d, *J* = 6.9 Hz, 2H), 7.36 – 7.24 (m, 5H), 7.01 (d, *J* = 8.4 Hz, 2H), 6.92 (d, *J* = 8.1 Hz, 2H), 1.30 (s, 12H). ^{13}C NMR (126 MHz, $CDCl_3$) δ 143.95, 142.49, 141.51, 140.36, 138.97, 137.48, 135.65, 132.66, 131.70, 131.55, 131.50, 131.43, 131.40, 131.38, 130.91, 130.76, 130.04, 128.77, 122.04, 121.95, 121.29, 120.70, 84.47, 84.20, 24.92.**GCMS (EI)** m/z calcd for $C_{20}H_{21}BBrO_2$ [M^+] 464.0, found 464.1. The data are in accordance with those reported in the literature.[11]

(Z)-2-(1-(4-methoxyphenyl)-2-phenylvinyl)-4,4,5,5-tetramethyl-1,3,2-dioxaborolane (34) and (Z)-2-(2-(4-methoxyphenyl)-1-phenylvinyl)-4,4,5,5-tetramethyl-1,3,2-dioxaborolane (34')

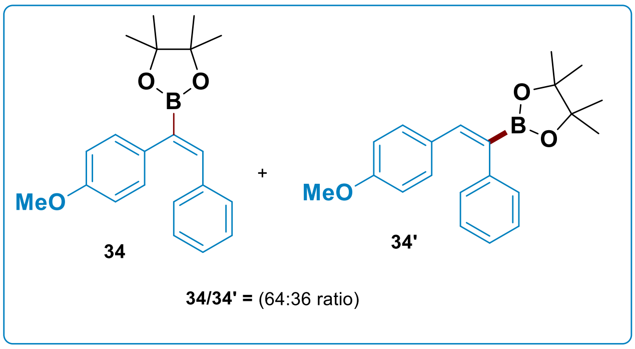

White solid, 34/34', 87% yield, (64:36 ratio); **^{1}H NMR** (500 MHz, $CDCl_3$) δ 7.29 (dd, *J* = 17.4, 8.0 Hz, 3H), 7.20 (dd, *J* = 20.5, 7.1 Hz, 2H), 7.11 (dq, *J* = 12.6, 3.8 Hz, 6H), 7.00 (d, *J* = 8.4 Hz, 1H), 6.81 (d, *J* = 8.5 Hz, 2H), 6.65 (d, *J* = 8.7 Hz, 1H), 3.77 (d, *J* = 31.5 Hz, 3H), 1.30 (d, *J* = 6.6 Hz, 12H). **^{13}C NMR** (126 MHz, $CDCl_3$) δ 159.21, 158.28, 142.89, 142.82, 141.03, 137.37, 132.67, 131.65, 130.22, 130.02, 129.77, 128.97, 128.47, 128.00, 127.57, 126.26, 113.84, 113.42, 83.88, 83.79, 55.25, 24.94, 24.92. **GCMS (EI)** m/z calcd for $C_{21}H_{25}BO_3$ [M^+] 336.2, found 336.2. The data are in accordance with those reported in the literature.[9-10, 12]

(Z)-2-(2-(4-chlorophenyl)-1-phenylvinyl)-4,4,5,5-tetramethyl-1,3,2-dioxaborolane (35) and (Z)-2-(1-(4-chlorophenyl)-2-phenylvinyl)-4,4,5,5-tetramethyl-1,3,2-dioxaborolane (35')

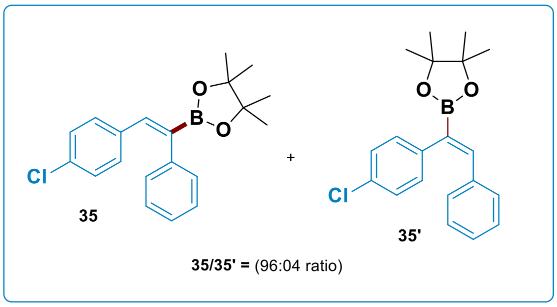

White solid, 35/35', 82% yield, (96:04 ratio); **^{1}H NMR** (500 MHz, $CDCl_3$) δ 7.42 – 7.19 (m, 4H), 7.17 – 7.03 (m, 5H), 7.00 – 6.94 (m, 1H), 1.31 (s, 12H).**^{13}C NMR** (126 MHz, $CDCl_3$) δ 143.96, 141.83, 140.14, 138.95, 136.78, 135.57, 133.40, 131.29, 130.52, 130.02, 128.86, 128.61, 128.51, 128.23, 128.13, 127.95, 126.62, 84.06, 84.03, 24.92. **GCMS (EI)** m/z calcd for $C_{20}H_{22}BClO_2$ [M^+] 340.1, found 340.2. The data are in accordance with those reported in the literature[9, 12-13].

(Z)-2-(1-(3,5-dimethylphenyl)-2-phenylvinyl)-4,4,5,5-tetramethyl-1,3,2-dioxaborolane (36) and (Z)-2-(2-(3,5-dimethylphenyl)-1-phenylvinyl)-4,4,5,5-tetramethyl-1,3,2-dioxaborolane (36')

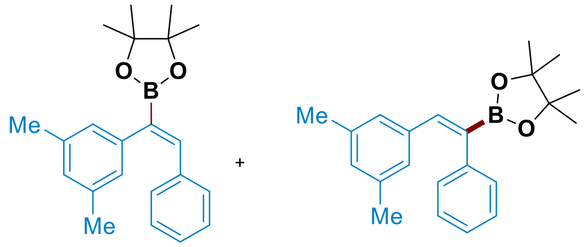

36 36'
36/36' = (55:45 ratio)

White solid, 36/36', 88% yield, (55:45 ratio); ^{1}**H NMR** (500 MHz, $CDCl_3$) δ 7.33 (s, 1H), 7.30 – 7.25 (m, 1H), 7.20 (dd, *J* = 20.8, 7.6 Hz, 2H), 7.12 (d, *J* = 3.2 Hz, 2H), 7.09 (d, *J* = 6.4 Hz, 1H), 6.86 (s, 1H), 6.78 (s, 2H), 6.67 (s, 1H), 2.26 (s, 4H), 2.12 (s, 3H), 1.32 (s, 15H). ^{13}**C NMR** (126 MHz, $CDCl_3$) δ 143.66, 142.86, 140.85, 140.41, 137.62, 137.21, 137.18, 136.89, 130.12, 129.46, 128.89, 128.25, 128.18, 128.06, 127.91, 127.65, 126.47, 126.28, 83.82, 24.91, 24.89, 21.51, 21.29. **GCMS (EI)** m/z calcd for $C_{22}H_{27}BO_2$ [M^+] 334.3, found 334.2. The data are in accordance with those reported in the literature.[9, 12, 14]

(Z)-4,4,5,5-tetramethyl-2-(2-(naphthalen-1-yl)-1-phenylvinyl)-1,3,2-dioxaborolane (37)

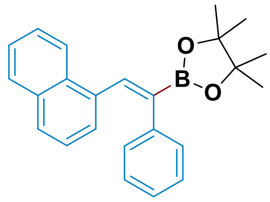
37

White solid, 49% yield; ^{1}**H NMR** (500 MHz, $CDCl_3$) δ 8.17 (d, *J* = 8.1 Hz, 1H), 8.00 (s, 1H), 7.84 (d, *J* = 7.0 Hz, 1H), 7.68 (d, *J* = 8.2 Hz, 1H), 7.61 – 7.45 (m, 2H), 7.21 – 7.12 (m, 1H), 7.15 – 7.06 (m, 5H), 7.03 (d, *J* = 7.2 Hz, 1H), 1.40 (s, 12H). ^{13}**C NMR** (126 MHz, $CDCl_3$) δ 142.01, 139.99, 135.05, 133.54, 132.07, 129.53, 128.54, 127.89, 127.75, 127.66, 126.23, 126.03, 125.85, 125.29, 124.86, 84.01, 25.00. **GCMS (EI)** m/z calcd for $C_{24}H_{25}BO_2$ [M^+] 356.2, found 356.3. The data are in accordance with those reported in the literature.[15]

(Z)-4,4,5,5-tetramethyl-2-(1-(naphthalen-1-yl)-2-phenylvinyl)-1,3,2-dioxaborolane (37')

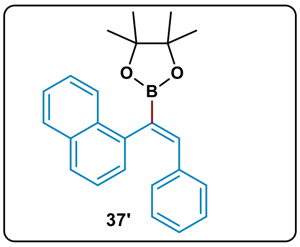

White solid, 37% yield; **^{1}H NMR** (500 MHz, $CDCl_3$) δ 7.87 (t, J = 7.7 Hz, 2H), 7.78 (d, J = 8.2 Hz, 1H), 7.62 (s, 1H), 7.44 (dt, J = 15.4, 7.5 Hz, 2H), 7.37 (t, J = 7.6 Hz, 1H), 7.22 (d, J = 7.0 Hz, 1H), 7.05 (t, J = 7.2 Hz, 1H), 6.99 (t, J = 7.5 Hz, 2H), 6.91 (d, J = 7.7 Hz, 2H), 1.28 (s, 12H). **^{13}C NMR** (126 MHz, $CDCl_3$) δ 144.69, 139.09, 136.86, 133.90, 131.61, 129.99, 128.41, 128.02, 127.93, 126.83, 126.11, 125.84, 125.78, 125.59, 125.38, 83.89, 24.99, 24.93, 24.60. **GCMS (EI)** m/z calcd for $C_{24}H_{25}BO_2$ [M$^+$] 356.2, found 356.3. The data are in accordance with those reported in the literature.[15]

(Z)-4,4,5,5-tetramethyl-2-(1-phenylhex-1-en-2-yl)-1,3,2-dioxaborolane (38)

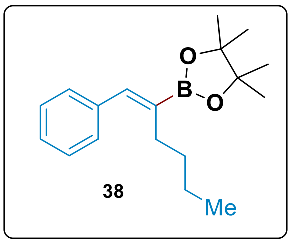

White solid, 78% yield; **^{1}H NMR** (500 MHz, $CDCl_3$) δ 7.33 (d, J = 5.3 Hz, 4H), 7.22 (d, J = 10.1 Hz, 2H), 2.38 (t, J = 7.9 Hz, 2H), 1.47 (p, J = 7.5 Hz, 2H), 1.35 (d, J = 7.3 Hz, 2H), 1.31 (s, 12H), 0.89 (t, J = 7.3 Hz, 3H). **^{13}C NMR** (126 MHz, $CDCl_3$) δ 141.75, 138.14, 129.13, 128.19, 127.09, 83.49, 32.36, 29.33, 24.91, 22.96, 14.18. **GCMS (EI)** m/z calcd for $C_{18}H_{27}BO_2$ [M$^+$] 286.2, found 286.2. The data are in accordance with those reported in the literature.[16]

2-(4-(tert-butyl)phenethyl)-4,4,5,5-tetramethyl-1,3,2-dioxaborolane (39)

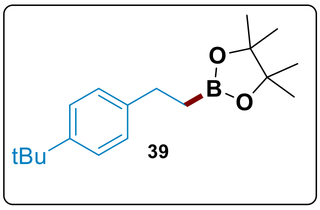

White solid, 78% yield; ^{1}H NMR (500 MHz, $CDCl_3$) δ 7.29 (d, J = 7.9 Hz, 2H), 7.16 (d, J = 7.8 Hz, 2H), 2.72 (t, J = 8.3 Hz, 2H), 1.31 (s, 9H), 1.23 (s, 12H), 1.14 (t, J = 8.3 Hz, 2H). ^{13}C NMR (126 MHz, $CDCl_3$) δ 148.37, 141.49, 127.77, 125.19, 83.20, 34.44, 31.57, 29.50, 24.94. **GCMS (EI)** m/z calcd for $C_{18}H_{29}BO_2$ [M$^+$] 288.2, found 288.3. The data are in accordance with those reported in the literature.[1]

2-(4-fluorophenethyl)-4,4,5,5-tetramethyl-1,3,2-dioxaborolane (40)

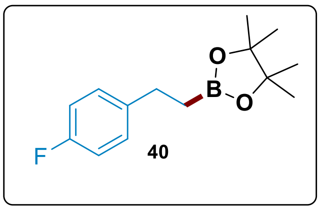

White solid, 82% yield; ^{1}**H NMR** (500 MHz, $CDCl_3$) δ 7.16 (dd, *J* = 8.3, 5.5 Hz, 2H), 6.93 (t, *J* = 8.6 Hz, 2H), 2.71 (t, *J* = 8.1 Hz, 2H), 1.21 (s, 12H), 1.11 (t, *J* = 8.1 Hz, 2H). ^{13}**C NMR** (126 MHz, $CDCl_3$) δ 162.20, 160.27, 140.08 (d, *J* = 3.2 Hz), 129.44 (d, *J* = 7.7 Hz), 114.94 (d, *J* = 21.0 Hz), 83.27, 29.28, 24.92. **GCMS (EI)** m/z calcd for $C_{14}H_{20}BFO_2$ [M^+] 250.1, found 250.1. The data are in accordance with those reported in the literature.[17]

2-(2-([1,1'-biphenyl]-4-yl)ethyl)-4,4,5,5-tetramethyl-1,3,2-dioxaborolane (41)

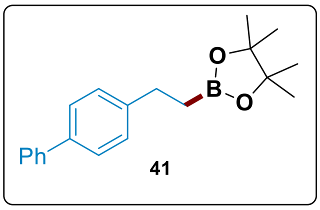

White solid, 80% yield; ^{1}H NMR (500 MHz, $CDCl_3$) δ 7.61 (d, *J* = 8.2 Hz, 2H), 7.54 (d, *J* = 8.1 Hz, 2H), 7.45 (t, *J* = 7.6 Hz, 2H), 7.34 (t, *J* = 9.2 Hz, 3H), 2.83 (t, *J* = 8.2 Hz, 2H), 1.26 (s, 12H), 1.22 (t, *J* = 8.2 Hz, 2H). ^{13}C NMR (126 MHz, $CDCl_3$) δ 143.69, 141.35, 138.58, 128.79, 128.55, 127.08, 127.05, 127.01, 83.24, 29.71, 24.93. **GCMS (EI)** m/z calcd for $C_{20}H_{25}BO_2$ [M^+] 308.2, found 308.3. The data are in accordance with those reported in the literature.[1]

2-(3-chlorophenethyl)-4,4,5,5-tetramethyl-1,3,2-dioxaborolane (42)

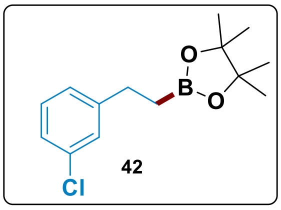

White solid, 79% yield**;** ^{1}**H NMR** (500 MHz, $CDCl_3$) δ 7.22 (d, *J* = 1.8 Hz, 1H), 7.17 (t, *J* = 7.8 Hz, 1H), 7.15 – 7.10 (m, 1H), 7.09 (t, *J* = 9.5 Hz, 1H), 2.72 (t, *J* = 8.1 Hz, 2H), 1.22 (s, 12H), 1.12 (t, *J* = 8.0 Hz, 2H). ^{13}**C NMR** (126 MHz, $CDCl_3$) δ 146.56, 134.00, 129.56, 128.41, 126.38, 125.80, 83.34, 29.82, 24.93. **GCMS (EI)** m/z calcd for $C_{14}H_{20}BClO_2$ [M^+] 266.1, found 266.1. The data are in accordance with those reported in the literature.[1, 18].

4,4,5,5-tetramethyl-2-(3-(trifluoromethyl)phenethyl)-1,3,2-dioxaborolane (43)

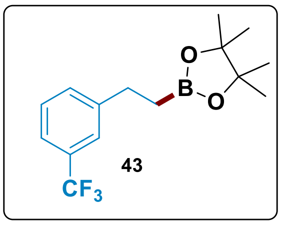

White solid, 77% yield; **^{1}H NMR** (500 MHz, $CDCl_3$) δ 7.49 (s, 1H), 7.38 (dq, *J* = 15.0, 7.8 Hz, 3H), 2.80 (t, *J* = 7.9 Hz, 2H), 1.21 (s, 12H), 1.15 (t, *J* = 8.0 Hz, 2H). ^{13}C NMR (126 MHz, $CDCl_3$) δ 145.35, 131.67, 130.54 (q, *J* = 31.9 Hz), 128.70, 128.46, 127.74, 125.58, 125.03 (q, *J* = 3.8 Hz), 123.41, 122.56 (d, *J* = 3.8 Hz), 121.25, 83.39, 29.94, 24.91. **GCMS (EI)** m/z calcd for $C_{15}H_{20}BF_3O_2$ [M^+] 300.1, found 300.1. The data are in accordance with those reported in the literature.[1, 18].

4,4,5,5-tetramethyl-2-(2-(naphthalen-2-yl)ethyl)-1,3,2-dioxaborolane (44)

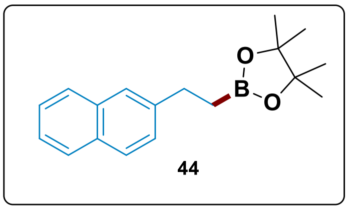

White solid, 75% yield; **^{1}H NMR** (500 MHz, $CDCl_3$) δ 7.82 – 7.73 (m, 3H), 7.66 (s, 1H), 7.41 (dq, *J* = 15.4, 8.3 Hz, 3H), 2.93 (t, *J* = 8.2 Hz, 2H), 1.28 – 1.21 (m, 15H). **^{13}C NMR** (126 MHz, $CDCl_3$) δ 142.10, 133.78, 132.04, 127.82, 127.69, 127.56, 127.41, 125.85, 125.82, 125.04, 83.28, 30.26, 24.96. **GCMS (EI)** m/z calcd for $C_{18}H_{23}BO_2$ [M^+] 282.2, found 282.2. The data are in accordance with those reported in the literature.[1, 18].

2-(2,2-diphenylethyl)-4,4,5,5-tetramethyl-1,3,2-dioxaborolane (45)

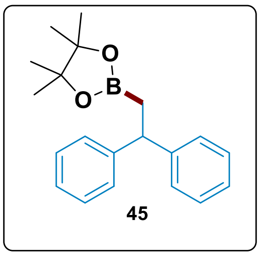

White solid, 88% yield; **^{1}H NMR** (500 MHz, $CDCl_3$) δ 7.26 (s, 8H), 7.14 (t, *J* = 7.0 Hz, 2H), 4.29 (t, *J* = 8.5 Hz, 1H), 1.60 (d, *J* = 8.5 Hz, 2H), 1.06 (s, 12H). **^{13}C NMR** (126 MHz, $CDCl_3$) δ 146.72, 128.36, 127.81, 126.03, 83.26, 46.66, 24.71. **GCMS (EI)** m/z calcd for $C_{20}H_{25}BO_2$ [M^+] 308.2, found 308.2. The data are in accordance with those reported in the literature.[1].

20230625-20-Sagadevan.Arunachalam_169829-SFA-210.10.fid
SFA-210

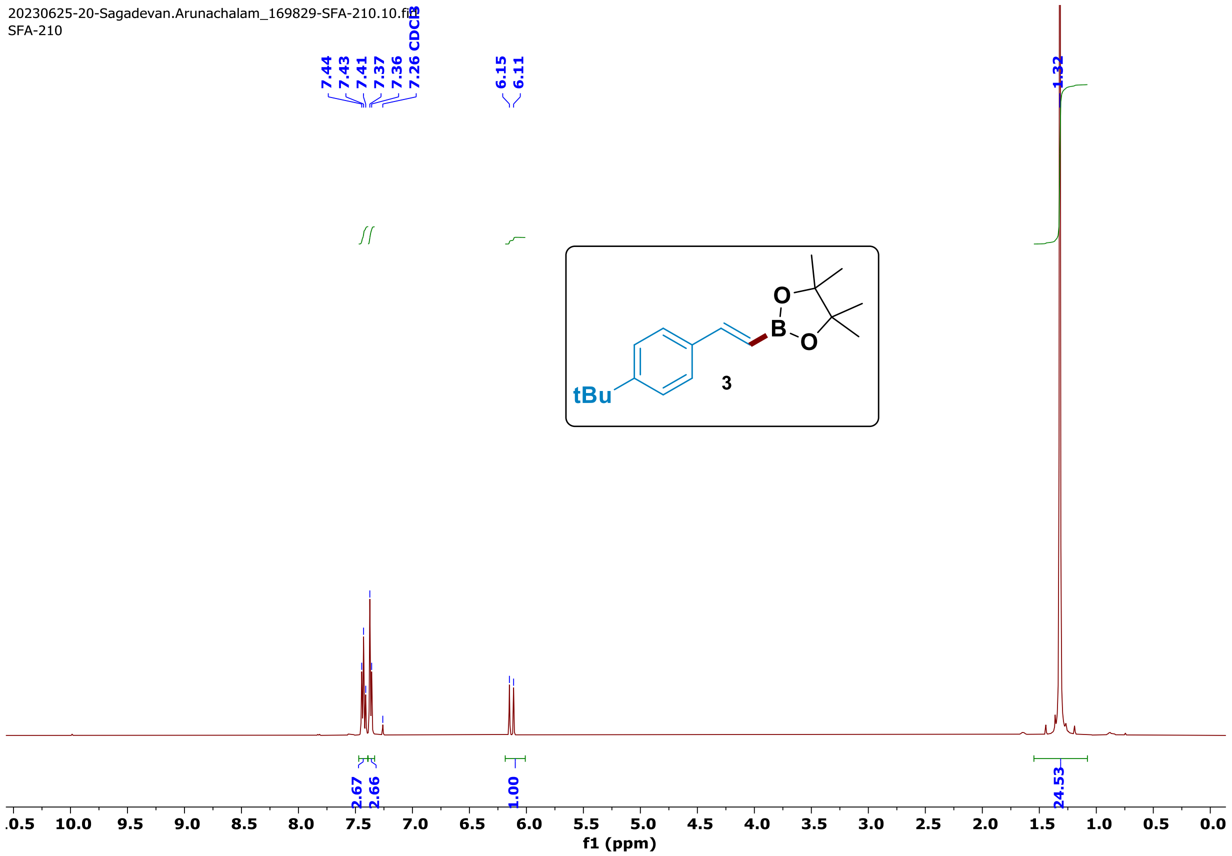

20230626-58-Sagadevan.Arunachalam_169829SFA-210.10.fid
SFA-210

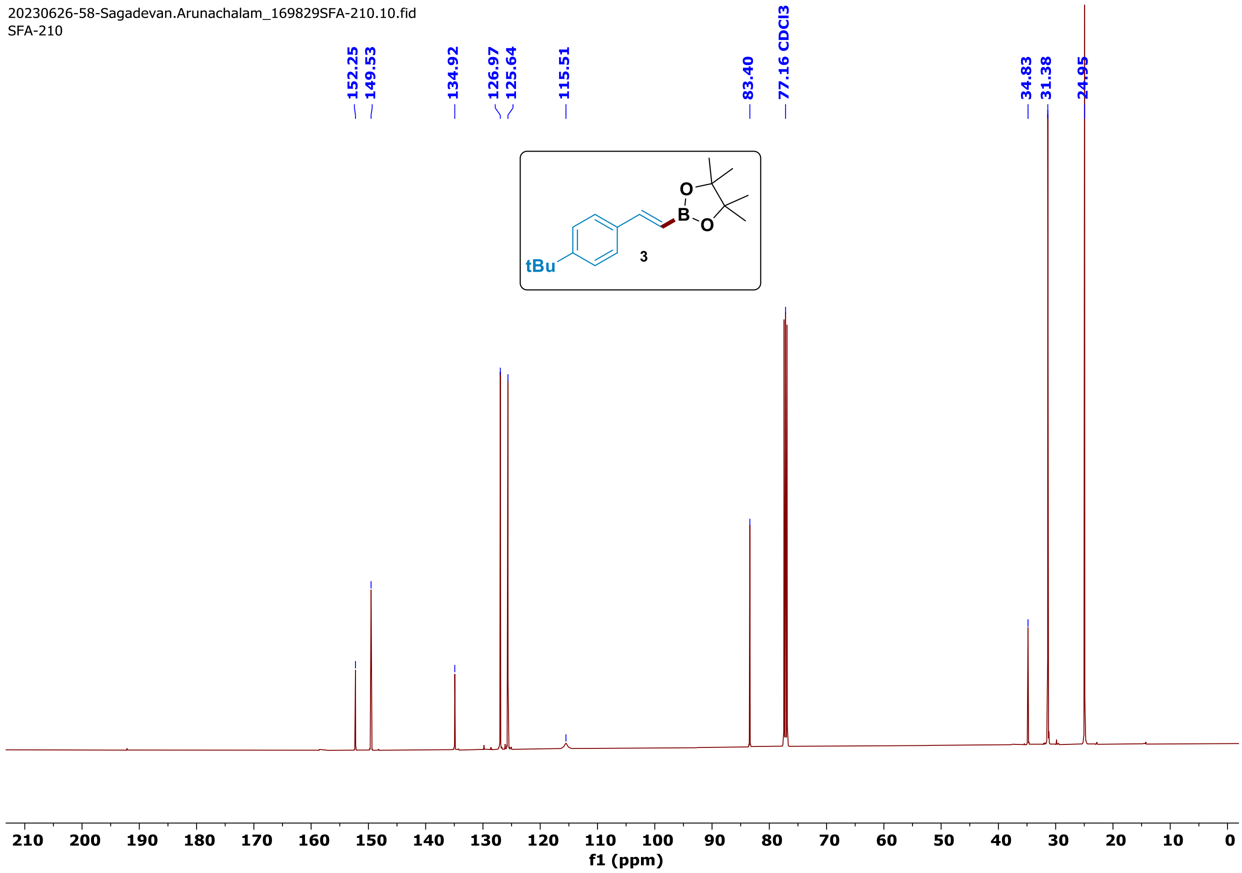

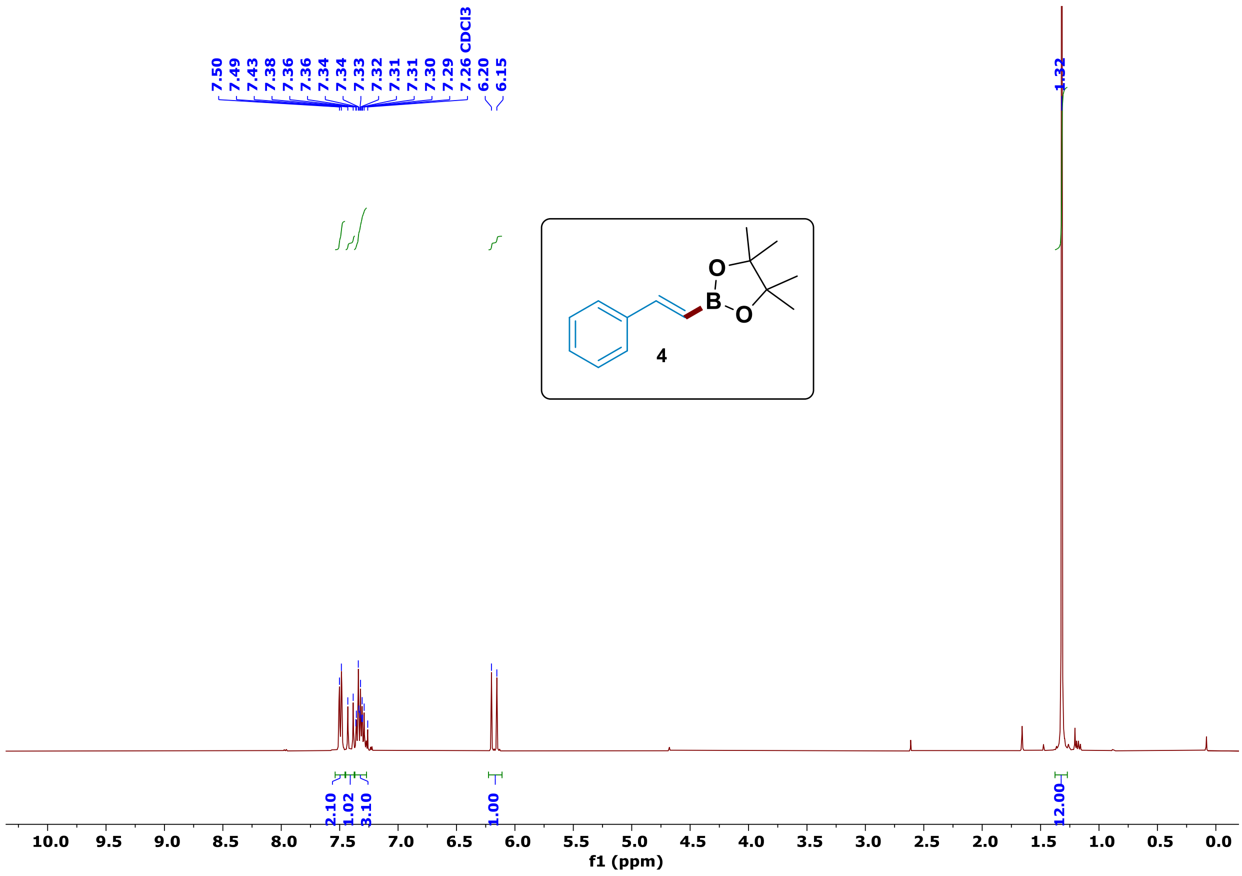
7.50
7.49
7.43
7.38
7.36
7.36
7.34
7.34
7.33
7.32
7.31
7.31
7.30
7.29
7.26 CDCl3
6.20
6.15
1.32
O
B
O
4
2.10
1.02
3.10
1.00
12.00
10.0 9.5 9.0 8.5 8.0 7.5 7.0 6.5 6.0 5.5 5.0 4.5 4.0 3.5 3.0 2.5 2.0 1.5 1.0 0.5 0.0
f1 (ppm)

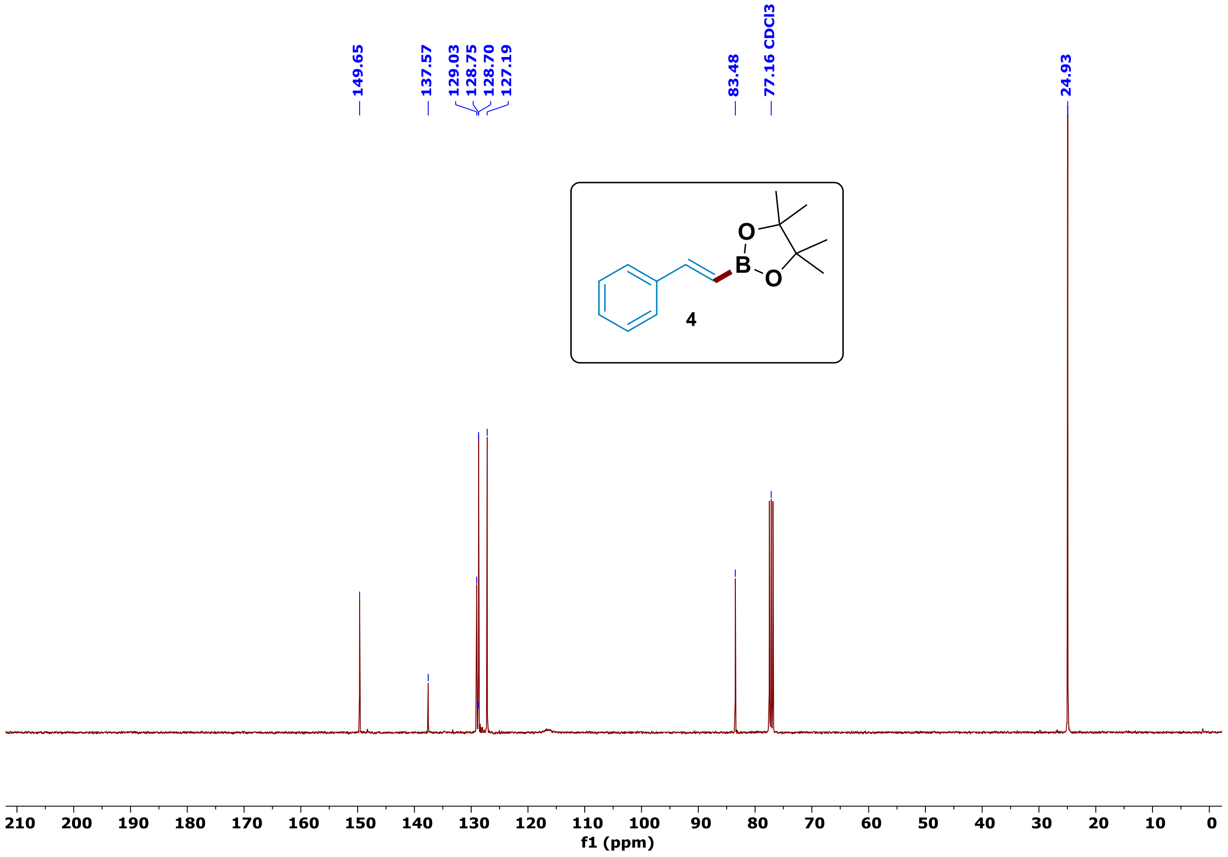
149.65
137.57
129.03
128.75
128.70
127.19
83.48
77.16 CDCl3
24.93
O
B
O
4
210 200 190 180 170 160 150 140 130 120 110 100 90 80 70 60 50 40 30 20 10 0
f1 (ppm)

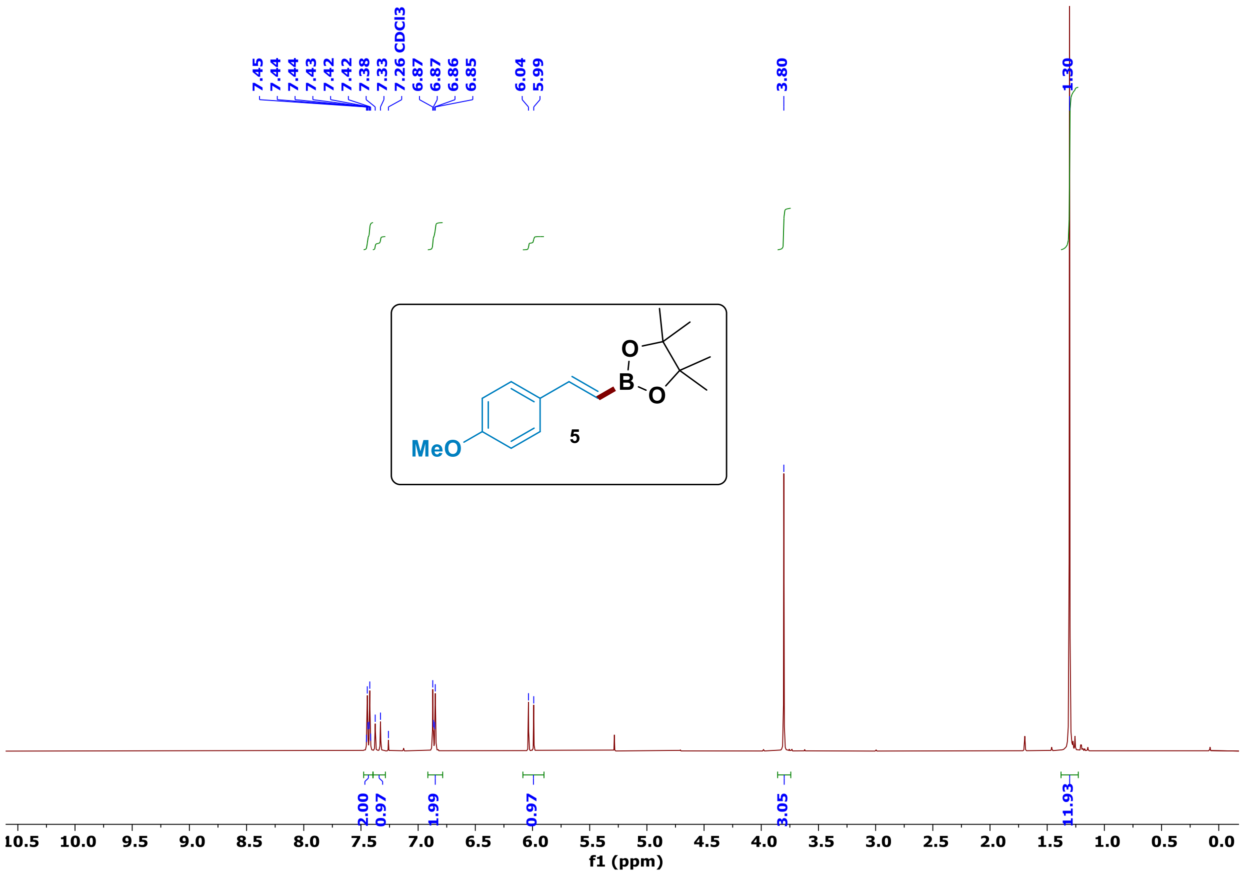
7.45 7.44 7.44 7.43 7.42 7.42 7.38 7.33 7.26 CDCl3 6.87 6.87 6.86 6.85 6.04 5.99 3.80 1.30
MeO
B
O
O
5
2.00 0.97 1.99 0.97 3.05 11.93
10.5 10.0 9.5 9.0 8.5 8.0 7.5 7.0 6.5 6.0 5.5 5.0 4.5 4.0 3.5 3.0 2.5 2.0 1.5 1.0 0.5 0.0
f1 (ppm)

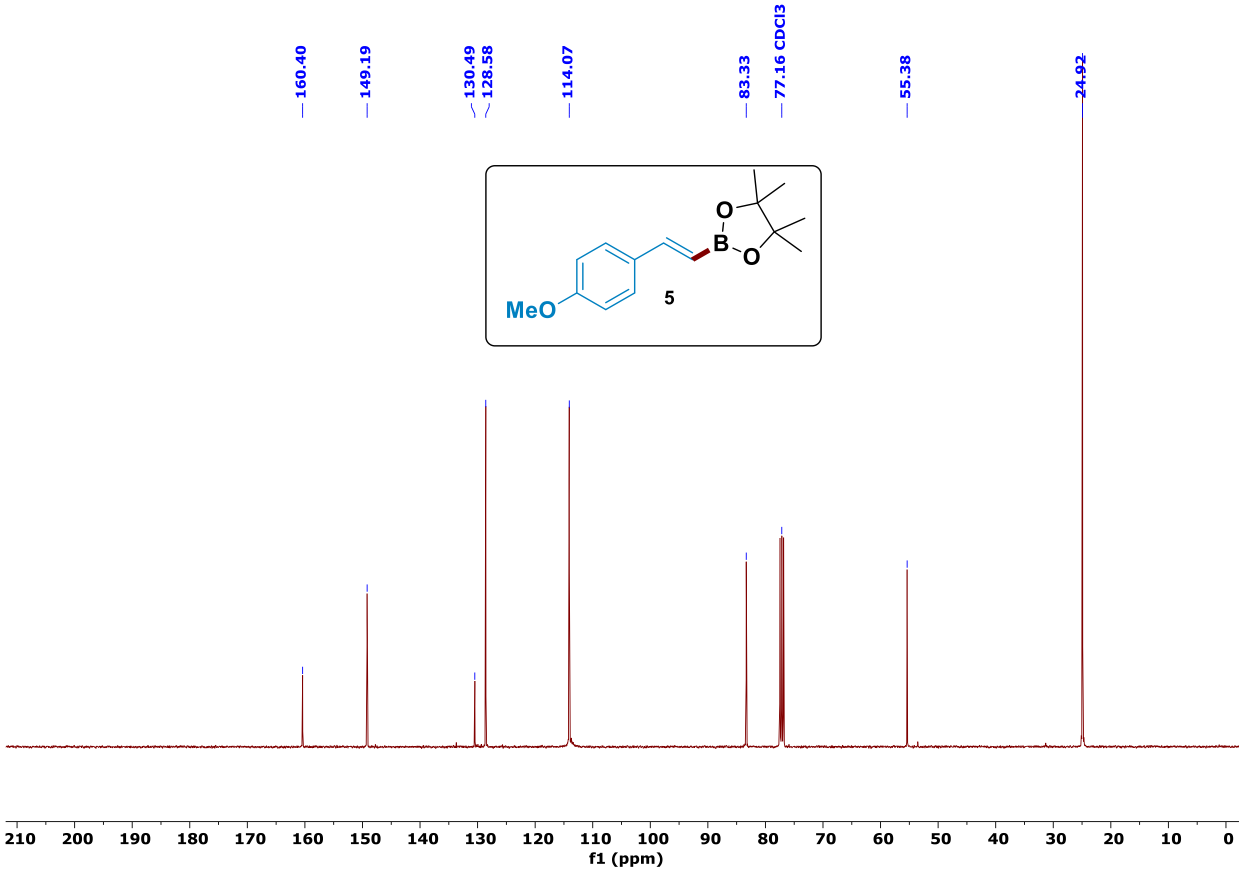
160.40 149.19 130.49 128.58 114.07 83.33 77.16 CDCl3 55.38 24.92
MeO
B
O
O
5
210 200 190 180 170 160 150 140 130 120 110 100 90 80 70 60 50 40 30 20 10 0
f1 (ppm)

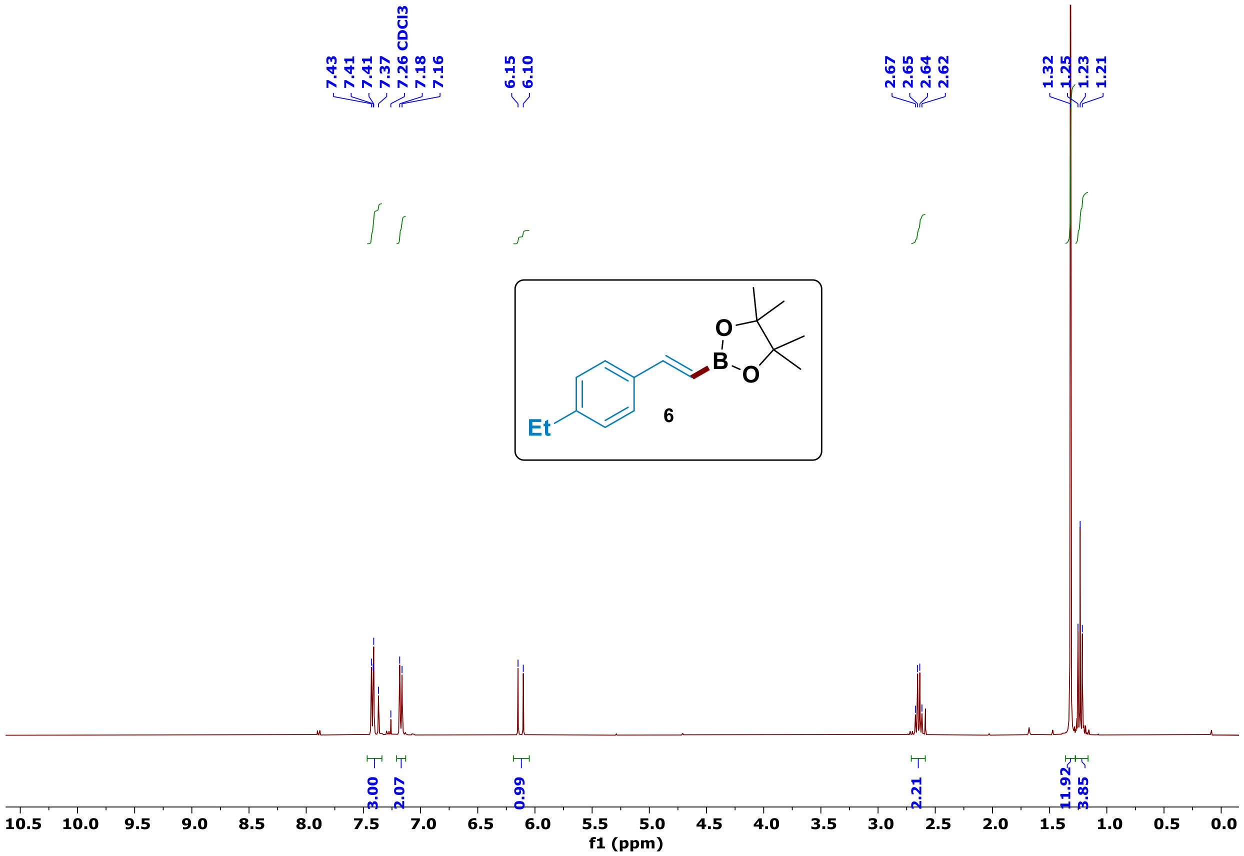
7.43
7.41
7.41
7.37
7.26 CDCl3
7.18
7.16
6.15
6.10
2.67
2.65
2.64
2.62
1.32
1.25
1.23
1.21
Et
O
B
O
6
3.00
2.07
0.99
2.21
11.92
3.85
f1 (ppm)

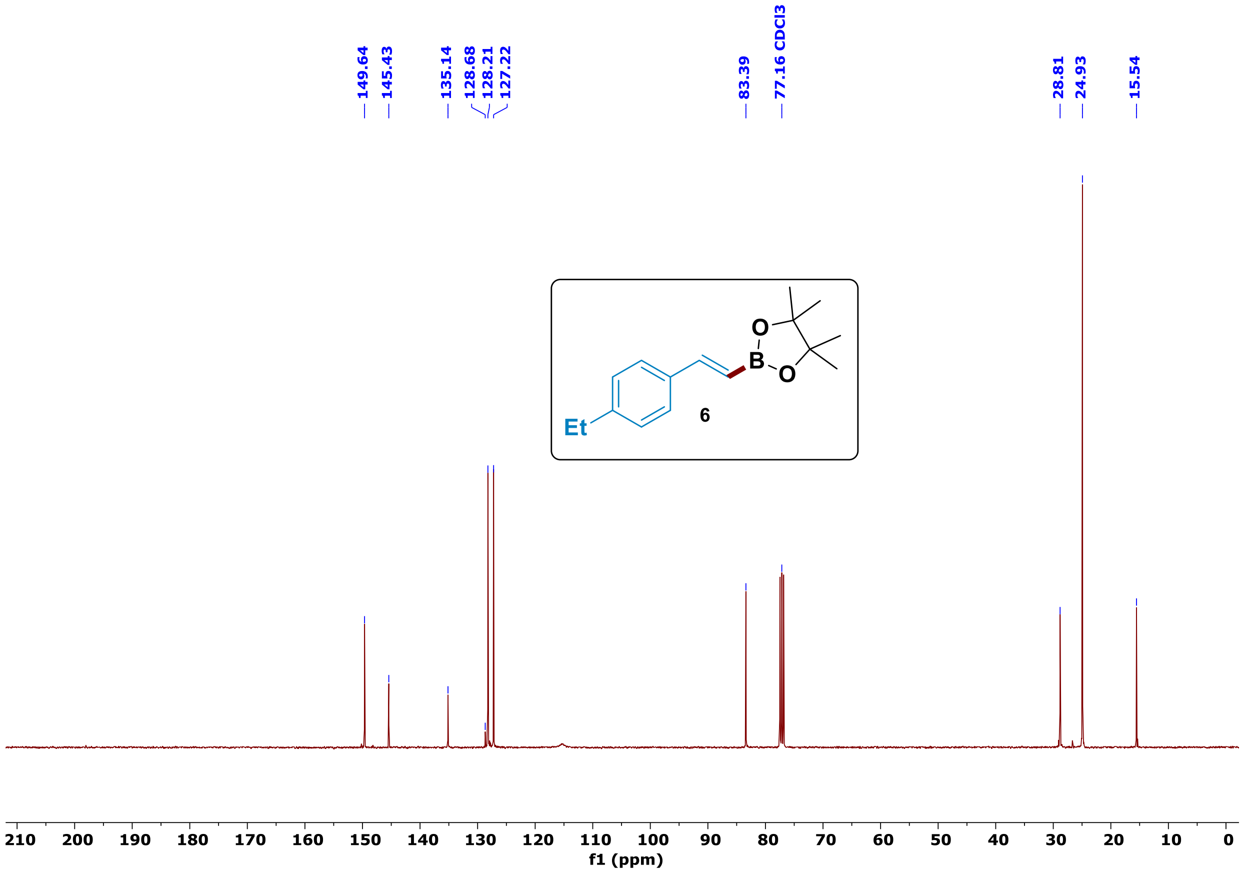
149.64
145.43
135.14
128.68
128.21
127.22
83.39
77.16 CDCl3
28.81
24.93
15.54
Et
O
B
O
6
f1 (ppm)

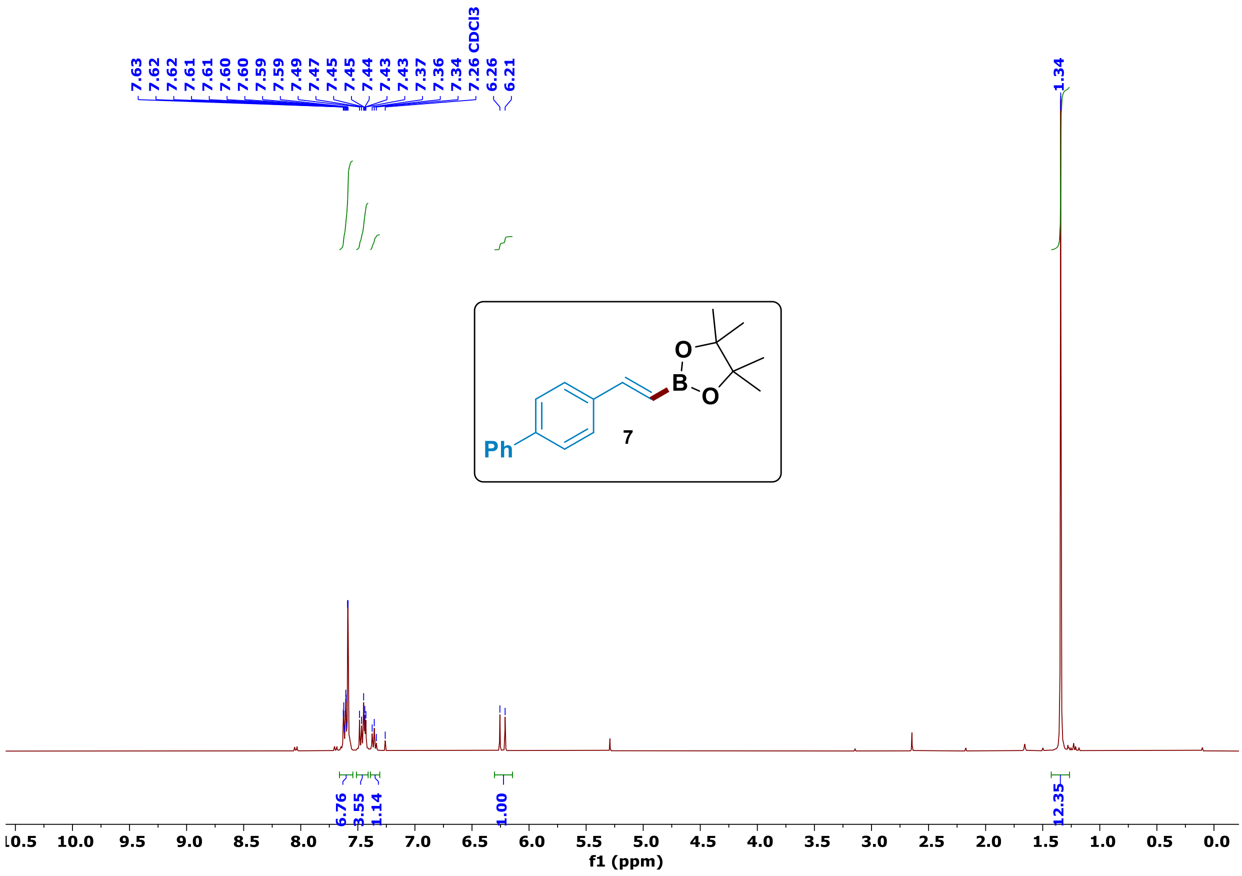
7.63
7.62
7.61
7.61
7.60
7.59
7.59
7.49
7.47
7.45
7.45
7.44
7.43
7.43
7.37
7.36
7.34
7.26 CDCl3
6.26
6.21
1.34
O
B
O
Ph
7
6.76
3.55
1.14
1.00
12.35
f1 (ppm)

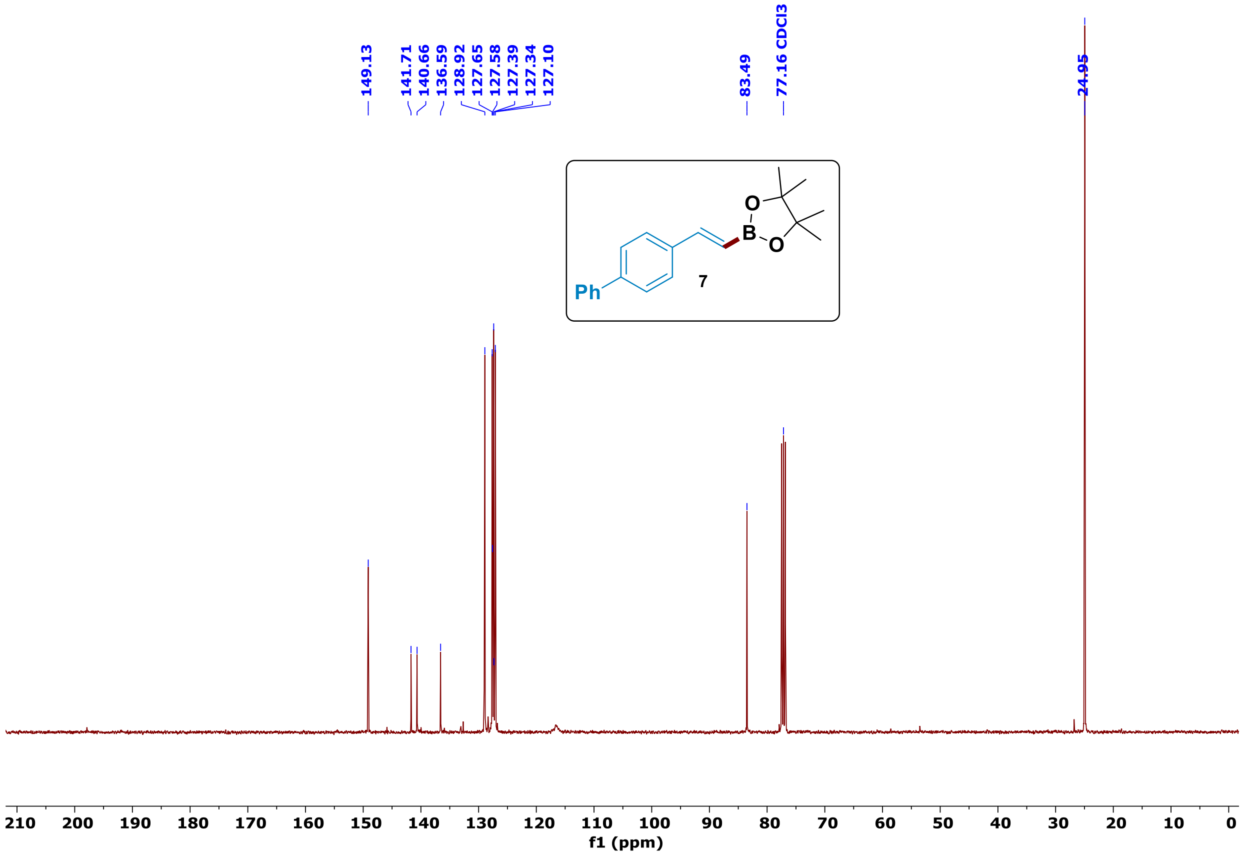
149.13
141.71
140.66
136.59
128.92
127.65
127.58
127.39
127.34
127.10
83.49
77.16 CDCl3
24.95
O
B
O
Ph
7
f1 (ppm)

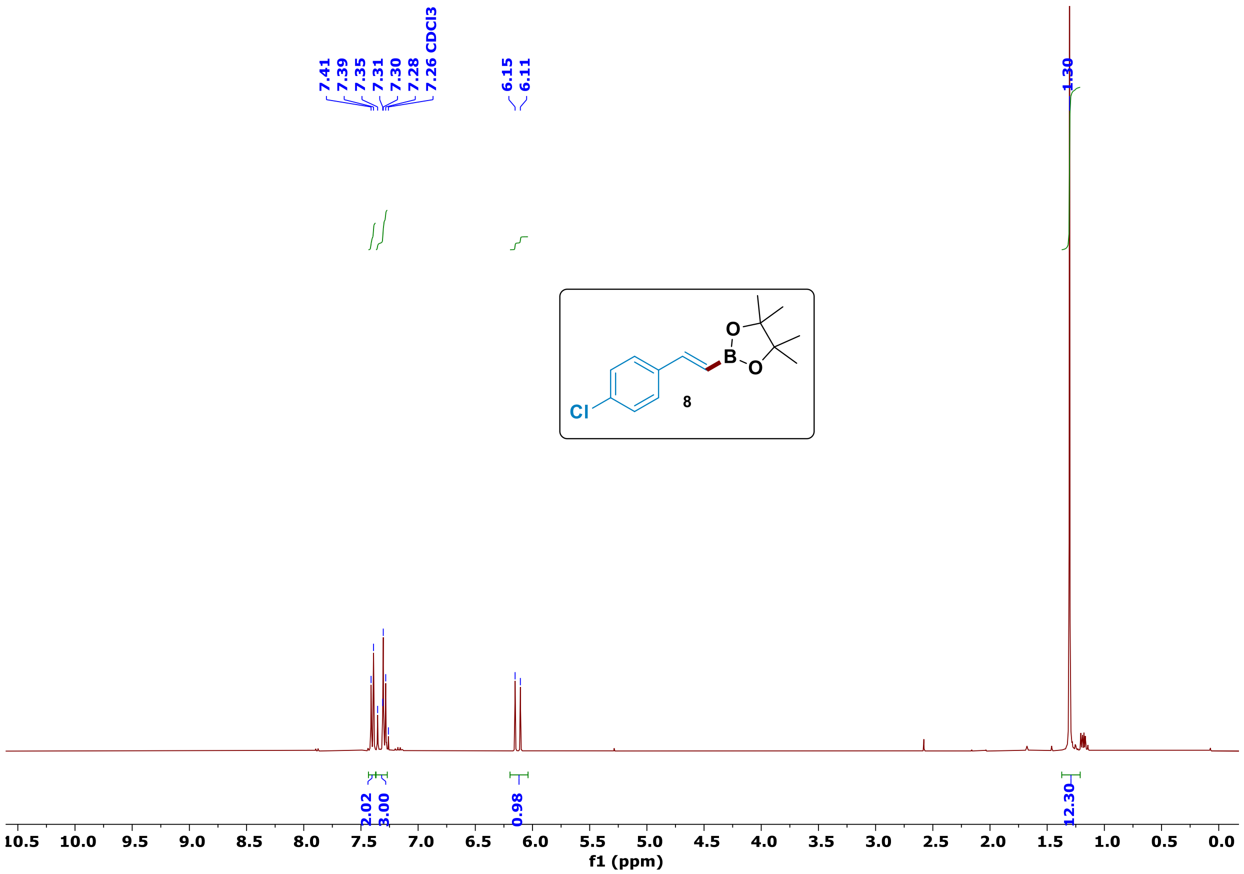
7.41
7.39
7.35
7.31
7.30
7.28
7.26 CDCl3
6.15
6.11
1.30
Cl
O
B
O
8
2.02
3.00
0.98
12.30
f1 (ppm)

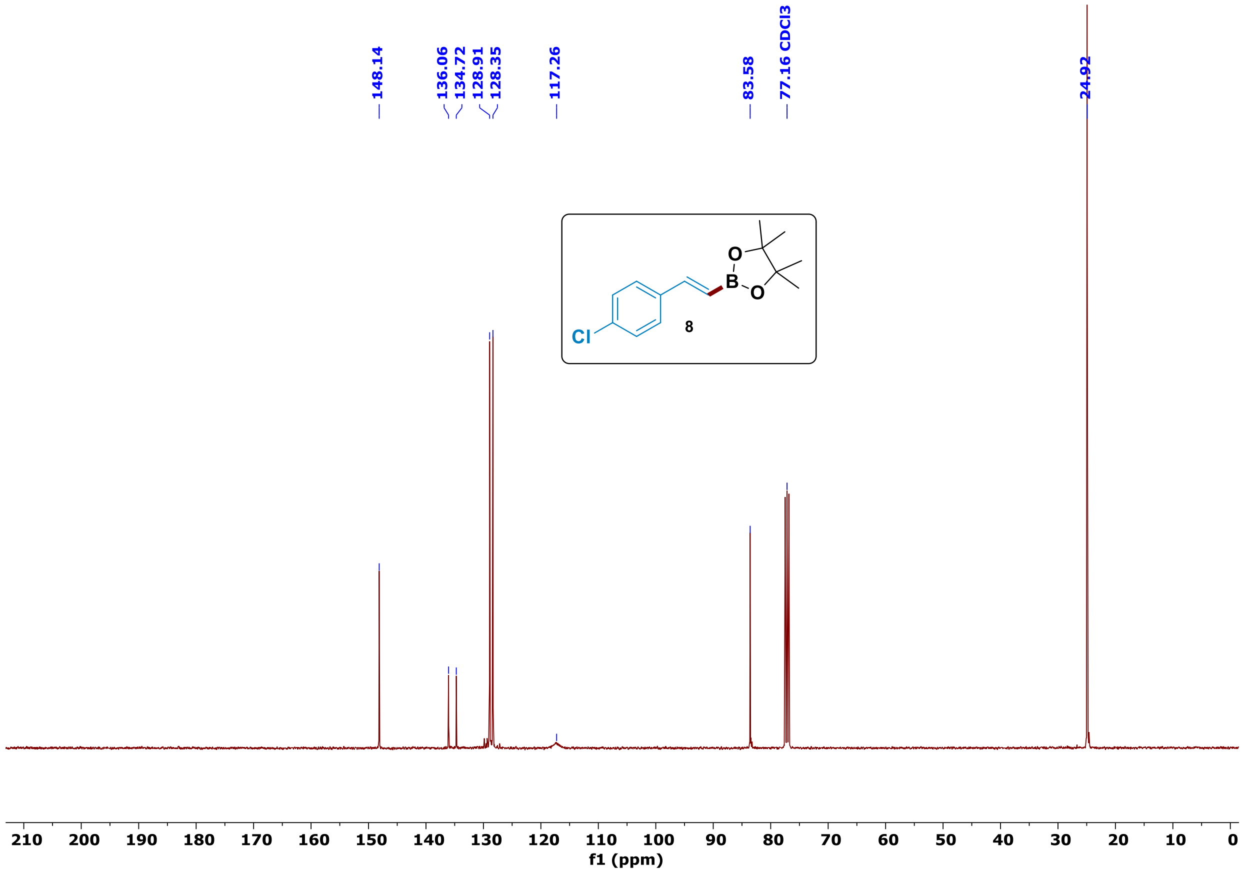
148.14
136.06
134.72
128.91
128.35
117.26
83.58
77.16 CDCl3
24.92
Cl
O
B
O
8
f1 (ppm)

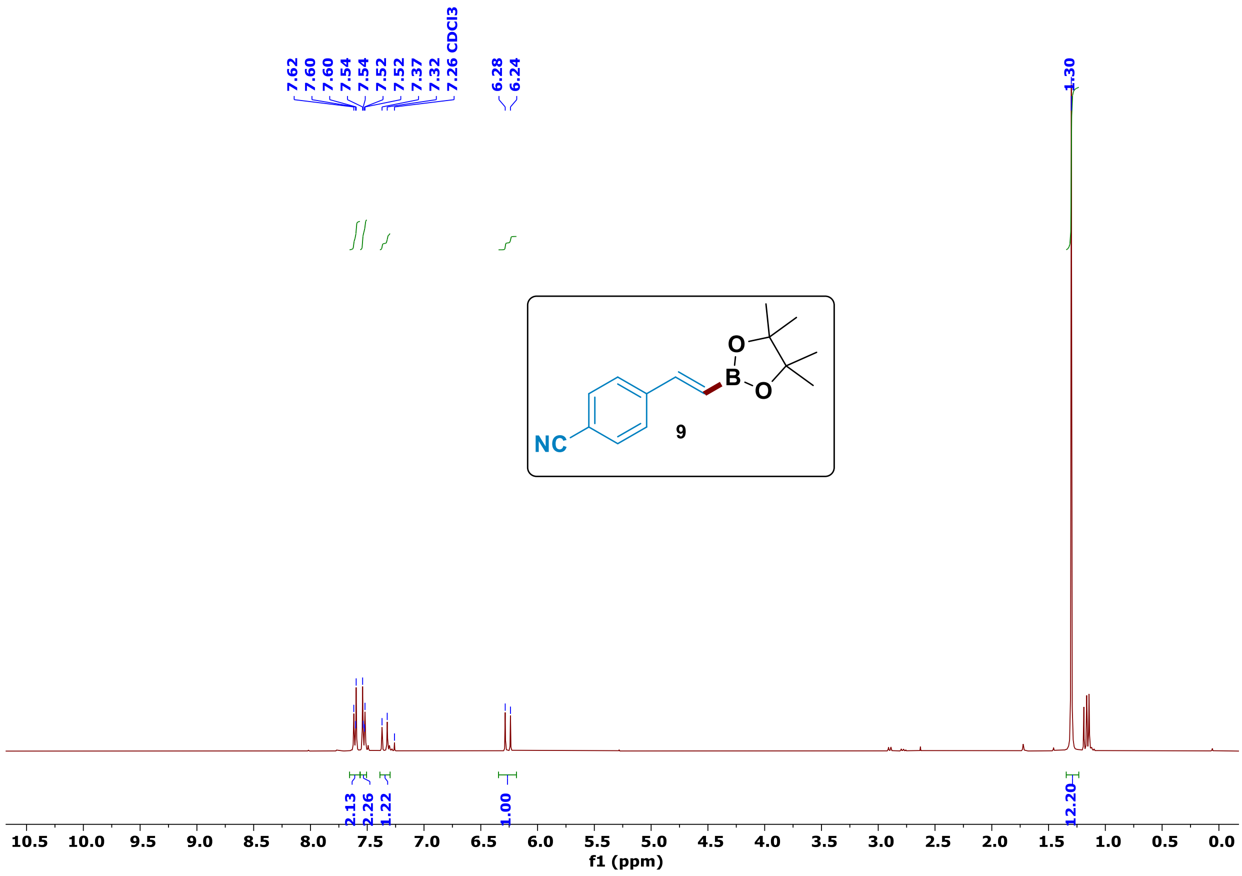
7.62
7.60
7.60
7.54
7.54
7.52
7.52
7.37
7.32
7.26 CDCl3
6.28
6.24
1.30
NC
9
O
B
O
2.13
2.26
1.22
1.00
12.20
10.5 10.0 9.5 9.0 8.5 8.0 7.5 7.0 6.5 6.0 5.5 5.0 4.5 4.0 3.5 3.0 2.5 2.0 1.5 1.0 0.5 0.0
f1 (ppm)

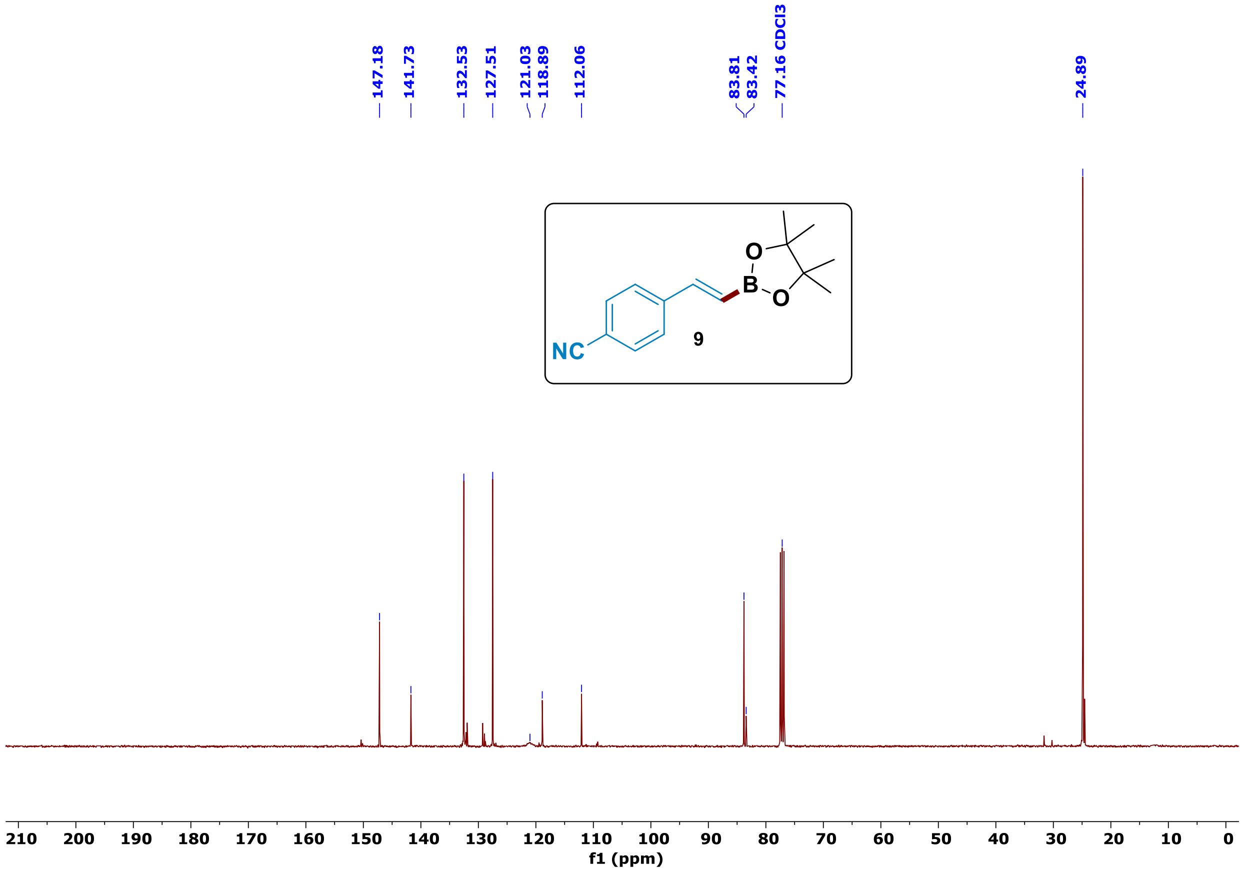
147.18
141.73
132.53
127.51
121.03
118.89
112.06
83.81
83.42
77.16 CDCl3
24.89
NC
9
O
B
O
210 200 190 180 170 160 150 140 130 120 110 100 90 80 70 60 50 40 30 20 10 0
f1 (ppm)

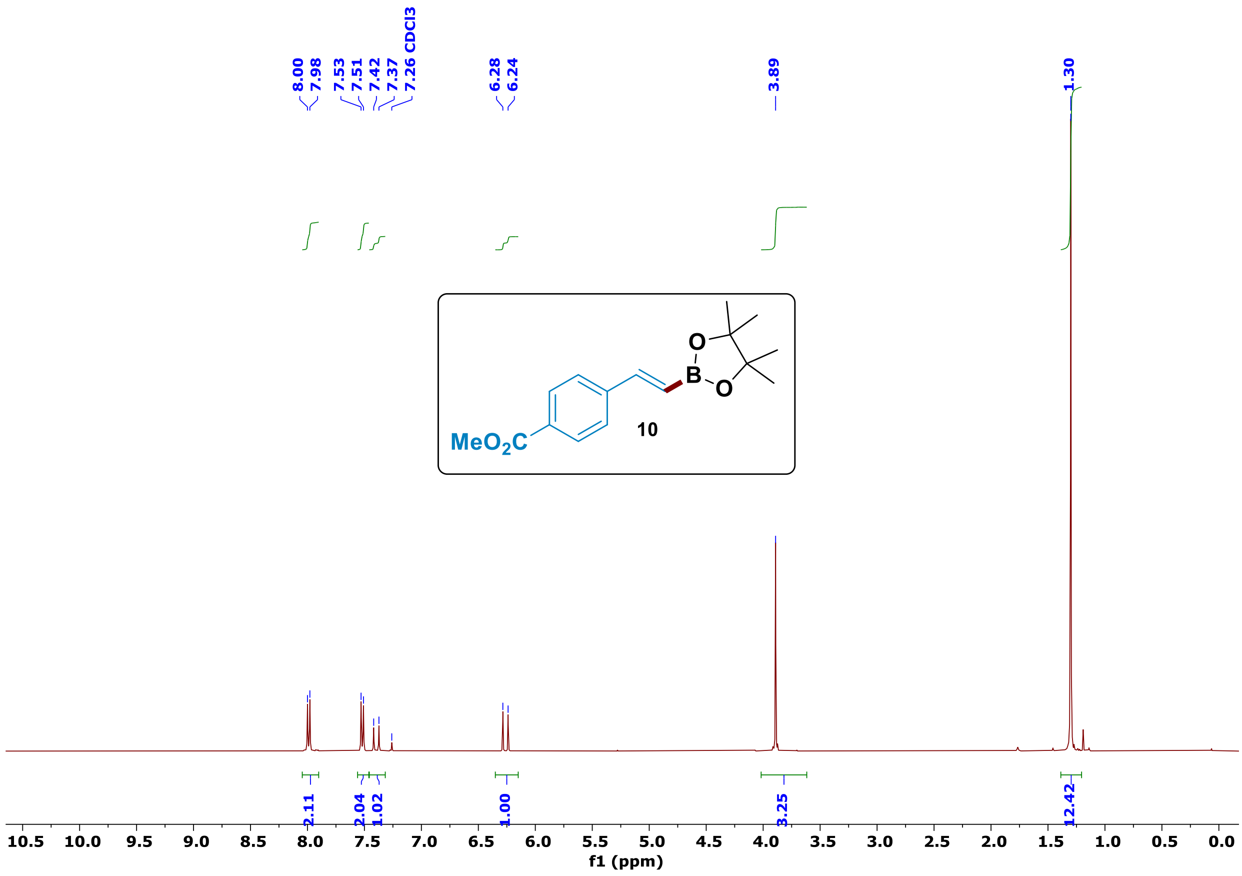
8.00
7.98
7.53
7.51
7.42
7.37
7.26 CDCl3
6.28
6.24
3.89
1.30
MeO2C
O
B
O
10
2.11
2.04
1.02
1.00
3.25
12.42
10.5 10.0 9.5 9.0 8.5 8.0 7.5 7.0 6.5 6.0 5.5 5.0 4.5 4.0 3.5 3.0 2.5 2.0 1.5 1.0 0.5 0.0
f1 (ppm)

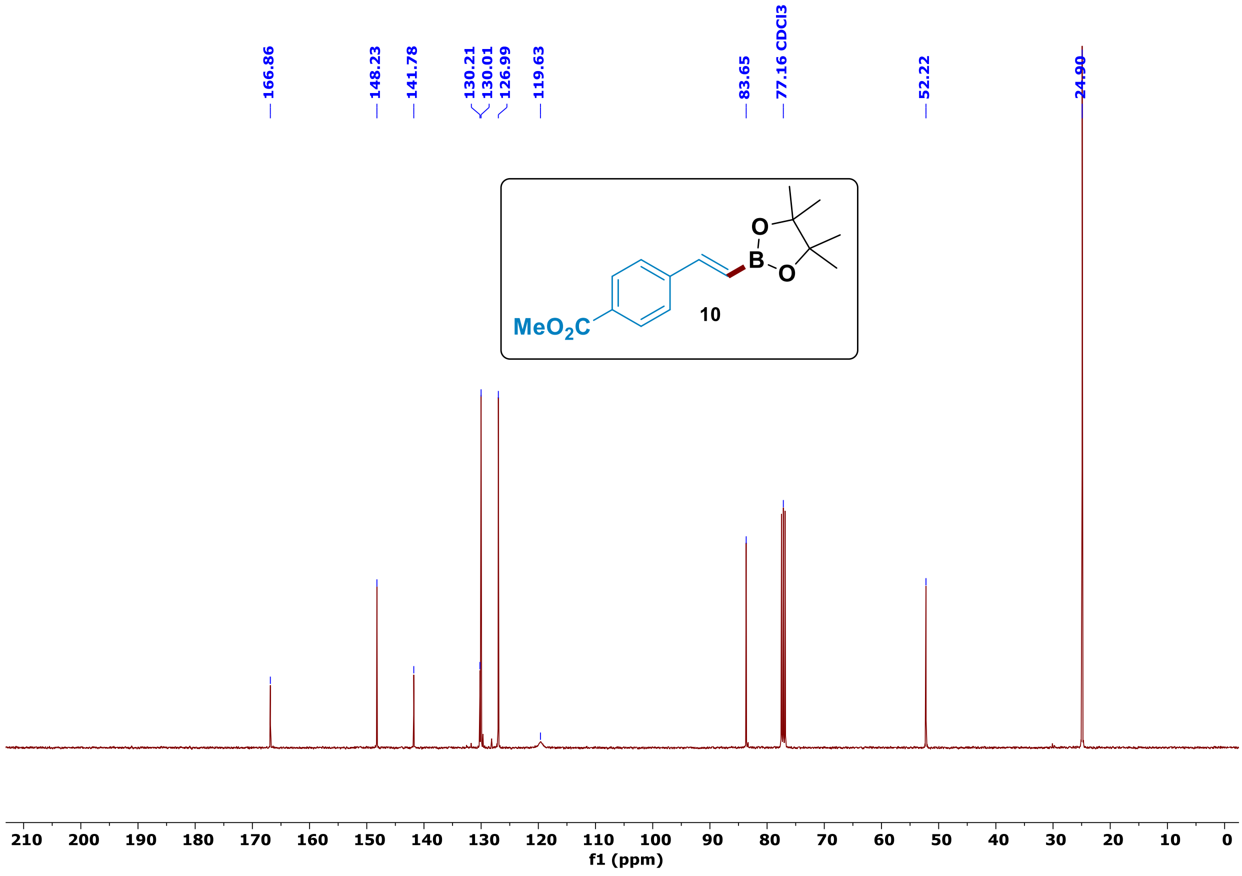
166.86
148.23
141.78
130.21
130.01
126.99
119.63
83.65
77.16 CDCl3
52.22
24.90
MeO2C
O
B
O
10
210 200 190 180 170 160 150 140 130 120 110 100 90 80 70 60 50 40 30 20 10 0
f1 (ppm)

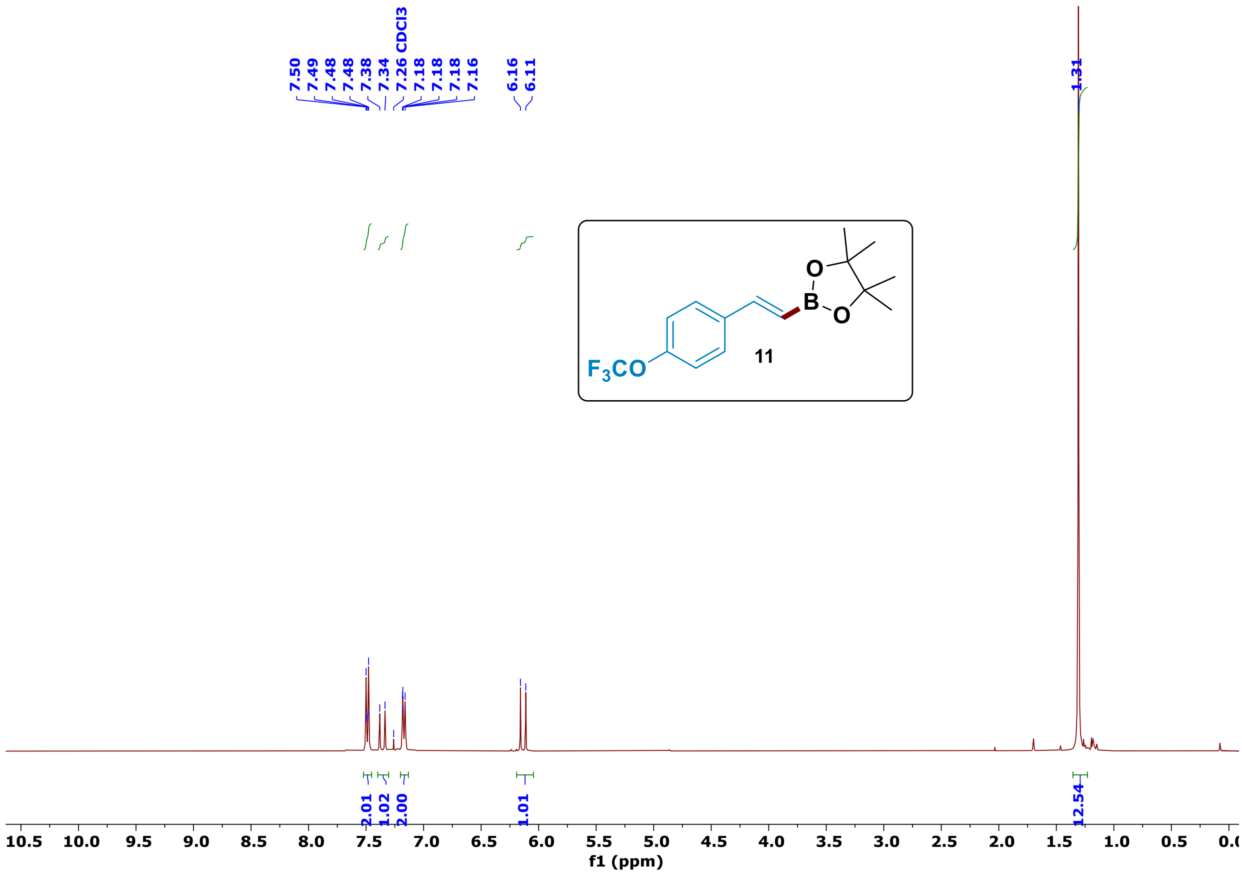
7.50
7.49
7.48
7.48
7.38
7.34
7.26 CDCl3
7.18
7.18
7.18
7.16
6.16
6.11
1.31
F3CO
O
B
O
11
2.01
1.02
2.00
1.01
12.54
f1 (ppm)

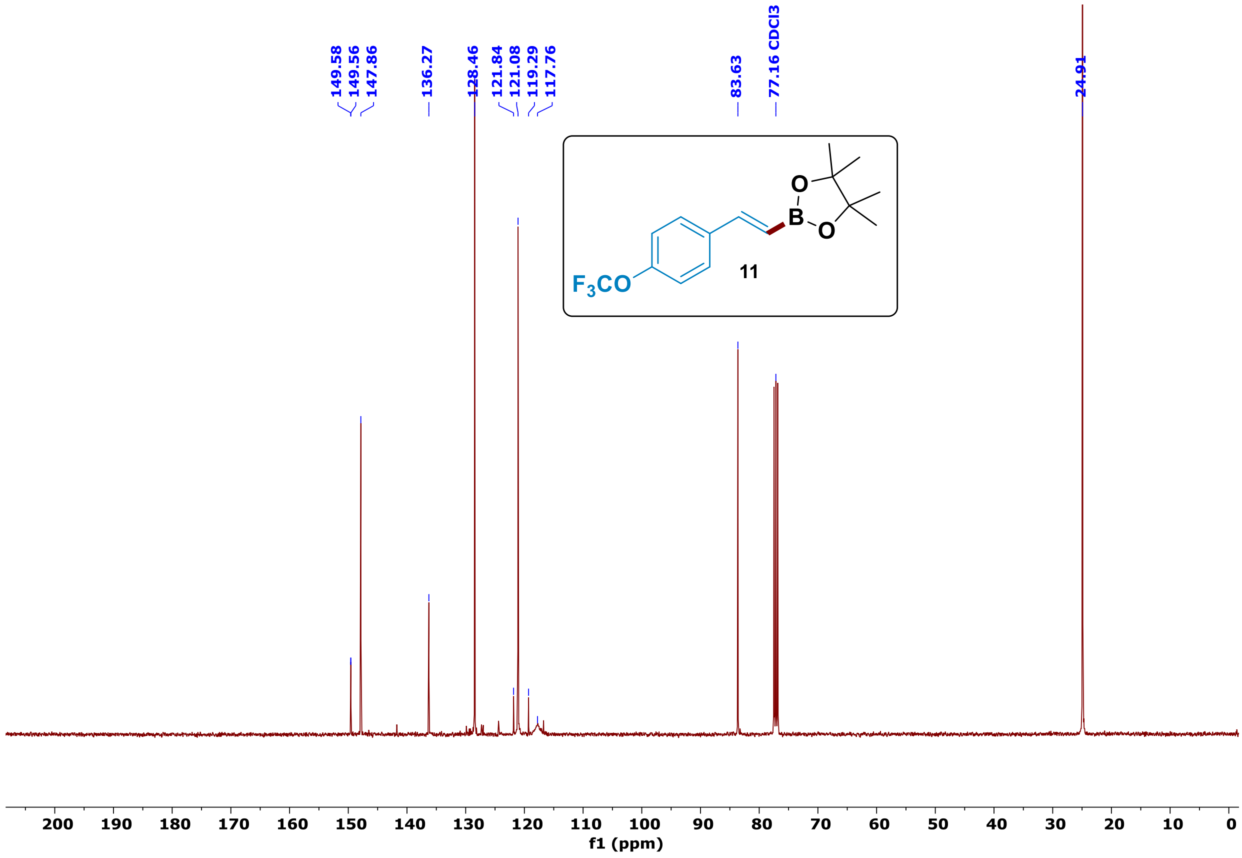
149.58
149.56
147.86
136.27
128.46
121.84
121.08
119.29
117.76
83.63
77.16 CDCl3
24.91
F3CO
O
B
O
11
f1 (ppm)

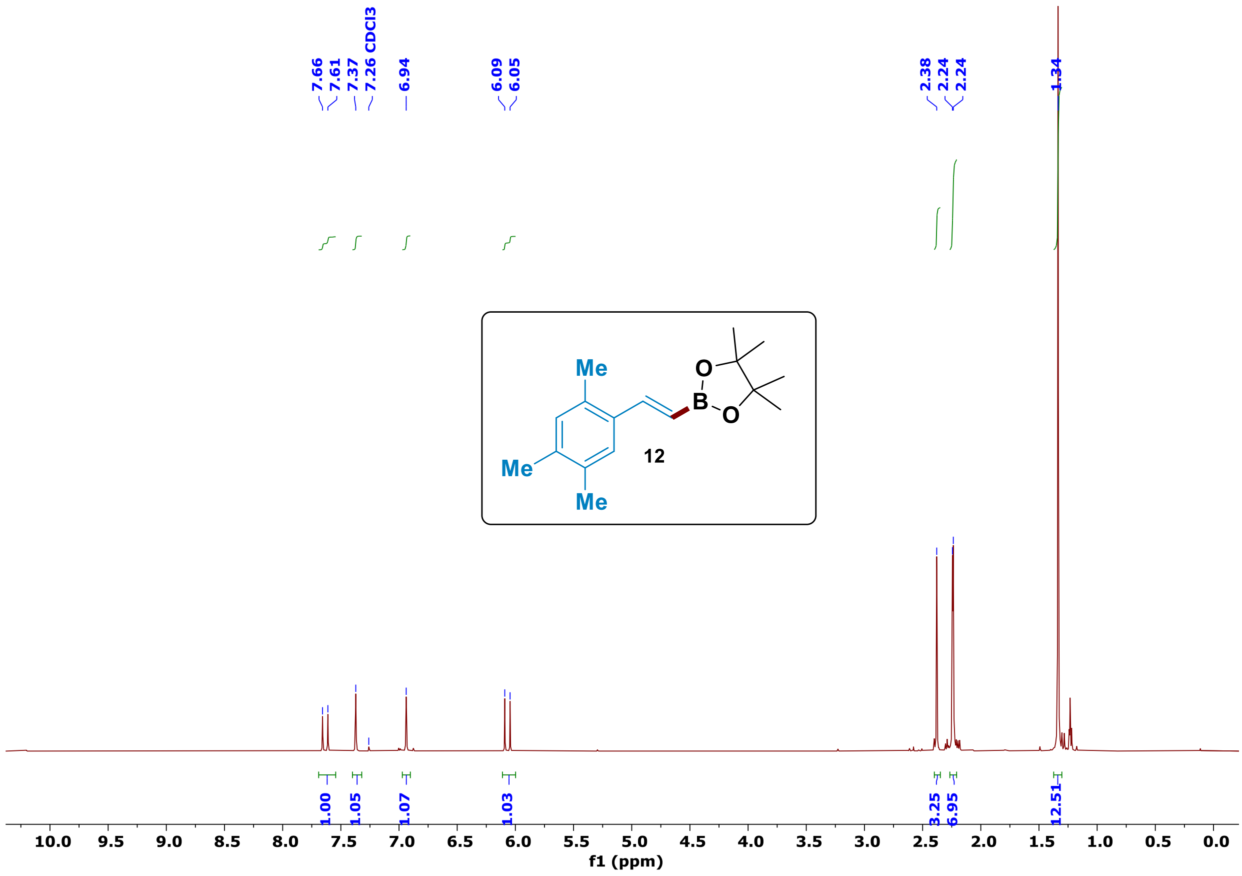
7.66
7.61
7.37
7.26 CDCl3
6.94
6.09
6.05
2.38
2.24
2.24
1.34
Me
Me
Me
O
B
O
12
1.00
1.05
1.07
1.03
3.25
6.95
12.51
10.0 9.5 9.0 8.5 8.0 7.5 7.0 6.5 6.0 5.5 5.0 4.5 4.0 3.5 3.0 2.5 2.0 1.5 1.0 0.5 0.0
f1 (ppm)

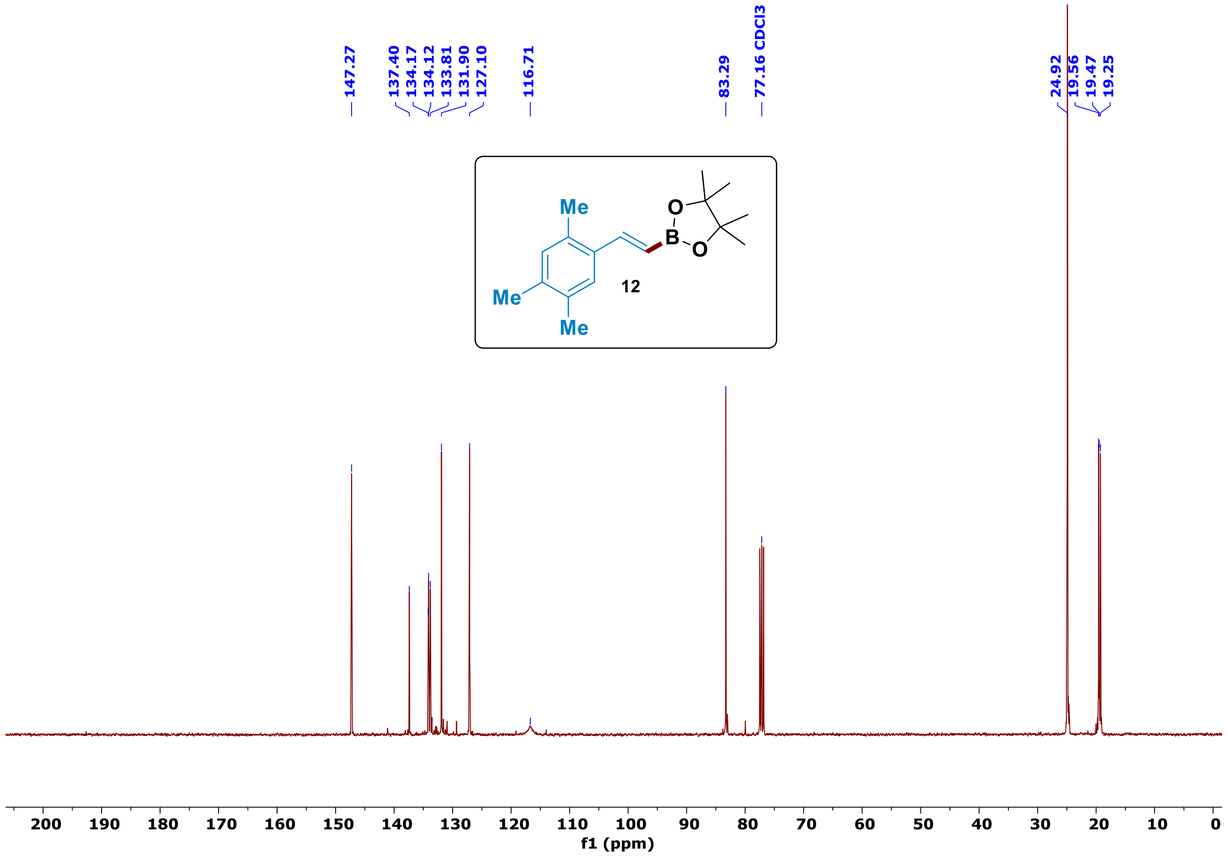
147.27
137.40
134.17
134.12
133.81
131.90
127.10
116.71
83.29
77.16 CDCl3
24.92
19.56
19.47
19.25
Me
Me
Me
O
B
O
12
200 190 180 170 160 150 140 130 120 110 100 90 80 70 60 50 40 30 20 10 0
f1 (ppm)

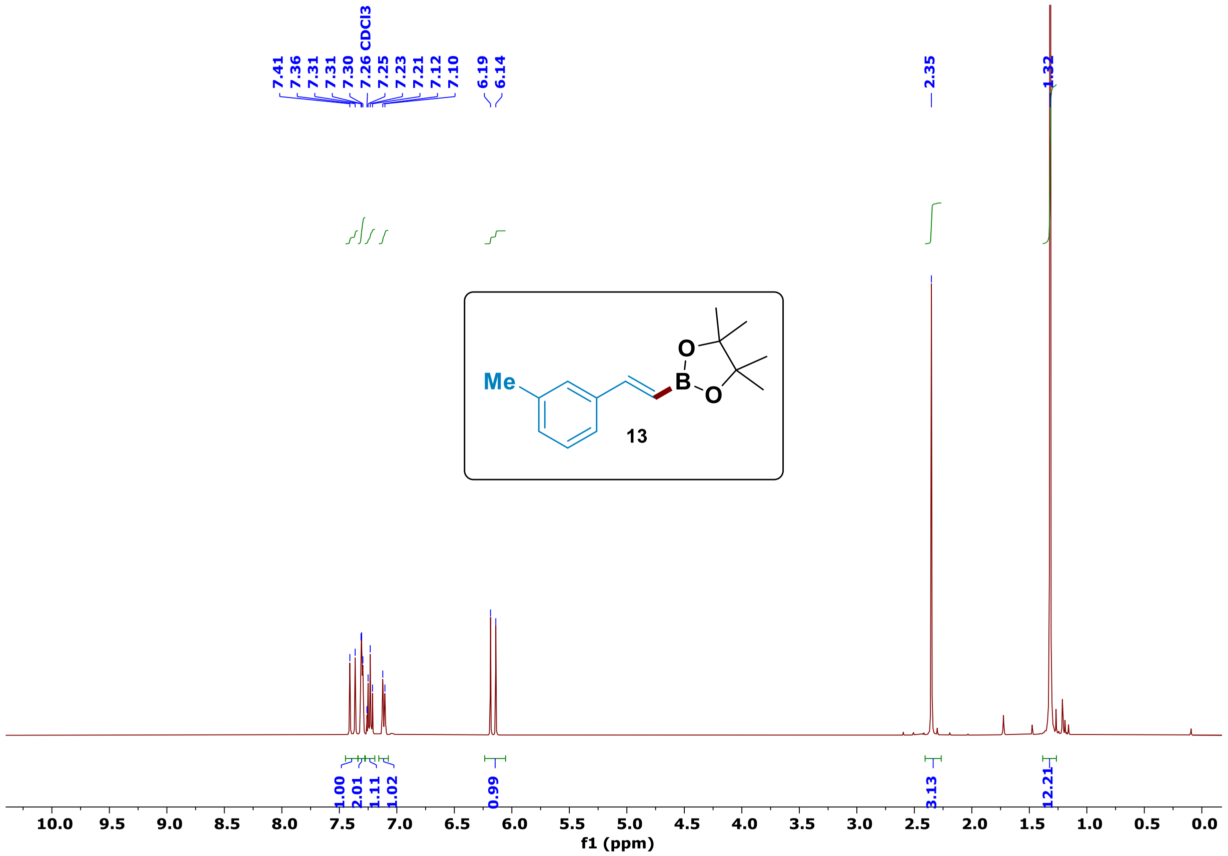

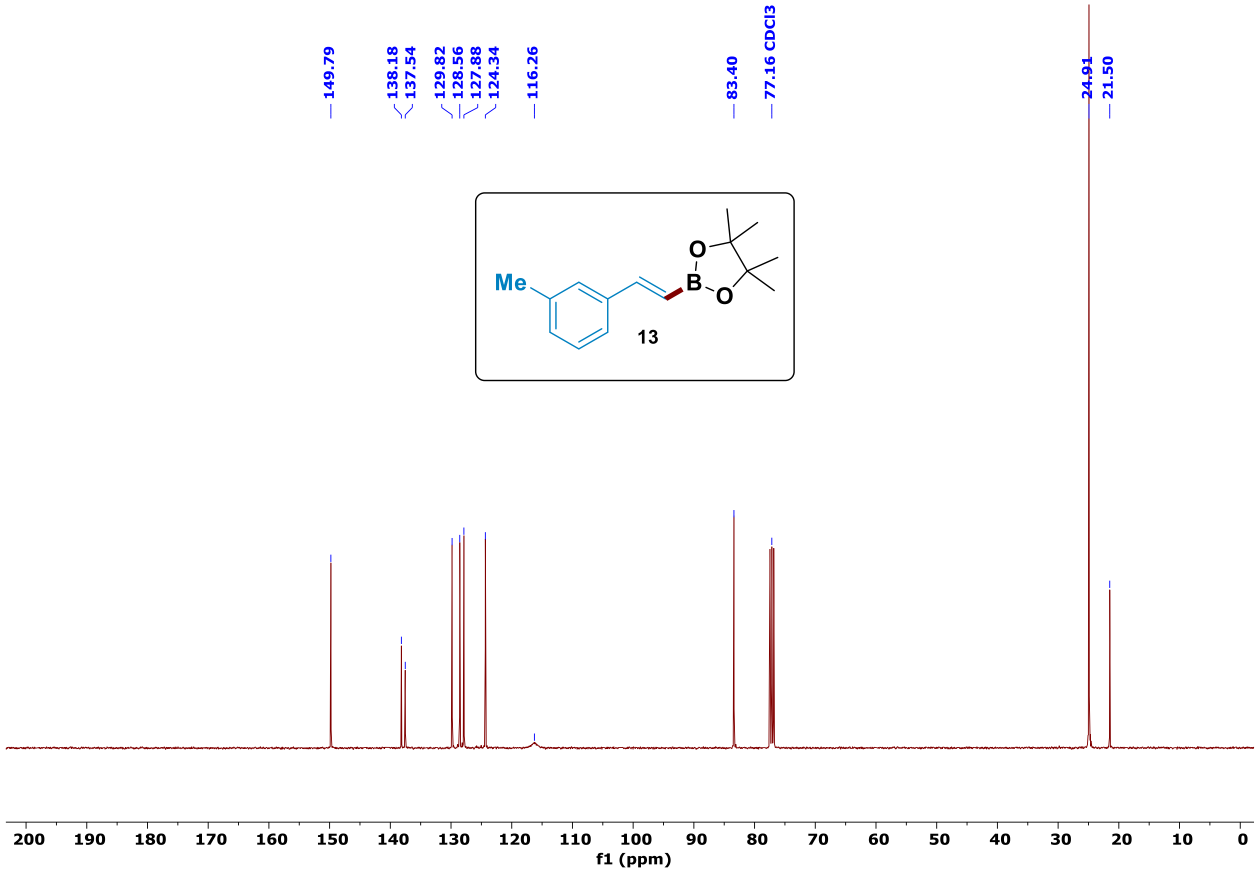

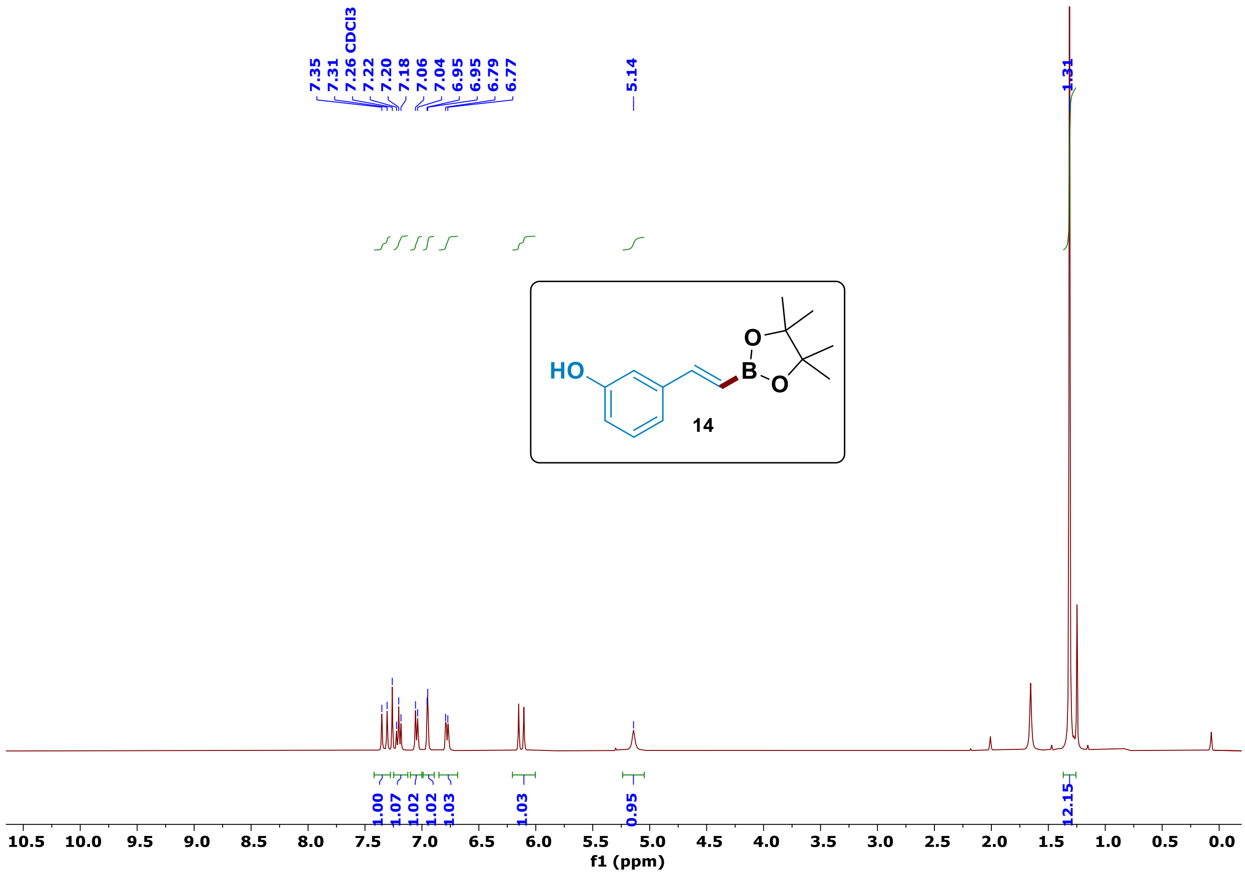
7.35
7.31
7.26 CDCl3
7.22
7.20
7.18
7.06
7.04
6.95
6.95
6.79
6.77
5.14
1.31
HO
O
B
O
14
1.00
1.07
1.02
1.02
1.03
1.03
0.95
12.15
f1 (ppm)

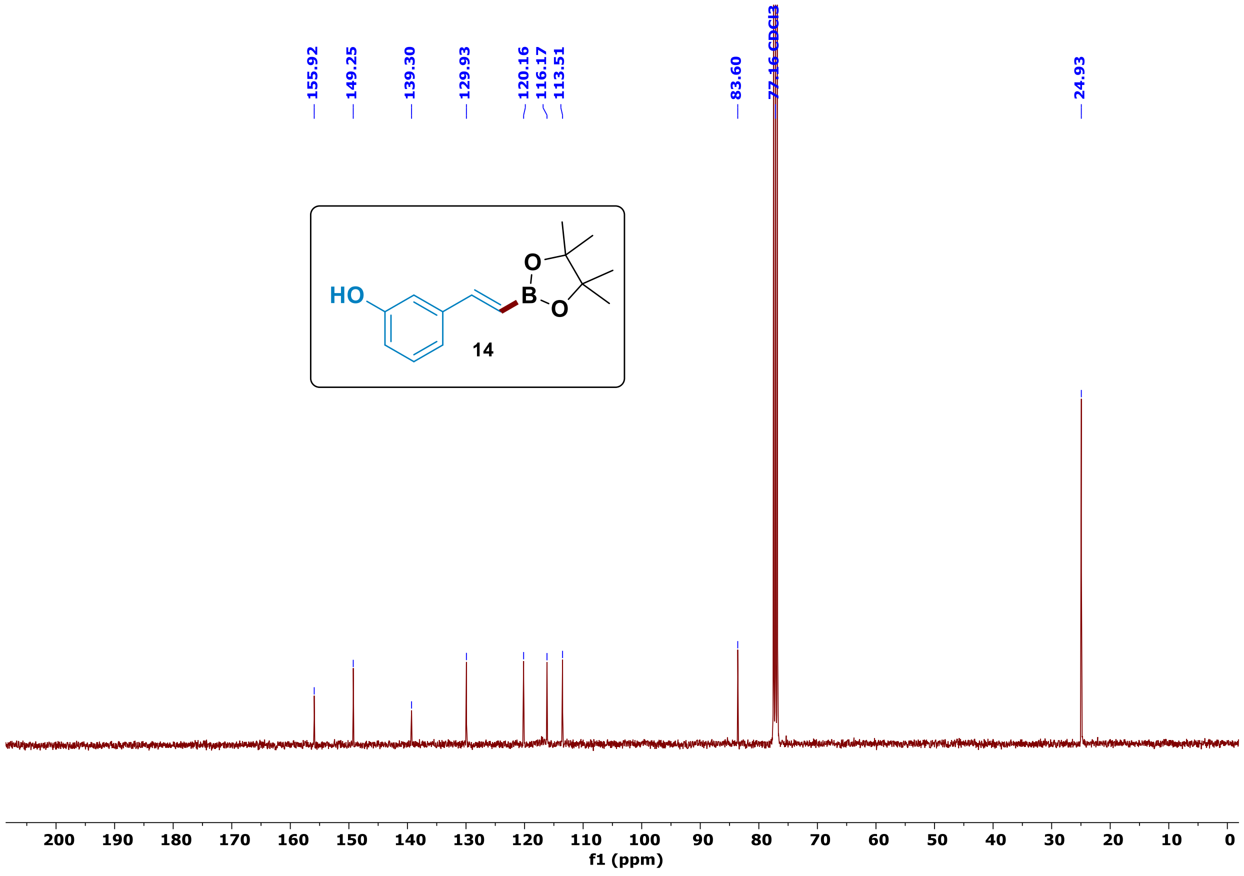
155.92
149.25
139.30
129.93
120.16
116.17
113.51
83.60
77.16 CDCl3
24.93
HO
O
B
O
14
f1 (ppm)

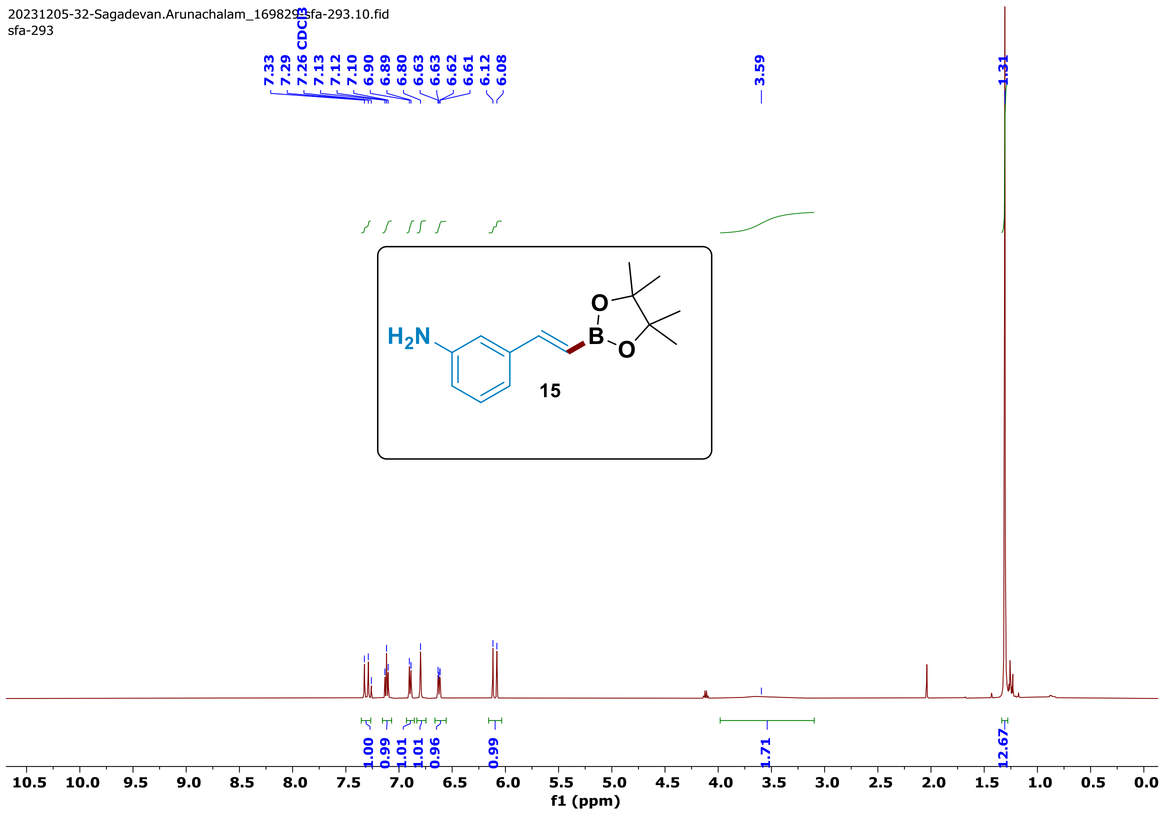
20231205-32-Sagadevan.Arunachalam_169829-sfa-293.10.fid
sfa-293
7.33 7.29 7.26 CDCl3 7.13 7.12 7.10 6.90 6.89 6.80 6.63 6.63 6.62 6.61 6.12 6.08
3.59
1.31
H2N
15
B
O
O
1.00 0.99 1.01 1.01 0.96 0.99 1.71 12.67
10.5 10.0 9.5 9.0 8.5 8.0 7.5 7.0 6.5 6.0 5.5 5.0 4.5 4.0 3.5 3.0 2.5 2.0 1.5 1.0 0.5 0.0
f1 (ppm)

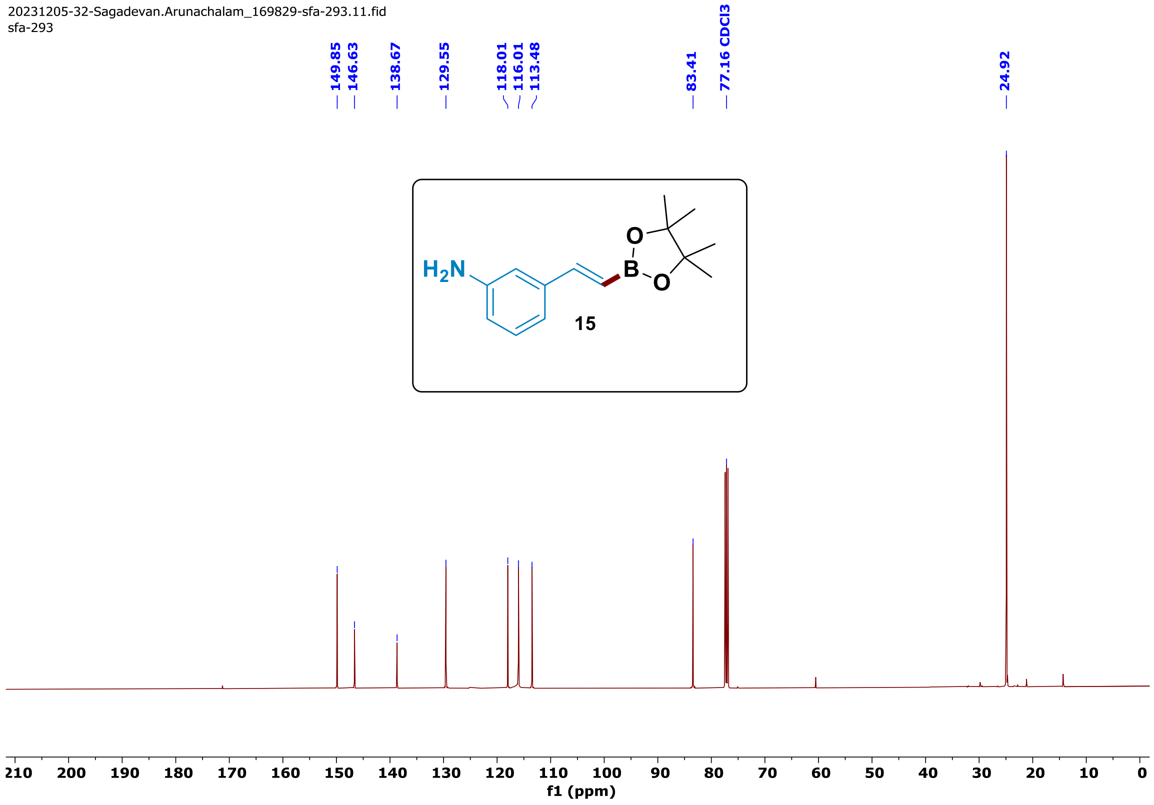
20231205-32-Sagadevan.Arunachalam_169829-sfa-293.11.fid
sfa-293
149.85 146.63 138.67 129.55 118.01 116.01 113.48 83.41 77.16 CDCl3 24.92
H2N
15
B
O
O
210 200 190 180 170 160 150 140 130 120 110 100 90 80 70 60 50 40 30 20 10 0
f1 (ppm)

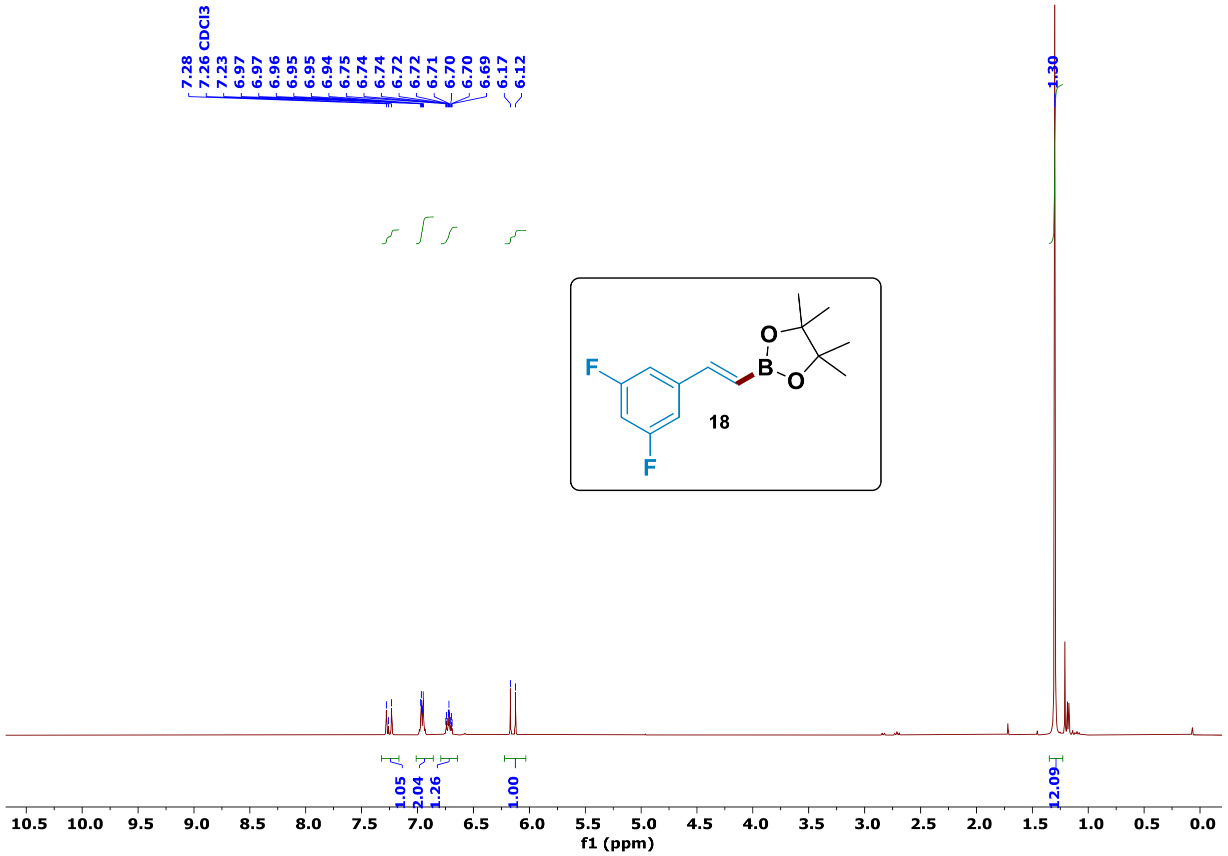
7.28
7.26 CDCl3
7.23
6.97
6.97
6.96
6.95
6.95
6.94
6.75
6.74
6.74
6.72
6.72
6.71
6.70
6.70
6.69
6.17
6.12
1.30
F
F
O
B
O
18
1.05
2.04
1.26
1.00
12.09
10.5 10.0 9.5 9.0 8.5 8.0 7.5 7.0 6.5 6.0 5.5 5.0 4.5 4.0 3.5 3.0 2.5 2.0 1.5 1.0 0.5 0.0
f1 (ppm)

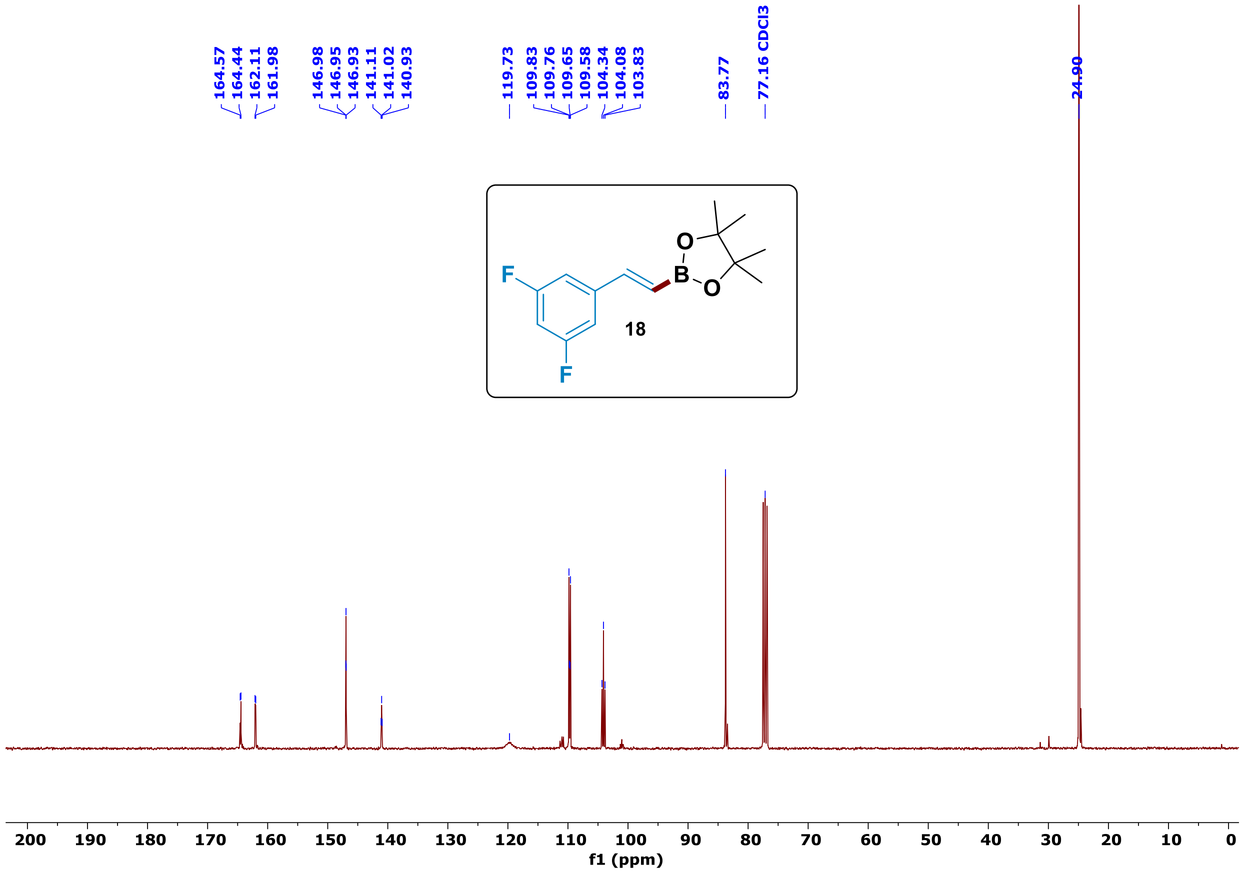
164.57
164.44
162.11
161.98
146.98
146.95
146.93
141.11
141.02
140.93
119.73
109.83
109.76
109.65
109.58
104.34
104.08
103.83
83.77
77.16 CDCl3
24.90
F
F
O
B
O
18
200 190 180 170 160 150 140 130 120 110 100 90 80 70 60 50 40 30 20 10 0
f1 (ppm)

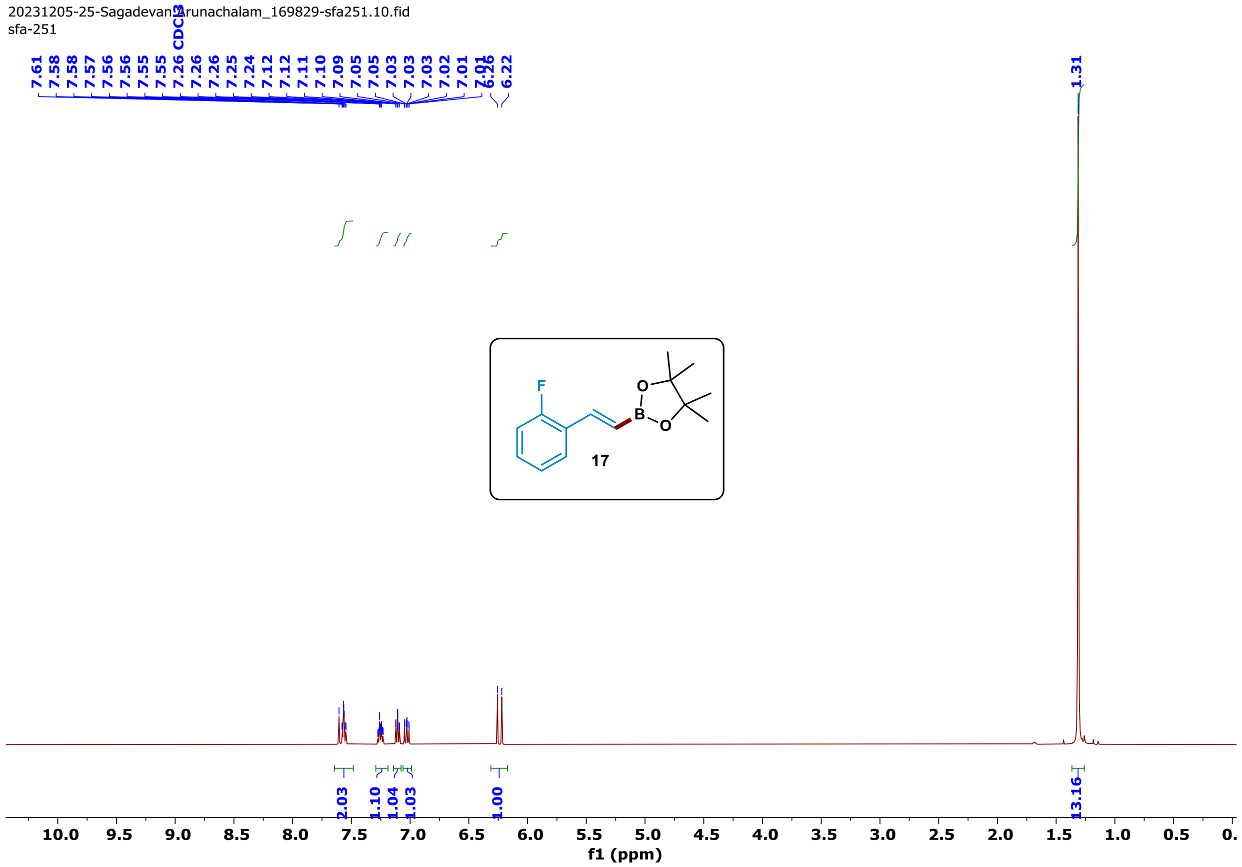
20231205-25-Sagadevan.Arunachalam_169829-sfa251.10.fid
sfa-251
7.61 7.58 7.58 7.57 7.56 7.56 7.55 7.55 7.26 CDCl3 7.26 7.26 7.25 7.24 7.12 7.12 7.11 7.10 7.09 7.05 7.05 7.03 7.03 7.03 7.02 7.01 7.01 6.26 6.22
1.31
F
O
B
O
17
2.03 1.10 1.04 1.03 1.00 13.16
f1 (ppm)

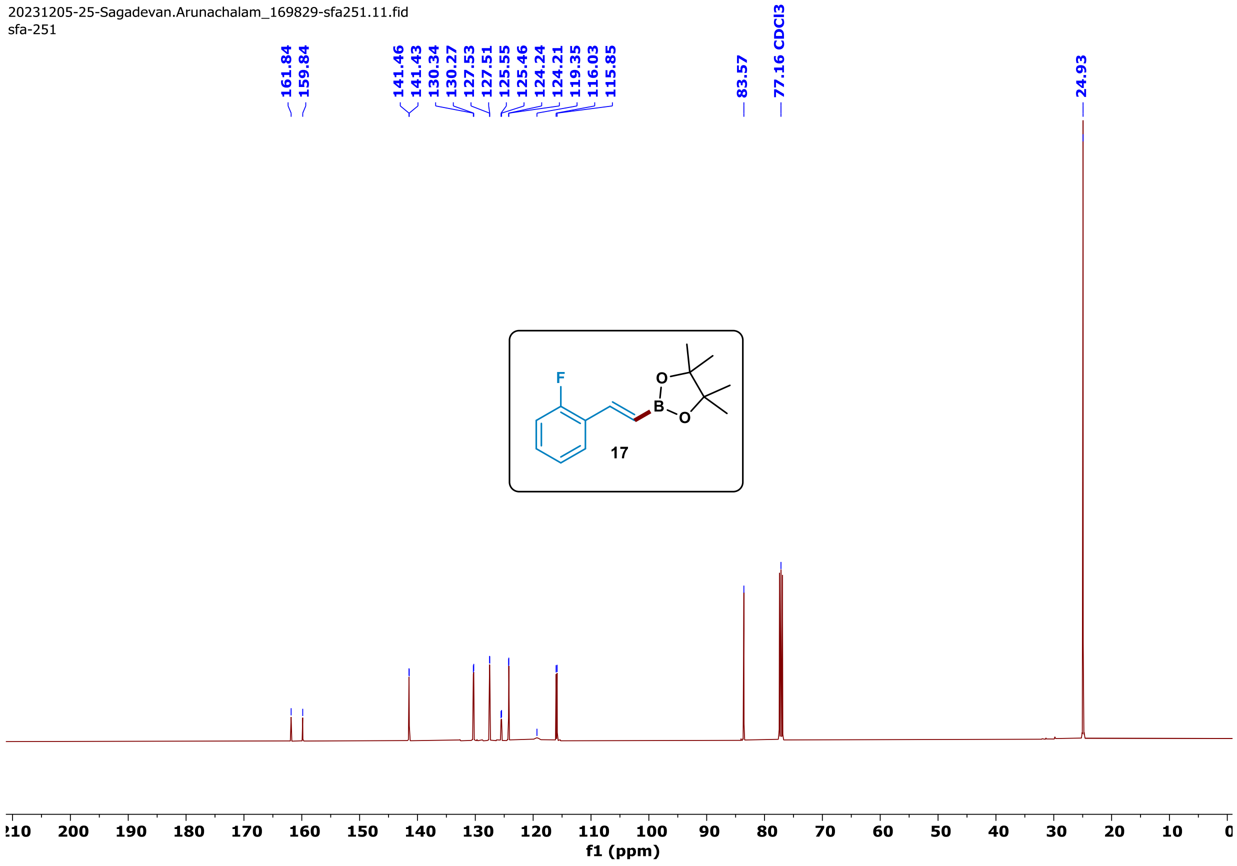
20231205-25-Sagadevan.Arunachalam_169829-sfa251.11.fid
sfa-251
161.84 159.84 141.46 141.43 130.34 130.27 127.53 127.51 125.55 125.46 124.24 124.21 119.35 116.03 115.85
83.57
77.16 CDCl3
24.93
F
O
B
O
17
f1 (ppm)

20231205-20-Sagadevan.Arunachalam_169829-sfa-252.10.fid
sfa-252

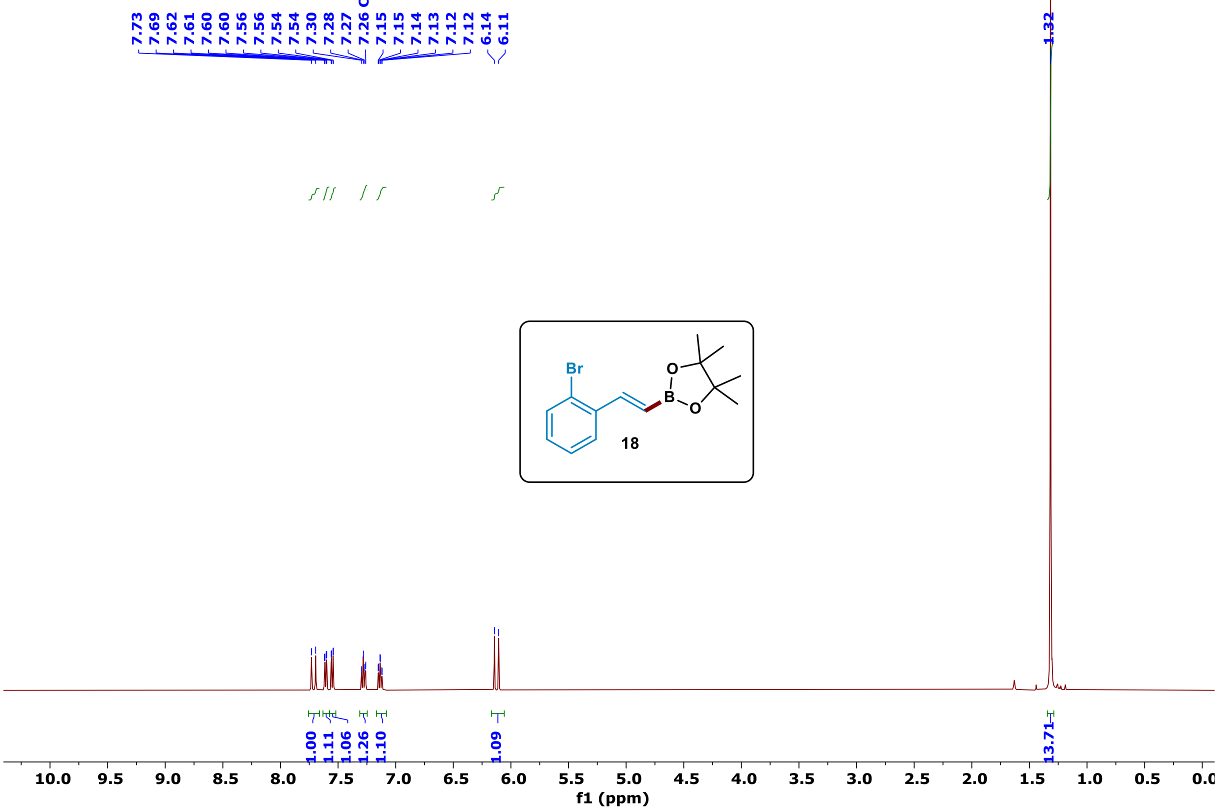

20231205-20-Sagadevan.Arunachalam_169829-sfa-252.11.fid
sfa-252

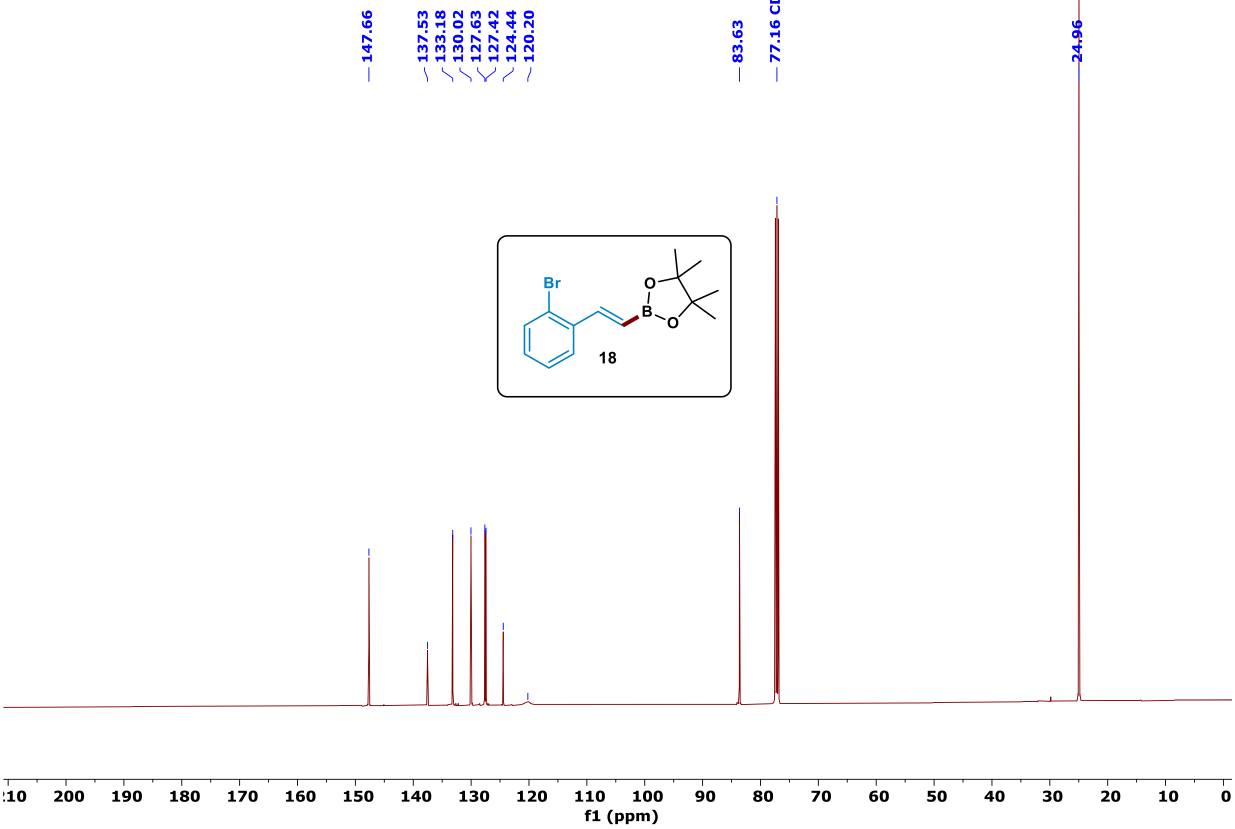

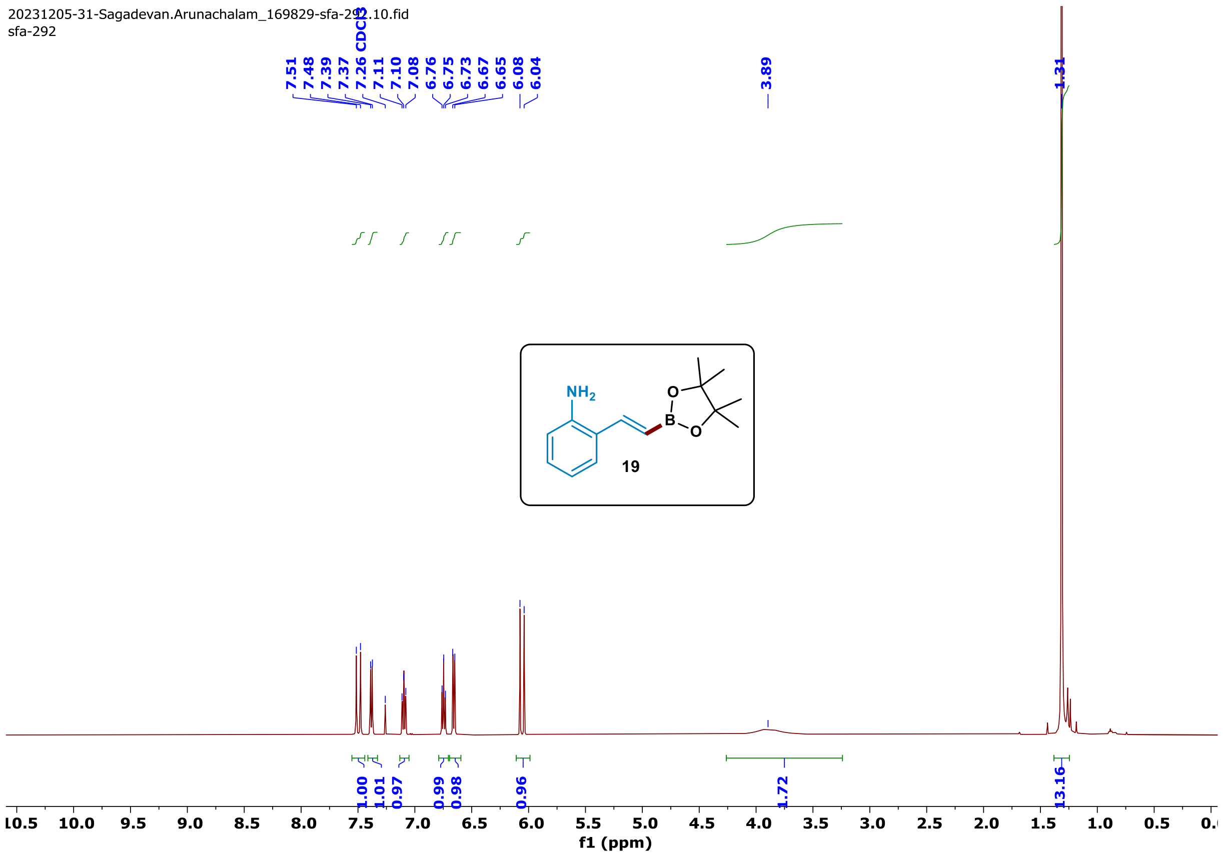
20231205-31-Sagadevan.Arunachalam_169829-sfa-292.10.fid
sfa-292
7.51 7.48 7.39 7.37 7.26 CDCl3 7.11 7.10 7.08 6.76 6.75 6.73 6.67 6.65 6.08 6.04
3.89
1.31
NH2
O
B
O
19
1.00 1.01 0.97 0.99 0.98 0.96 1.72 13.16
f1 (ppm)

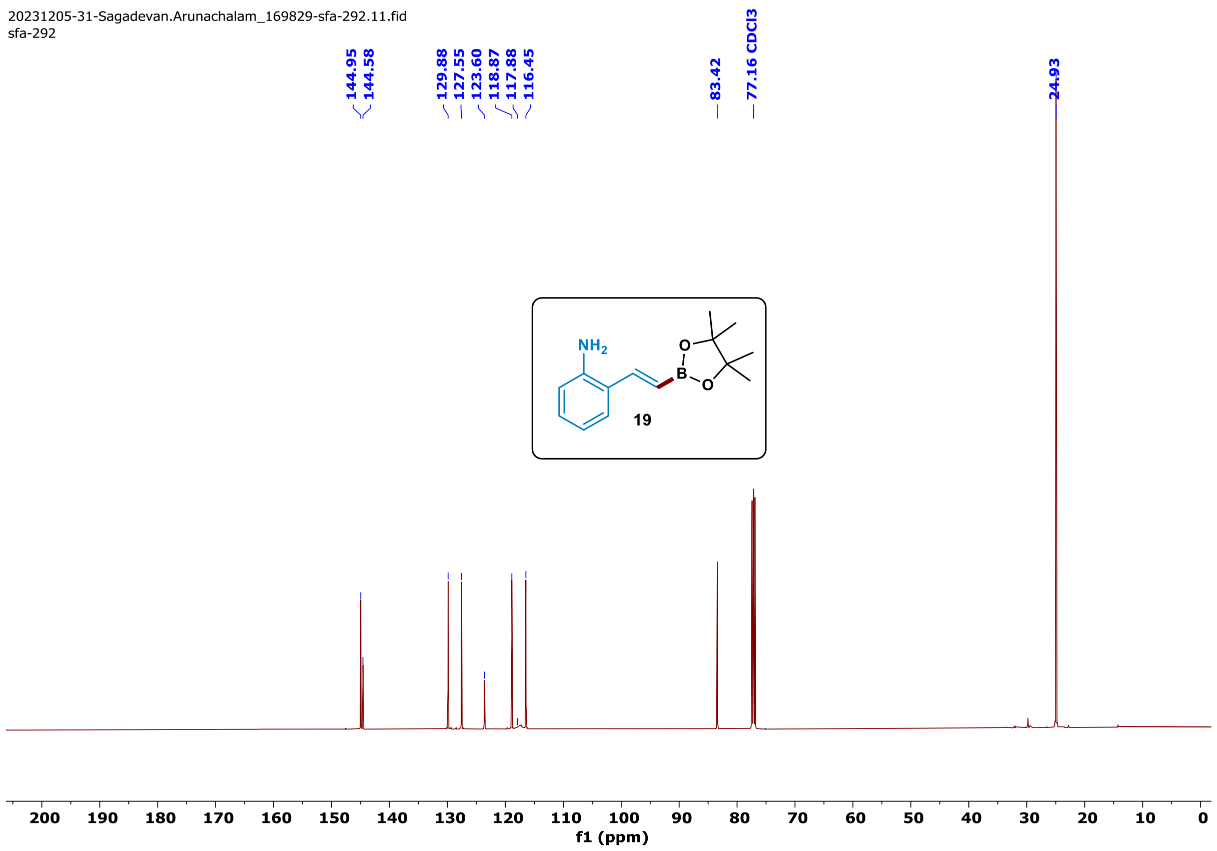
20231205-31-Sagadevan.Arunachalam_169829-sfa-292.11.fid
sfa-292
144.95 144.58
129.88 127.55 123.60 118.87 117.88 116.45
83.42
77.16 CDCl3
24.93
NH2
O
B
O
19
f1 (ppm)

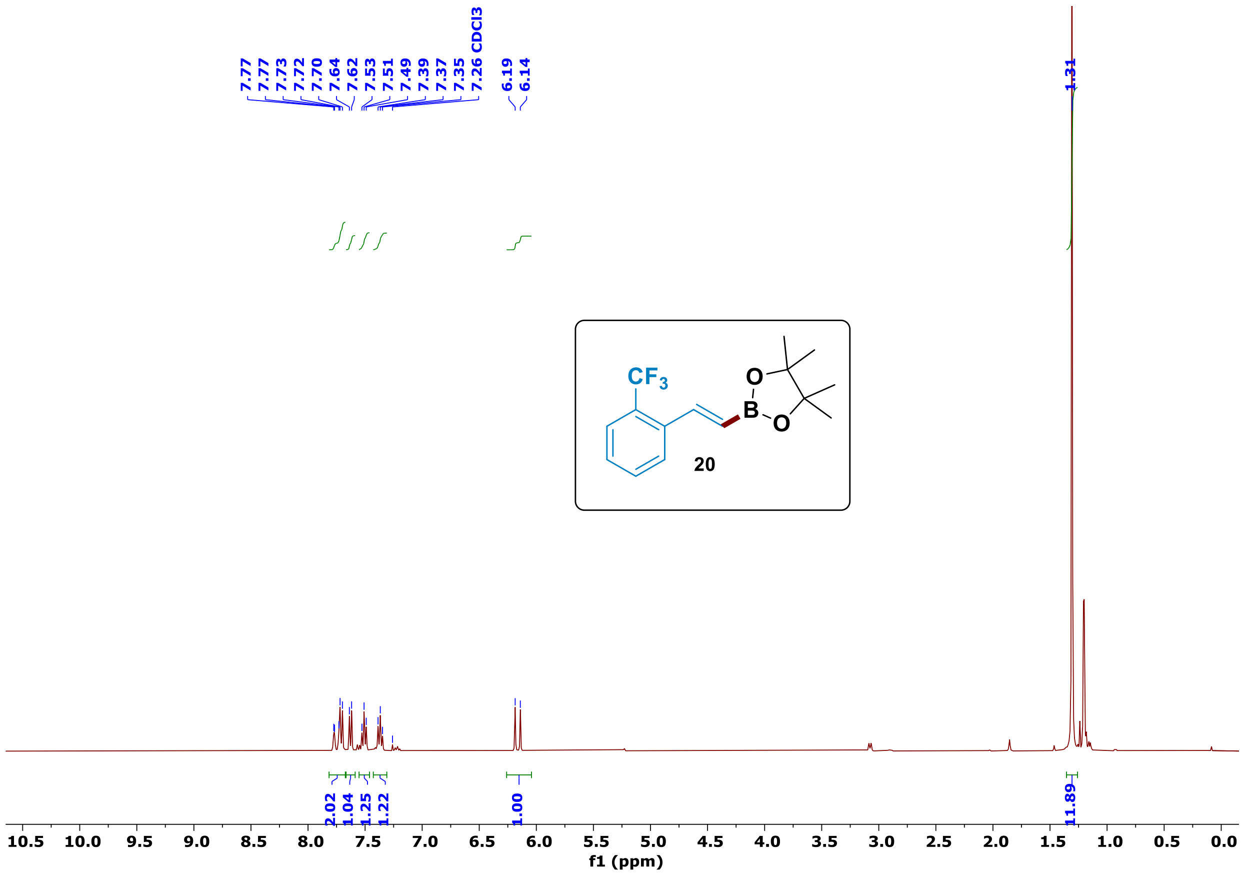
7.77 7.77 7.73 7.72 7.70 7.64 7.62 7.53 7.51 7.49 7.39 7.37 7.35 7.26 CDCl3
6.19 6.14
1.31
CF_3
20
2.02 1.04 1.25 1.22
1.00
11.89
f1 (ppm)

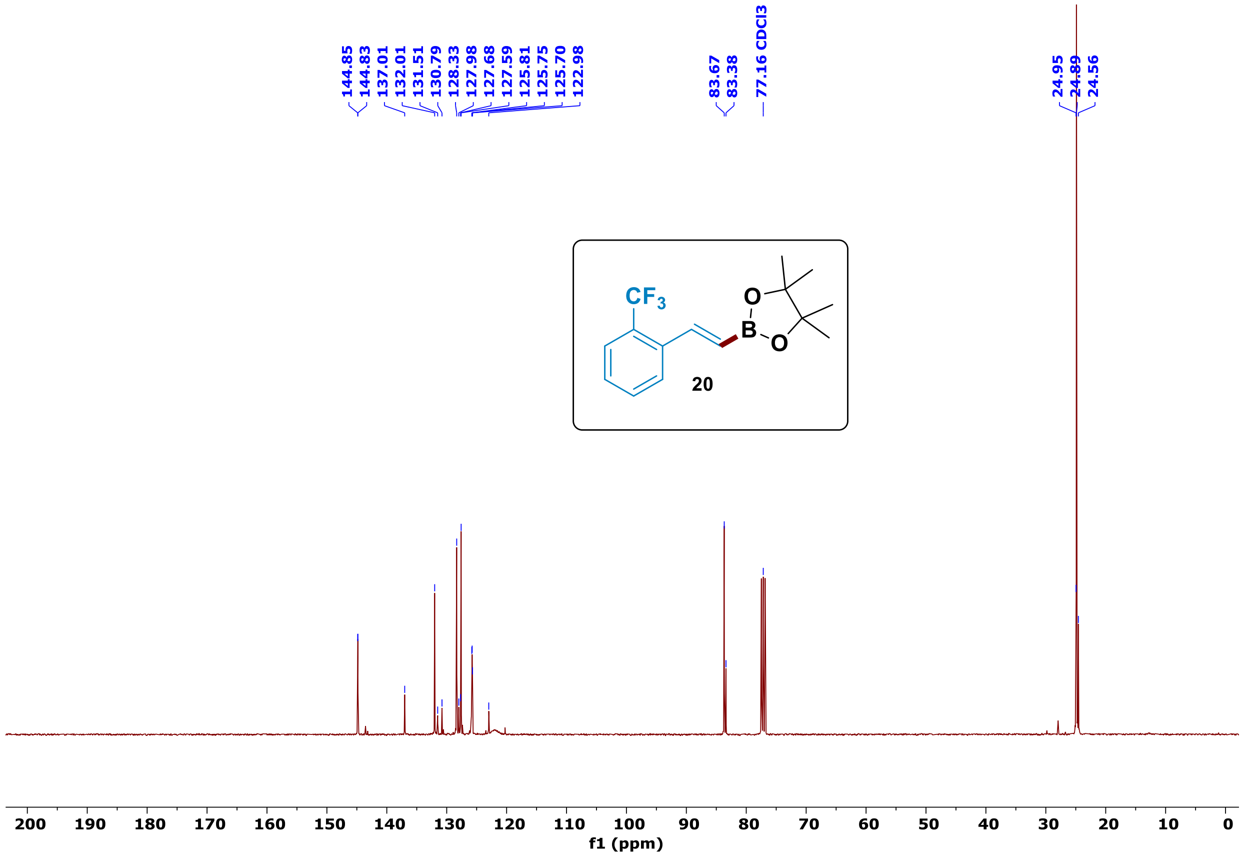
144.85 144.83 137.01 132.01 131.51 130.79 128.33 127.98 127.68 127.59 125.81 125.75 125.70 122.98
83.67 83.38
77.16 CDCl3
24.95 24.89 24.56
CF_3
20
f1 (ppm)

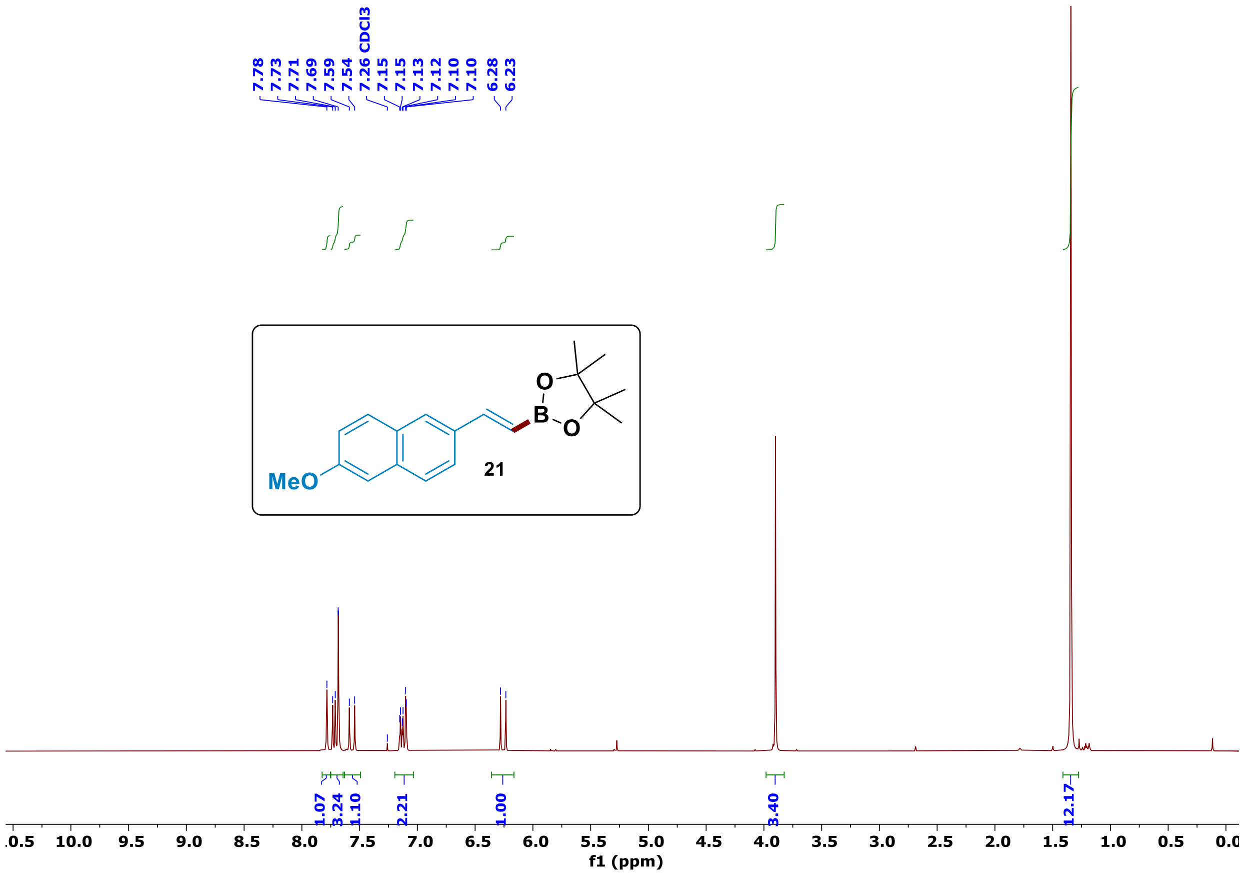

7.78
7.73
7.71
7.69
7.59
7.54
7.26 CDCl3
7.15
7.15
7.13
7.12
7.10
7.10
6.28
6.23
MeO
21
1.07
3.24
1.10
2.21
1.00
3.40
12.17
f1 (ppm)

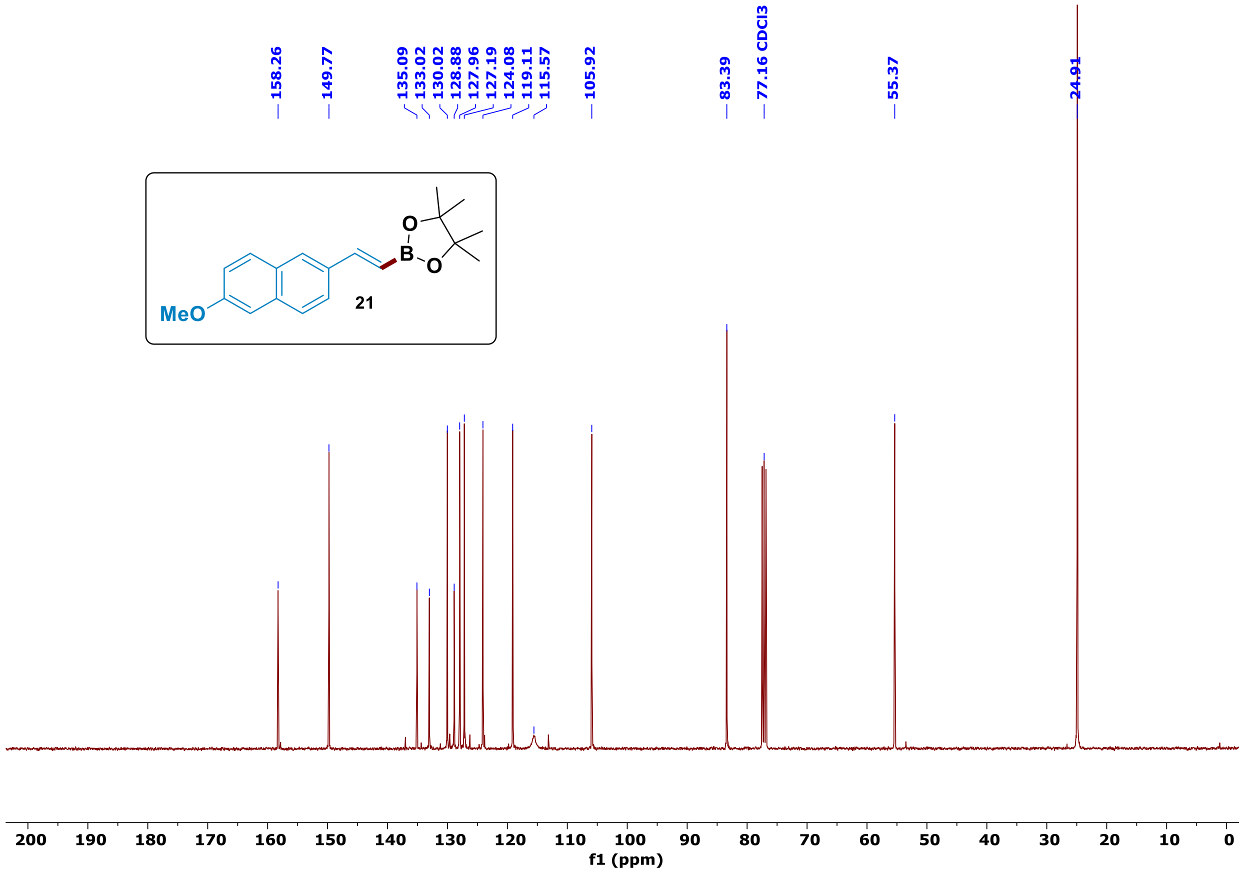

158.26
149.77
135.09
133.02
130.02
128.88
127.96
127.19
124.08
119.11
115.57
105.92
83.39
77.16 CDCl3
55.37
24.91
MeO
21
f1 (ppm)

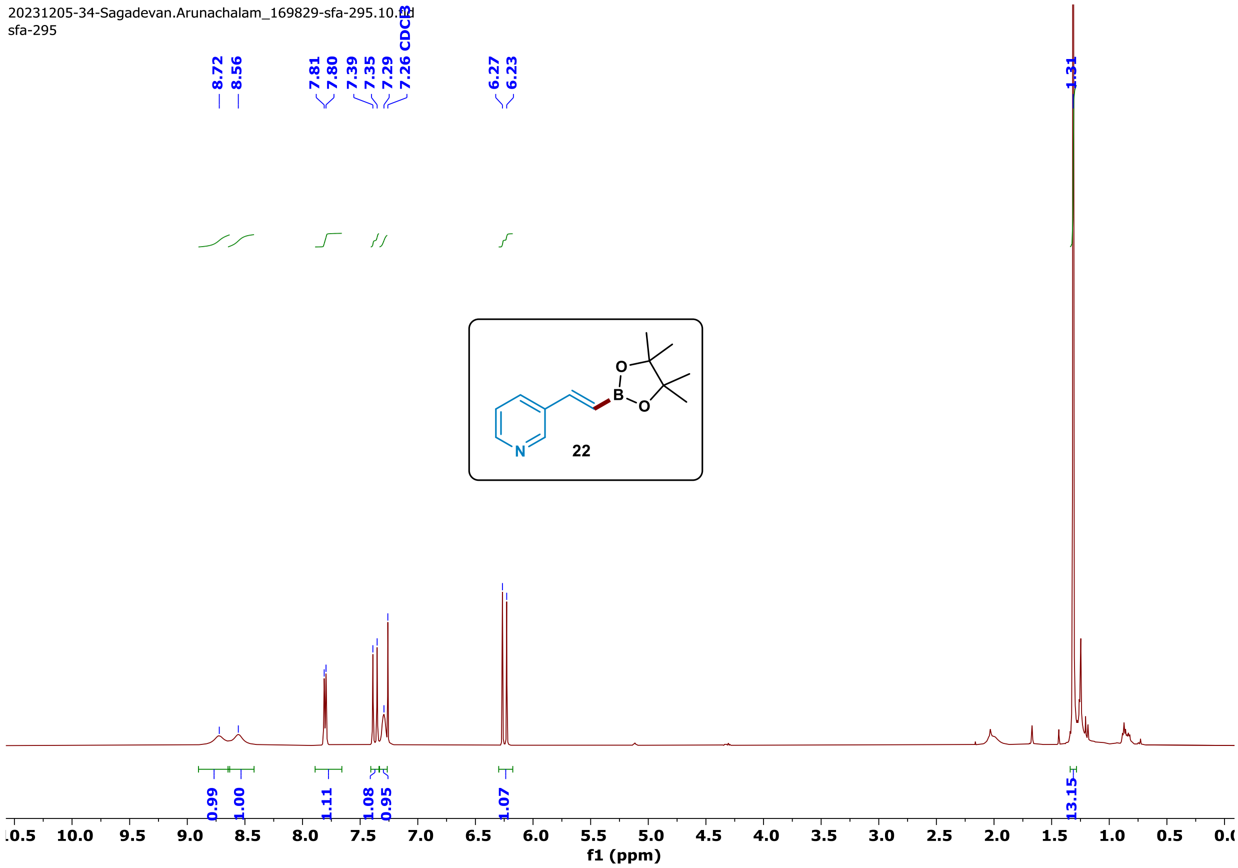
20231205-34-Sagadevan.Arunachalam_169829-sfa-295.10.fid
sfa-295
8.72
8.56
7.81
7.80
7.39
7.35
7.29
7.26 CDCl3
6.27
6.23
1.31
N
O
B
O
22
0.99
1.00
1.11
1.08
0.95
1.07
13.15
f1 (ppm)

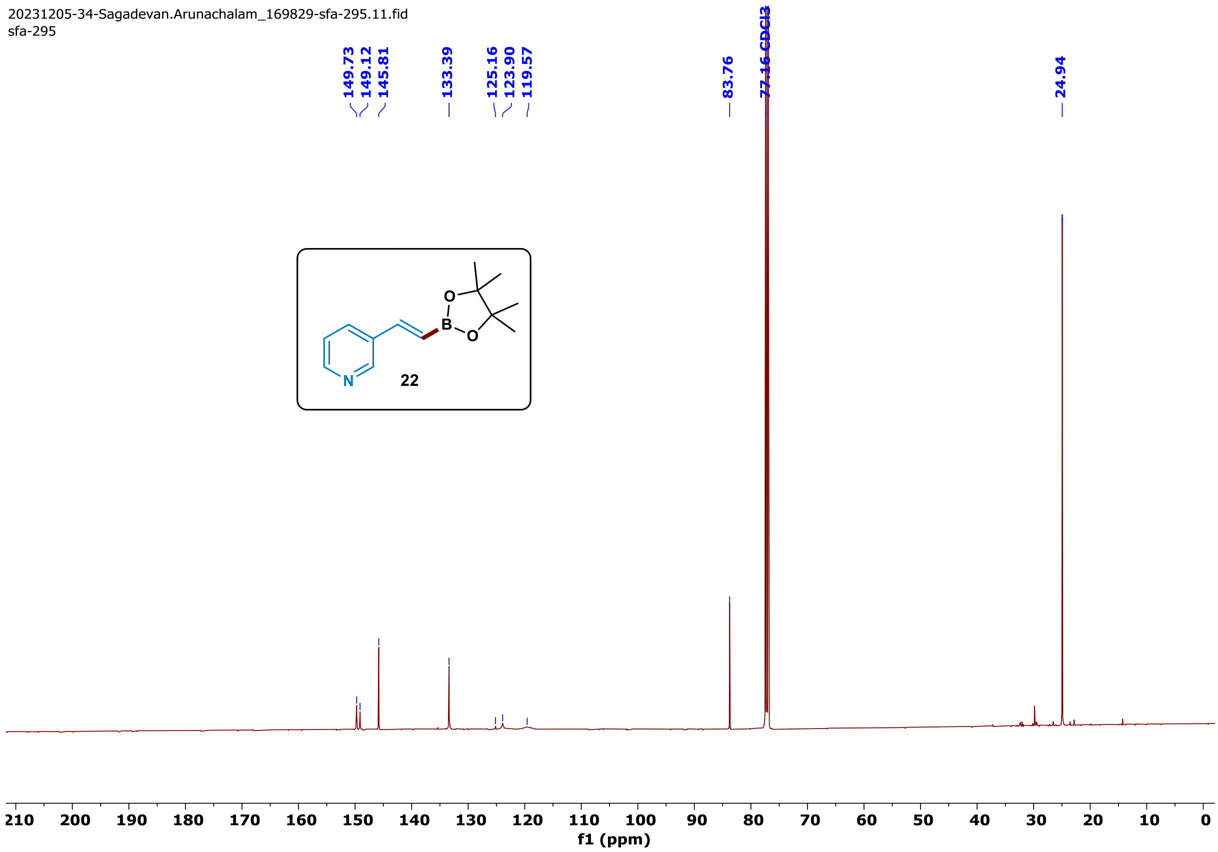
20231205-34-Sagadevan.Arunachalam_169829-sfa-295.11.fid
sfa-295
149.73
149.12
145.81
133.39
125.16
123.90
119.57
83.76
77.16 CDCl3
24.94
N
O
B
O
22
f1 (ppm)

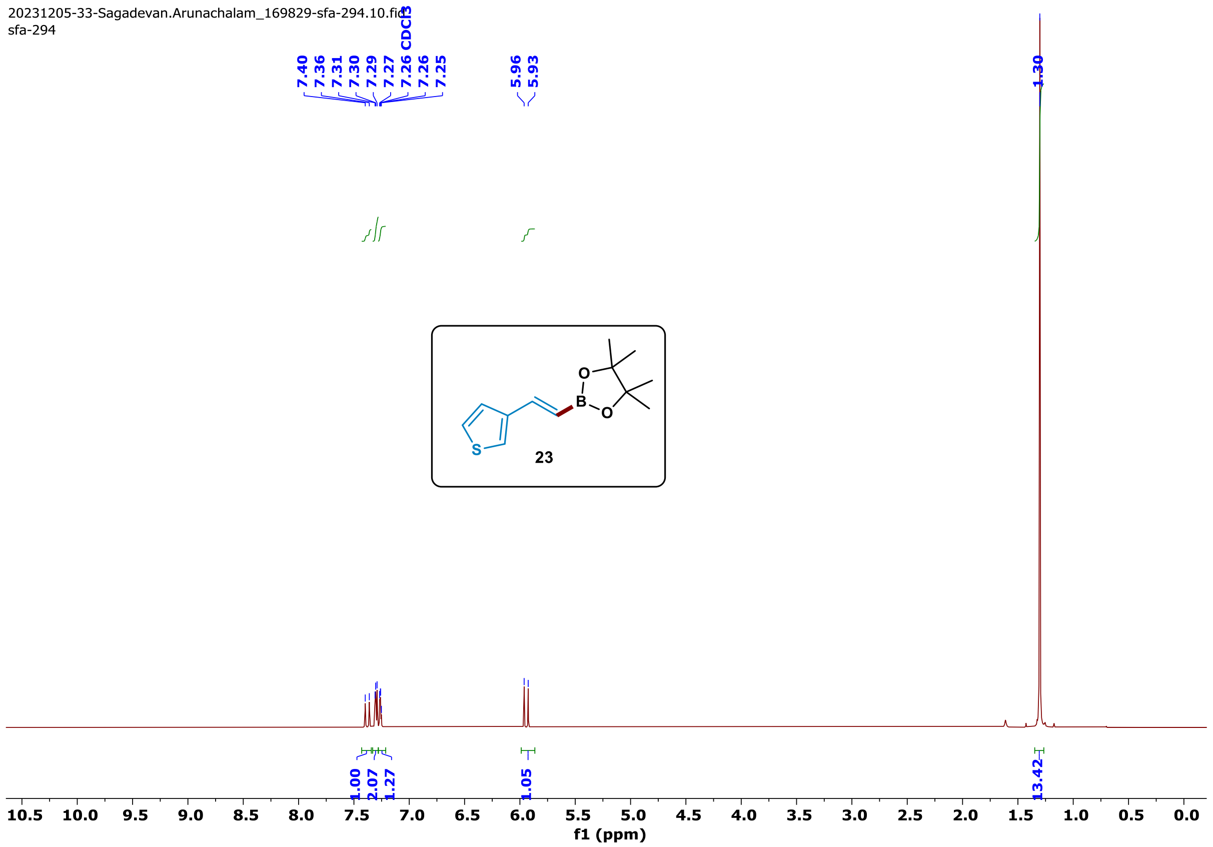
20231205-33-Sagadevan.Arunachalam_169829-sfa-294.10.fid
sfa-294
7.40
7.36
7.31
7.30
7.29
7.27
7.26 CDCl3
7.26
7.25
5.96
5.93
1.30
S
O
B
O
23
1.00
2.07
1.27
1.05
13.42
10.5 10.0 9.5 9.0 8.5 8.0 7.5 7.0 6.5 6.0 5.5 5.0 4.5 4.0 3.5 3.0 2.5 2.0 1.5 1.0 0.5 0.0
f1 (ppm)

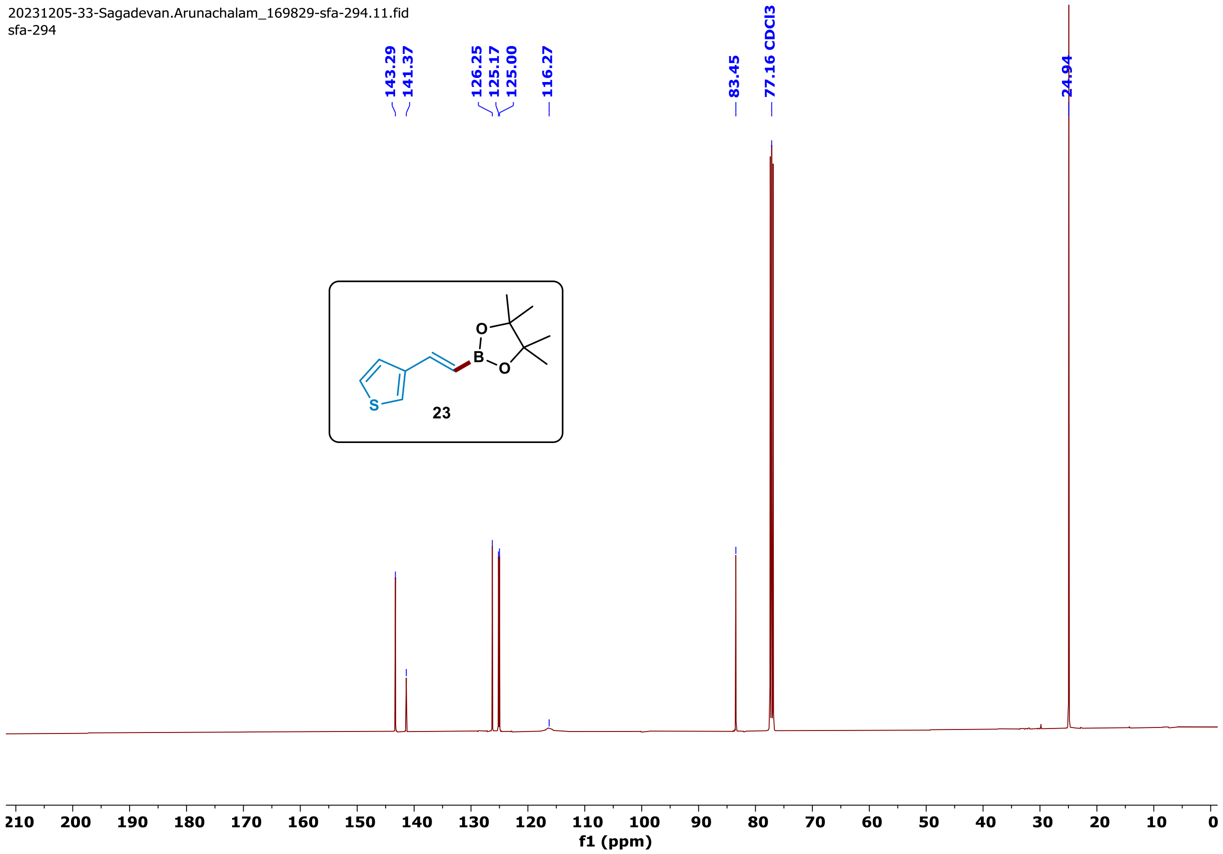
20231205-33-Sagadevan.Arunachalam_169829-sfa-294.11.fid
sfa-294
143.29
141.37
126.25
125.17
125.00
116.27
83.45
77.16 CDCl3
24.94
S
O
B
O
23
210 200 190 180 170 160 150 140 130 120 110 100 90 80 70 60 50 40 30 20 10 0
f1 (ppm)

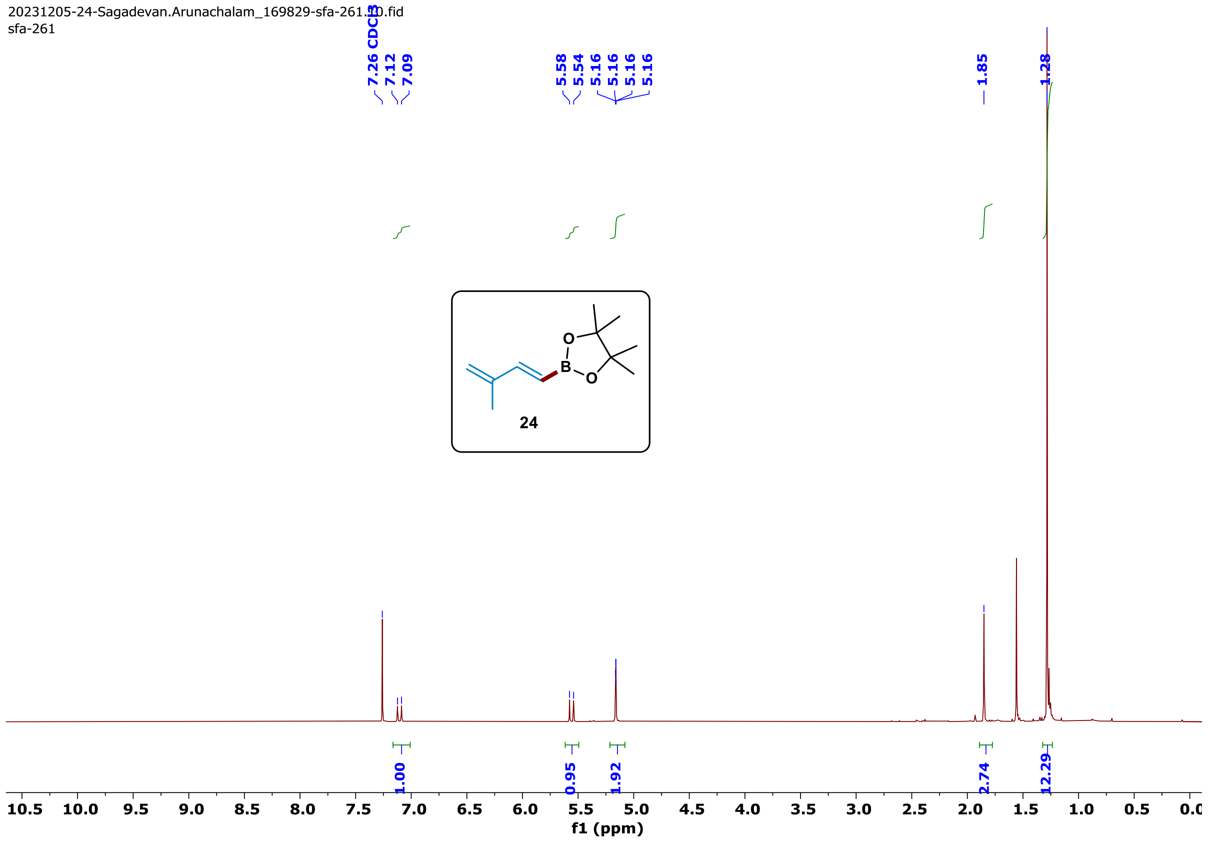
20231205-24-Sagadevan.Arunachalam_169829-sfa-261.10.fid
sfa-261
7.26 CDCl3
7.12
7.09
5.58
5.54
5.16
5.16
5.16
5.16
1.85
1.28
24
1.00
0.95
1.92
2.74
12.29
f1 (ppm)

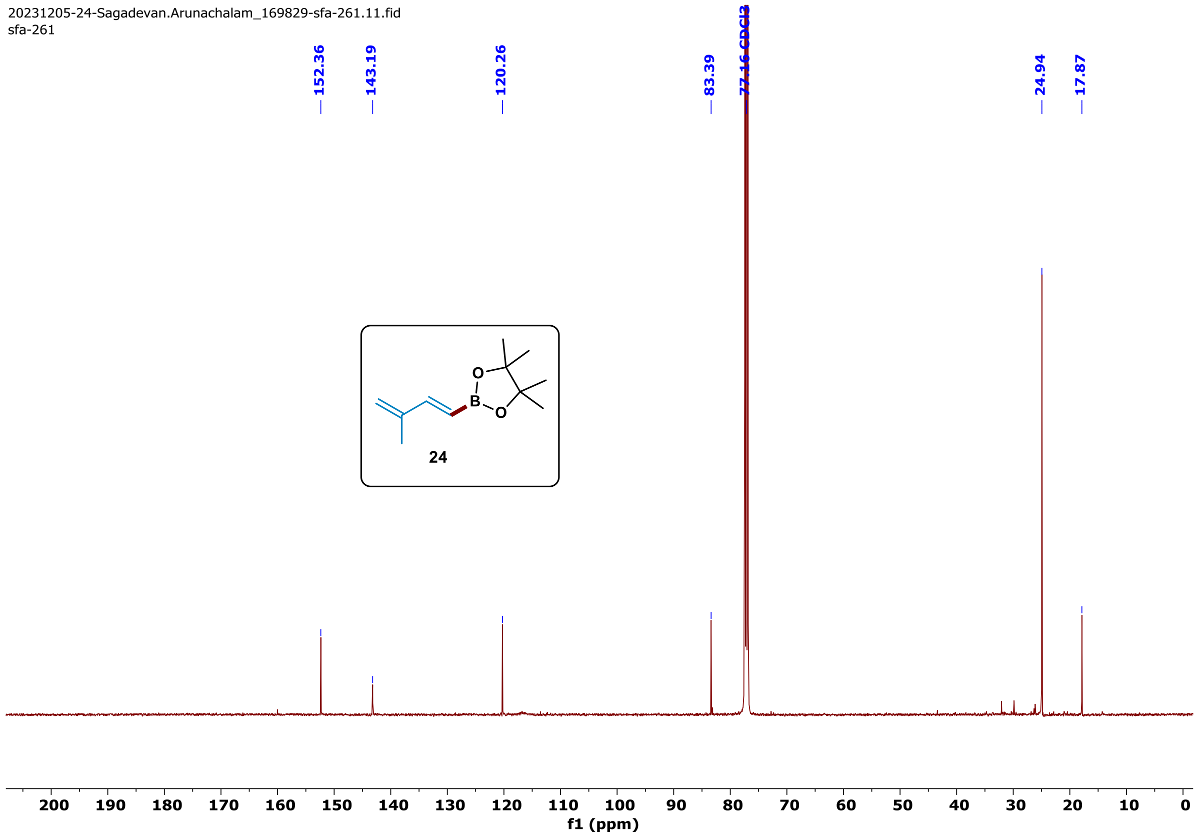
20231205-24-Sagadevan.Arunachalam_169829-sfa-261.11.fid
sfa-261
152.36
143.19
120.26
83.39
77.16 CDCl3
24.94
17.87
24
f1 (ppm)

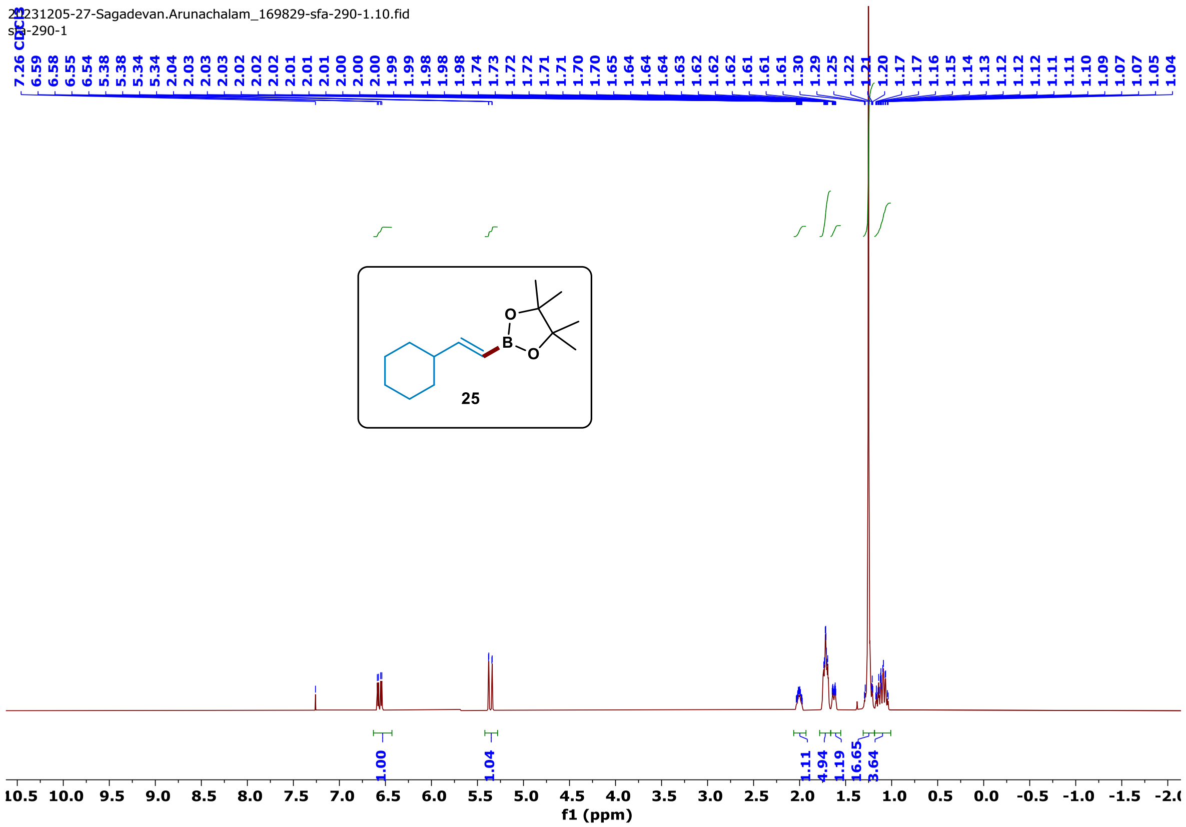
20231205-27-Sagadevan.Arunachalam_169829-sfa-290-1.10.fid
sfa-290-1
7.26 CDCl3
6.59 6.58 6.55 6.54 5.38 5.38 5.34 5.34 2.04 2.03 2.03 2.02 2.02 2.01 2.01 2.00 2.00 1.99 1.99 1.98 1.98 1.74 1.73 1.72 1.72 1.71 1.71 1.70 1.70 1.65 1.64 1.64 1.63 1.62 1.62 1.62 1.61 1.61 1.61 1.30 1.29 1.25 1.22 1.21 1.20 1.17 1.17 1.16 1.15 1.14 1.13 1.12 1.12 1.12 1.11 1.11 1.10 1.09 1.07 1.07 1.05 1.04
O
B
O
25
1.00
1.04
1.11
4.94
1.19
16.65
3.64
f1 (ppm)

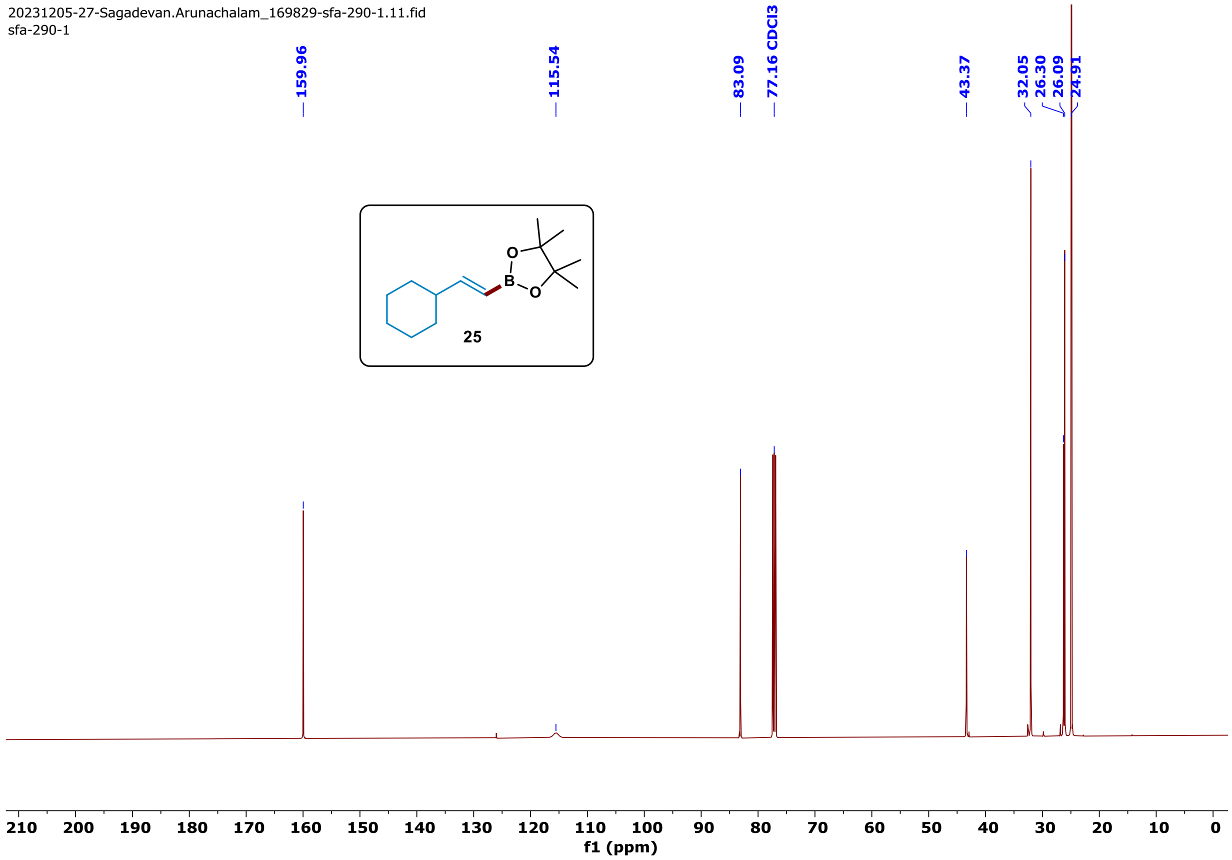
20231205-27-Sagadevan.Arunachalam_169829-sfa-290-1.11.fid
sfa-290-1
159.96
115.54
83.09
77.16 CDCl3
43.37
32.05
26.30
26.09
24.91
O
B
O
25
f1 (ppm)

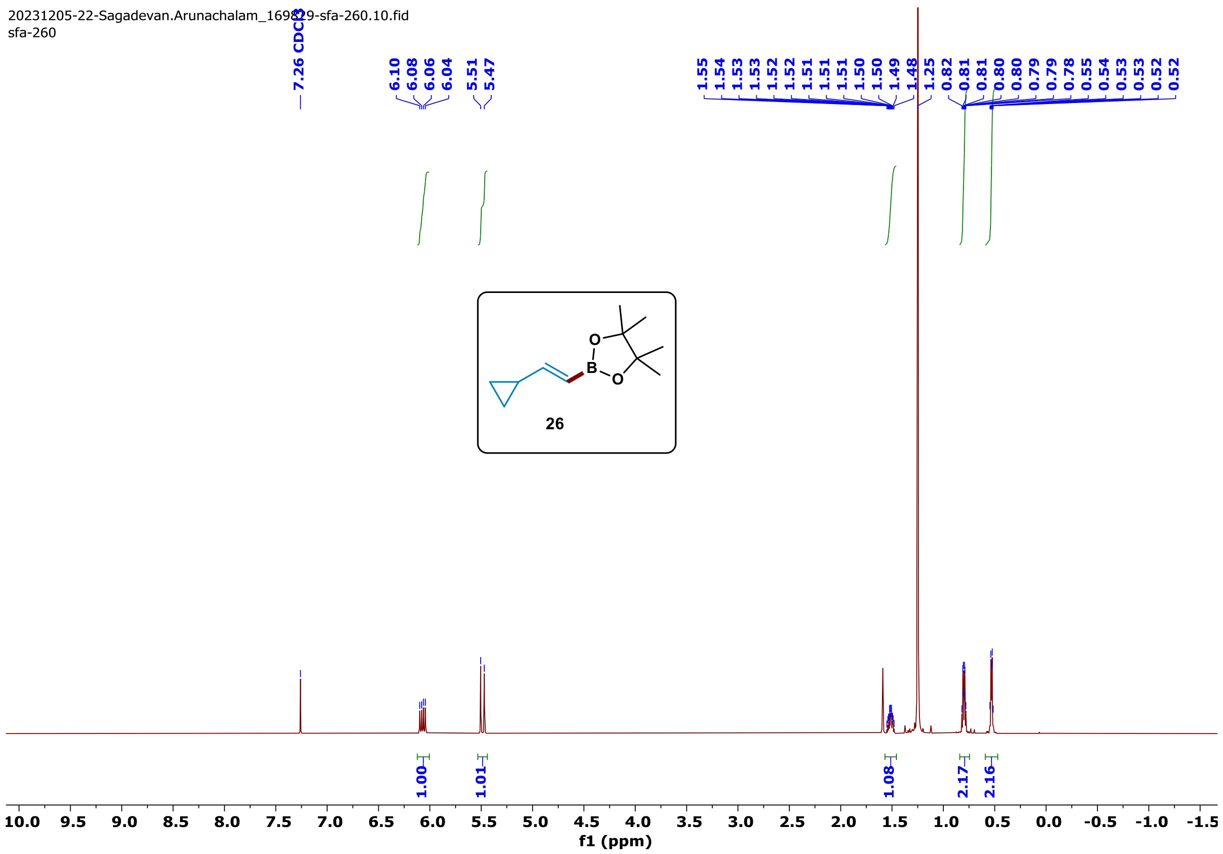
20231205-22-Sagadevan.Arunachalam_169829-sfa-260.10.fid
sfa-260
7.26 CDCl3
6.10
6.08
6.06
6.04
5.51
5.47
1.55
1.54
1.53
1.53
1.52
1.52
1.51
1.51
1.51
1.50
1.50
1.49
1.48
1.25
0.82
0.81
0.81
0.80
0.80
0.79
0.79
0.78
0.55
0.54
0.53
0.53
0.52
0.52
B
O
O
26
1.00
1.01
1.08
2.17
2.16
f1 (ppm)

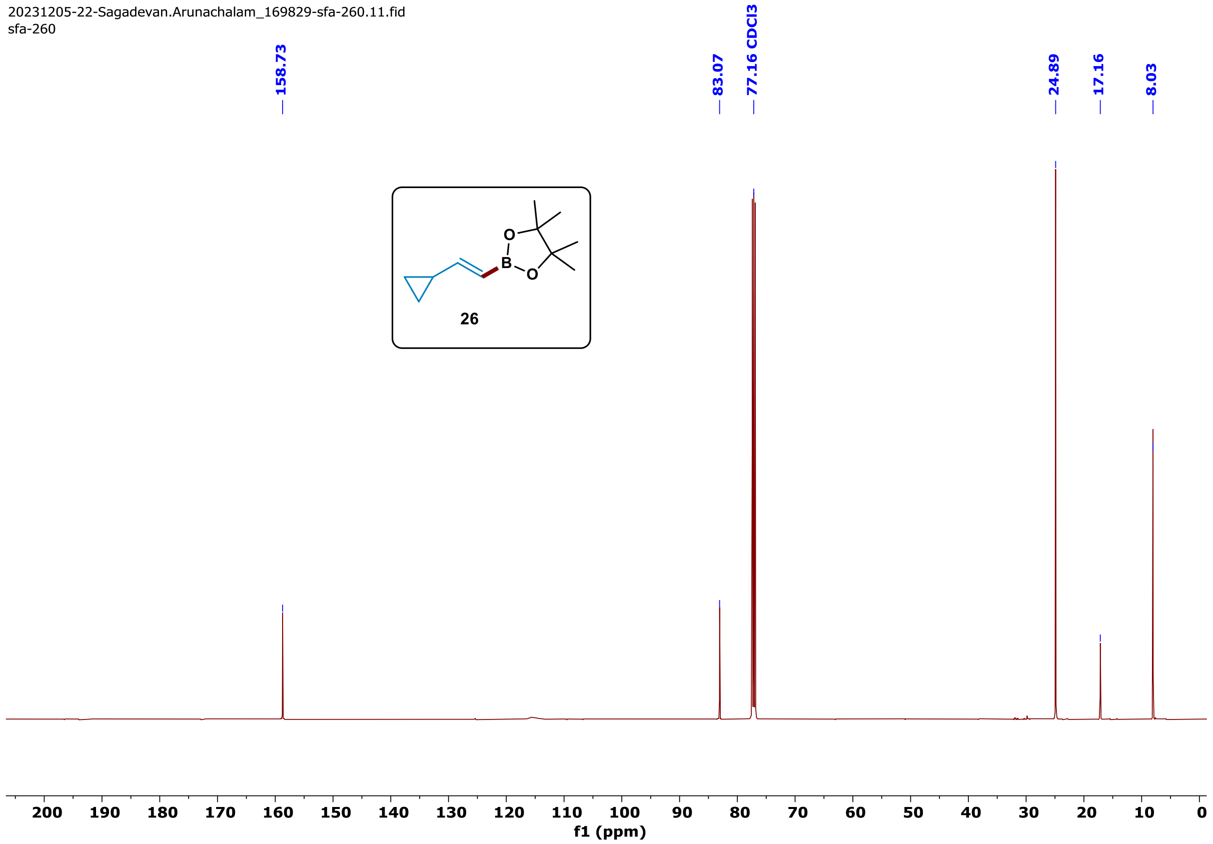
20231205-22-Sagadevan.Arunachalam_169829-sfa-260.11.fid
sfa-260
158.73
83.07
77.16 CDCl3
24.89
17.16
8.03
B
O
O
26
f1 (ppm)

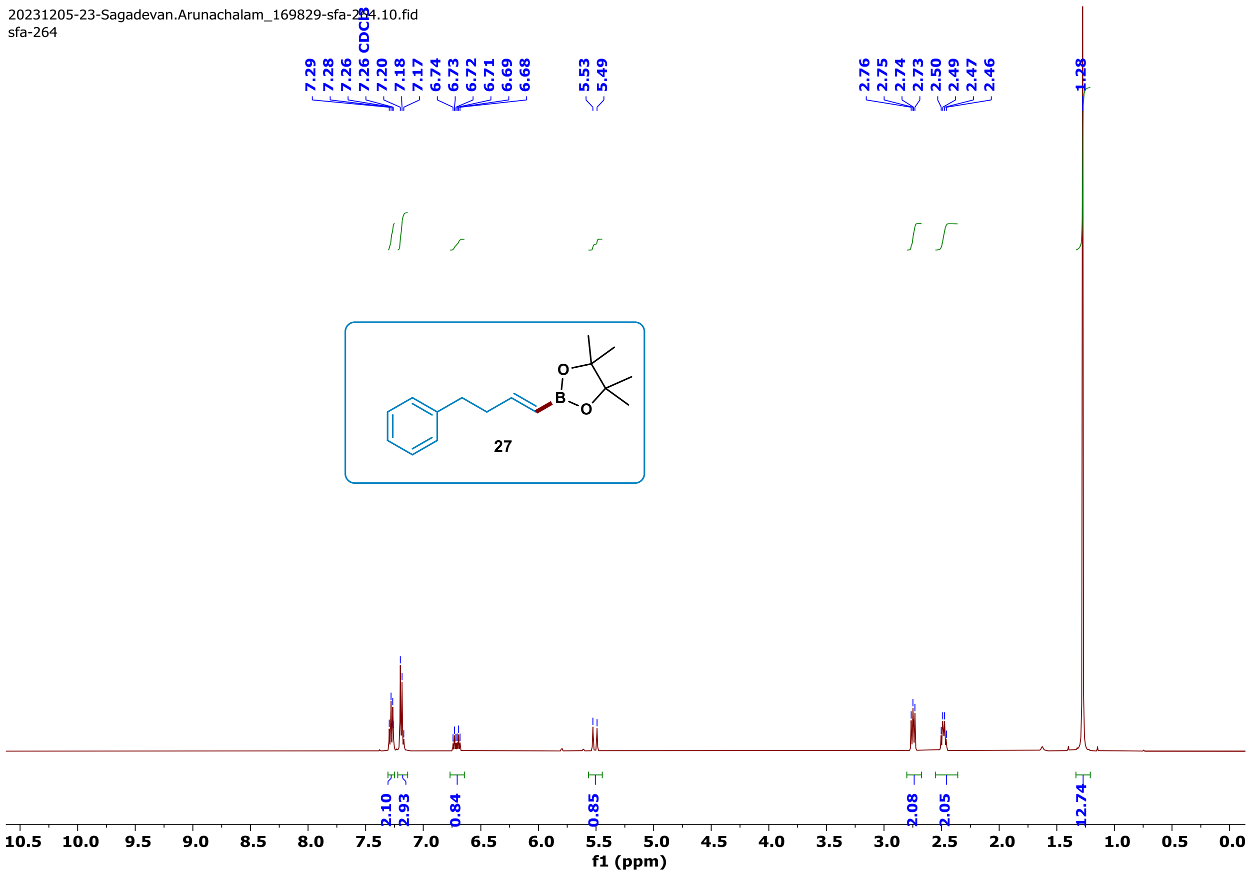
20231205-23-Sagadevan.Arunachalam_169829-sfa-264.10.fid
sfa-264
7.29
7.28
7.26
7.26 CDCl3
7.20
7.18
7.17
6.74
6.73
6.72
6.71
6.69
6.68
5.53
5.49
2.76
2.75
2.74
2.73
2.50
2.49
2.47
2.46
1.28
B
O
O
27
2.10
2.93
0.84
0.85
2.08
2.05
12.74
10.5
10.0
9.5
9.0
8.5
8.0
7.5
7.0
6.5
6.0
5.5
5.0
4.5
4.0
3.5
3.0
2.5
2.0
1.5
1.0
0.5
0.0
f1 (ppm)

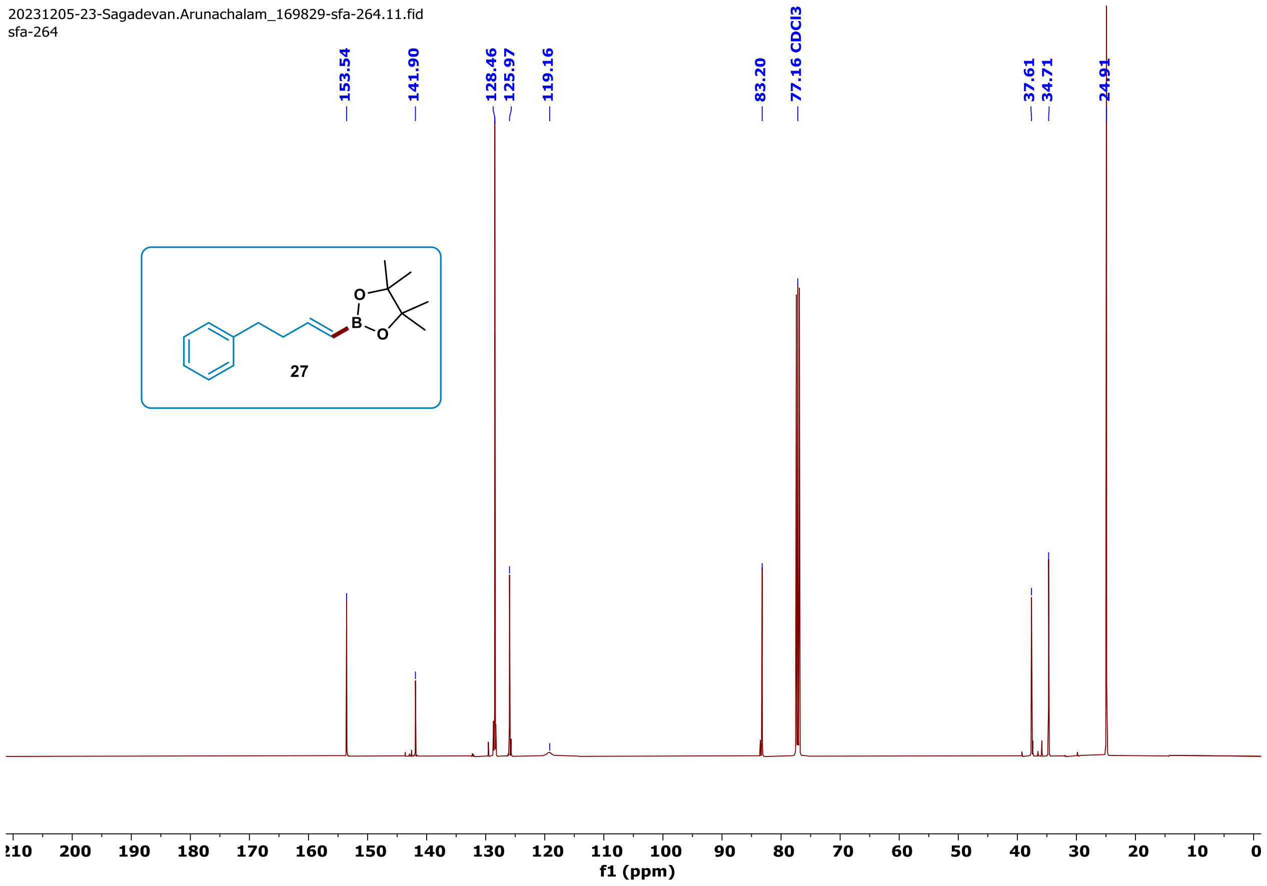
20231205-23-Sagadevan.Arunachalam_169829-sfa-264.11.fid
sfa-264
153.54
141.90
128.46
125.97
119.16
83.20
77.16 CDCl3
37.61
34.71
24.91
B
O
O
27
210
200
190
180
170
160
150
140
130
120
110
100
90
80
70
60
50
40
30
20
10
0
f1 (ppm)

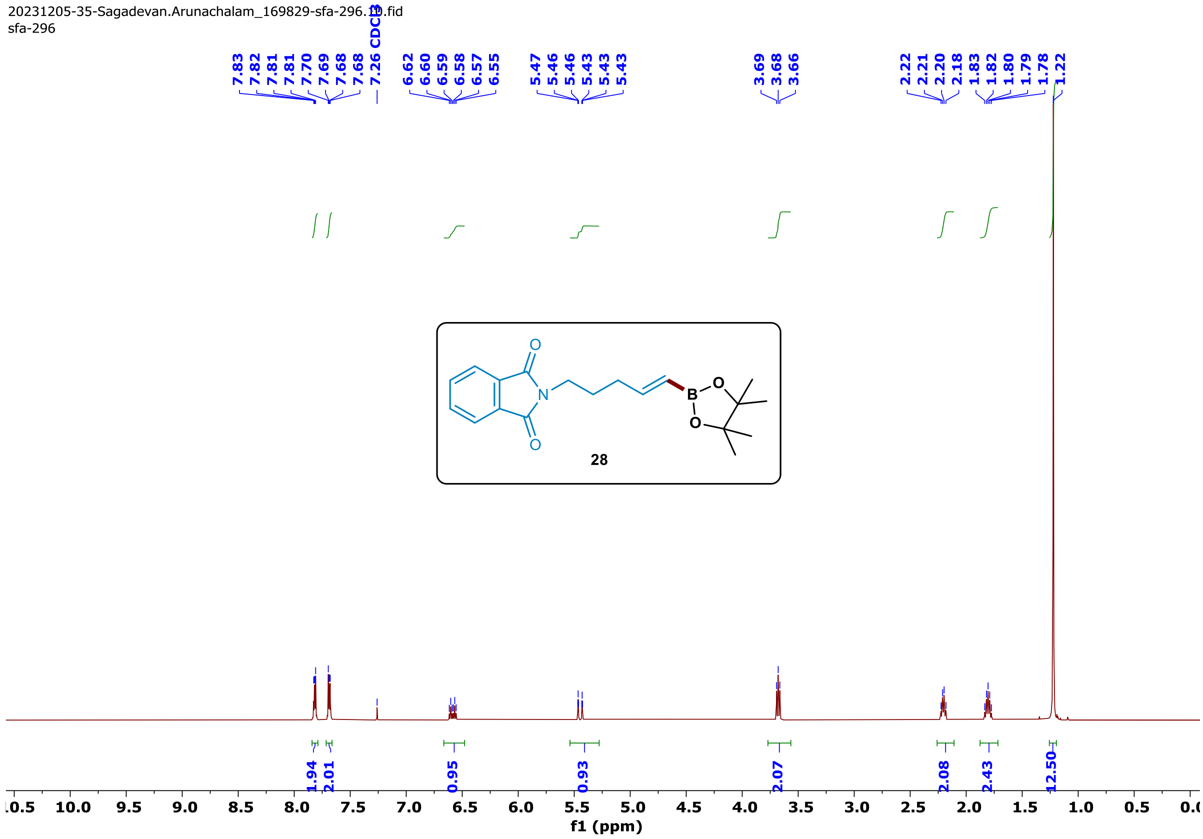
20231205-35-Sagadevan.Arunachalam_169829-sfa-296.10.fid
sfa-296
7.83 7.82 7.81 7.81 7.70 7.69 7.68 7.68 7.26 CDCl3 6.62 6.60 6.59 6.58 6.57 6.55 5.47 5.46 5.46 5.43 5.43 5.43 3.69 3.68 3.66 2.22 2.21 2.20 2.18 1.83 1.82 1.80 1.79 1.78 1.22
28
1.94 2.01 0.95 0.93 2.07 2.08 2.43 12.50
f1 (ppm)

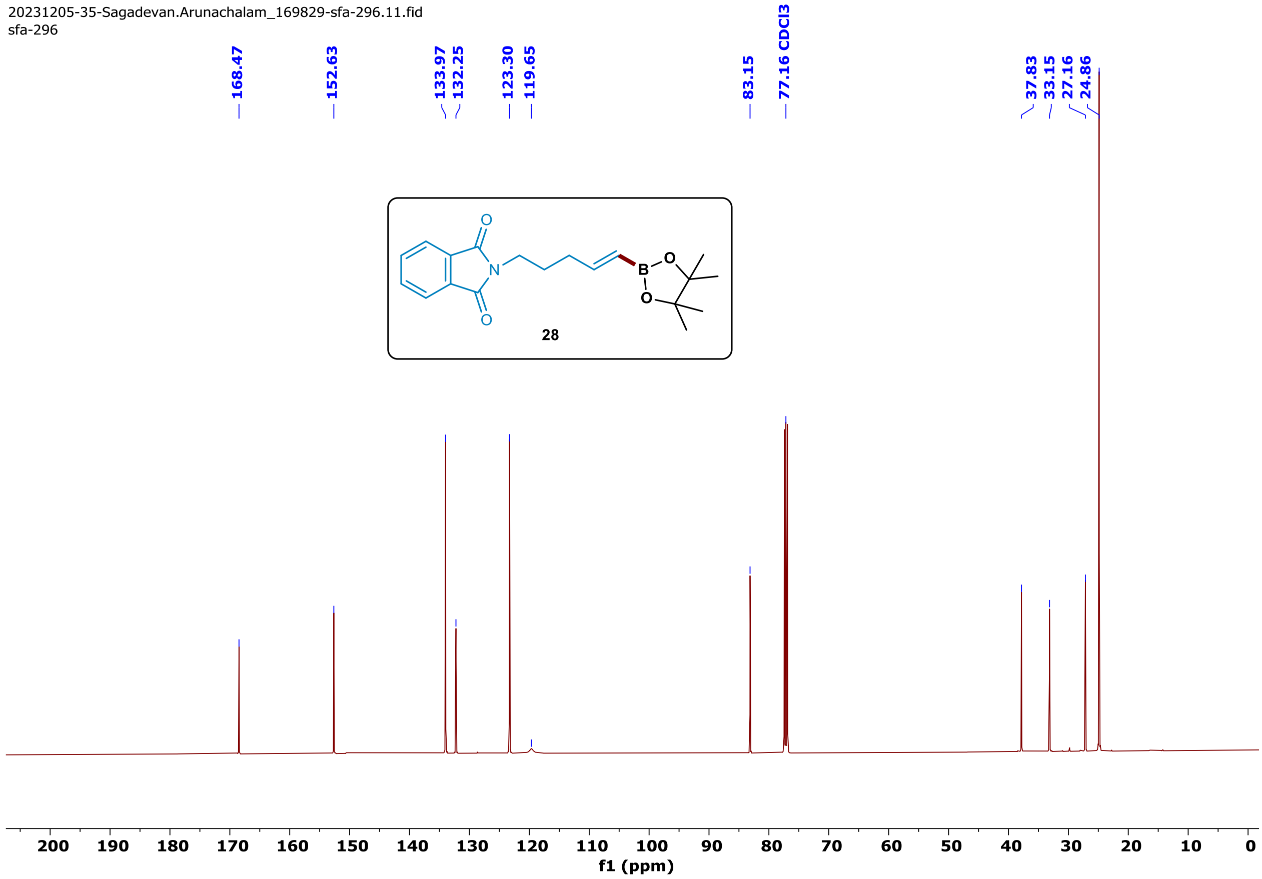
20231205-35-Sagadevan.Arunachalam_169829-sfa-296.11.fid
sfa-296
168.47 152.63 133.97 132.25 123.30 119.65 83.15 77.16 CDCl3 37.83 33.15 27.16 24.86
28
f1 (ppm)

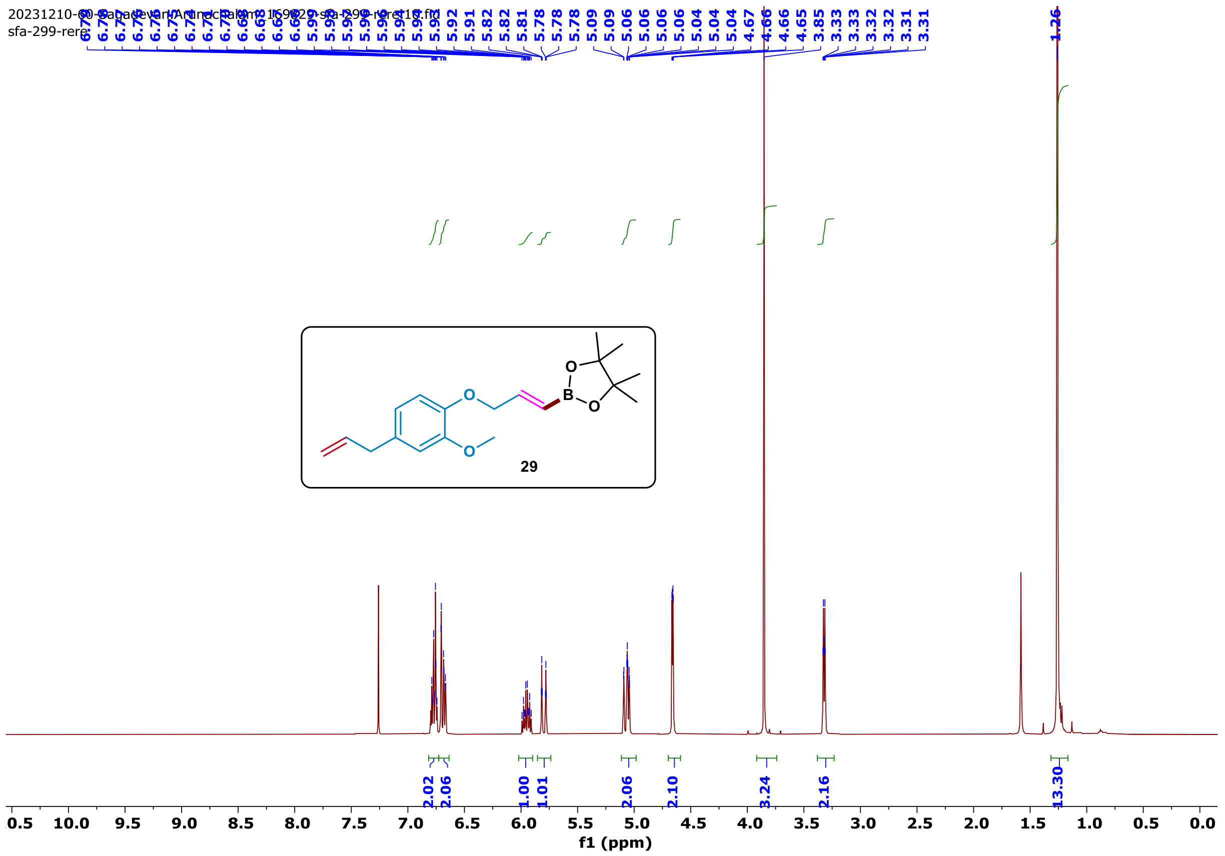
20231210-60-Sagadevan.Arunachalam_169829-sfa-299-rere.10.fid
sfa-299-rere
1.26
29
2.02
2.06
1.00
1.01
2.06
2.10
3.24
2.16
13.30
f1 (ppm)

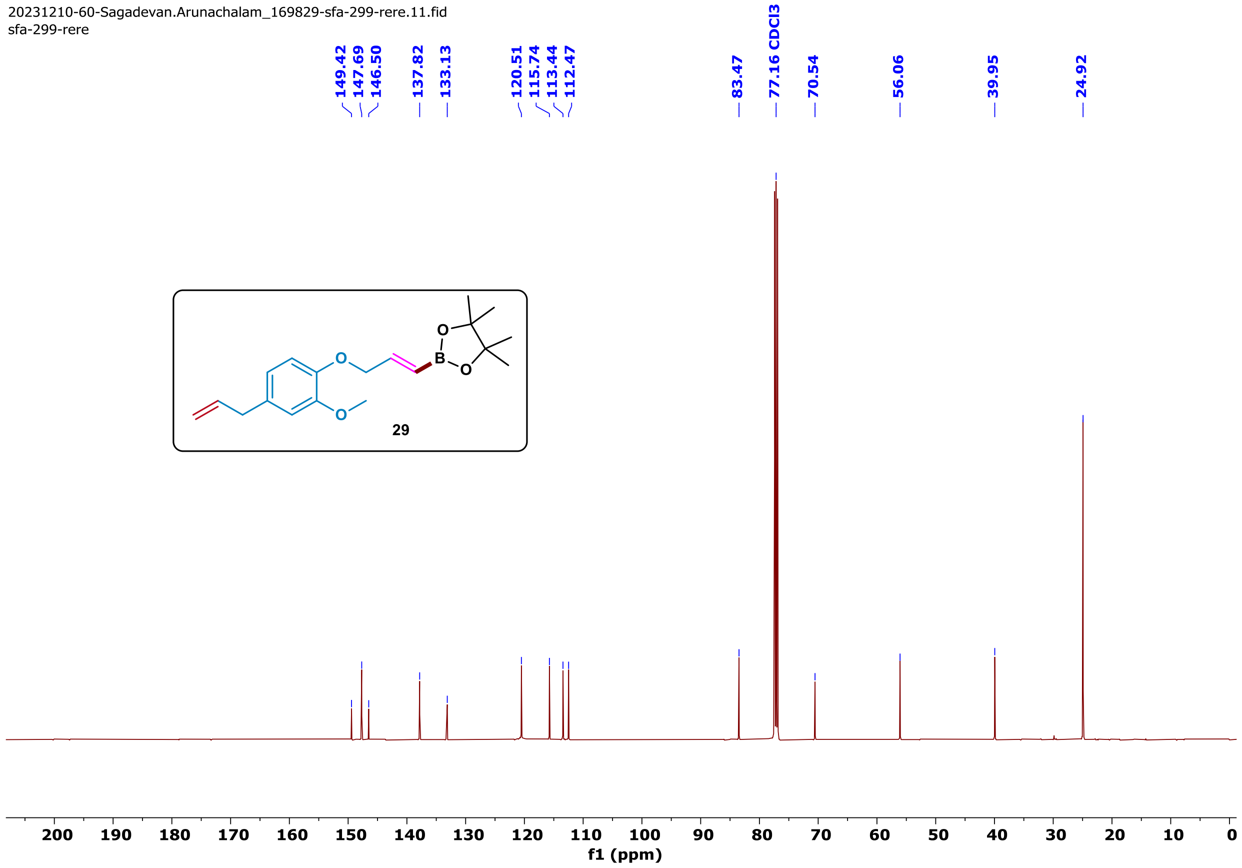
20231210-60-Sagadevan.Arunachalam_169829-sfa-299-rere.11.fid
sfa-299-rere
149.42
147.69
146.50
137.82
133.13
120.51
115.74
113.44
112.47
83.47
77.16 CDCl3
70.54
56.06
39.95
24.92
29
f1 (ppm)

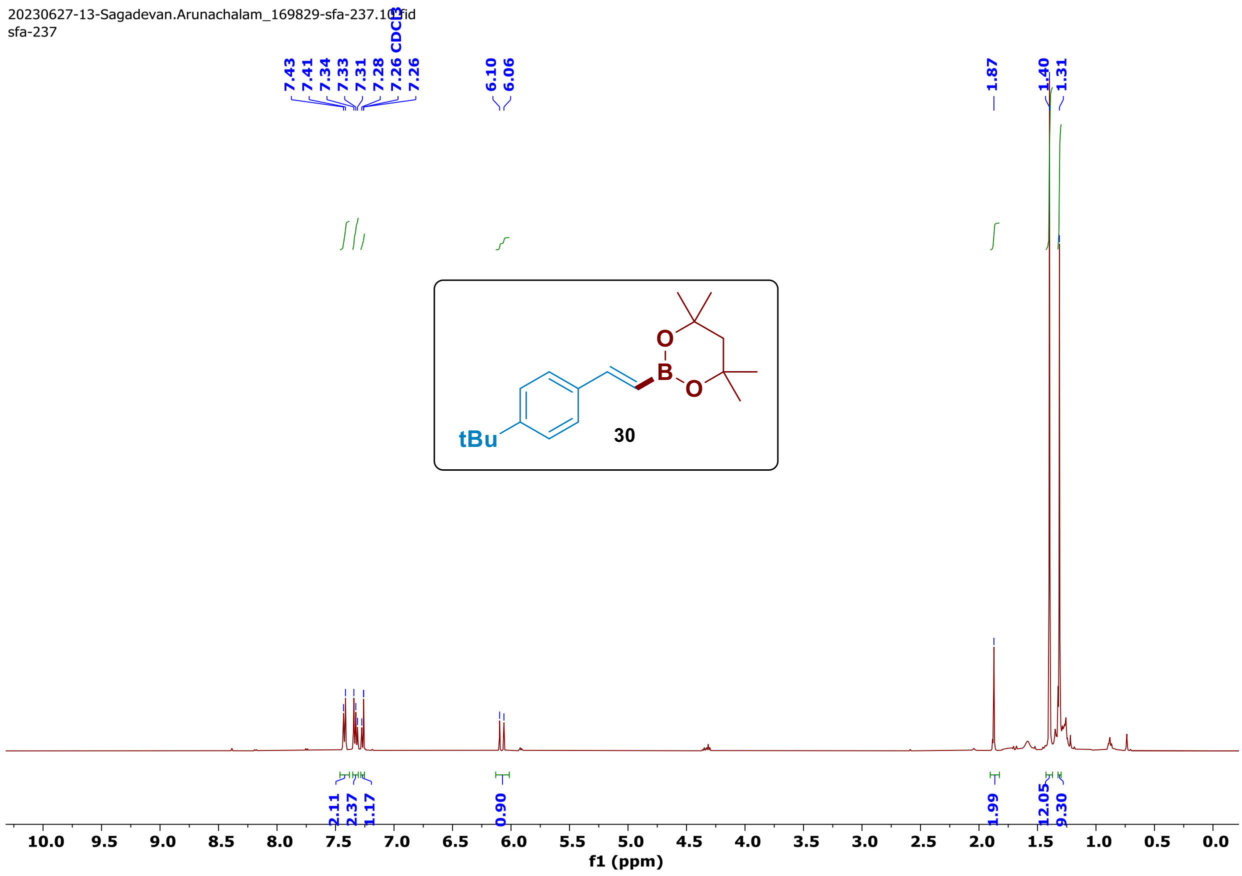
20230627-13-Sagadevan.Arunachalam_169829-sfa-237.10.fid
sfa-237
7.43 7.41 7.34 7.33 7.31 7.28 7.26 CDCl3 7.26
6.10 6.06
1.87
1.40 1.31
tBu
30
2.11 2.37 1.17
0.90
1.99
12.05 9.30
10.0 9.5 9.0 8.5 8.0 7.5 7.0 6.5 6.0 5.5 5.0 4.5 4.0 3.5 3.0 2.5 2.0 1.5 1.0 0.5 0.0
f1 (ppm)

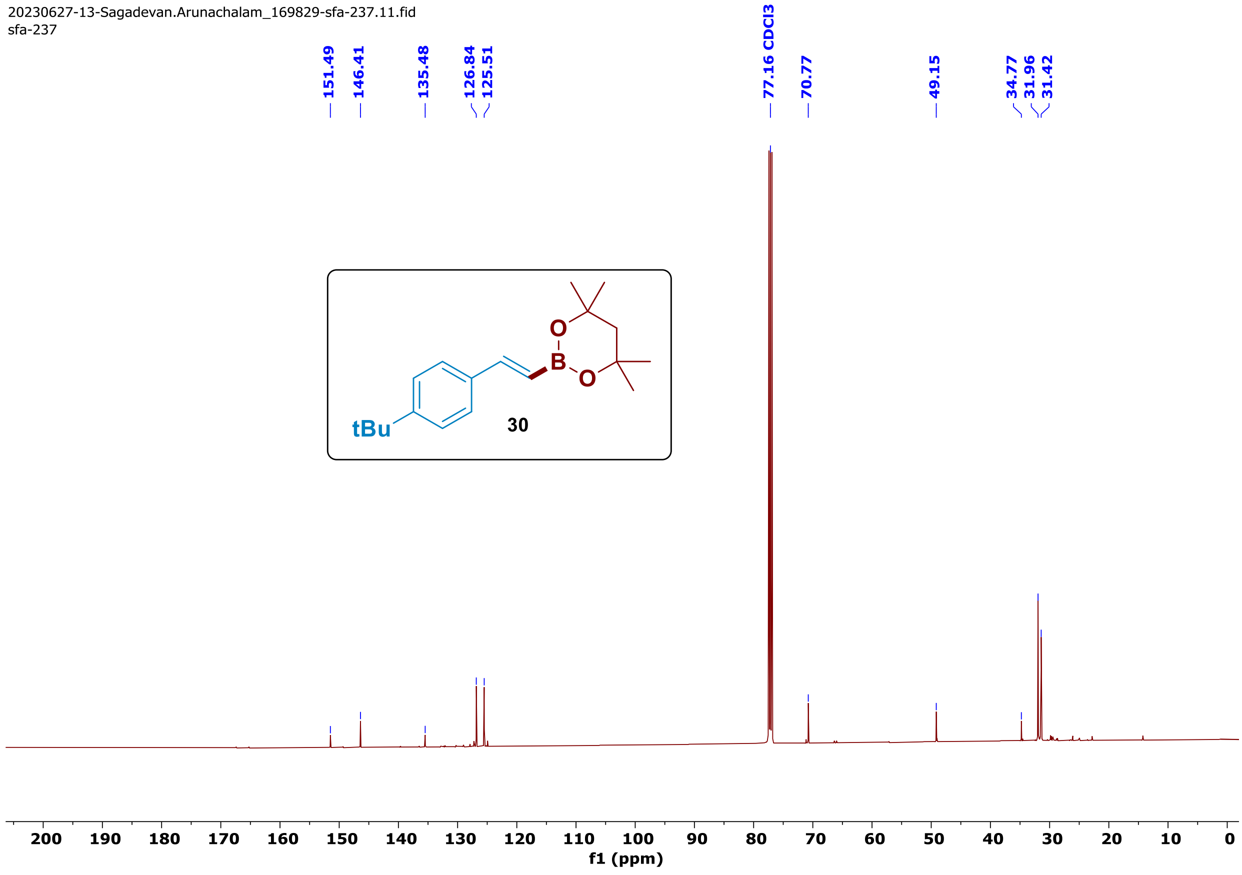
20230627-13-Sagadevan.Arunachalam_169829-sfa-237.11.fid
sfa-237
151.49
146.41
135.48
126.84 125.51
77.16 CDCl3
70.77
49.15
34.77 31.96 31.42
tBu
30
200 190 180 170 160 150 140 130 120 110 100 90 80 70 60 50 40 30 20 10 0
f1 (ppm)

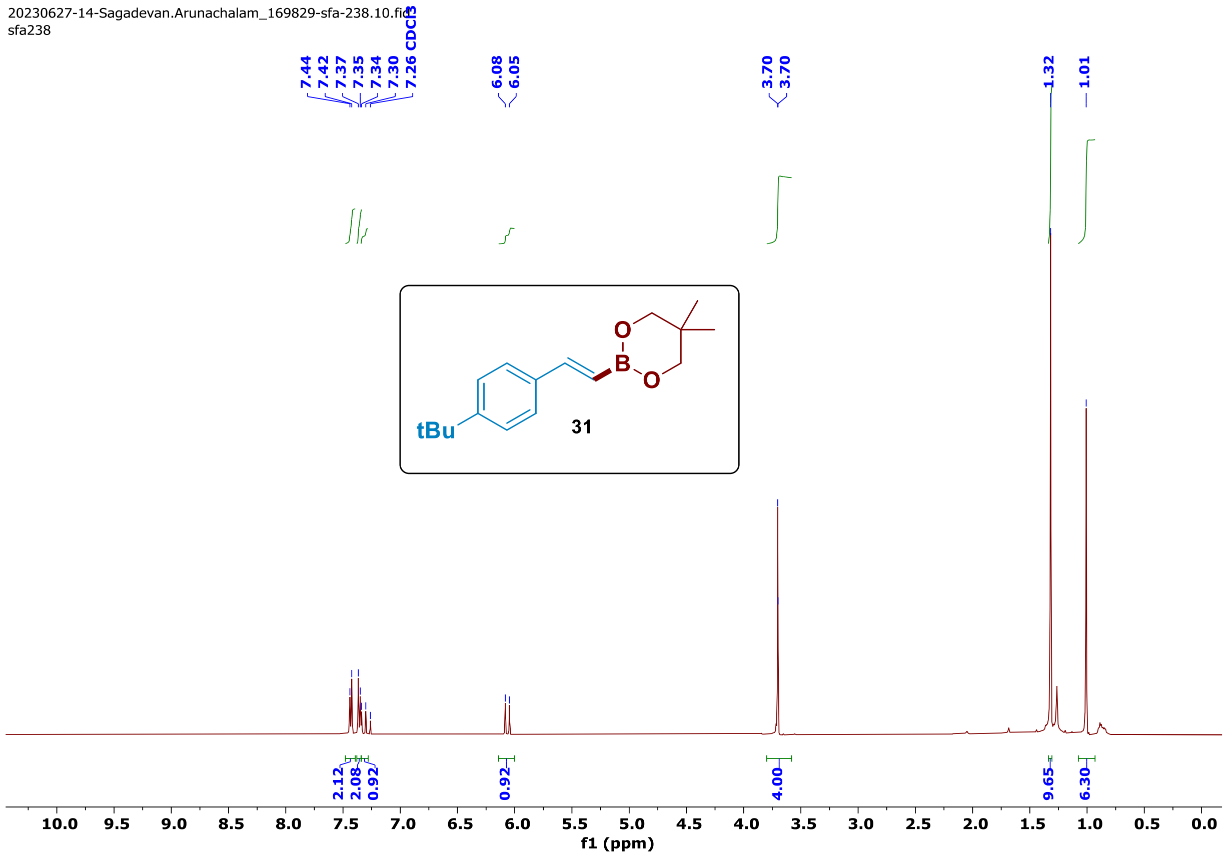
20230627-14-Sagadevan.Arunachalam_169829-sfa-238.10.fid
sfa238
7.44
7.42
7.37
7.35
7.34
7.30
7.26 CDCl3
6.08
6.05
3.70
3.70
1.32
1.01
tBu
31
2.12
2.08
0.92
0.92
4.00
9.65
6.30
f1 (ppm)

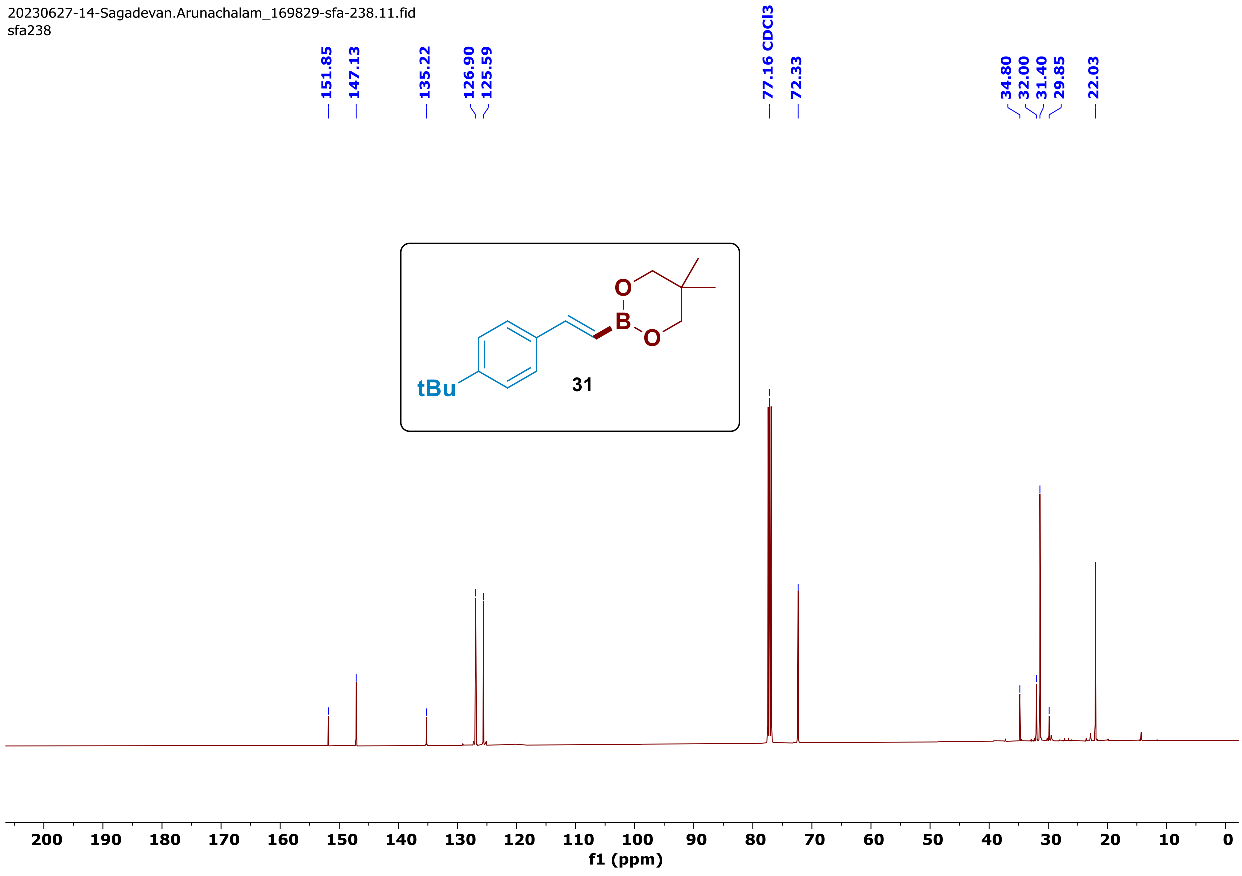
20230627-14-Sagadevan.Arunachalam_169829-sfa-238.11.fid
sfa238
151.85
147.13
135.22
126.90
125.59
77.16 CDCl3
72.33
34.80
32.00
31.40
29.85
22.03
tBu
31
f1 (ppm)

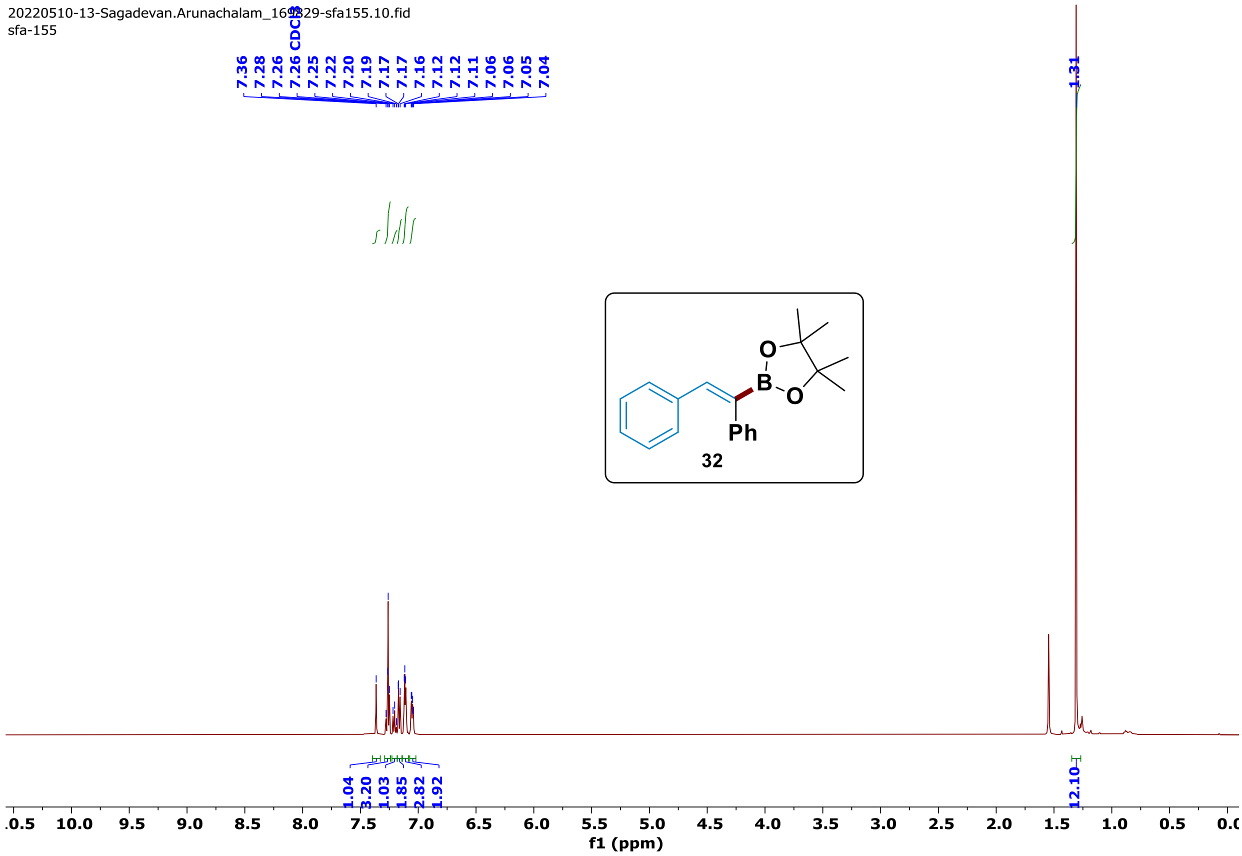
20220510-13-Sagadevan.Arunachalam_169829-sfa155.10.fid
sfa-155
7.36
7.28
7.26
7.26 CDCl3
7.25
7.22
7.20
7.19
7.17
7.17
7.16
7.12
7.12
7.11
7.06
7.06
7.05
7.04
1.31
O
B
O
Ph
32
1.04
3.20
1.03
1.85
2.82
1.92
12.10
f1 (ppm)

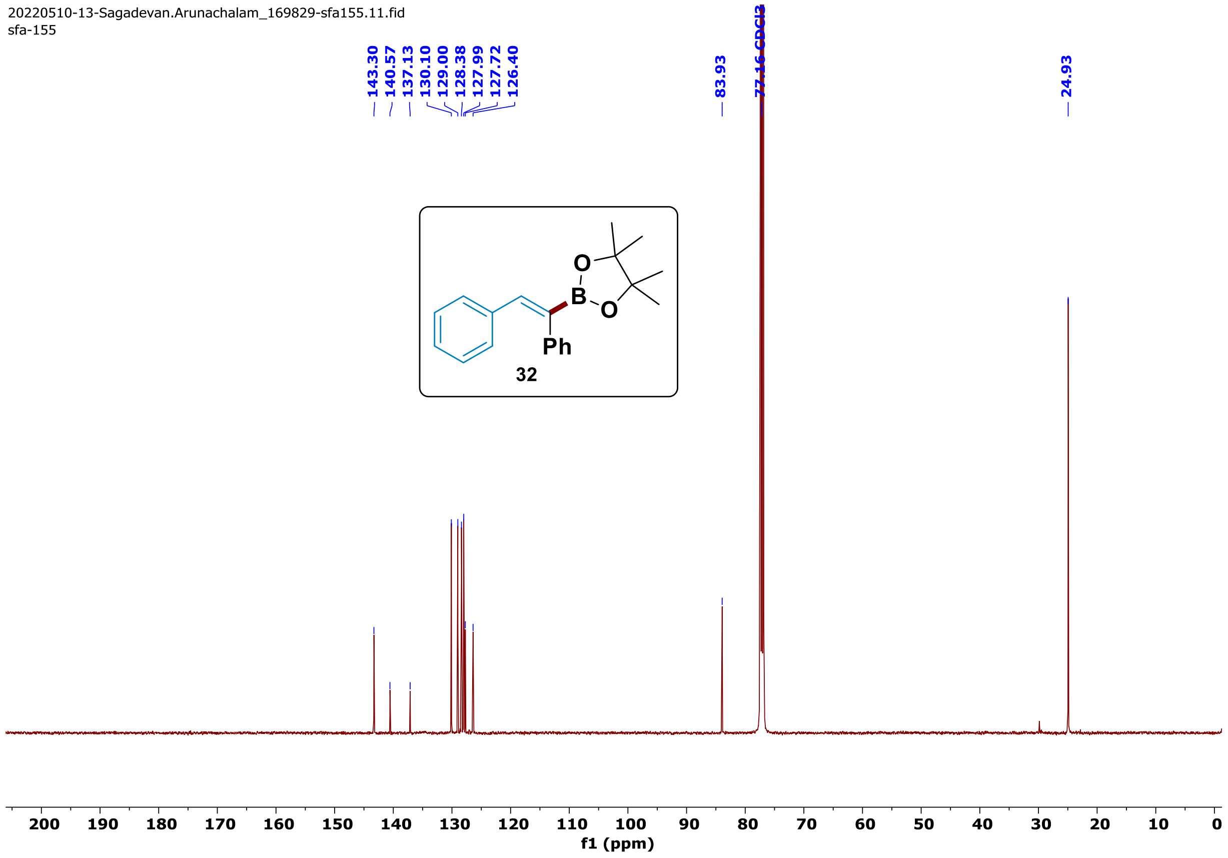
20220510-13-Sagadevan.Arunachalam_169829-sfa155.11.fid
sfa-155
143.30
140.57
137.13
130.10
129.00
128.38
127.99
127.72
126.40
83.93
77.16 CDCl3
24.93
O
B
O
Ph
32
f1 (ppm)

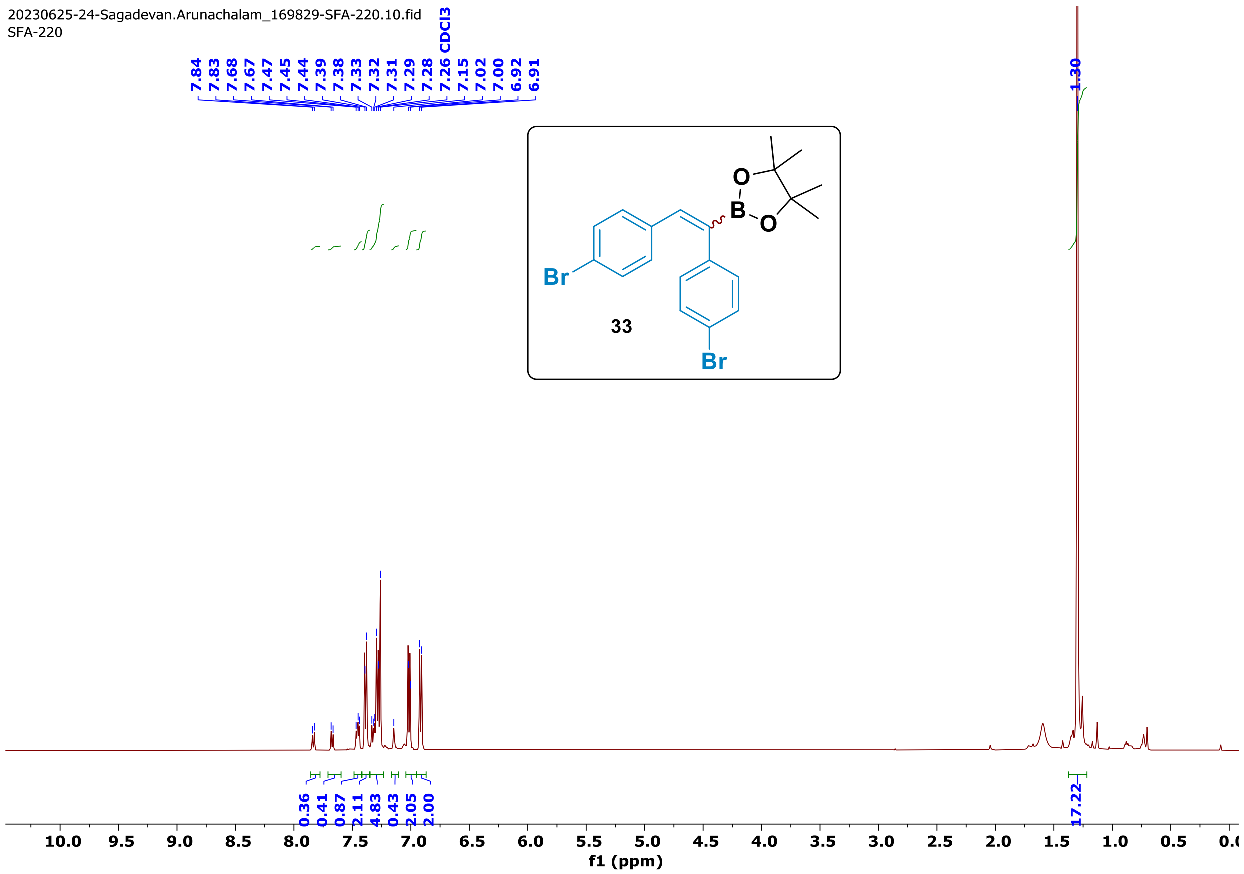
20230625-24-Sagadevan.Arunachalam_169829-SFA-220.10.fid
SFA-220
7.84
7.83
7.68
7.67
7.47
7.45
7.44
7.39
7.38
7.33
7.32
7.31
7.29
7.28
7.26 CDCl3
7.15
7.02
7.00
6.92
6.91
1.30
O
B
O
Br
Br
33
0.36
0.41
0.87
2.11
4.83
0.43
2.05
2.00
17.22
10.0 9.5 9.0 8.5 8.0 7.5 7.0 6.5 6.0 5.5 5.0 4.5 4.0 3.5 3.0 2.5 2.0 1.5 1.0 0.5
f1 (ppm)

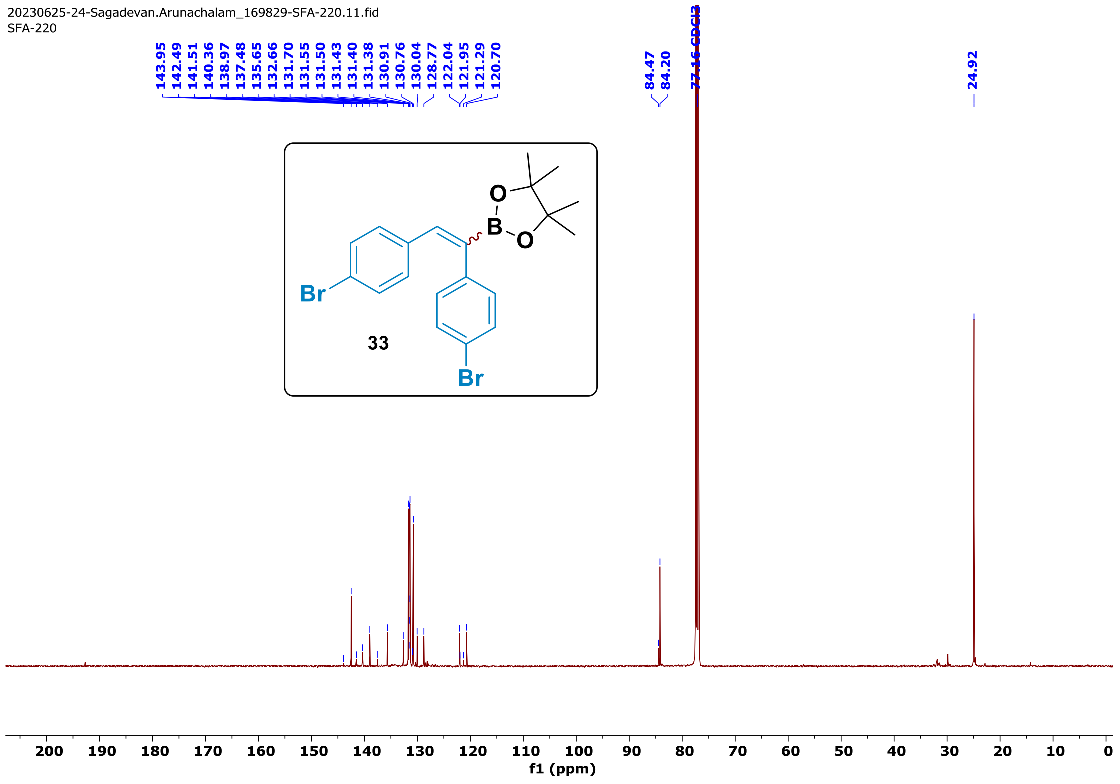
20230625-24-Sagadevan.Arunachalam_169829-SFA-220.11.fid
SFA-220
143.95
142.49
141.51
140.36
138.97
137.48
135.65
132.66
131.70
131.55
131.50
131.43
131.40
131.38
130.91
130.76
130.04
128.77
122.04
121.95
121.29
120.70
84.47
84.20
77.16 CDCl3
24.92
O
B
O
Br
Br
33
200 190 180 170 160 150 140 130 120 110 100 90 80 70 60 50 40 30 20 10 0
f1 (ppm)

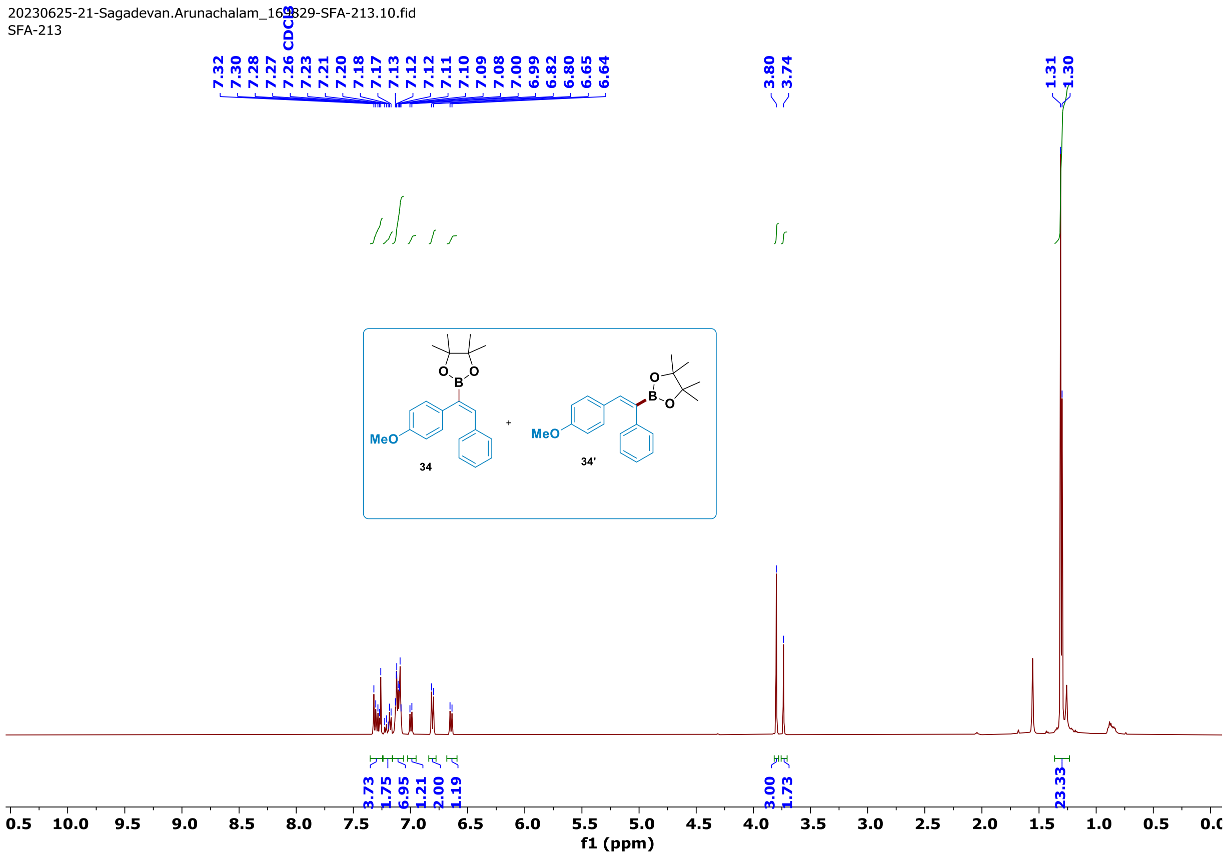
20230625-21-Sagadevan.Arunachalam_169829-SFA-213.10.fid
SFA-213
7.32 7.30 7.28 7.27 7.26 CDCl3 7.23 7.21 7.20 7.18 7.17 7.13 7.12 7.12 7.11 7.10 7.09 7.08 7.00 6.99 6.82 6.80 6.65 6.64
3.80 3.74
1.31 1.30
MeO
34
34'
3.73 1.75 6.95 1.21 2.00 1.19
3.00 1.73
23.33
f1 (ppm)

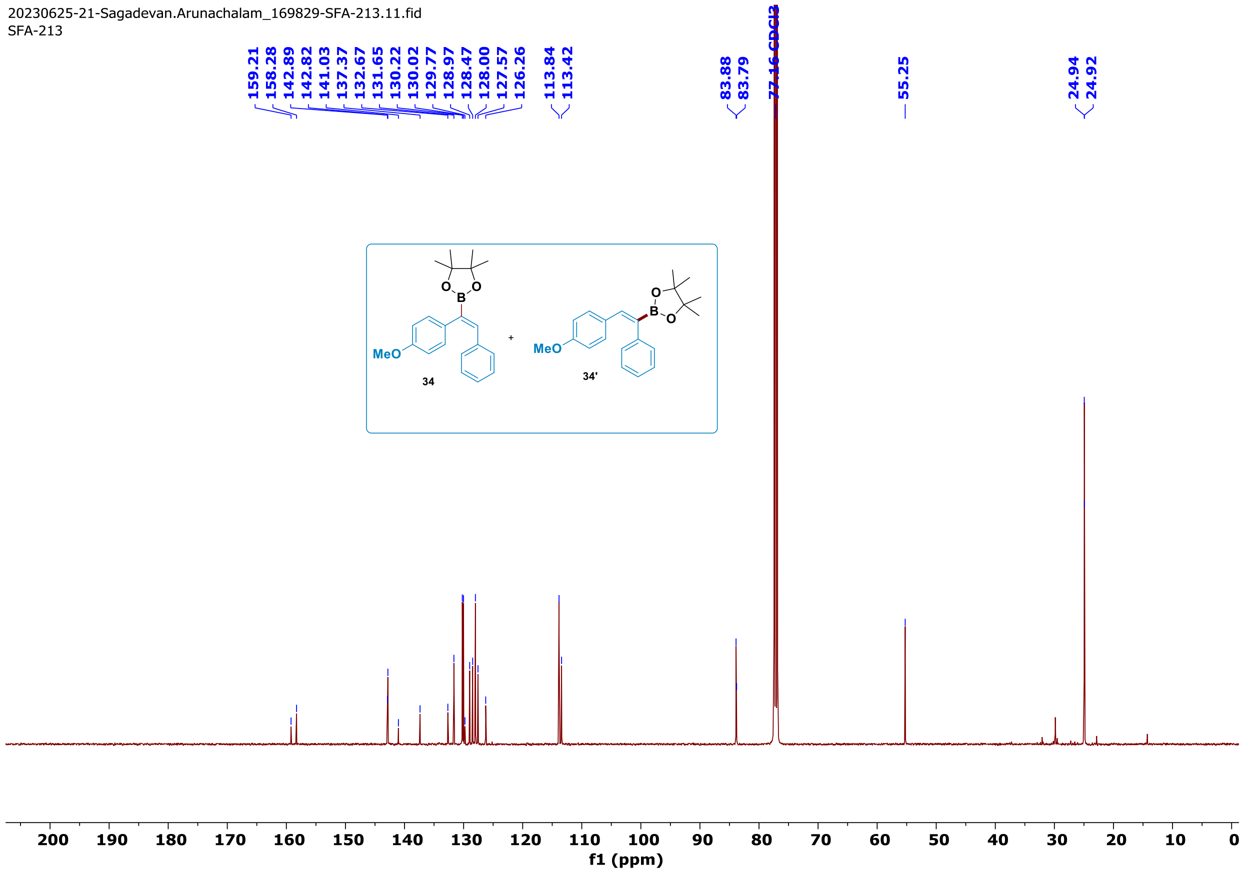
20230625-21-Sagadevan.Arunachalam_169829-SFA-213.11.fid
SFA-213
159.21 158.28 142.89 142.82 141.03 137.37 132.67 131.65 130.22 130.02 129.77 128.97 128.47 128.00 127.57 126.26
113.84 113.42
83.88 83.79
77.16 CDCl3
55.25
24.94 24.92
MeO
34
34'
f1 (ppm)

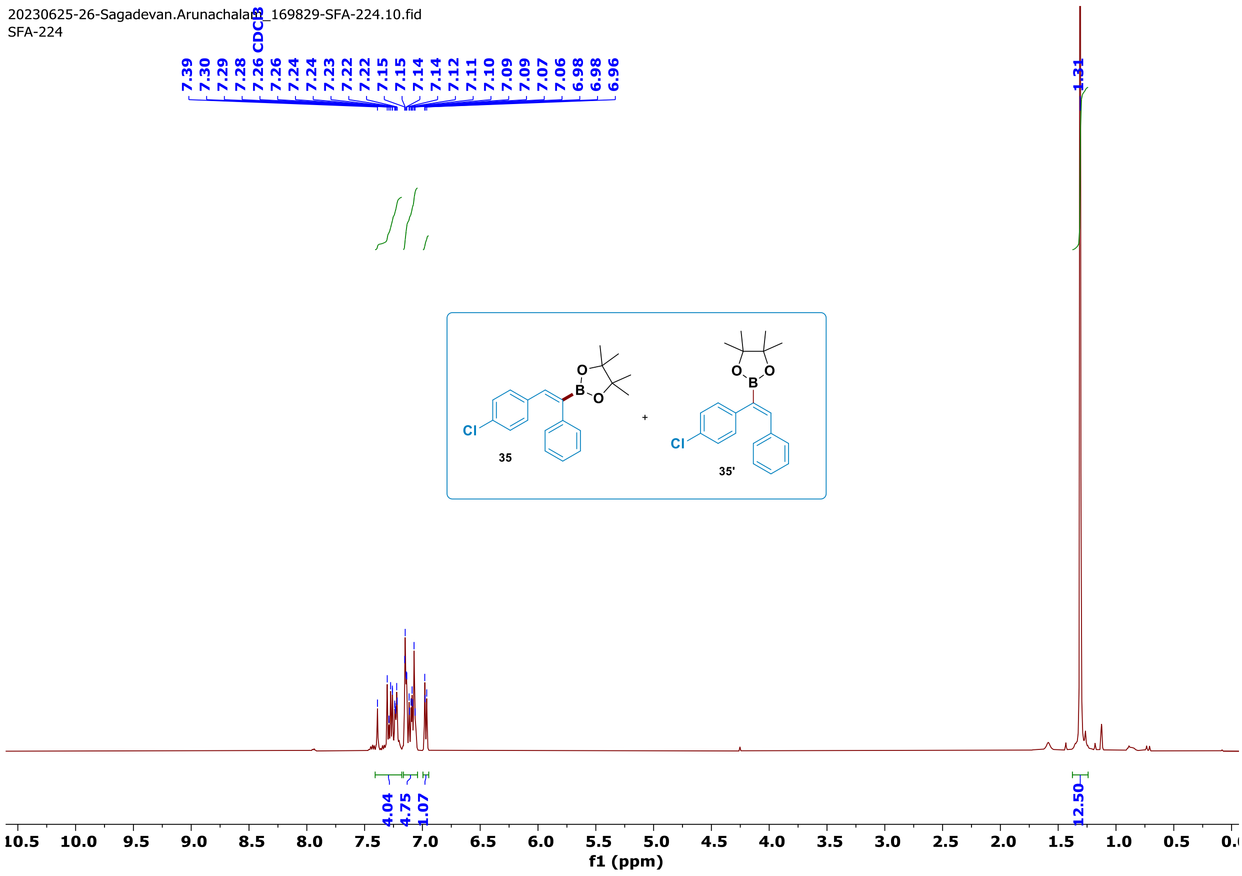
20230625-26-Sagadevan.Arunachalam_169829-SFA-224.10.fid
SFA-224
7.39 7.30 7.29 7.28 7.26 CDCl3 7.26 7.24 7.24 7.23 7.22 7.22 7.15 7.15 7.14 7.14 7.12 7.11 7.10 7.09 7.09 7.07 7.06 6.98 6.98 6.96
1.31
Cl
35
35'
4.04
4.75
1.07
12.50
10.5 10.0 9.5 9.0 8.5 8.0 7.5 7.0 6.5 6.0 5.5 5.0 4.5 4.0 3.5 3.0 2.5 2.0 1.5 1.0 0.5
f1 (ppm)

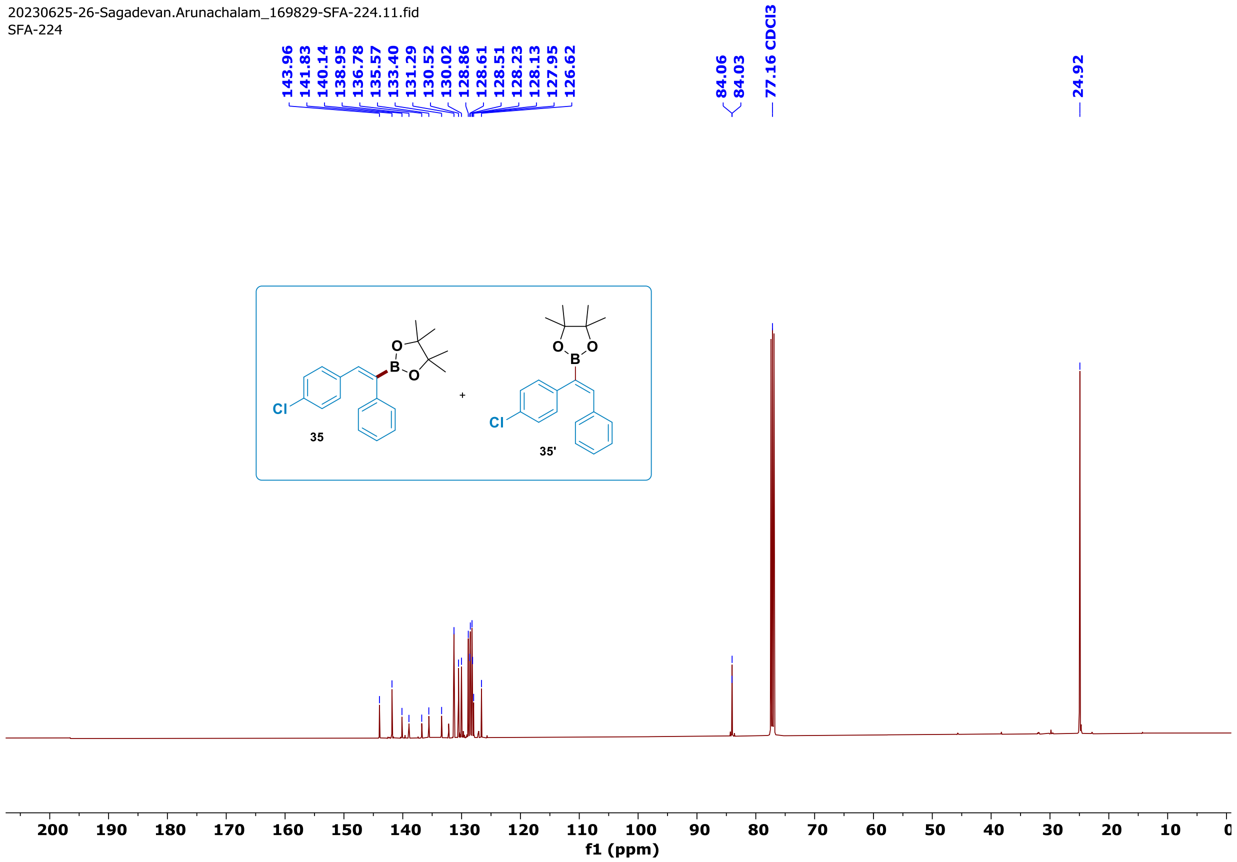
20230625-26-Sagadevan.Arunachalam_169829-SFA-224.11.fid
SFA-224
143.96 141.83 140.14 138.95 136.78 135.57 133.40 131.29 130.52 130.02 128.86 128.61 128.51 128.23 128.13 127.95 126.62
84.06 84.03
77.16 CDCl3
24.92
Cl
35
35'
200 190 180 170 160 150 140 130 120 110 100 90 80 70 60 50 40 30 20 10
f1 (ppm)

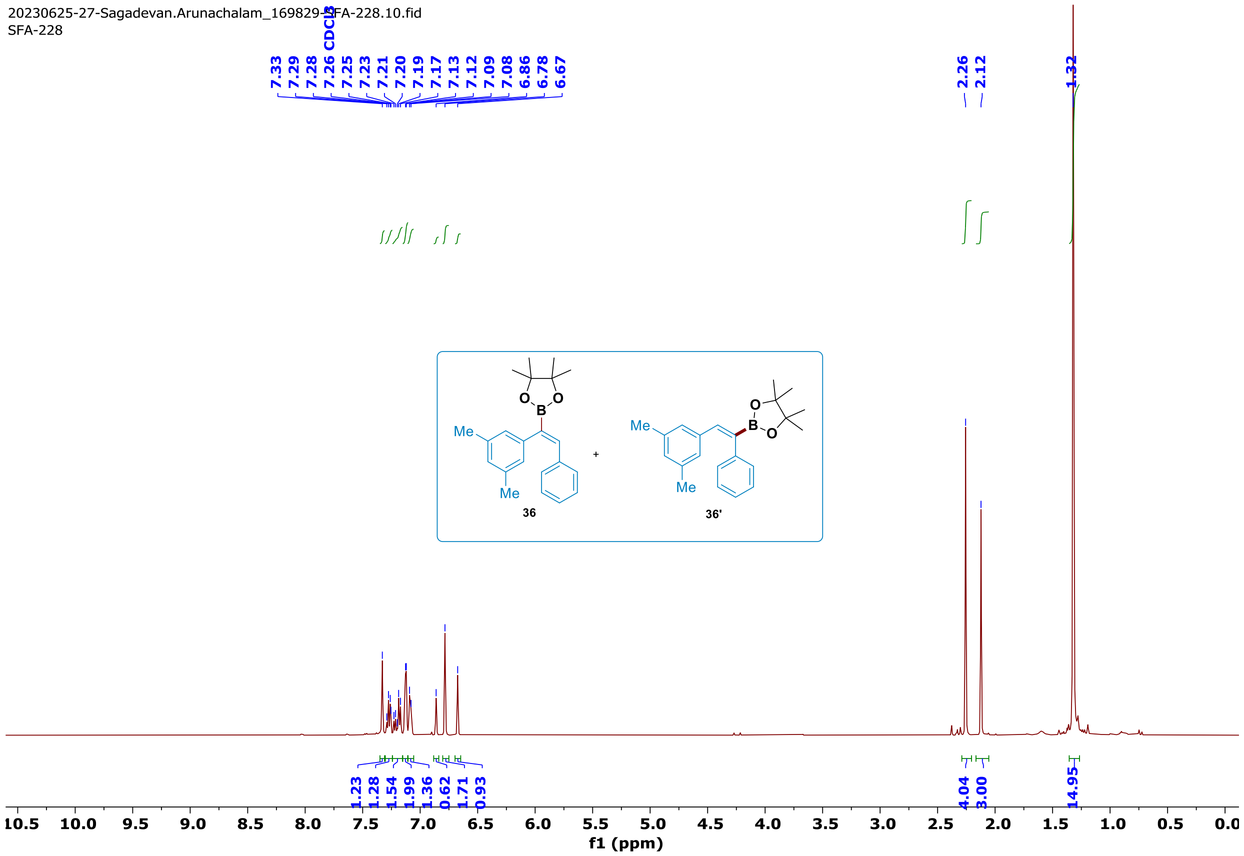
20230625-27-Sagadevan.Arunachalam_169829-SFA-228.10.fid
SFA-228
7.33 7.29 7.28 7.26 CDCl3 7.25 7.23 7.21 7.20 7.19 7.17 7.13 7.12 7.09 7.08 6.86 6.78 6.67
2.26 2.12
1.32
Me
Me
36
+
Me
Me
36'
1.23 1.28 1.54 1.99 1.36 0.62 1.71 0.93
4.04 3.00
14.95
f1 (ppm)

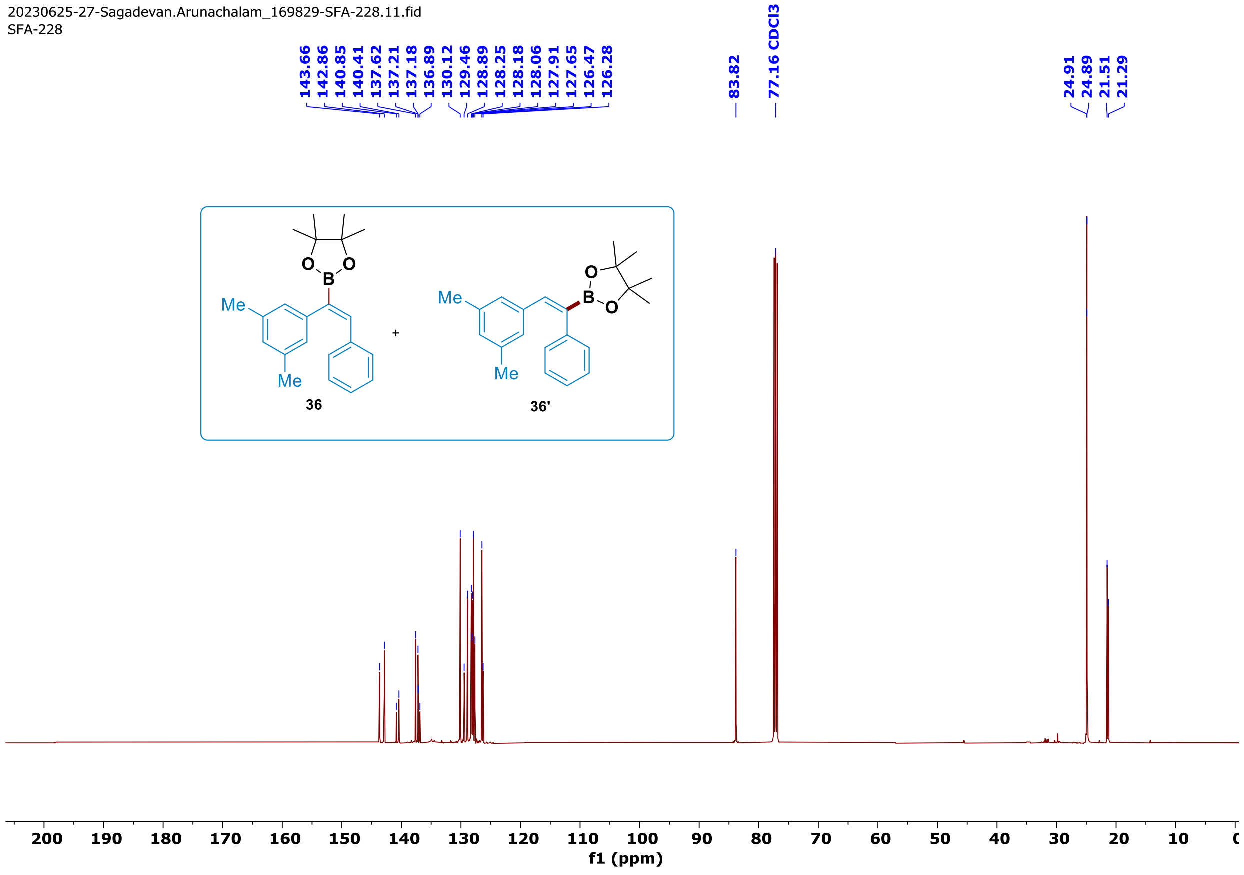
20230625-27-Sagadevan.Arunachalam_169829-SFA-228.11.fid
SFA-228
143.66 142.86 140.85 140.41 137.62 137.21 137.18 136.89 130.12 129.46 128.89 128.25 128.18 128.06 127.91 127.65 126.47 126.28
83.82
77.16 CDCl3
24.91 24.89 21.51 21.29
Me
Me
36
+
Me
Me
36'
f1 (ppm)

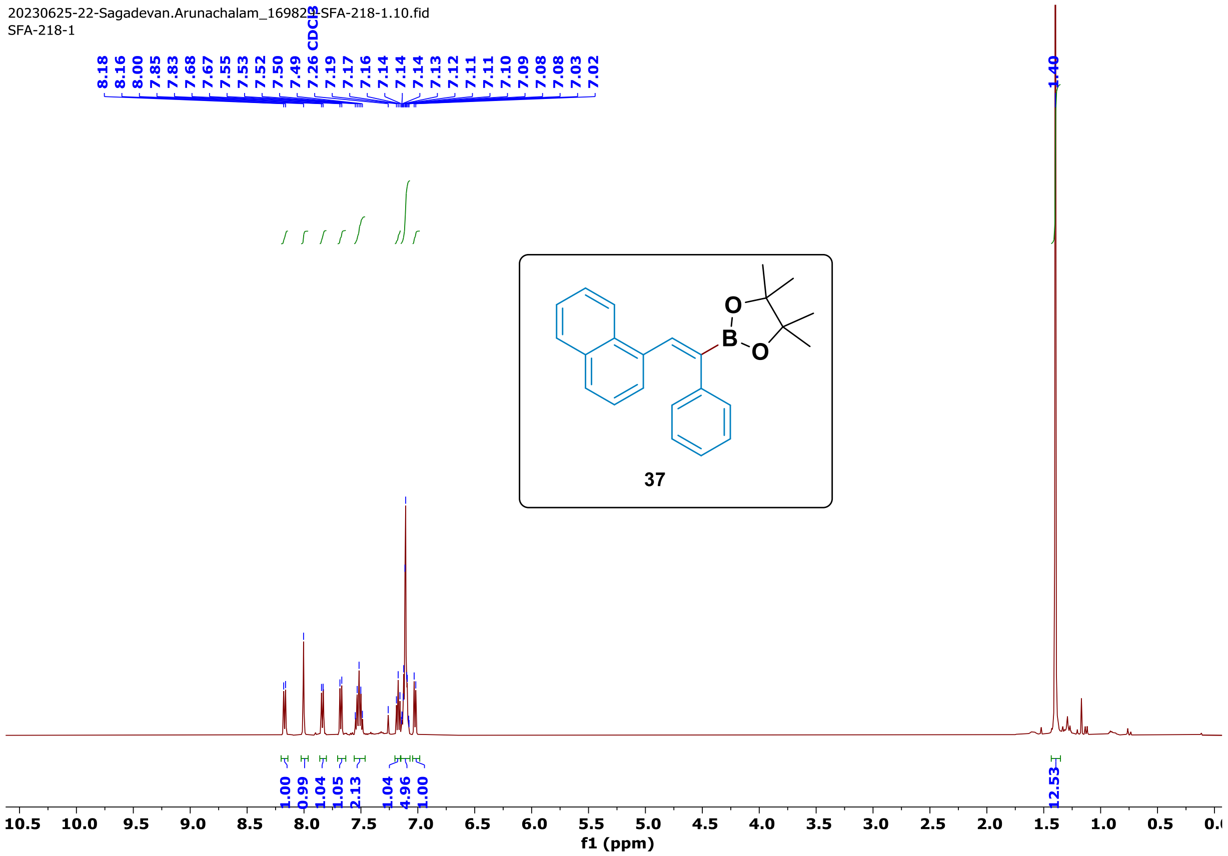
20230625-22-Sagadevan.Arunachalam_169829-SFA-218-1.10.fid
SFA-218-1
8.18 8.16 8.00 7.85 7.83 7.68 7.67 7.55 7.53 7.52 7.50 7.49 7.26 CDCl3 7.19 7.17 7.16 7.14 7.14 7.14 7.13 7.12 7.11 7.11 7.10 7.09 7.08 7.08 7.03 7.02
1.40
1.00 0.99 1.04 1.05 2.13 1.04 4.96 1.00
12.53
37
f1 (ppm)

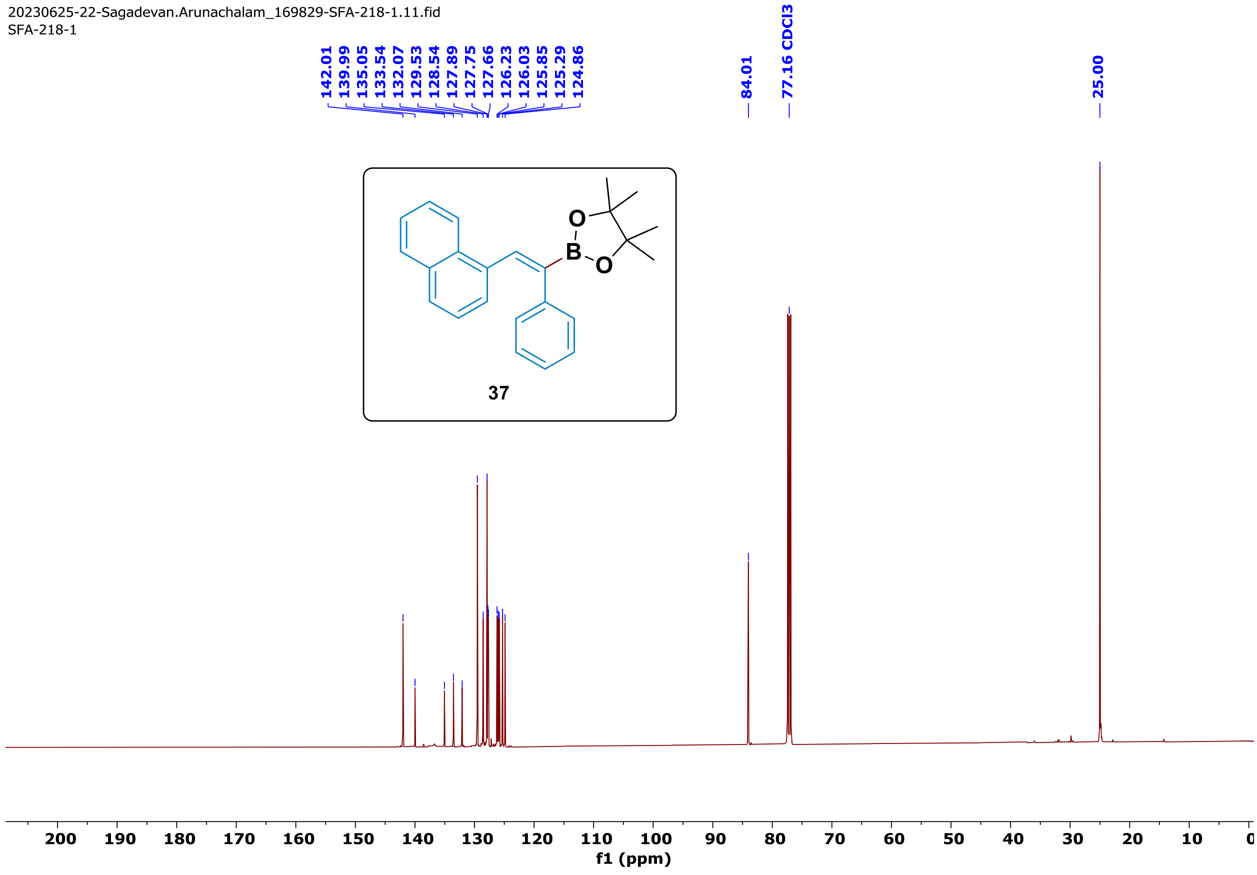
20230625-22-Sagadevan.Arunachalam_169829-SFA-218-1.11.fid
SFA-218-1
142.01 139.99 135.05 133.54 132.07 129.53 128.54 127.89 127.75 127.66 126.23 126.03 125.85 125.29 124.86
84.01
77.16 CDCl3
25.00
37
f1 (ppm)

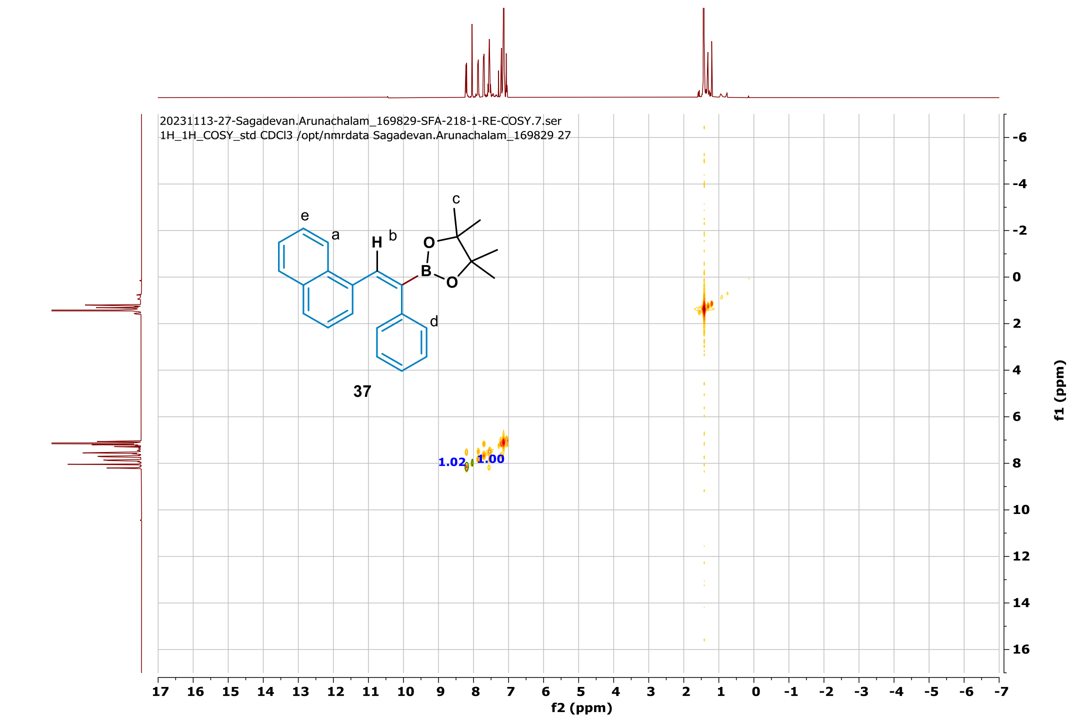
20231113-27-Sagadevan.Arunachalam_169829-SFA-218-1-RE-COSY.7.ser
1H_1H_COSY_std CDCl3 /opt/nmrdata Sagadevan.Arunachalam_169829 27
e
a
H
b
c
O
B
O
d
37
1.02
1.00
f1 (ppm)
f2 (ppm)

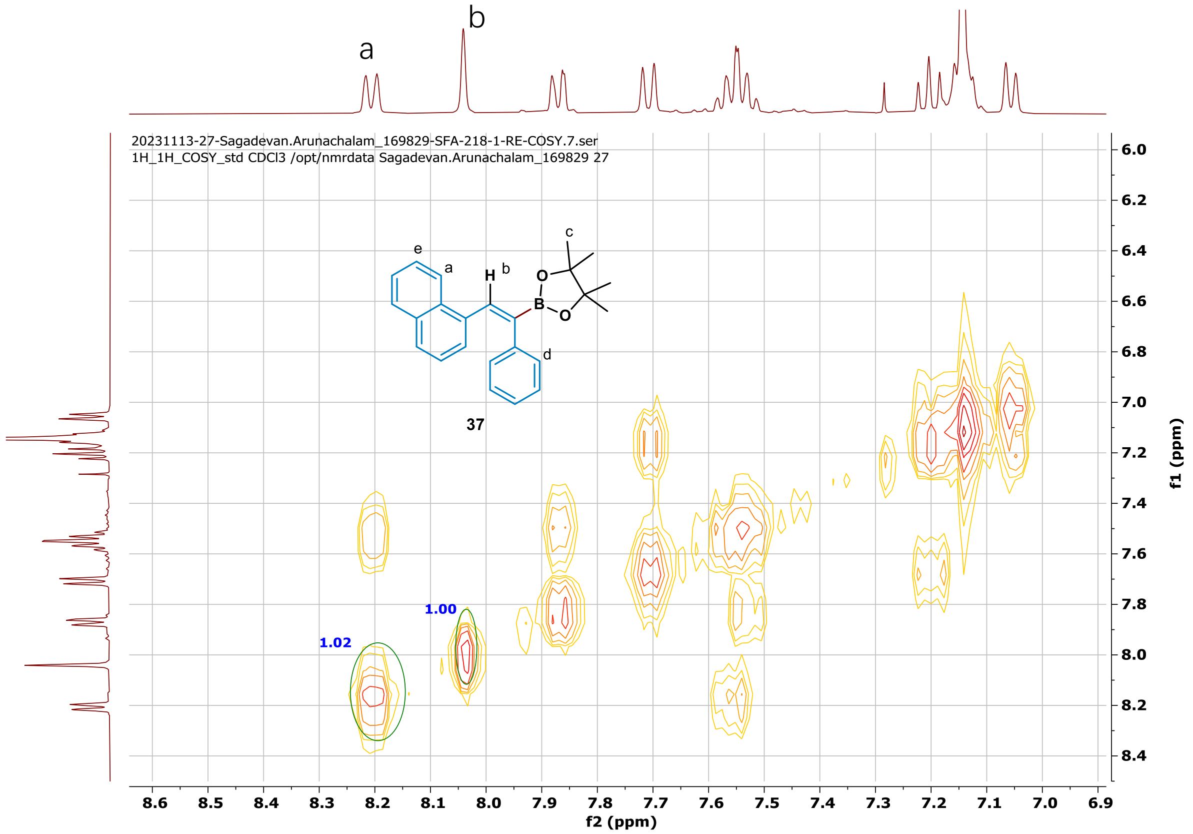
a
b
20231113-27-Sagadevan.Arunachalam_169829-SFA-218-1-RE-COSY.7.ser
1H_1H_COSY_std CDCl3 /opt/nmrdata Sagadevan.Arunachalam_169829 27
e
a
H
b
c
O
B
O
d
37
1.02
1.00
f1 (ppm)
f2 (ppm)

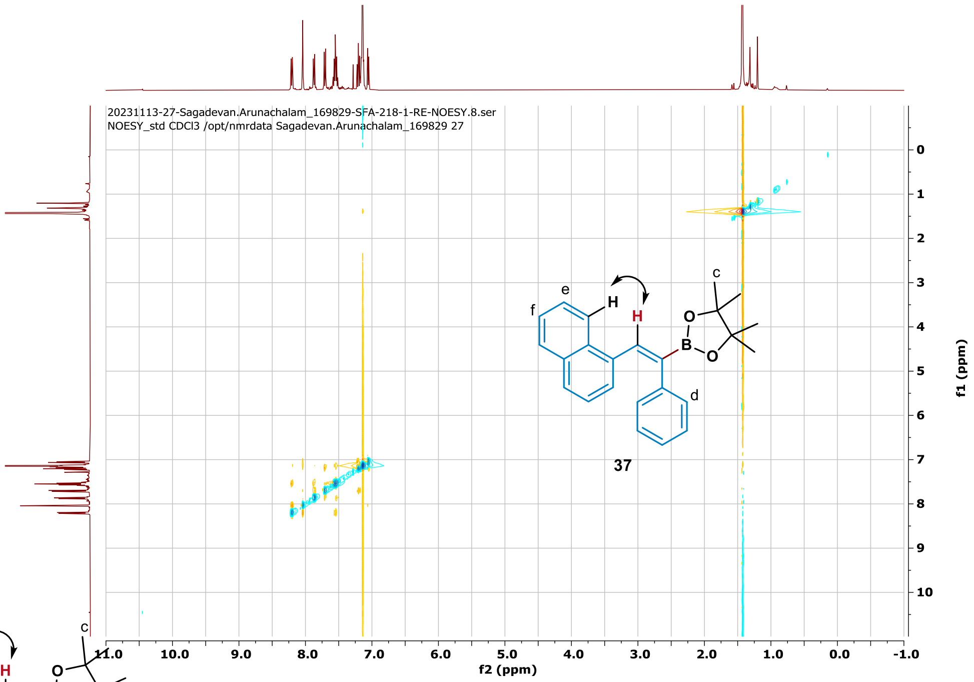
20231113-27-Sagadevan.Arunachalam_169829-SFA-218-1-RE-NOESY.8.ser
NOESY_std CDCl3 /opt/nmrdata Sagadevan.Arunachalam_169829 27
e
f
H
H
c
O
B
O
d
37
f2 (ppm)
f1 (ppm)

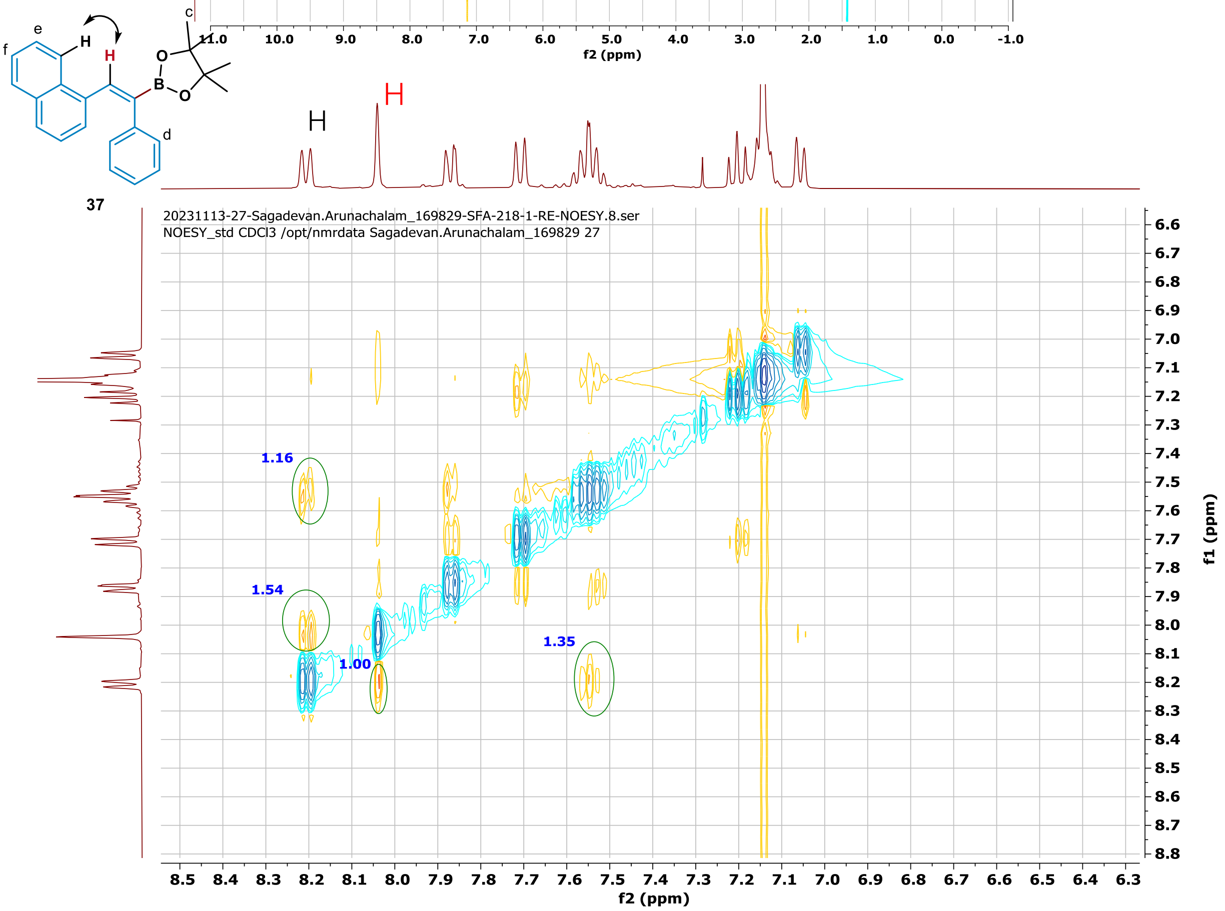
e
f
H
H
c
O
B
O
d
37
H
H
20231113-27-Sagadevan.Arunachalam_169829-SFA-218-1-RE-NOESY.8.ser
NOESY_std CDCl3 /opt/nmrdata Sagadevan.Arunachalam_169829 27
1.16
1.54
1.00
1.35
f2 (ppm)
f1 (ppm)

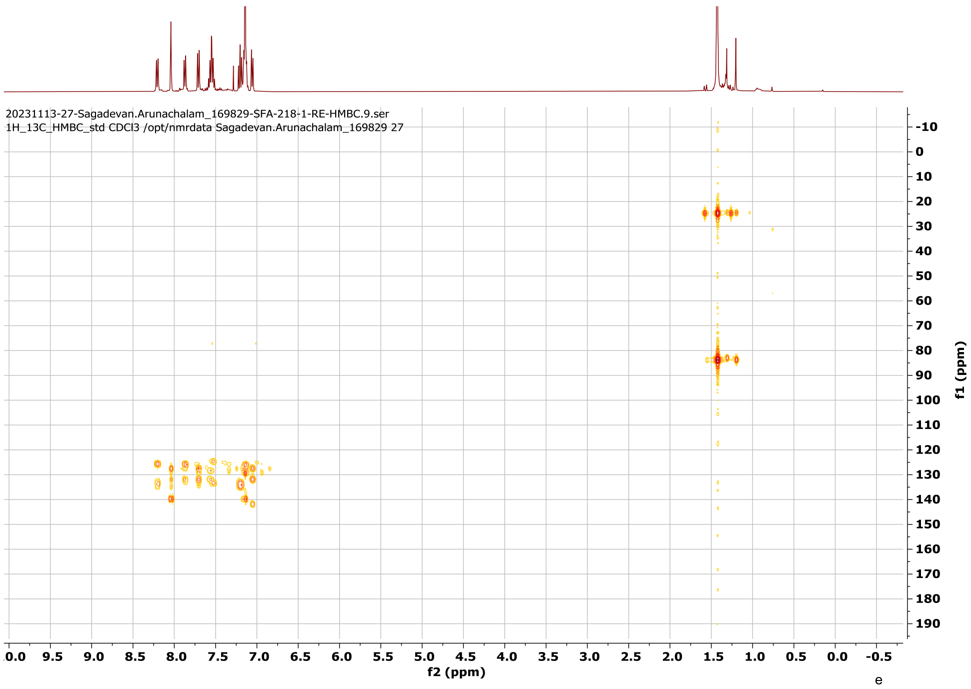
20231113-27-Sagadevan.Arunachalam_169829-SFA-218-1-RE-HMBC.9.ser
1H_13C_HMBC_std CDCl3 /opt/nmrdata Sagadevan.Arunachalam_169829 27
f1 (ppm)
f2 (ppm)

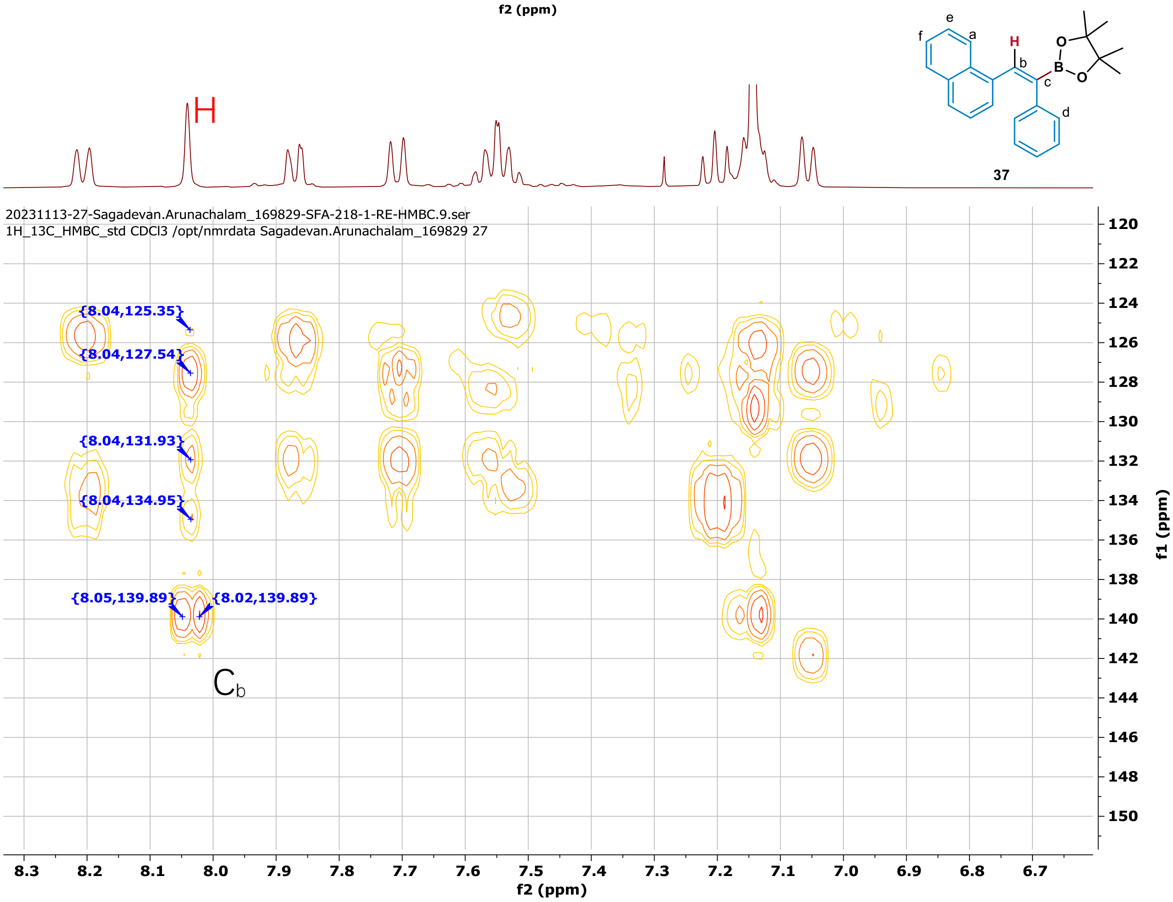
H
20231113-27-Sagadevan.Arunachalam_169829-SFA-218-1-RE-HMBC.9.ser
1H_13C_HMBC_std CDCl3 /opt/nmrdata Sagadevan.Arunachalam_169829 27
{8.04,125.35}
{8.04,127.54}
{8.04,131.93}
{8.04,134.95}
{8.05,139.89}
{8.02,139.89}
Cb
f1 (ppm)
f2 (ppm)
37

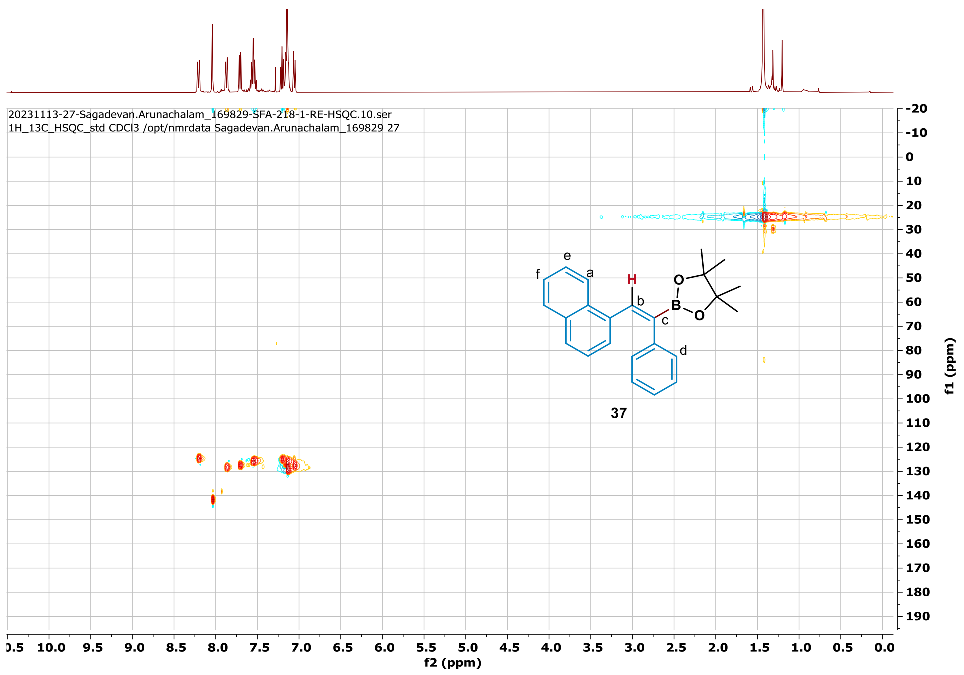
20231113-27-Sagadevan.Arunachalam_169829-SFA-218-1-RE-HSQC.10.ser
1H_13C_HSQC_std CDCl3 /opt/nmrdata Sagadevan.Arunachalam_169829 27
37
f2 (ppm)
f1 (ppm)

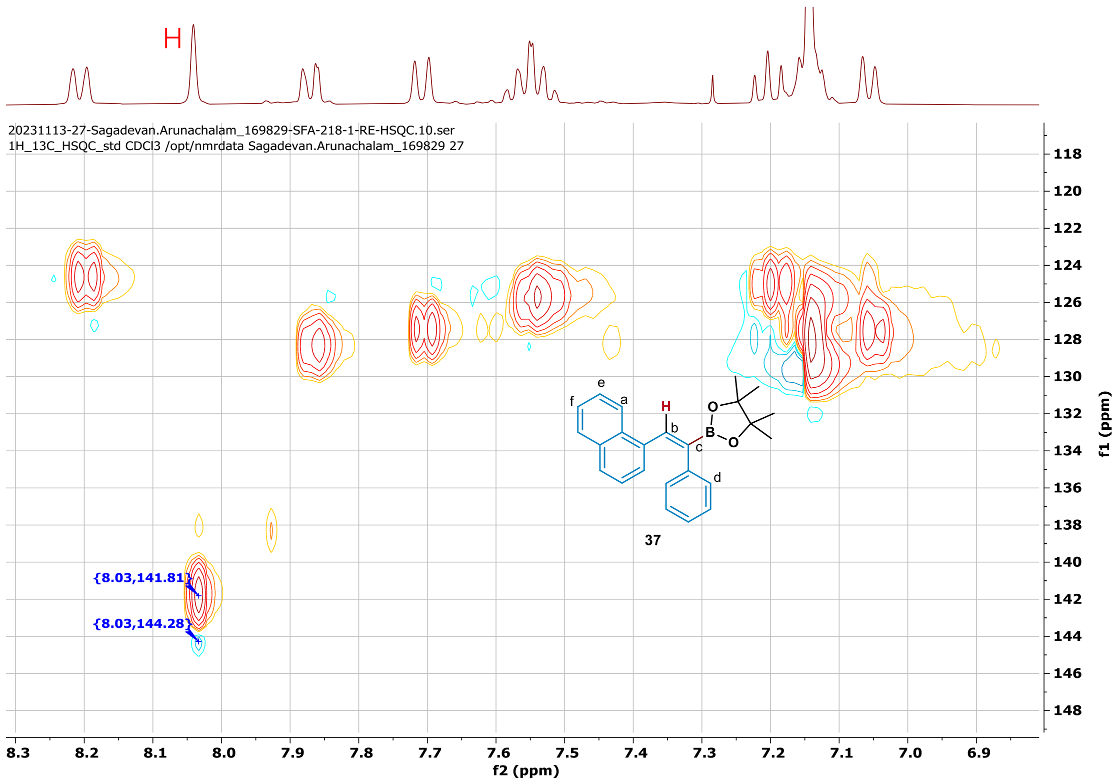
H
20231113-27-Sagadevan.Arunachalam_169829-SFA-218-1-RE-HSQC.10.ser
1H_13C_HSQC_std CDCl3 /opt/nmrdata Sagadevan.Arunachalam_169829 27
{8.03,141.81}
{8.03,144.28}
37
f2 (ppm)
f1 (ppm)

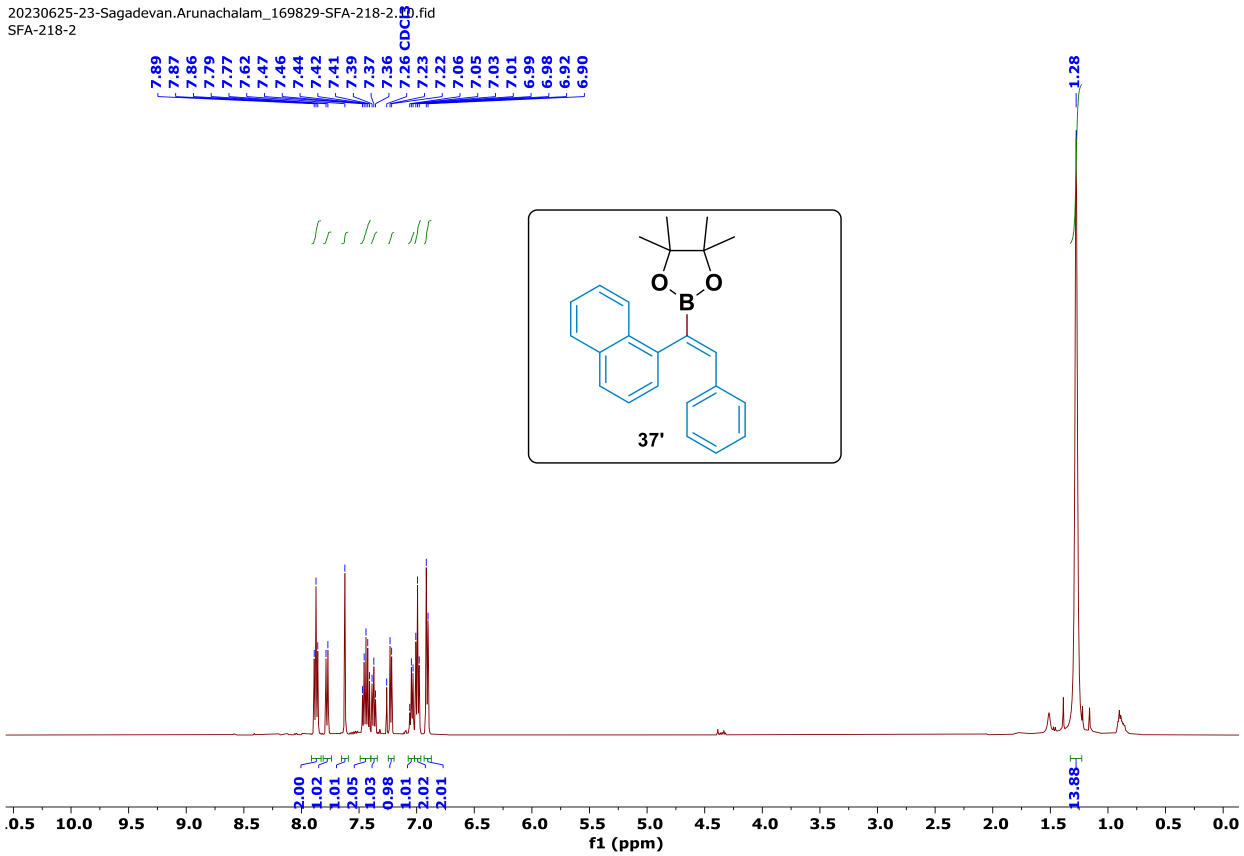
20230625-23-Sagadevan.Arunachalam_169829-SFA-218-2.10.fid
SFA-218-2
7.89 7.87 7.86 7.79 7.77 7.62 7.47 7.46 7.44 7.42 7.41 7.39 7.37 7.36 7.26 CDCl3 7.23 7.22 7.06 7.05 7.03 7.01 6.99 6.98 6.92 6.90
1.28
O B O
37'
2.00 1.02 1.01 2.05 1.03 0.98 1.01 2.02 2.01
13.88
f1 (ppm)

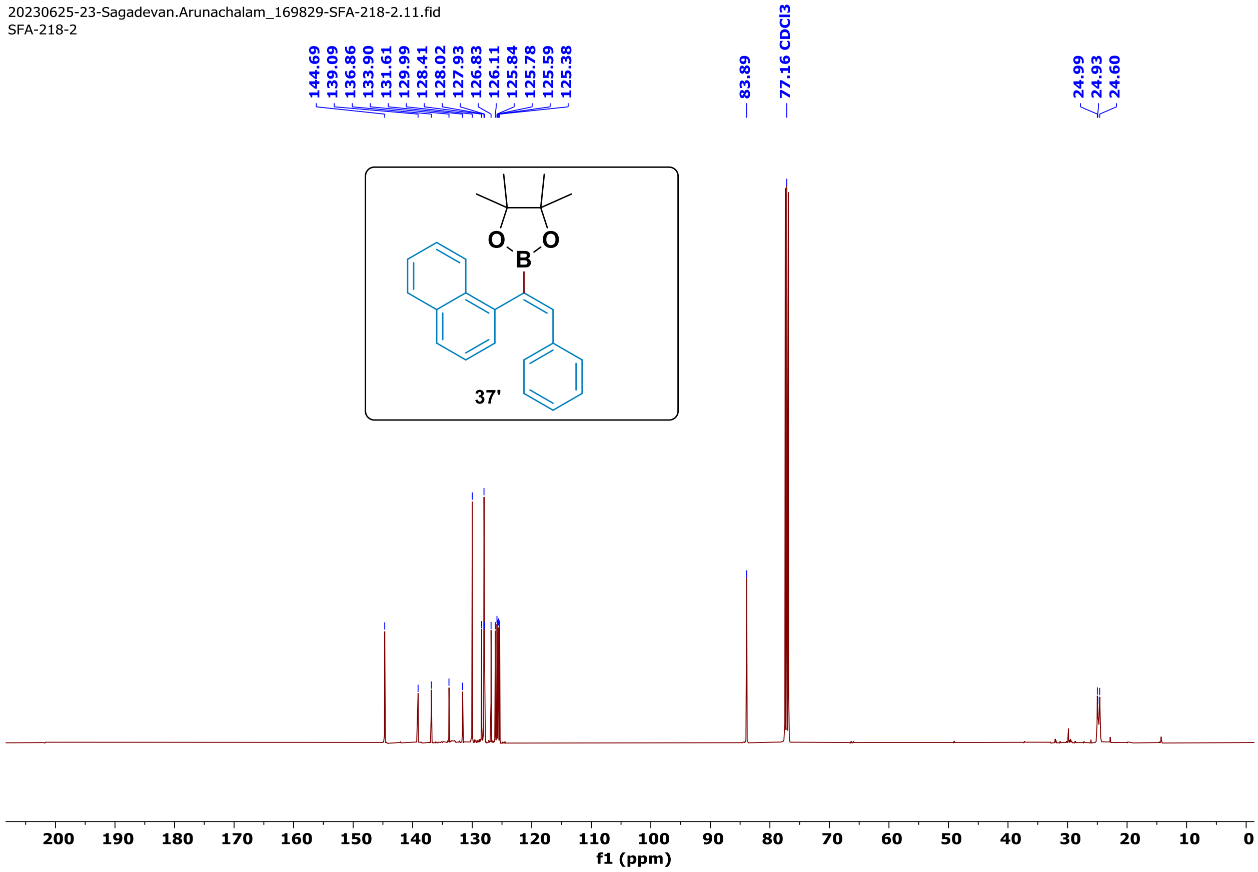
20230625-23-Sagadevan.Arunachalam_169829-SFA-218-2.11.fid
SFA-218-2
144.69 139.09 136.86 133.90 131.61 129.99 128.41 128.02 127.93 126.83 126.11 125.84 125.78 125.59 125.38
83.89
77.16 CDCl3
24.99 24.93 24.60
O B O
37'
f1 (ppm)

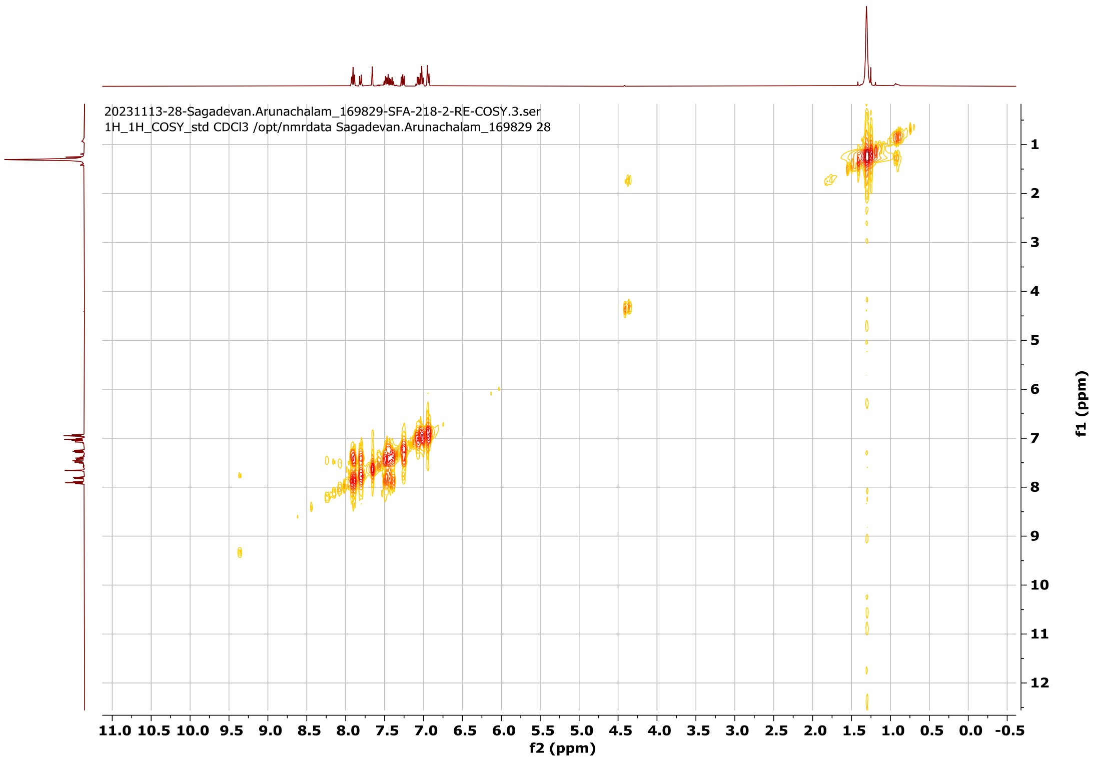
20231113-28-Sagadevan.Arunachalam_169829-SFA-218-2-RE-COSY.3.ser
1H_1H_COSY_std CDCl3 /opt/nmrdata Sagadevan.Arunachalam_169829 28
f1 (ppm)
f2 (ppm)

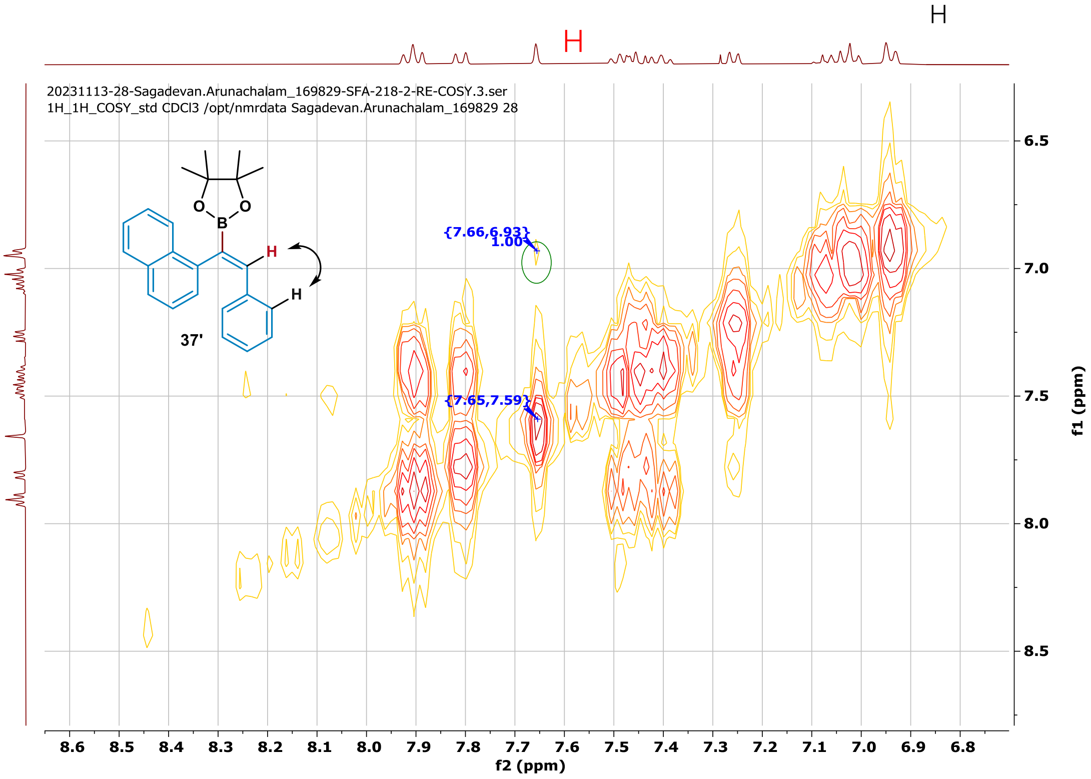
H
H
20231113-28-Sagadevan.Arunachalam_169829-SFA-218-2-RE-COSY.3.ser
1H_1H_COSY_std CDCl3 /opt/nmrdata Sagadevan.Arunachalam_169829 28
O
B
O
H
H
37'
{7.66,6.93}
1.00
{7.65,7.59}
f1 (ppm)
f2 (ppm)

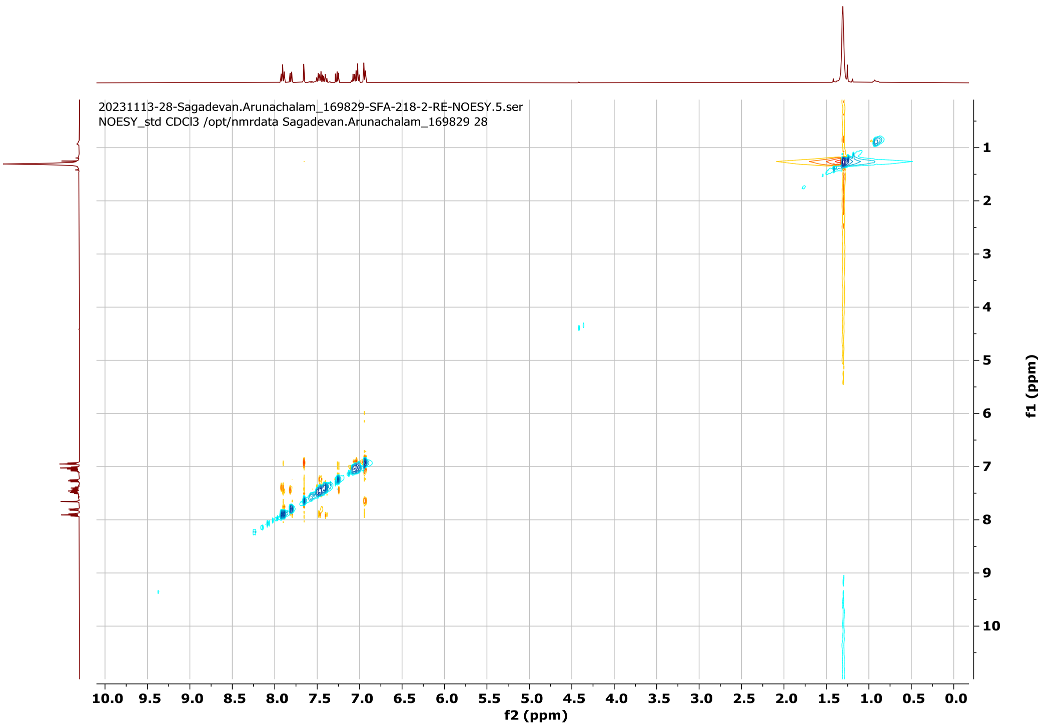
20231113-28-Sagadevan.Arunachalam_169829-SFA-218-2-RE-NOESY.5.ser
NOESY_std CDCl3 /opt/nmrdata Sagadevan.Arunachalam_169829 28
f1 (ppm)
f2 (ppm)

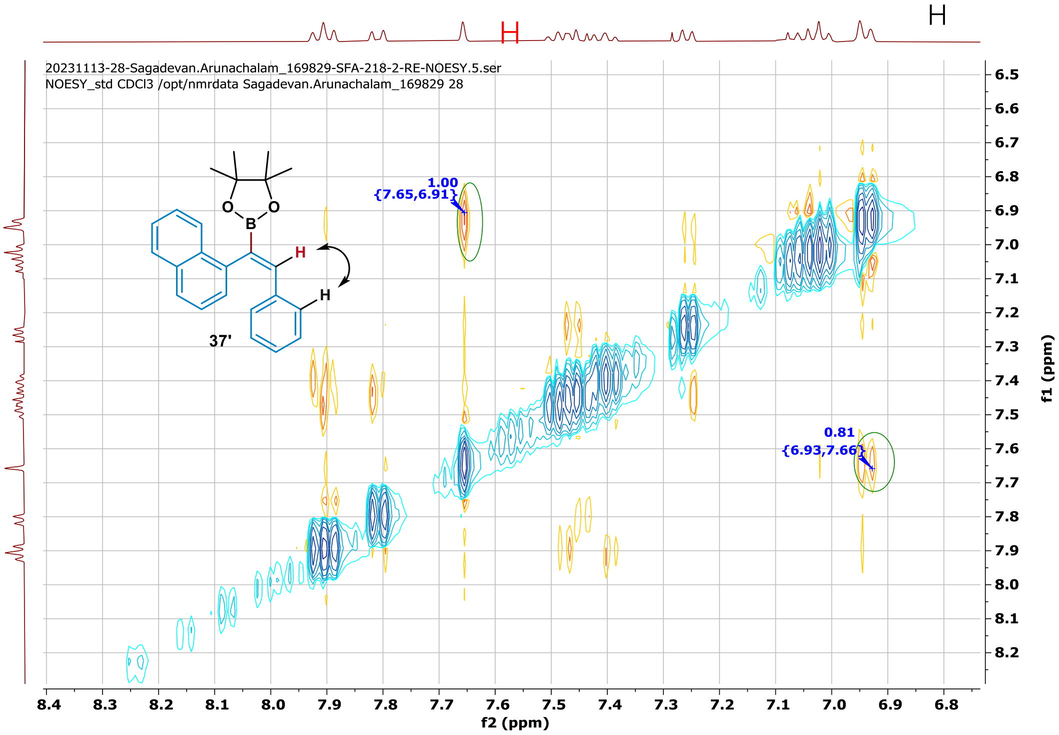
H
H
20231113-28-Sagadevan.Arunachalam_169829-SFA-218-2-RE-NOESY.5.ser
NOESY_std CDCl3 /opt/nmrdata Sagadevan.Arunachalam_169829 28
1.00
{7.65,6.91}
0.81
{6.93,7.66}
37'
f1 (ppm)
f2 (ppm)

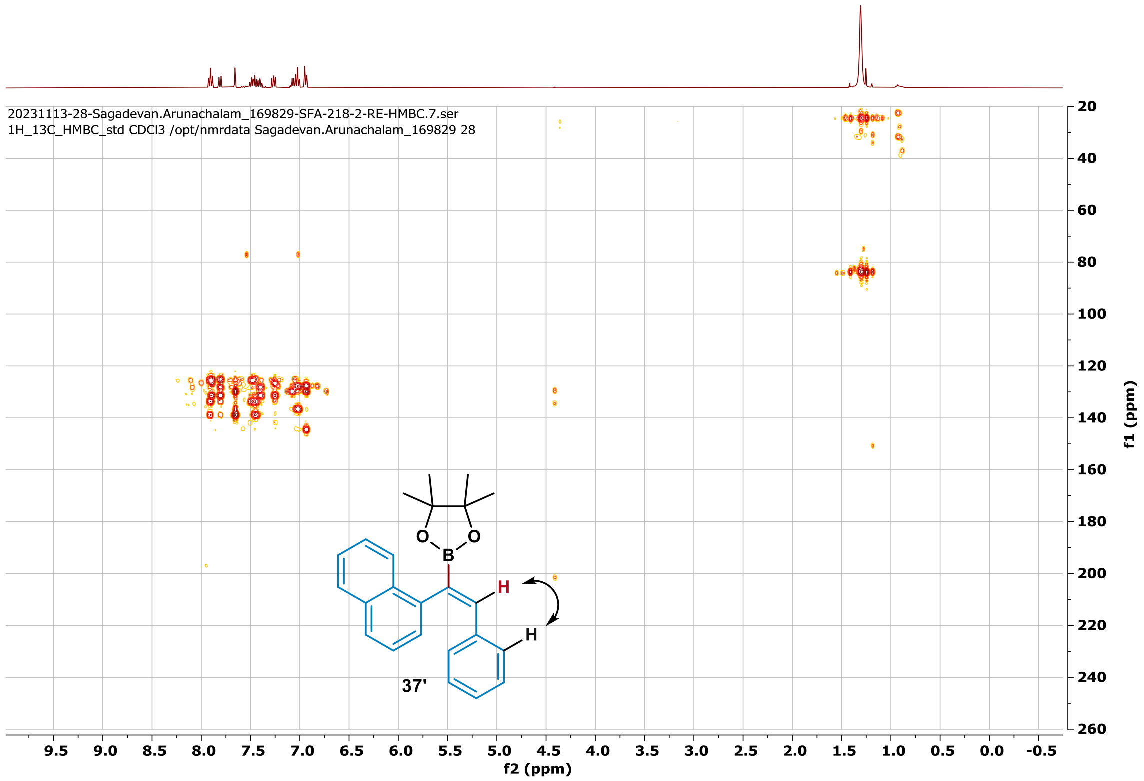
20231113-28-Sagadevan.Arunachalam_169829-SFA-218-2-RE-HMBC.7.ser
1H_13C_HMBC_std CDCl3 /opt/nmrdata Sagadevan.Arunachalam_169829 28
37'
f2 (ppm)
f1 (ppm)

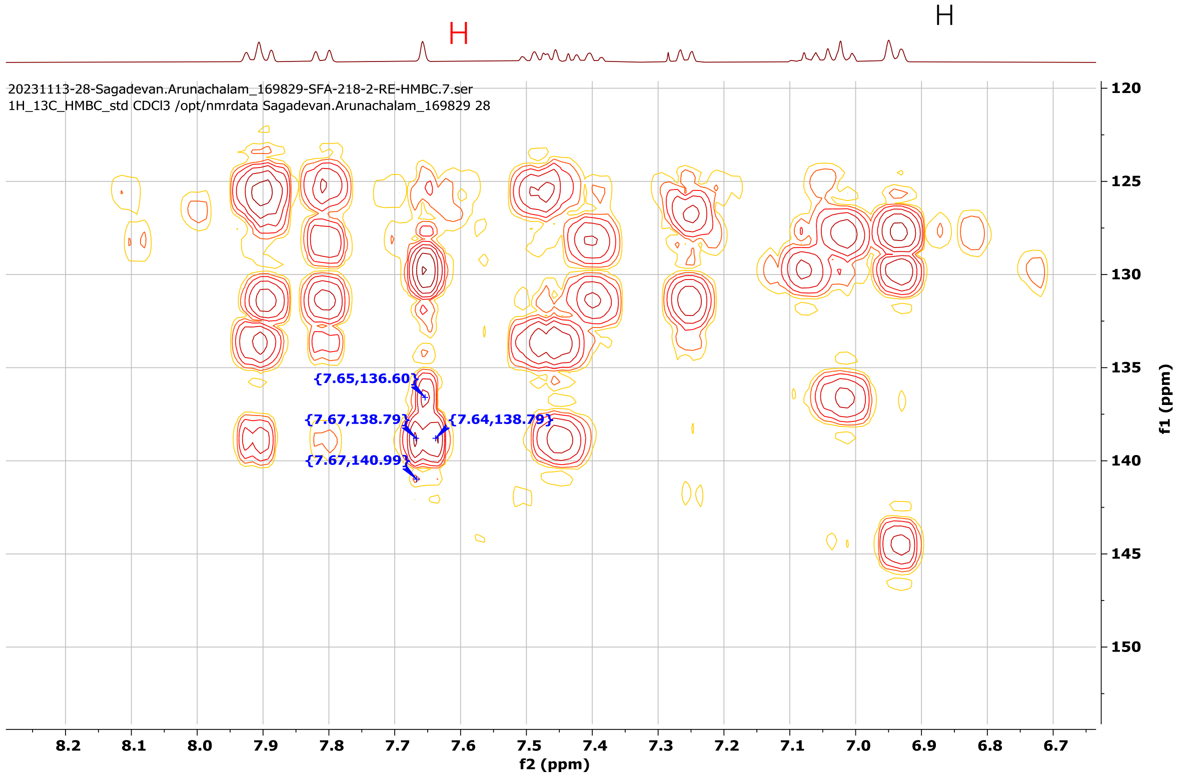
20231113-28-Sagadevan.Arunachalam_169829-SFA-218-2-RE-HMBC.7.ser
1H_13C_HMBC_std CDCl3 /opt/nmrdata Sagadevan.Arunachalam_169829 28
H
H
{7.65,136.60}
{7.67,138.79}
{7.64,138.79}
{7.67,140.99}
f2 (ppm)
f1 (ppm)

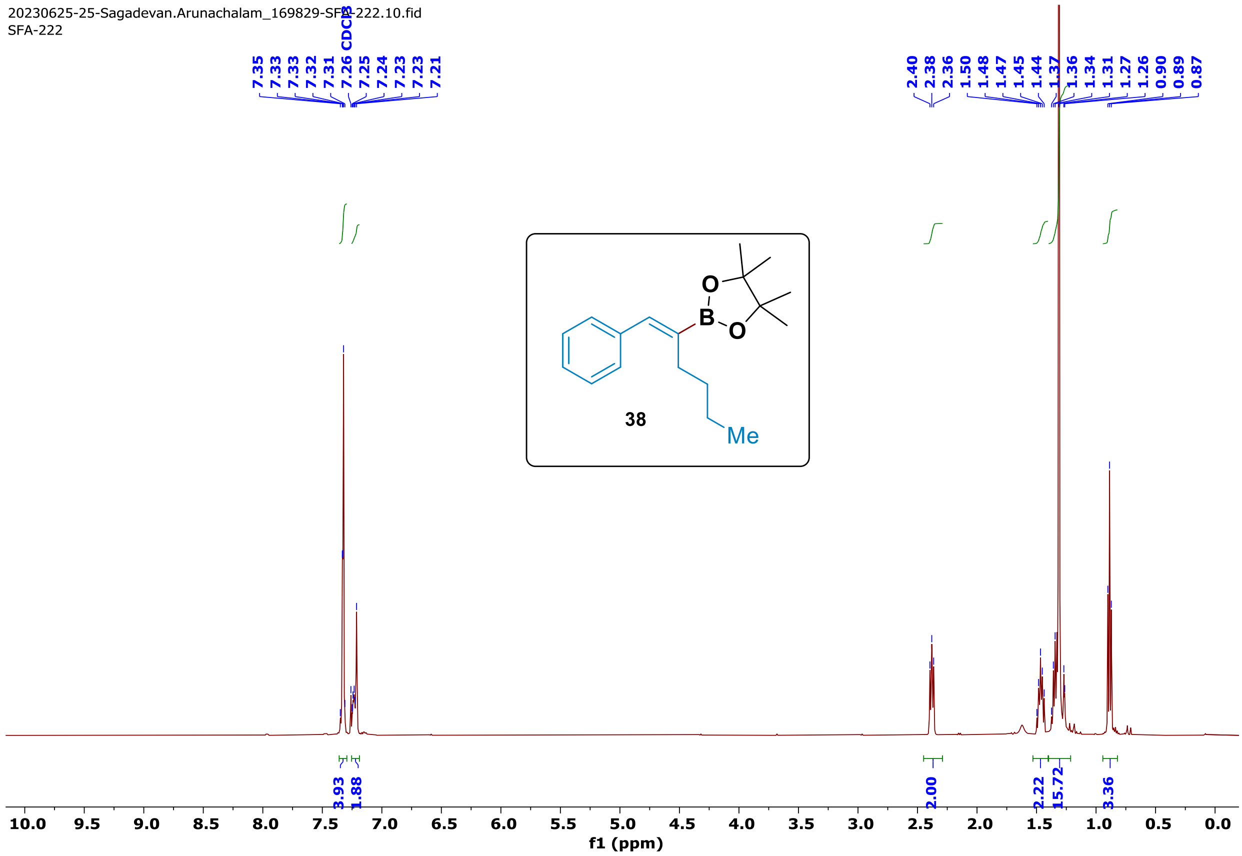
20230625-25-Sagadevan.Arunachalam_169829-SFA-222.10.fid
SFA-222
7.35 7.33 7.33 7.32 7.31 7.26 CDCl3 7.25 7.24 7.23 7.23 7.21
2.40 2.38 2.36 1.50 1.48 1.47 1.45 1.44 1.37 1.36 1.34 1.31 1.27 1.26 0.90 0.89 0.87
38
Me
3.93 1.88 2.00 2.22 15.72 3.36
f1 (ppm)

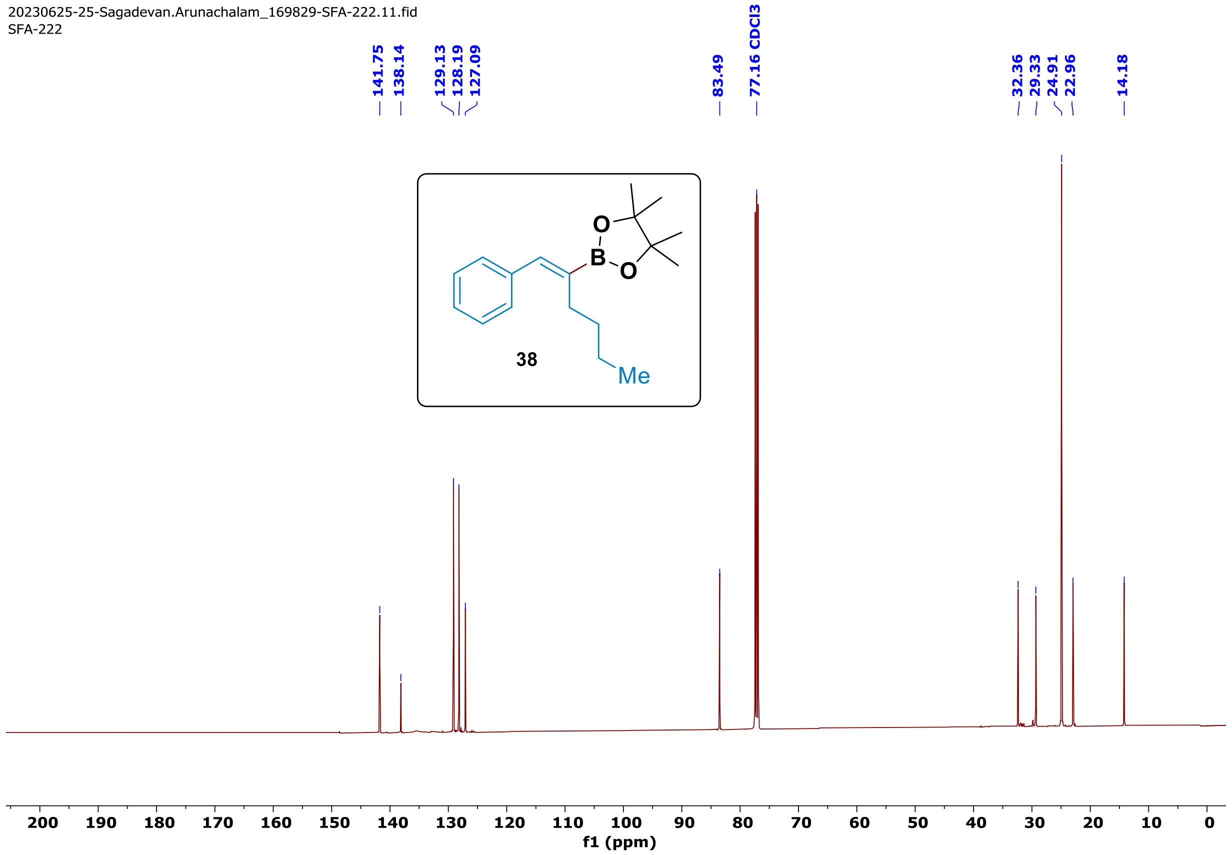
20230625-25-Sagadevan.Arunachalam_169829-SFA-222.11.fid
SFA-222
141.75 138.14 129.13 128.19 127.09 83.49 77.16 CDCl3 32.36 29.33 24.91 22.96 14.18
38
Me
f1 (ppm)

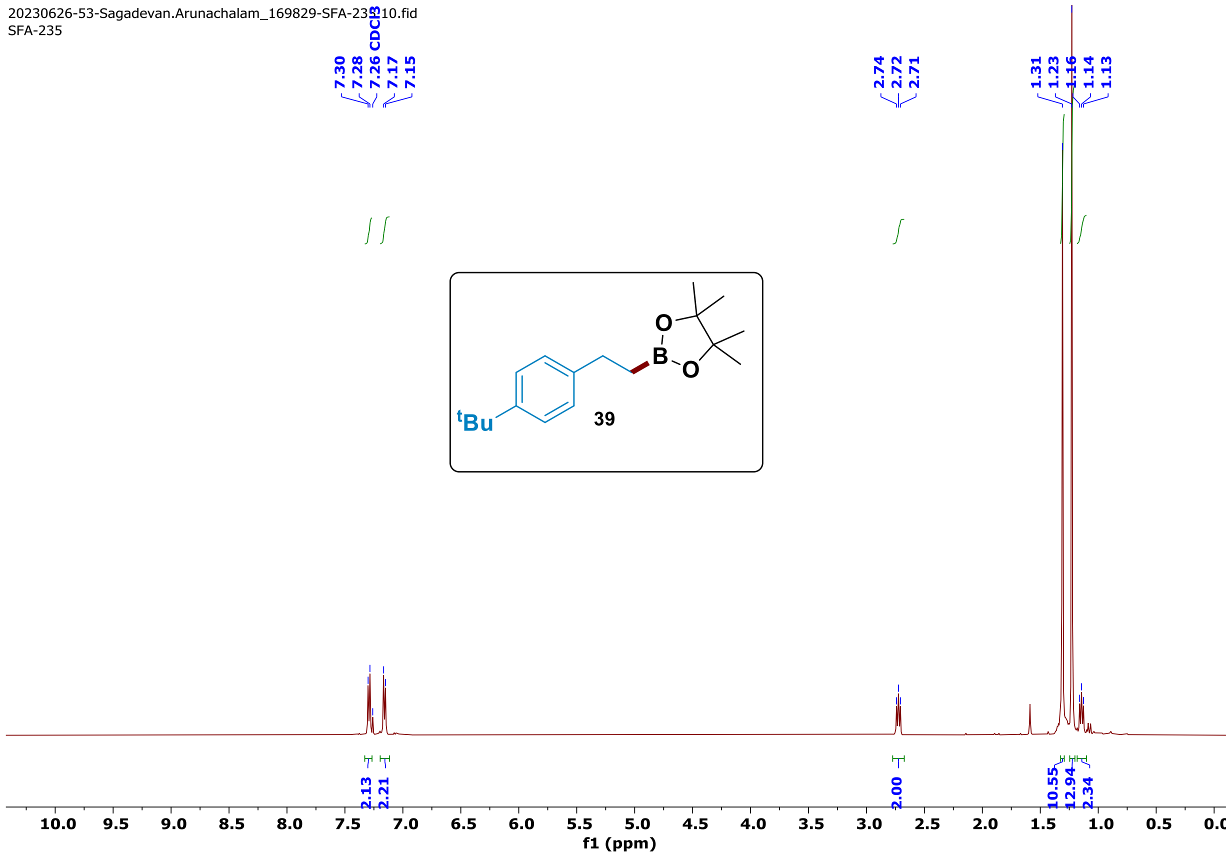
SFA-235
7.30
7.28
7.26 CDCl3
7.17
7.15
2.74
2.72
2.71
1.31
1.23
1.16
1.14
1.13
O
B
O
tBu
39
2.13
2.21
2.00
10.55
12.94
2.34
10.0 9.5 9.0 8.5 8.0 7.5 7.0 6.5 6.0 5.5 5.0 4.5 4.0 3.5 3.0 2.5 2.0 1.5 1.0 0.5
f1 (ppm)

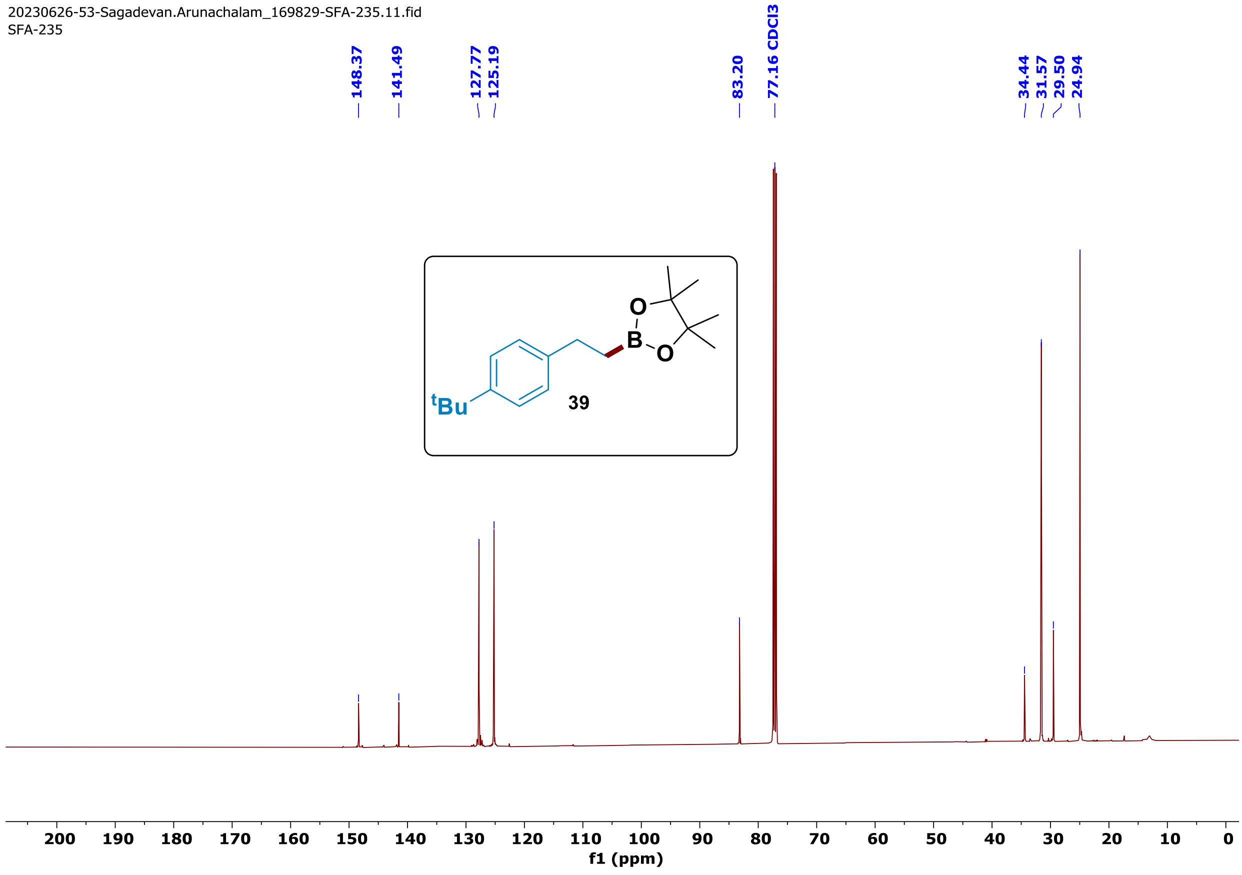
20230626-53-Sagadevan.Arunachalam_169829-SFA-235.11.fid
SFA-235
148.37
141.49
127.77
125.19
83.20
77.16 CDCl3
34.44
31.57
29.50
24.94
O
B
O
tBu
39
200 190 180 170 160 150 140 130 120 110 100 90 80 70 60 50 40 30 20 10 0
f1 (ppm)

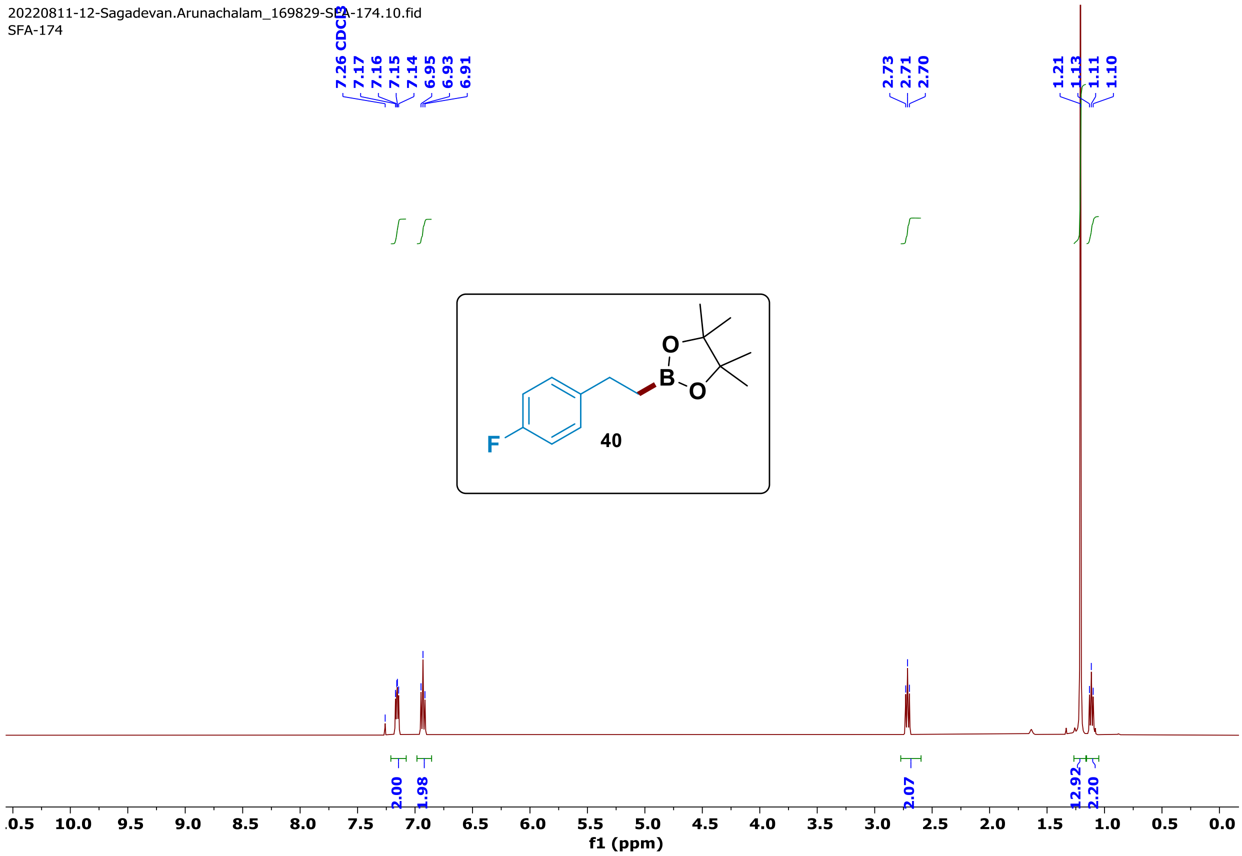
20220811-12-Sagadevan.Arunachalam_169829-SFA-174.10.fid
SFA-174
7.26 CDCl3
7.17
7.16
7.15
7.14
6.95
6.93
6.91
2.73
2.71
2.70
1.21
1.13
1.11
1.10
F
O
B
O
40
2.00
1.98
2.07
12.92
2.20
f1 (ppm)

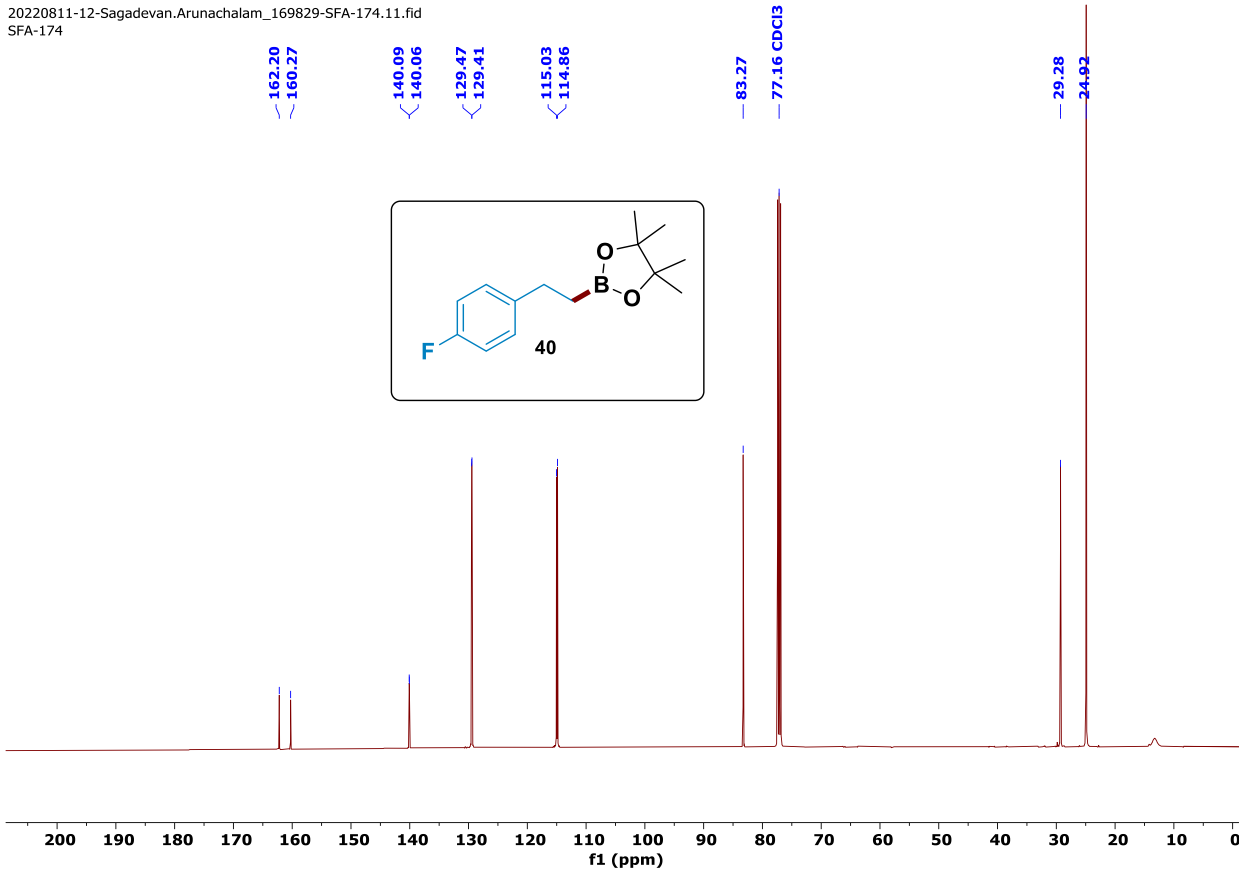
20220811-12-Sagadevan.Arunachalam_169829-SFA-174.11.fid
SFA-174
162.20
160.27
140.09
140.06
129.47
129.41
115.03
114.86
83.27
77.16 CDCl3
29.28
24.92
F
O
B
O
40
f1 (ppm)

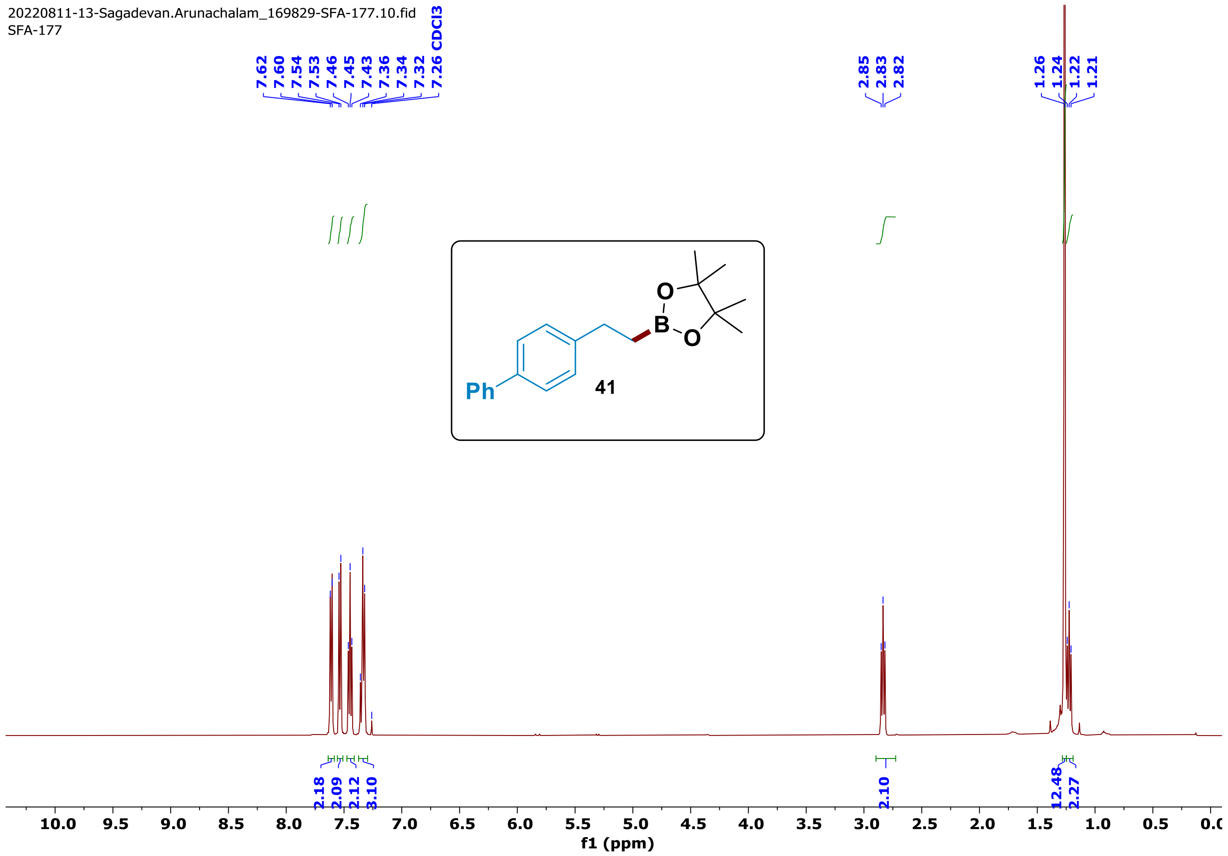
20220811-13-Sagadevan.Arunachalam_169829-SFA-177.10.fid
SFA-177
7.62 7.60 7.54 7.53 7.46 7.45 7.43 7.36 7.34 7.32 7.26 CDCl3
2.85 2.83 2.82
1.26 1.24 1.22 1.21
Ph
41
2.18 2.09 2.12 3.10
2.10
12.48 2.27
10.0 9.5 9.0 8.5 8.0 7.5 7.0 6.5 6.0 5.5 5.0 4.5 4.0 3.5 3.0 2.5 2.0 1.5 1.0 0.5
f1 (ppm)

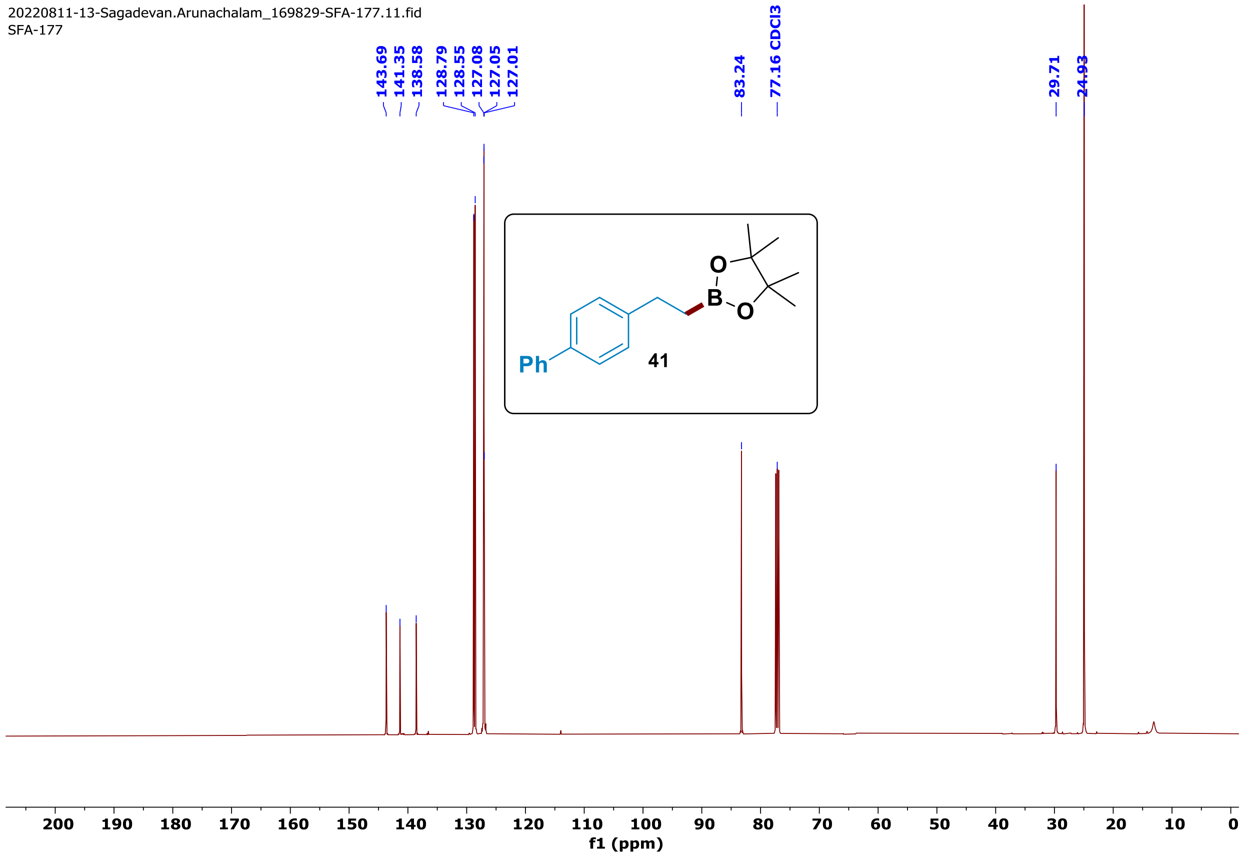
20220811-13-Sagadevan.Arunachalam_169829-SFA-177.11.fid
SFA-177
143.69 141.35 138.58
128.79 128.55 127.08 127.05 127.01
83.24
77.16 CDCl3
29.71
24.93
Ph
41
200 190 180 170 160 150 140 130 120 110 100 90 80 70 60 50 40 30 20 10 0
f1 (ppm)

20230626-57-Sagadevan.Arunachalam_169829.10.fid
SFA-232

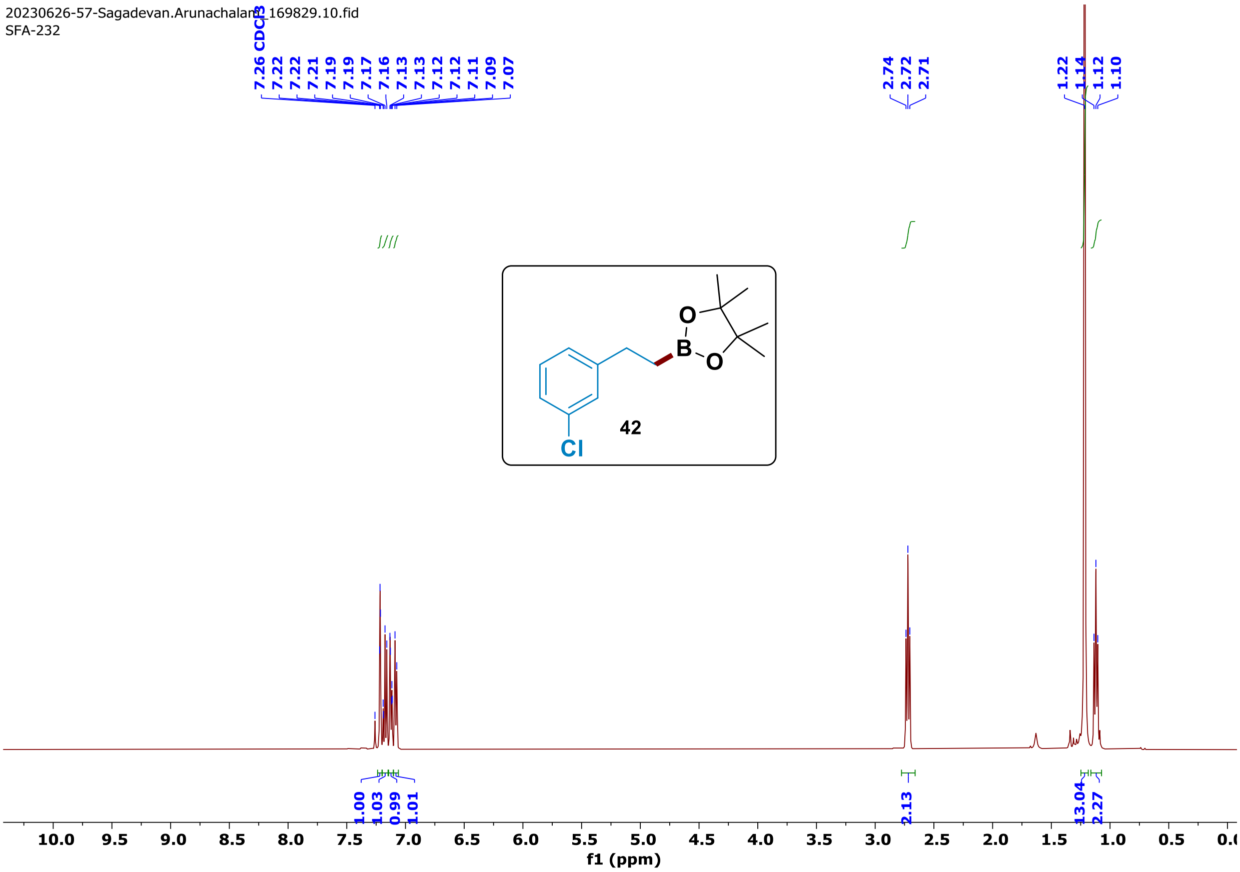

20230626-57-Sagadevan.Arunachalam_169829.11.fid
SFA-232

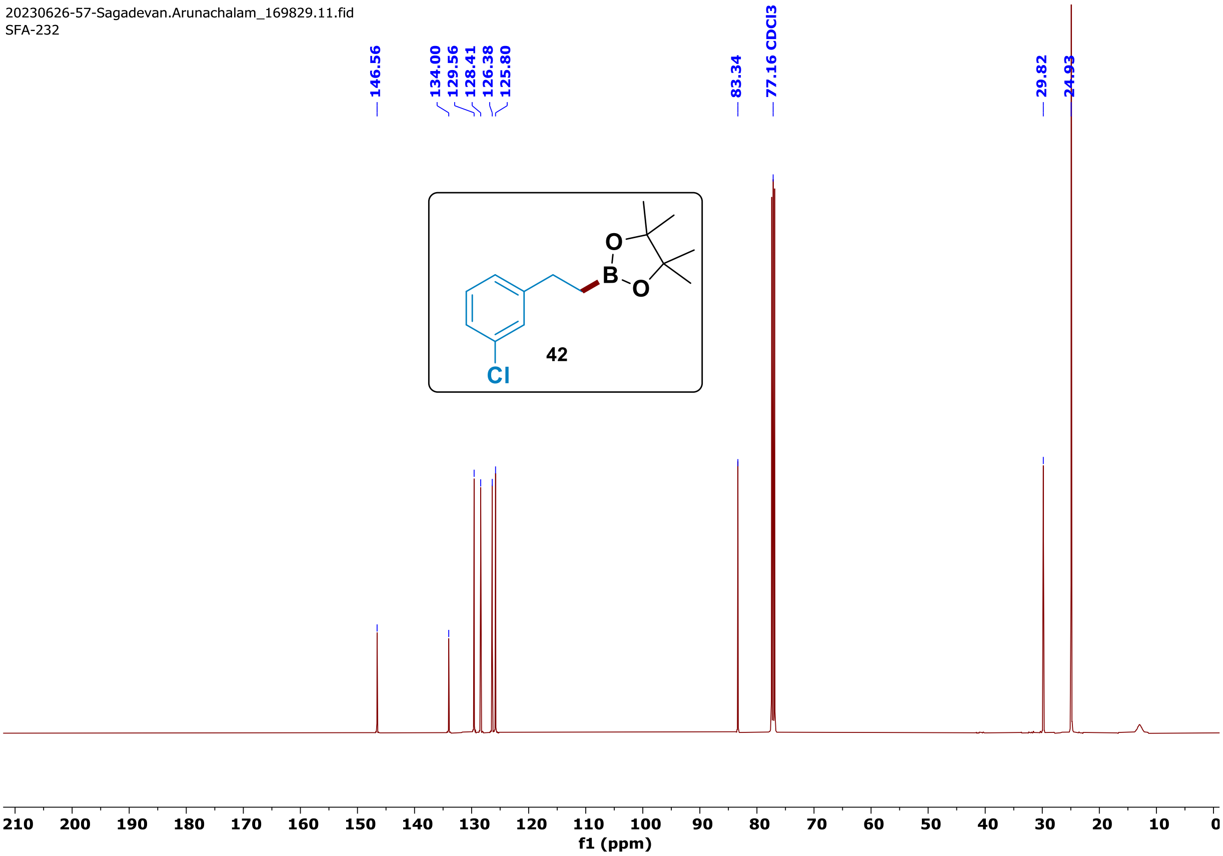

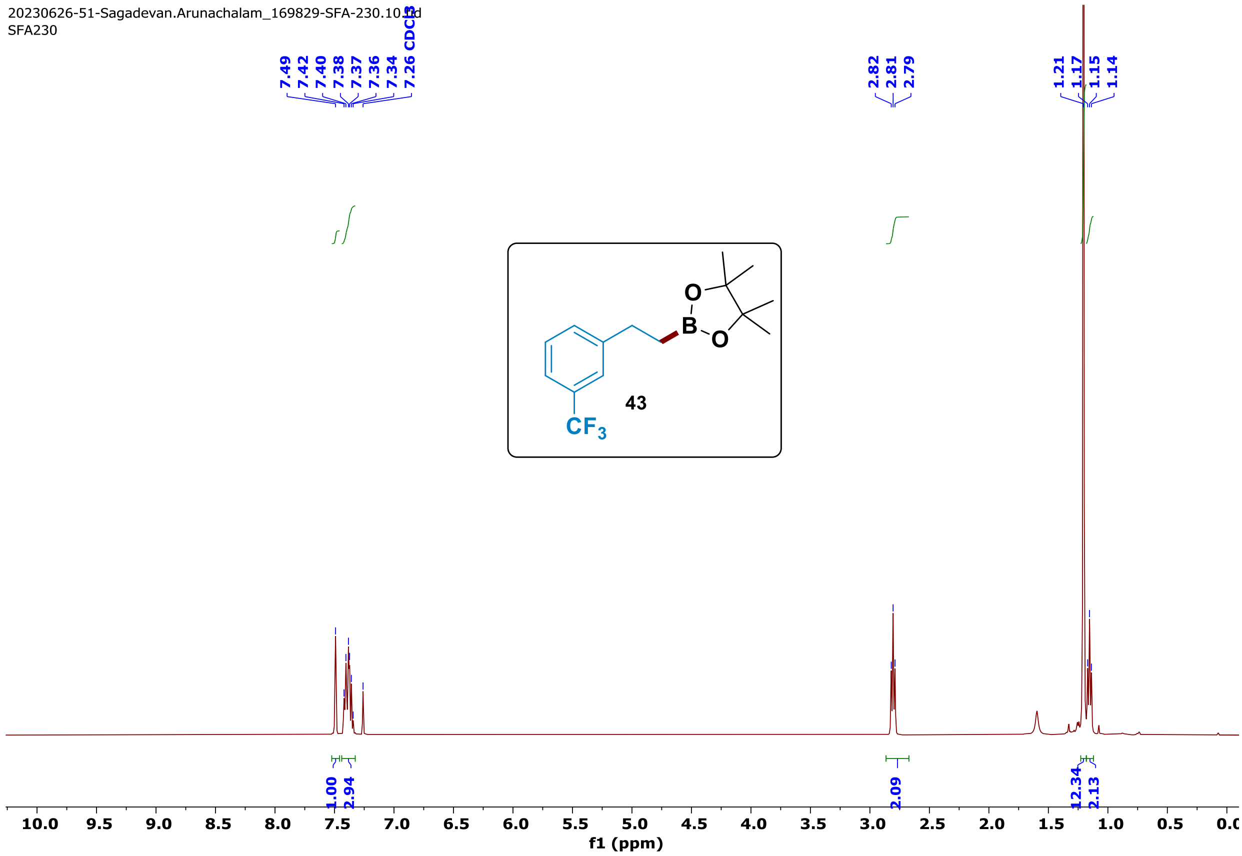
20230626-51-Sagadevan.Arunachalam_169829-SFA-230.10.fid
SFA230
7.49
7.42
7.40
7.38
7.37
7.36
7.34
7.26 CDCl3
2.82
2.81
2.79
1.21
1.17
1.15
1.14
O
B
O
CF3
43
1.00
2.94
2.09
12.34
2.13
10.0 9.5 9.0 8.5 8.0 7.5 7.0 6.5 6.0 5.5 5.0 4.5 4.0 3.5 3.0 2.5 2.0 1.5 1.0 0.5
f1 (ppm)

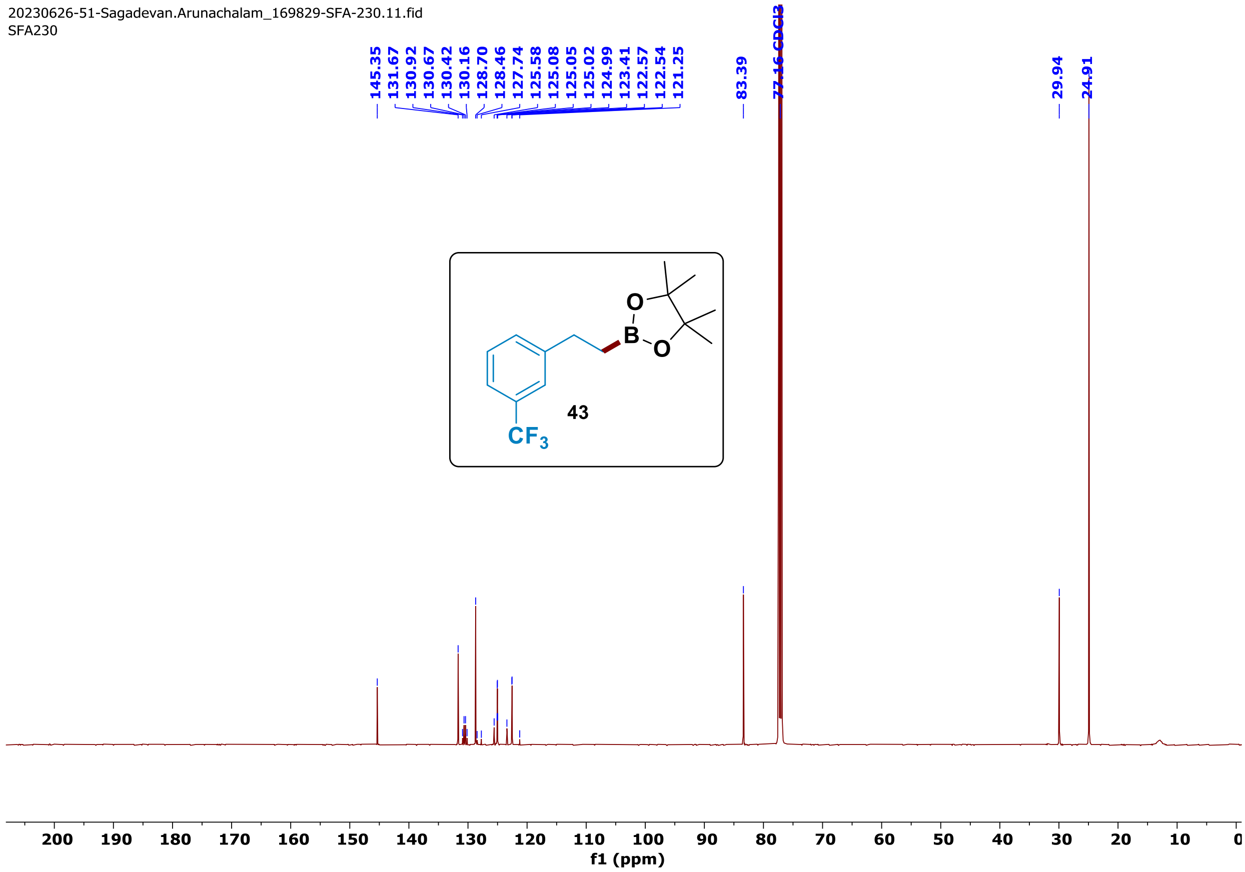
20230626-51-Sagadevan.Arunachalam_169829-SFA-230.11.fid
SFA230
145.35
131.67
130.92
130.67
130.42
130.16
128.70
128.46
127.74
125.58
125.08
125.05
125.02
124.99
123.41
122.57
122.54
121.25
83.39
77.16 CDCl3
29.94
24.91
O
B
O
CF3
43
200 190 180 170 160 150 140 130 120 110 100 90 80 70 60 50 40 30 20 10
f1 (ppm)

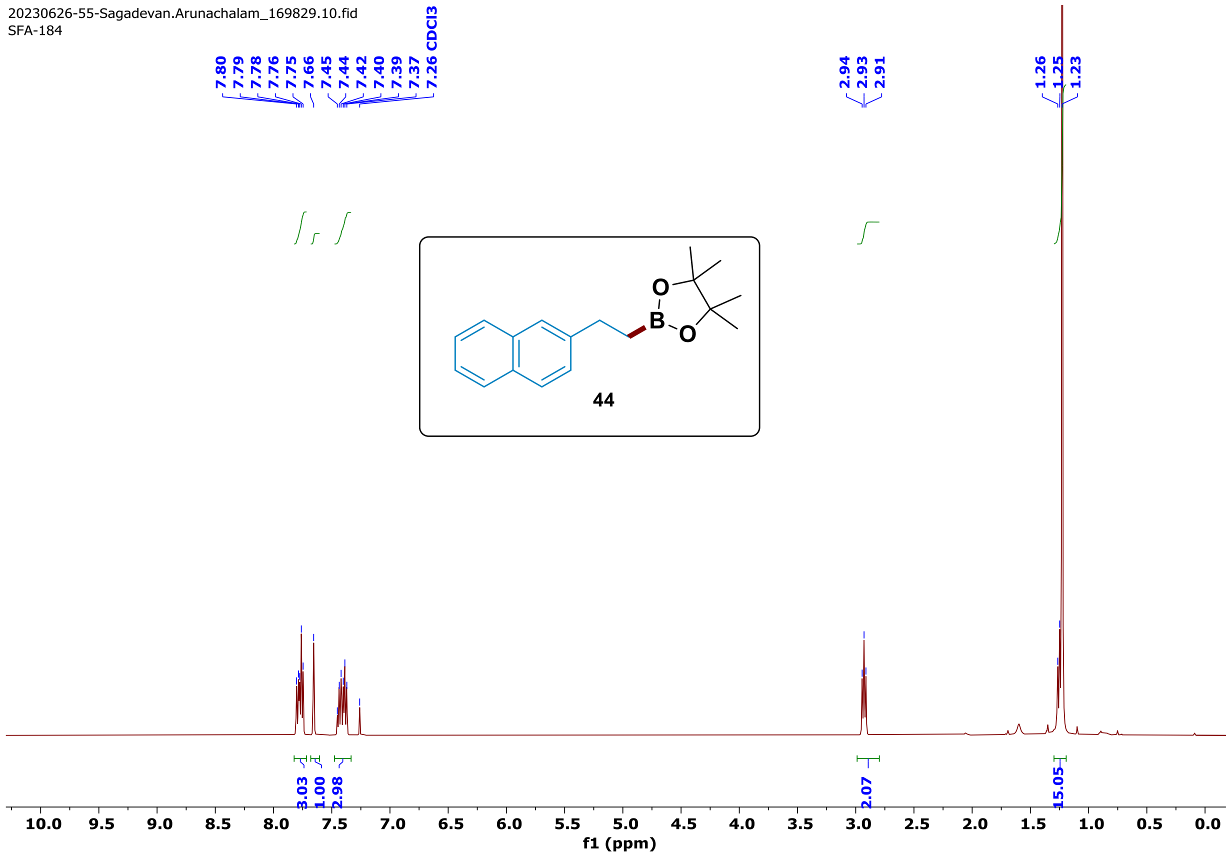
20230626-55-Sagadevan.Arunachalam_169829.10.fid
SFA-184
7.80 7.79 7.78 7.76 7.75 7.66 7.45 7.44 7.42 7.40 7.39 7.37 7.26 CDCl3
2.94 2.93 2.91
1.26 1.25 1.23
44
3.03 1.00 2.98 2.07 15.05
f1 (ppm)

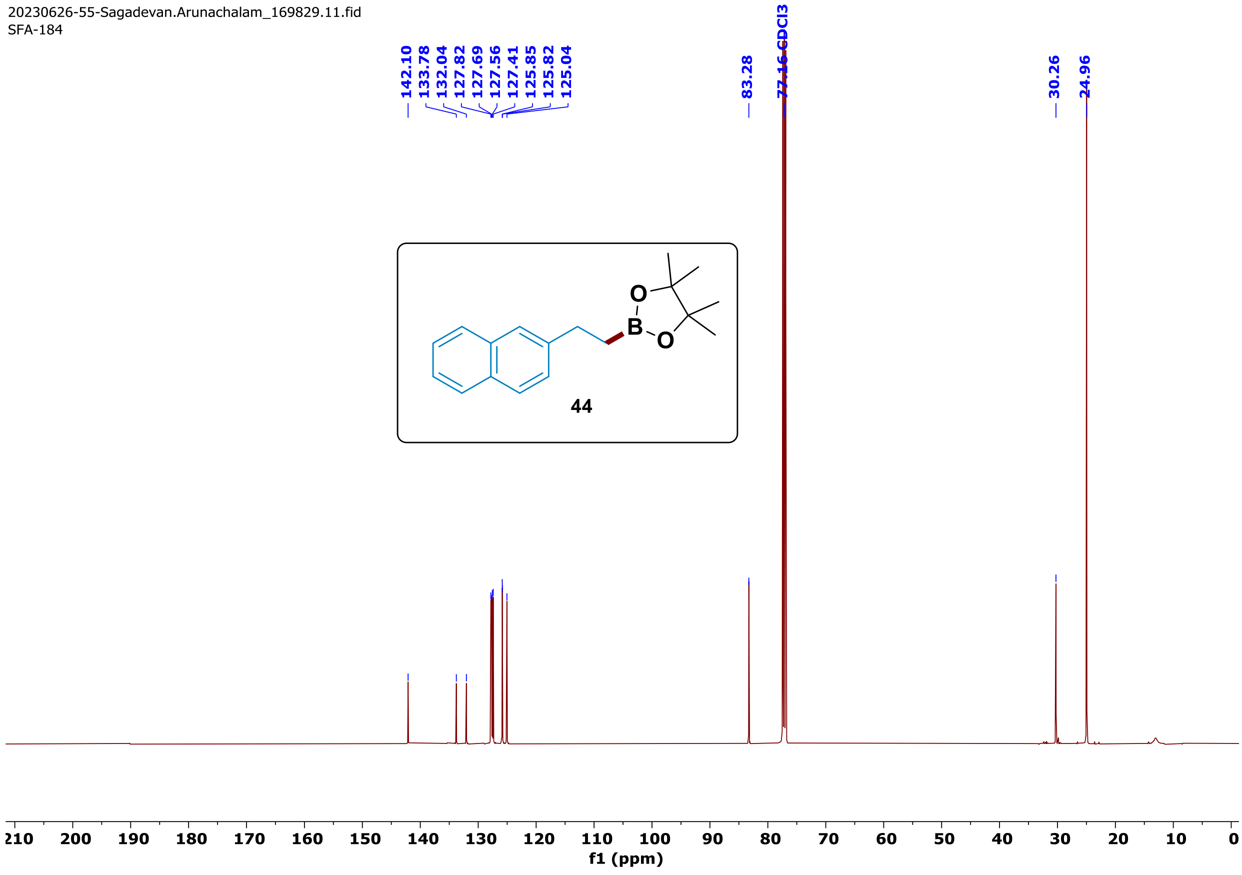
20230626-55-Sagadevan.Arunachalam_169829.11.fid
SFA-184
142.10 133.78 132.04 127.82 127.69 127.56 127.41 125.85 125.82 125.04
83.28
77.16 CDCl3
30.26
24.96
44
f1 (ppm)

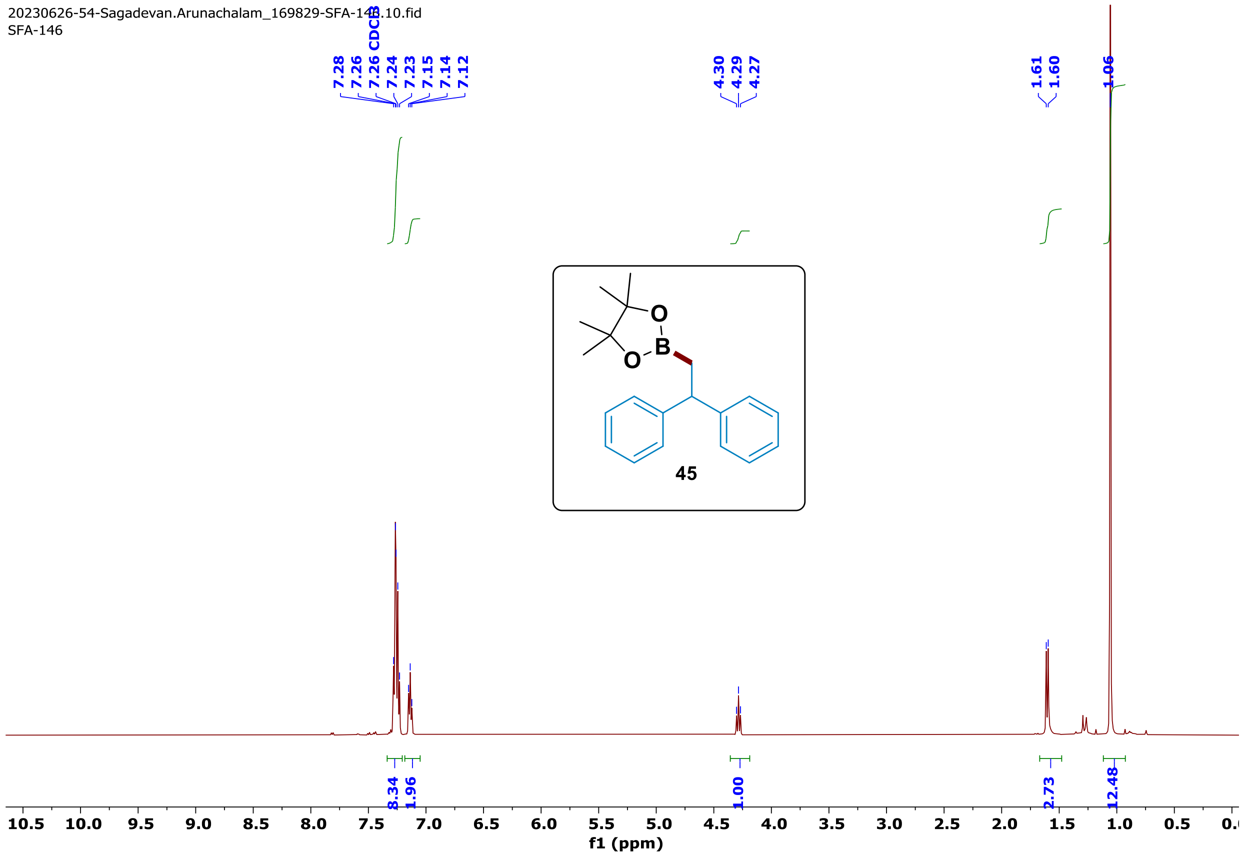
SFA-146
7.28
7.26
7.26 CDCl3
7.24
7.23
7.15
7.14
7.12
4.30
4.29
4.27
1.61
1.60
1.06
45
8.34
1.96
1.00
2.73
12.48
10.5 10.0 9.5 9.0 8.5 8.0 7.5 7.0 6.5 6.0 5.5 5.0 4.5 4.0 3.5 3.0 2.5 2.0 1.5 1.0 0.5
f1 (ppm)

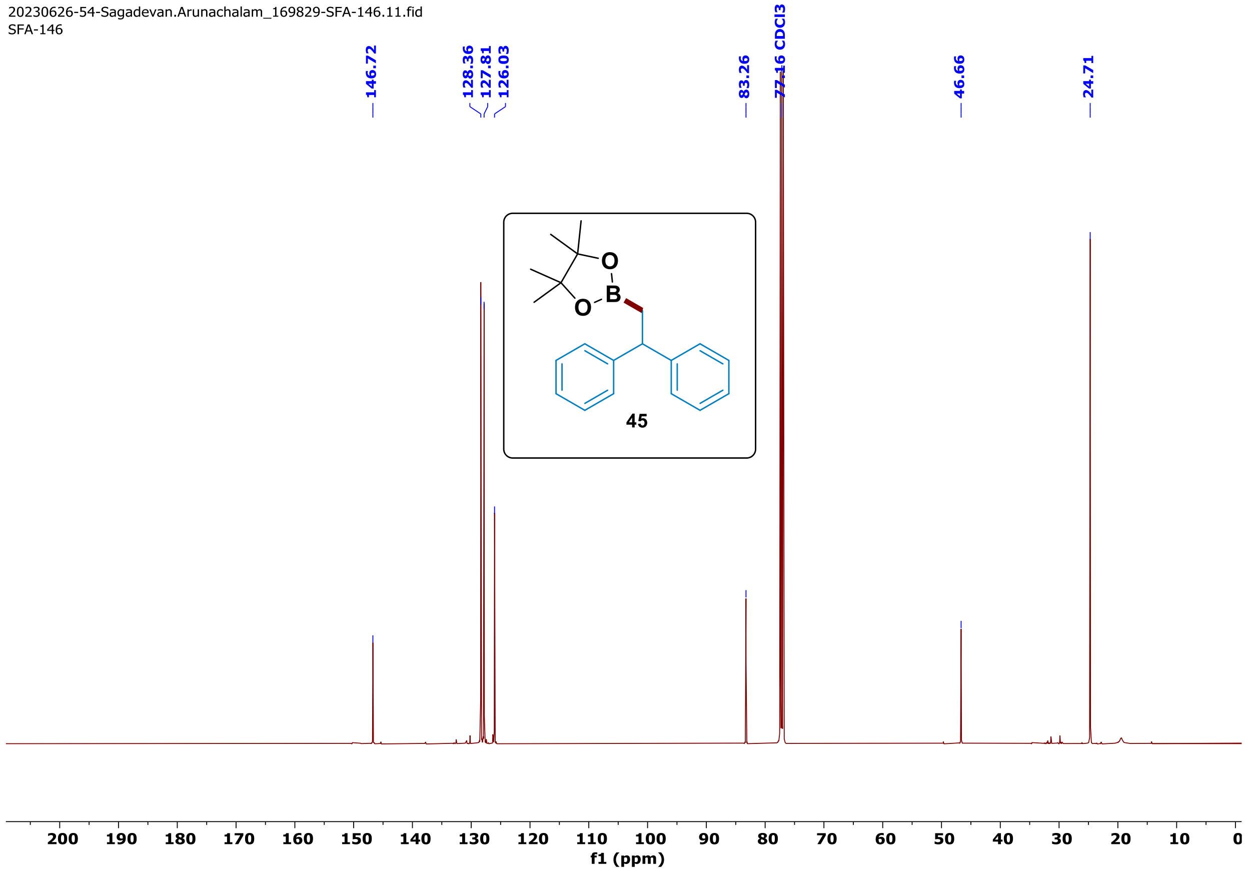
20230626-54-Sagadevan.Arunachalam_169829-SFA-146.11.fid
SFA-146
146.72
128.36
127.81
126.03
83.26
77.16 CDCl3
46.66
24.71
45
200 190 180 170 160 150 140 130 120 110 100 90 80 70 60 50 40 30 20 10 0
f1 (ppm)

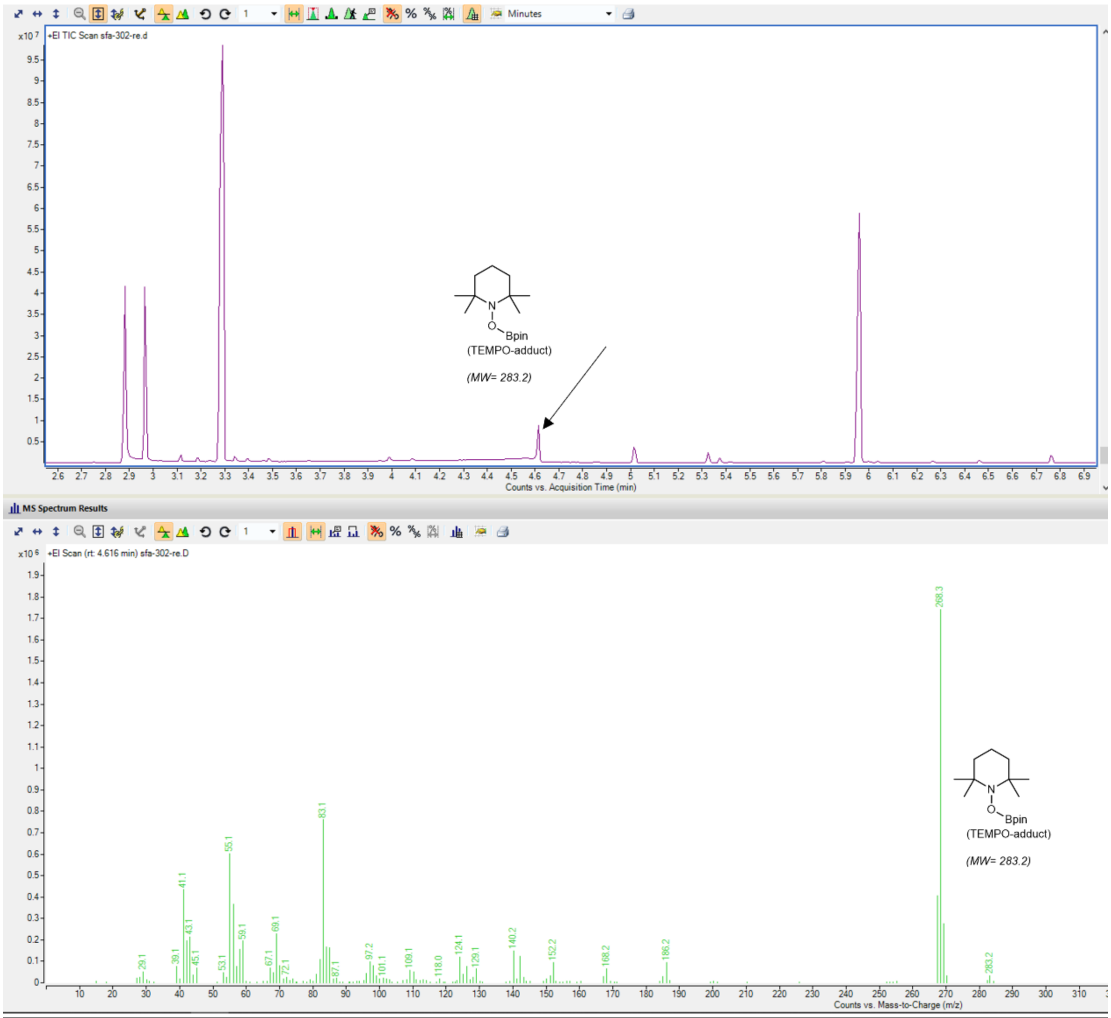

Figure S22: GC/MS spectra of TEMPO radical quenching experiment.

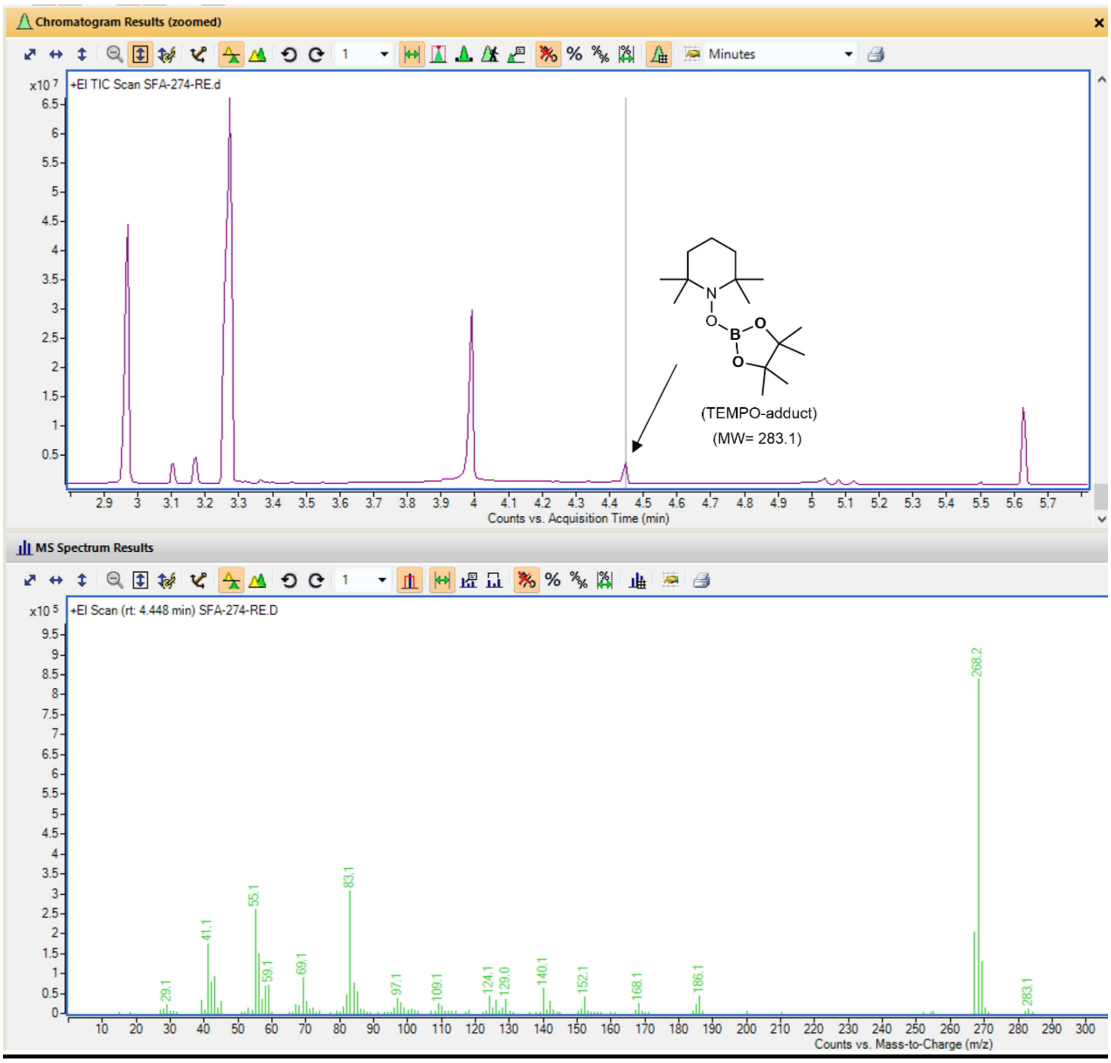

Figure S23: GC/MS spectra of TEMPO radical quenching experiment.

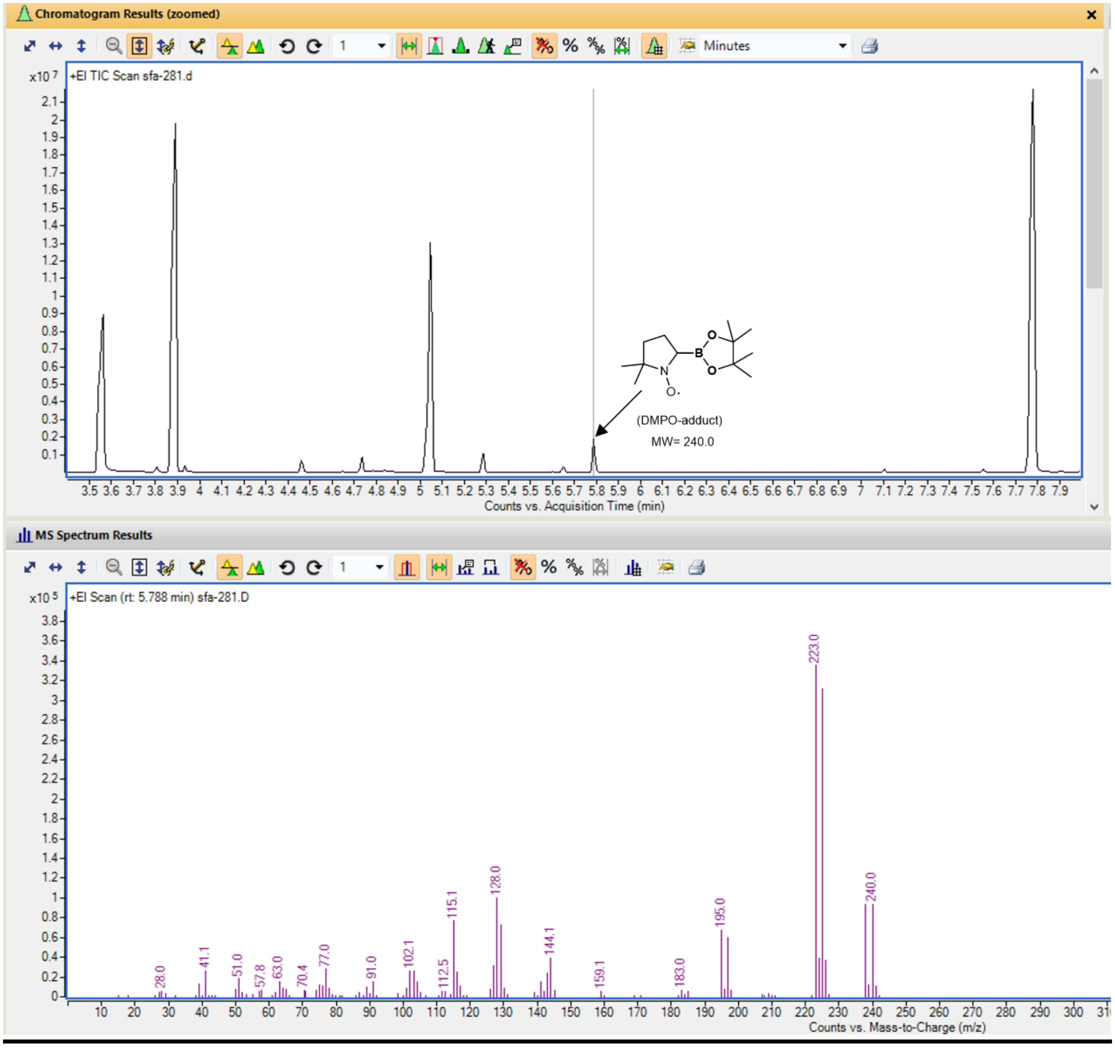

Figure S24: GC/MS spectra of DMPO radical quenching experiment.

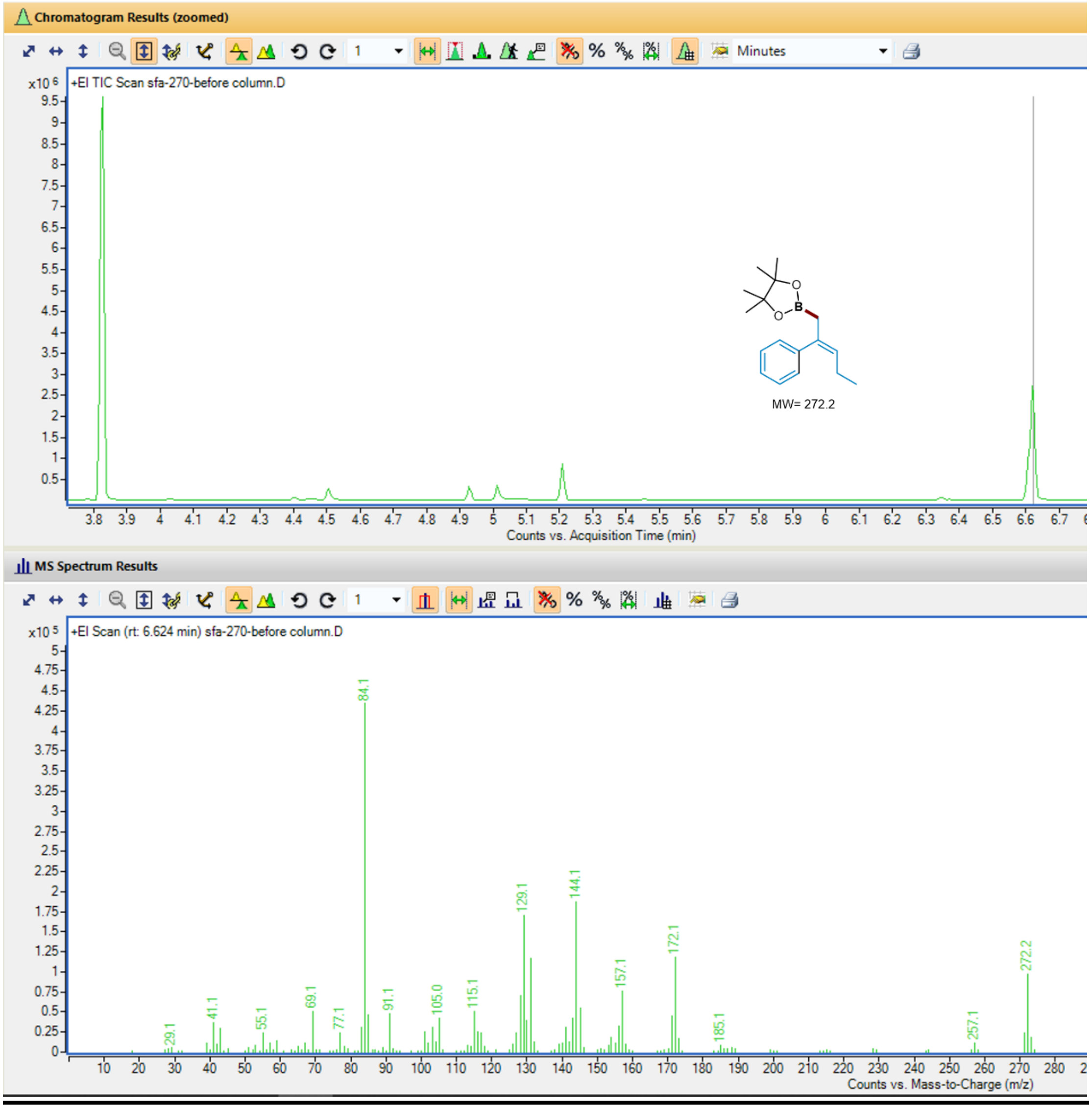

Figure S25: GC/MS spectra of radical clock experiment.

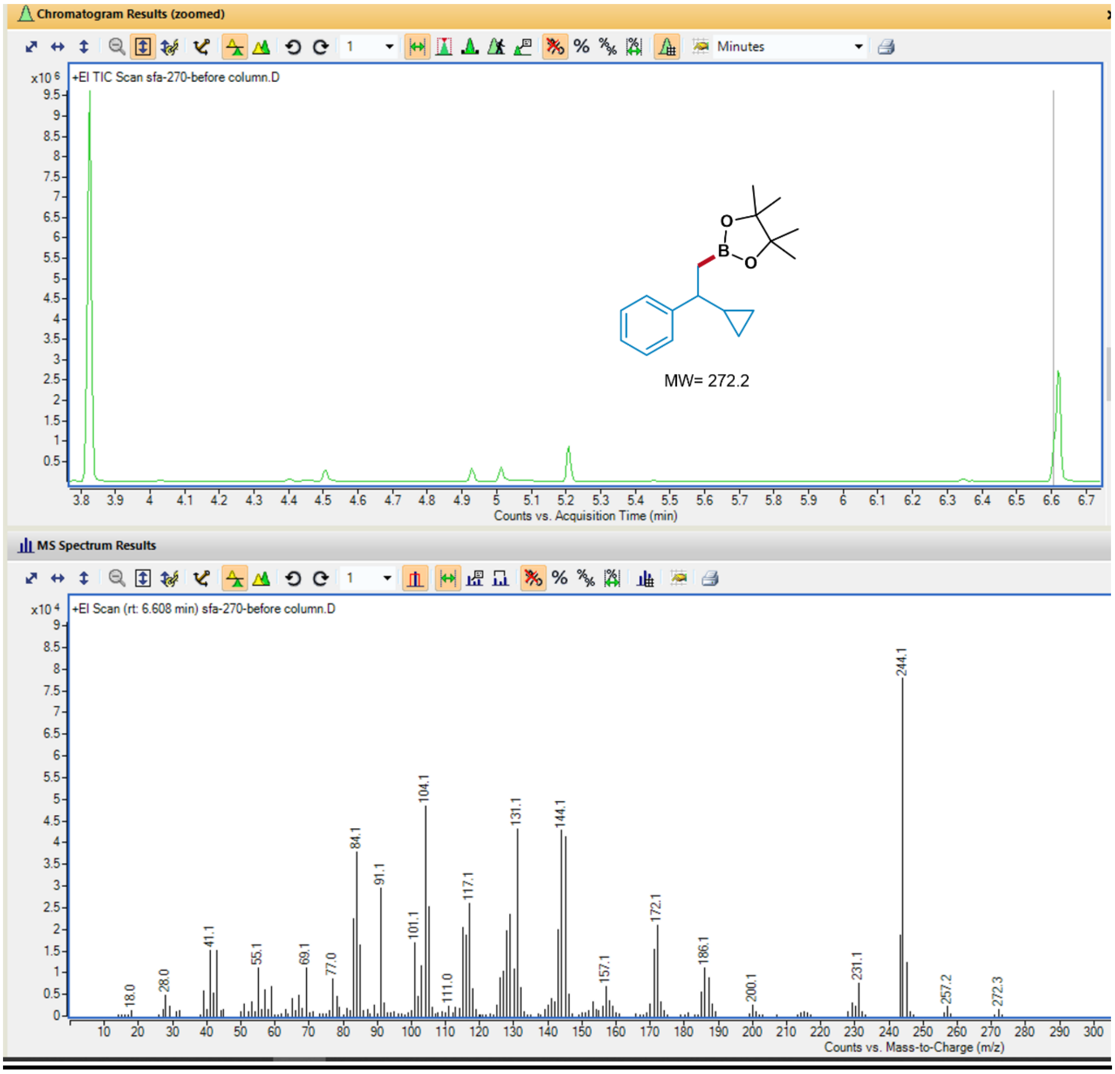

Figure S26: GC/MS spectra of radical clock experiment.

Supporting information References:

1. Zhong, M.; Gagné, Y.; Hope, T. O.; Pannecoucke, X.; Frenette, M.; Jubault, P.; Poisson, T., Copper-Photocatalyzed Hydroboration of Alkynes and Alkenes. *Angew. Chem., Int. Ed.* **2021,** *60*, 14498-14503.
2. Aelterman, M.; Sayes, M.; Jubault, P.; Poisson, T., Electrochemical Hydroboration of Alkynes. *Chem. Eur. J.* **2021,** *27*, 8277-8282.
3. Bhawar, R.; Saini, S.; Nagaraju, D. H.; Bose, S. K., CeO2-Nanorods-Catalyzed Protoboration of Alkenes and Alkynes with Bis(pinacolato)diboron. *Adv. Synth. Catal.* **2023,** *365*, 584-593.
4. Yasu, Y.; Koike, T.; Akita, M., Visible-light-induced synthesis of a variety of trifluoromethylated alkenes from potassium vinyltrifluoroborates by photoredox catalysis. *Chem. Commun.* **2013,** *49*, 2037-2039.
5. Liu, J.; Wu, C.; Hu, T.; Yang, W.; Xie, Y.; Shi, Y.; Liu, Q.; Shao, Y.; Zhang, F., Hexamethyldisilazane Lithium (LiHMDS)-Promoted Hydroboration of Alkynes and Alkenes with Pinacolborane. *J. Org. Chem.* **2022,** *87*, 3442-3452.
6. Zhang, G.; Zeng, H.; Zheng, S.; Neary, M. C.; Dub, P. A., Vanadium-Catalyzed Stereo- and Regioselective Hydroboration of Alkynes to Vinyl Boronates. *ACS Catal.* **2022,** *12*, 5425-5429.
7. Tai, C.-C.; Yu, M.-S.; Chen, Y.-L.; Chuang, W.-H.; Lin, T.-H.; Yap, G. P. A.; Ong, T.-G., Synthesis of a guanidine NHC complex and its application in borylation reactions. *Chem. Commun.* **2014,** *50*, 4344-4346.
8. Birepinte, M.; Liautard, V.; Chabaud, L.; Pucheault, M., Zirconium-Catalyzed Synthesis of Alkenylaminoboranes: From a Reliable Preparation of Alkenylboronates to a Direct Stereodivergent Access to Alkenyl Bromides. *Org. Lett.* **2020,** *22*, 2838-2843.
9. González, M. J.; Bauer, F.; Breit, B., Cobalt-Catalyzed Hydroboration of Terminal and Internal Alkynes. *Org. Lett.* **2021,** *23*, 8199-8203.
10. Zhu, W.; Zhang, S.; Fan, W.; Yang, Y.; Zhao, H.; Fei, W.; Bi, H.; He, J.; Li, M.-B.; Wu, Z., Atomically Precise Metal Nanoclusters as Single Electron Transferers for Hydroborylation. *Precis. Chem.* **2023,** *1*, 175-182.
11. Fleige, M.; Möbus, J.; vom Stein, T.; Glorius, F.; Stephan, D. W., Lewis acid catalysis: catalytic hydroboration of alkynes initiated by Piers' borane. *Chem. Commun.* **2016,** *52*, 10830-10833.
12. Haberberger, M.; Enthaler, S., Straightforward Iron-Catalyzed Synthesis of Vinylboronates by the Hydroboration of Alkynes. *Chem. Asian J.* **2013,** *8*, 50-54.
13. Rami, F.; Bächtle, F.; Plietker, B., Hydroboration of internal alkynes catalyzed by FeH(CO)(NO)(PPh3)2: a case of boron-source controlled regioselectivity. *Catal. Sci. Technol.* **2020,** *10*, 1492-1497.
14. Zhou, Y.; You, W.; Smith, K. B.; Brown, M. K., Copper-Catalyzed Cross-Coupling of Boronic Esters with Aryl Iodides and Application to the Carboboration of Alkynes and Allenes. *Angew. Chem., Int. Ed.* **2014,** *53*, 3475-3479.
15. Ansell, M. B.; Menezes da Silva, V. H.; Heerdt, G.; Braga, A. A. C.; Spencer, J.; Navarro, O., An experimental and theoretical study into the facile, homogenous (N-heterocyclic carbene)2-Pd(0) catalyzed diboration of internal and terminal alkynes. *Catal. Sci. Technol.* **2016,** *6*, 7461-7467.

16. Semba, K.; Fujihara, T.; Terao, J.; Tsuji, Y., Copper-Catalyzed Highly Regio- and Stereoselective Directed Hydroboration of Unsymmetrical Internal Alkynes: Controlling Regioselectivity by Choice of Catalytic Species. *Chem. Eur. J.* **2012,** *18*, 4179-4184.
17. Harinath, A.; Banerjee, I.; Bhattacharjee, J.; Panda, T. K., Aluminium complex-catalysed hydroboration of alkenes and alkynes. *New J. Chem.* **2019,** *43*, 10531-10536.
18. Saini, S.; Gavali, D. S.; Bhawar, R.; Thapa, R.; Dhayal, R. S.; Bose, S. K., Facile synthesis of alkyl- and arylboronate esters enabled by a carbon nanotube supported copper catalyst. *Catal. Sci. Technol.* **2023,** *13*, 147-156.
